# A reaction mode of carbene-catalysed aryl aldehyde activation and induced phenol OH functionalization

**DOI:** 10.1038/ncomms15598

**Published:** 2017-05-25

**Authors:** Xingkuan Chen, Hongling Wang, Kazuki Doitomi, Chong Yih Ooi, Pengcheng Zheng, Wangsheng Liu, Hao Guo, Song Yang, Bao-An Song, Hajime Hirao, Yonggui Robin Chi

**Affiliations:** 1 Division of Chemistry & Biological Chemistry, School of Physical & Mathematical Sciences, Nanyang Technological University, Singapore 637371, Singapore; 2 Laboratory Breeding Base of Green Pesticide and Agricultural Bioengineering, Key Laboratory of Green Pesticide and Agricultural Bioengineering, Ministry of Education, Guizhou University, Huaxi District, Guiyang 550025, China; 3 Department of Biology and Chemistry, City University of Hong Kong, Tat Chee Avenue, Kowloon Tong, Hong Kong 999078, China; 4 Department of Chemistry, Fudan University, 220 Handan Road, Shanghai 200433, China

## Abstract

The research in the field of asymmetric carbene organic catalysis has primarily focused on the activation of carbon atoms in non-aromatic scaffolds. Here we report a reaction mode of carbene catalysis that allows for aromatic aldehyde activation and remote oxygen atom functionalization. The addition of a carbene catalyst to the aldehyde moiety of 2-hydroxyl aryl aldehyde eventually enables dearomatization and remote OH activation. The catalytic process generates a type of carbene-derived intermediate with an oxygen atom as the reactive centre. Inexpensive achiral urea co-catalyst works cooperatively with the carbene catalyst, leading to consistent enhancement of the reaction enantioselectivity. Given the wide presence of aromatic moieties and heteroatoms in natural products and synthetic functional molecules, we expect our reaction mode to significantly expand the power of carbene catalysis in asymmetric chemical synthesis.

## Abstract (web summary)

Selective functionalization of aromatic compounds is important in synthetic chemistry. Here the authors report an *N*-heterocyclic carbene-catalysed approach for enantioselectively converting *ortho*-hydroxyaldehydes to cyclic products, via activation of both phenol and aldehyde groups.

Aromatic moieties are among the most common functional groups in natural products, bioactive molecules and polymeric materials. Thus, selective functionalization of aromatic molecules and their derivatives has received continuous attention in organic synthesis. Currently, the field of aromatic molecule functionalization, such as aromatic *sp*^2^-CH activation and benzylic *sp*^3^-CH activation, is dominated by transition metal catalysis^1^,^2^,^3^. Organic catalyst-enabled activation of aromatic or benzylic carbon as reactive centre has been much less developed. Representative examples in this direction include photoredox/organocatalytic radical reactions from MacMillan and colleagues^4^ and Melchiorre and colleagues^5^, and amine-mediated reactions via an enamine or iminium intermediate from Melchiorre and colleagues^6^, Jørgensen and colleagues^7^, Chen and colleagues^8^ and Xu *et al*.^9^. In the area of *N*-heterocyclic carbene (abbreviated as NHC or carbene in this manuscript) organocatalysis^10^,^11^, aldehydes^12^,^13^,^14^,^15^,^16^, esters^17^,^18^,^19^ and ketenes^20^,^21^ have been widely employed as substrates. However, nearly all these reactions were focused on using carbon atoms in non-aromatic (and acyclic) molecules as the reactive sites (Fig. 1a). For example, the α and β-carbons of acyclic α,β-unsaturated aldehydes^22^ have been widely explored with a large set of asymmetric reactions. In contrast, aromatic aldehydes are only used in benzoin-type reactions. It remains challenging for the stereo-electronic power of the carbene catalyst to go across the conjugated bonds of the aromatic frameworks to induce activation and selective reactions (Fig. 1b). In 2013, we disclosed that the benzylic *sp*^3^-CH of indole-type heteroaryl aldehyde^23^ could be activated via an analogous vinyl enolate intermediate (NHC-bound *o*-QDMs, Fig. 1b). However, the activation of simple aromatic aldehydes (such as benzaldehyde) without heteroatom incorporated in the aromatic rings remains difficult.

Here we demonstrate that the simple aryl aldehyde could be activated by a carbene catalyst (Fig. 1c). Addition of the carbene catalyst to the aldehyde moiety of 2-hydroxyl benzaldehyde (salicylaldehyde, **1a**) eventually leads to acyl azolium intermediate **I** under an oxidative condition. The electron-withdrawing effect from the acyl azolium moiety, in combination with the electron-donating ability of the OH group, enables a dearomatization^24^ process that forms azolium-bound *ortho*-quinone methide intermediate **II** (*o*-QM). *Ortho*-quinone methide is a reactive intermediate broadly used in the synthesis of sophisticated natural products and other functional molecules^25^,^26^. The oxygen atom in intermediate **II** behaves as an active site to react with ketone substrate **2a** to afford chiral ketal-like **3a** as the product. The overall process is a carbene-catalysed functionalization of the phenol OH group via a type of NHC-bound *o*-QMs intermediate (**II**). Notably, in nearly all of the reported reactions, only carbon atoms of the NHC-bound intermediates (Fig. 1a) were explored as the reactive centres^27^,^28^. The exceptions are NHC-mediated polymerization of lactone^29^ and Breslow intermediate-induced ring expansion^30^,^31^,^32^. Our present work realizes oxygen atom activation, in which the reactivity of the oxygen atom is modulated by the covalent-bound NHC catalyst in intermediate **II**. The azolium-bound *ortho*-quinone methides intermediate (**II**) constitutes a new mode for NHC catalysis. Additionally, we realize a urea/NHC cooperative catalytic process^33^ (**III**, Fig. 1c), in which an achiral urea co-catalyst significantly and consistently improves the enantioselectivity of the reaction.

## Results

### Reaction optimizations

**Figure 1:**
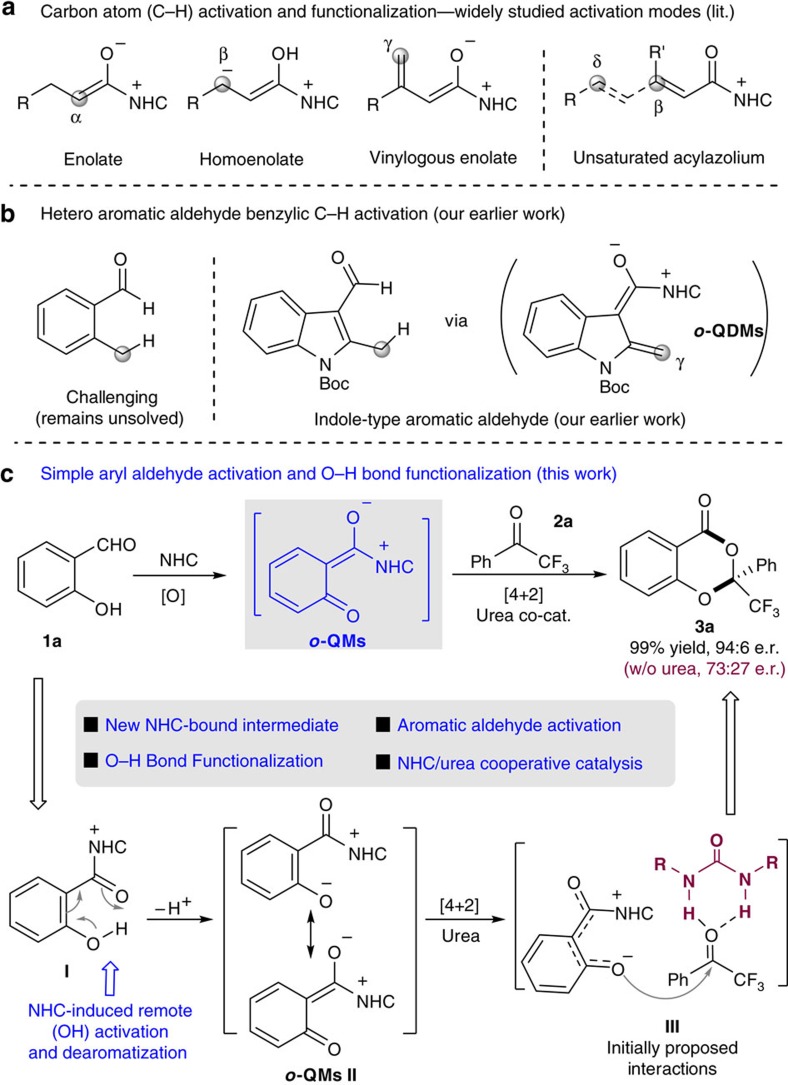
Activation modes in carbene organic catalysis. (**a**) Previous studies mainly dealt with carbon atom activation for acyclic non-aromatic carbonyl compounds. (**b**) Our earlier studies realized indole-type heteroaryl aldehyde benzylic carbon activation. (**c**) Addition of carbene catalyst eventually leads to simple phenyl aldehyde activation which induces phenol oxygen atom functionalization via a new azolium-bound intermediate (**II**).

Key results of initial optimization of reaction conditions are summarized in Table 1. Salicylaldehyde (**1a**) and trifluoroacetophenone (**2a**) were chosen as the model substrates to form 1,3-dioxin-4-one (**3a**). Studies on the solvent effects (see Supplementary Tables 2 and 3) found that a mixture of CH_2_Cl_2_/hexane (1:1, v/v) is the optimal choice. Quinone (**DQ**), first explored by Studer and colleagues in NHC catalysis^34^, is an effective oxidant. Several bases (see Supplementary Table 1) could produce the desired product in such transformations. In the absence of NHC catalyst, product **3a** was not formed. The two starting materials (**1a** and **2a**) remained unreacted and could be recovered (entry 1). The search for NHC catalysts found that aminoindanol-derived triazolium precatalyst **C1** with an *N*-mesityl substituent (first explored by Bode^35^) could mediate the formation of **3a** in 58% yield and with 63:37 e.r. (entry 2). The reaction yield could be dramatically increased to 97% when precatalyst **C2** with an *N*-trichlorophenyl substituent (first explored by Rovis^36^) was used (entry 3). Decreasing the reaction temperature from room temperature to −10 °C led to a small but consistent improvement in the e.r. value without affecting the reaction yield (entry 4). Further decreasing the reaction temperature, however, slowed down the reaction without enhancing the enantioselectivity. We then turned to identifying conditions that could improve the reaction enantioselectivity. In our studies on the solvent effects (see Supplementary Table 2), we found that solvents could affect the reaction enantioselectivity. For example, when THF was used as the solvent, the e.r. value of **3a** decreased from 73:27 to 52:48 (entry 4 versus 5). These results suggest that non-covalent interaction is likely to play an important role in controlling the enantioselectivity of our reactions. We thus examined non-covalent organic catalysts and found that using achiral thiourea^37^,^38^,^39^,^40^,^41^ **A1** as a co-catalyst could improve the e.r. values of **3a** from 73:27 to 85:15 (entry 6 versus 4). Increasing the amount of **A1** led to a lower yield of **3a** with little benefit to the enantioselectivity (entry 7). Further studies found that both thiourea and urea^42^ co-catalysts can improve the enantioselectivity (entries 8 and 9, and see Supplementary Table 4). We finally found that using asymmetric urea **A3** could lead to **3a** in 99% yield and with 94:6 e.r. (entry 9). The loading of the NHC catalyst (**C2**) could be decreased to 5 mol%, without affecting the reaction yield and e.r. (entry 11). The urea co-catalyst likely forms hydrogen-bonding interactions with the ketone substrate (for example, **2a**) and/or intermediate **II** (Fig. 1c) to improve the reaction enantioselectivity. The hydrogen-bonding interactions are sensitive to solvents. For example, switching the solvent from CH_2_Cl_2_/hexane to THF led to a dramatic drop in enantioselectivity (entries 10 versus 11).

### Substrate scope with 2-hydroxyl aryl aldehydes

With an acceptable reaction condition in hand (Table 1, entry 11), the generality of the reaction was then explored. First, we studied the scope of the 2-hydroxyl aryl aldehydes (Table 2). With trifluoroacetophenone **2a** as a model electrophile, different substituents and substitution patterns on the phenyl ring were examined. Electron-releasing substituents such as alkyl (**3b**, **3d** and **3k**) and alkoxyl (**3c**, **3g** and **3j**) units on the phenyl ring of the aldehyde substrates were well tolerated. Electron-withdrawing groups such as halogen atoms (**3e**, **3f** and **3h**) and carboxylic ester units (**3i**) could also be placed on the phenyl ring of the aldehyde substrates. It is worth noting that the steric effect of substituents in different positions on the phenyl ring does not affect the results, as seen for the substrates with 3- and 6- substituents on the 2-hydroxyl aryl aldehydes, but these substitution patterns both led to very good yields and e.r. (**3j**, **3k** and **3l**). Product **3m** bearing a sesamol moiety (3,4-methylenedioxyphenol) could also be made by our method. The benzene ring of **1a** could be extended to other aromatic frameworks, such as naphthyl units (**3n**–**3p**). Additionally, steric influence has little effect on the reaction outcomes for all the three naphthaldehyde substrates (**3n**–**3p**). Notably, in all cases, the reactions were highly efficient, and the products were obtained in excellent yields. The two starting materials were used with nearly a 1:1 ratio.

### Substrate scope with trifluoromethyl ketones

We next examined the reactions of trifluoromethyl ketone substrates **2** in our standard condition (Table 3). Here we chose 5-methylsalicyladehyde (**1b**) as a model aldehyde substrate because it is easier to handle than **1a**, as **1b** is a solid at room temperature and stable toward air-oxidation. Different substituents and substitution patterns on the phenyl ring of the ketone substrates were all tolerated (**3b** and **3q–3x**). Electron-donating groups such as methyl (**3q**), methoxy (**3r**) and *N,N*-dimethyl amino (**3s**) groups on the phenyl *para*-position were all tolerated, giving the corresponding products with excellent values of yield and e.r. Electron-withdrawing substituents on the phenyl *meta*- or *para*- position (**3t**, **3x**) had little effect on the yields and selectivities. The phenyl substituent of ketone **2a** could be replaced by a heteroaryl substituent (**3y**). Alkyl substituted trifluoromethyl ketones are typically challenging substrates when used as electrophiles because of the ketone/enol isomerization. These substrates could be used in our reactions as well (**3z**, **3za**), albeit with a small to moderate drop in the yield and e.r. values.

### DFT studies on NHC-bound intermediate II

**Figure 2:**
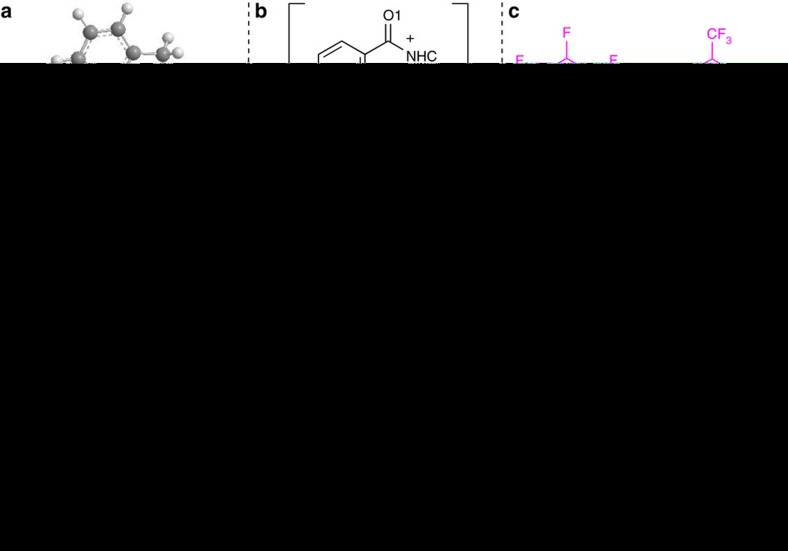
DFT studies of intermediate II and the roles of urea co-catalyst. (**a**) Structure of **II** and key interatomic distances (in Å). (**b**) Resonance structures of **II**. (**c**) Schematic drawing of A3-TS2_1-A_. (**d**) NCI plot for A3-TS2_1-A_. Green surfaces represent attractive interactions. Some atoms are omitted for clarity. (**e**) NCI plots for A3-TS2_1-A_ and A3-TS2_1-A-minor_. Relative free energies (kcal mol^−1^) are with respect to A3-TS2_1-A_.

DFT calculations were used to assess the nature of *o*-QM in intermediate **II**. The computational details are provided in the Supplementary Figs 56–65. Six conformers of **II** were obtained from DFT calculations. The optimized structure of the most stable conformer of intermediate **II** (**II-1**), which can later react with ketone **2a**, is shown in Fig. 2a. Other less stable structures are shown in the Supplementary Information. We also calculated Mulliken atomic charges for **II-1** and found that the O1 and O2 atoms have partial negative charges of −0.33 and −0.47, respectively (Fig. 2b); the magnitudes of these values are significantly smaller than 1. These fractional charges imply that **II-1** cannot be described as either of the two extreme resonance structures depicted in Fig. 2b; instead, the real state of *o*-QM should lie somewhere in between.

### DFT studies on the mechanisms of [4+2] annulation

To gain mechanistic insights, DFT calculations were performed for the reaction between intermediate **II** and ketone substrate **2a** in the presence or absence of a urea co-catalyst, taking into account the solvent effect of DCM (see the Supplementary Tables 6–11 for details). The calculations without urea suggest that NHC-bound intermediate **II** can react with **2a** in a [4+2] mechanism (Fig. 1c) to afford a ketal-like product, without having very high barriers, thus supporting the hypothesis regarding the involvement of *o*-QM in the reaction. The four conformers (**II-1**, **II-2**, **II-3** and **II-4**) of intermediate **II** in Supplementary Fig. 59 can react with **2a**, giving rise to several possible reaction pathways (16 pathways in total). DFT calculations without urea showed that this reaction consists of two steps: (1) a ring-annulation step and (2) an NHC dissociation step, and that step 2 should be the rate-determining step (see the Supplementary Information). On the basis of this result, we performed further calculations with co-catalyst **A3**.

The lowest-energy transition state for the rate-determining step on Path 1-A (A3-TS2_1-A_) with urea **A3** is connected to the major product. In this transition state, unlike our initial guess (Fig. 1c), urea **A3** forms hydrogen bonds preferentially with the carbonyl oxygen atom of the *o*-QM moiety (Fig. 2c), rather than with the trifluoromethyl ketone substrate (Fig. 1c). A3-TS2_1-A_ has attractive *π-π* stacking between the pentafluorophenyl group of **A3** and the indane moiety of the catalyst (Fig. 2d). Such *π-π* stacking is also observed in A3-TS2_1-A-minor_, but not in the other A3-TS2's. The somewhat greater stability of A3-TS2_1-A_ compared with A3-TS2_1-A-minor_ is attributed to the attractive dispersion interactions between the urea moiety and the phenyl group from ketone (Fig. 2e), which are prominent only in A3-TS2_1-A_. Our calculations therefore highlight the importance of dispersion interactions in determining the enantioselectivity.

### Application of the [4+2] annulation products

**Figure 3:**
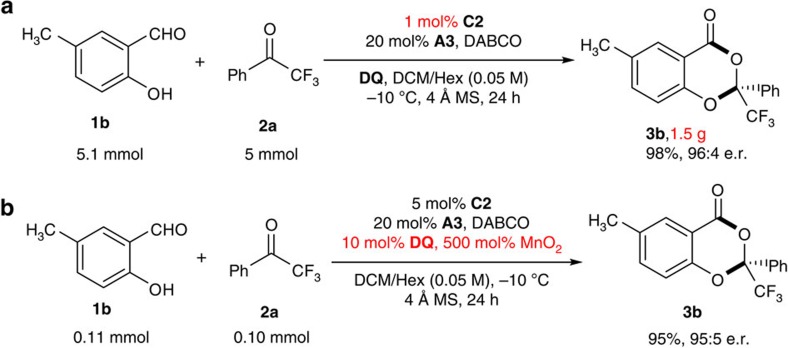
Scalable and practical synthesis. (**a**) Gram scale synthesis with 1 mol% NHC loading. (**b**) Reaction using MnO_2_ as terminal oxidant.

Our reaction is amenable for large-scale synthesis (Fig. 3a). Notably, in the gram scale synthesis, the use of 1 mol% NHC precatalyst was sufficient to produce **3b** (1.5 g) in 98% yield and with 96:4 e.r. Additionally, the organic oxidant (**DQ**) could be used in catalytic amount by using inexpensive MnO_2_ as a terminal oxidant^43^ (Fig. 3b).

The products of our catalytic reactions contain a benzo[1,3]dioxin scaffold. This chiral benzo[1,3]dioxin unit itself and its closely related structures are widely found in natural products and bioactive synthetic molecules. Examples of such molecules include natural product Epicocconigrone A with anticancer activities^44^ and Efavirenz, commercially used as an HIV reverse transcriptase inhibitor^45^. Our laboratories are interested in the antiviral and antibacterial activities of these compounds for agricultural use^46^,^47^,^48^. Therefore, we then evaluated the *in vitro* bioactivities of our products against several types of bacteria and fungi that can cause plant infections (Table 4). The commercially available and commonly applied bactericide Kresoxim-methyl was used as the positive control, and dimethyl sulfoxide was used as the negative control. Preliminary studies found that several of our compounds showed significant activities against Eggplant *Verticillium*, *Phytophthora infestans* and *Fusarium oxysporum*. For example, compound **3l** showed 25–38% inhibition rates against the above three fungi at a concentration of 50 μg ml^−1^.

## Discussion

In short, we have developed a reaction mode of carbene-catalysed activation of aryl aldehydes. Addition of the carbene catalyst to the aldehyde moiety eventually leads to remote phenol OH activation and dearomatization. The catalytic reaction involves an NHC-bound intermediate (*o*-QMs) with the oxygen atom acting as the reactive centre. A hydrogen-bond donating co-catalyst (urea or thiourea) works cooperatively with the NHC catalyst, resulting in significant and consistent enhancement of the reaction enantioselectivity. Our method is amenable for large-scale enantioselective synthesis with relatively low loading of the NHC catalyst. Preliminary studies on the bioactivities of our compounds identified a few leads with antifungal activities. We expect our study to encourage further exploration on new reaction modes and alternative intermediates with NHC organic catalysis. Our findings of NHC/urea cooperative catalysis will provide some possibilities in controlling challenging stereoselectivities, for example, in remote group functionalizations.

## Methods

### General strategy of [4+2] annulations

To a 10 ml flame-dry Schlenk reaction tube equipped with a magnetic stir bar, chiral NHC pre-catalyst **C2** (0.005 mmol, 5 mol%, 2.4 mg), urea **A3** (0.02 mmol, 20 mol%, 8.76 mg), DABCO (0.1 mmol, 100 mol%, 11.2 mg), oxidant **DQ** (0.11 mmol, 110 mol%, 45 mg), aldehyde (0.11 mmol) and 4 Å molecular sieves were added. The Schlenk tube was sealed with a septum, evacuated and refilled with nitrogen (3 cycles). Solvent (CH_2_Cl_2_/hexane=1:1, 2.0 ml) and trifluoromethyl ketone **2** (0.1 mmol) were then added via syringe. The reaction mixture was allowed to stir for 24 h at −10 °C. After completion of the reaction, monitored by a TLC plate, the reaction mixture was concentrated under reduced pressure, and the residue was subjected to column chromatography or TLC plate directly using hexane/EtOAc as eluent to afford the desired product **3**. For ^1^H, ^13^C, ^19^F NMR and high-performance liquid chromatography spectra of compounds in this manuscript, see Supplementary Figs 1–54.

### Data availability

The X-ray crystallographic coordinates for structures reported in this article have been deposited at the Cambridge Crystallographic Data Centre (**3h:** CCDC 1486140). These data could be obtained free of charge from The Cambridge Crystallographic Data Centre via www.ccdc.cam.ac.uk/data_request/cif. For XYZ coordinates of optimized structures in DFT studies on the mechanism, see Supplementary Data 1.

## Additional information

**How to cite this article:** Chen, X. *et al*. A reaction mode of carbene-catalysed aryl aldehyde activation and induced phenol OH functionalization. *Nat. Commun.* **8,** 15598 doi: 10.1038/ncomms15598 (2017).

**Publisher's note**: Springer Nature remains neutral with regard to jurisdictional claims in published maps and institutional affiliations.

## Supplementary Material

Supplementary Information Supplementary figures, supplementary tables, supplementary methods and supplementary references.

Supplementary data 1 In DFT study, XYZ coordinates of optimized structures are important parameters to understand the calculation models. For XYZ coordinates of optimized structures in DFT studies on the mechanism, see Supplementary Data 1.

## Figures and Tables

**Table 1:**
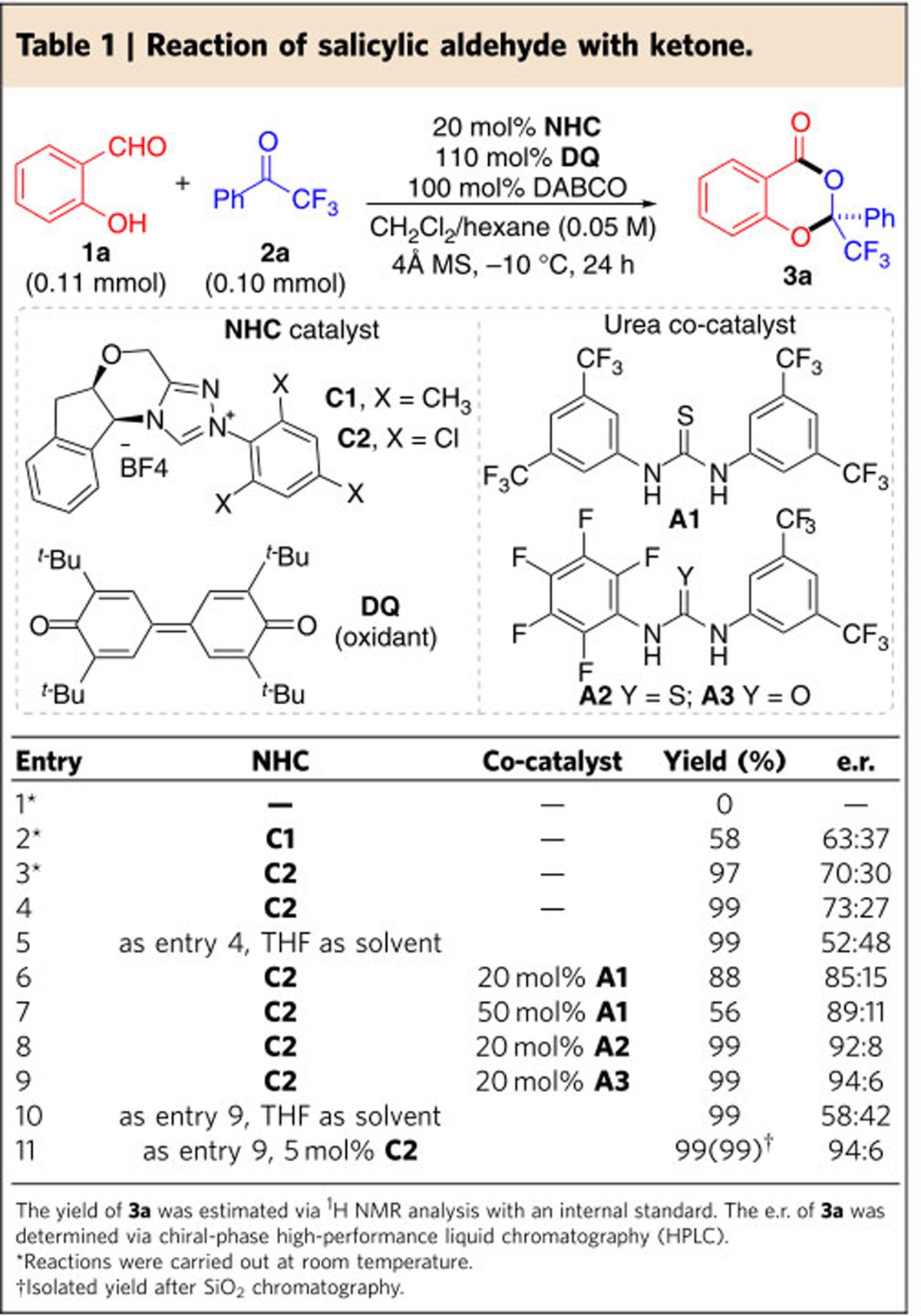
Reaction of salicylic aldehyde with ketone.

**Table 2:**
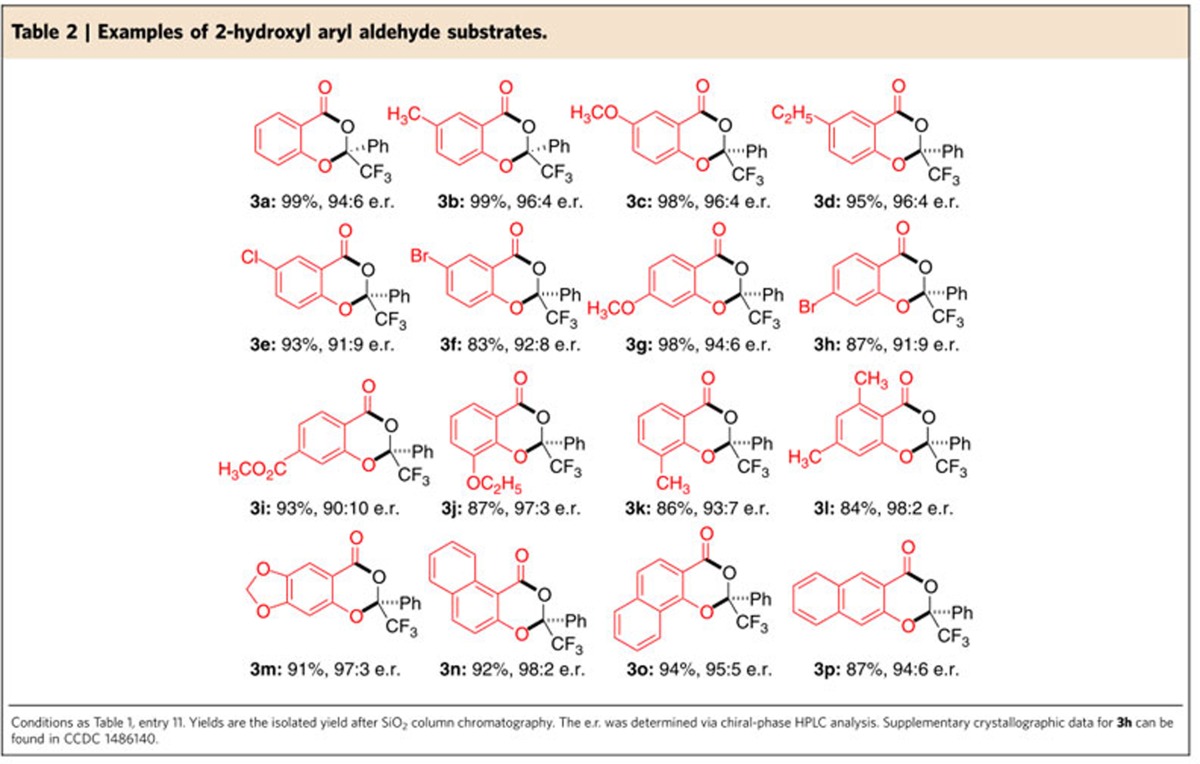
Examples of 2-hydroxyl aryl aldehyde substrates.

**Table 3:**
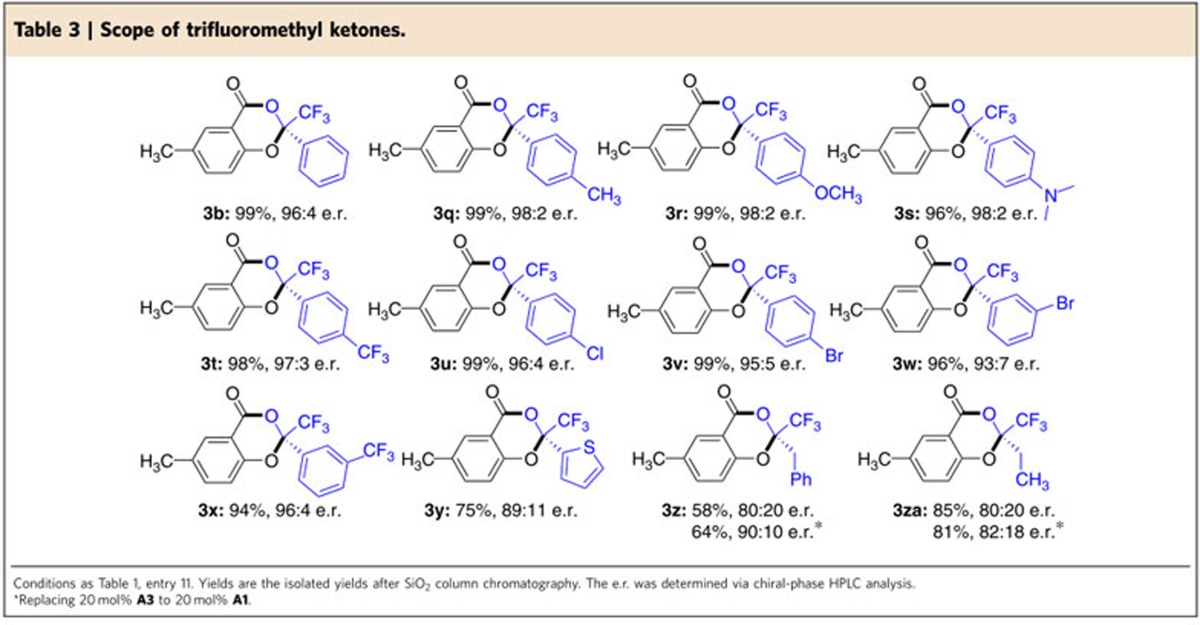
Scope of trifluoromethyl ketones.

**Table 4:** The antifungal activity of our products.

	**Inhibition rate (%)**		
**Sample**	**Eggplant *Verticillium***	***Phytophthora infestans***	***Fusarium oxysporum***
**3c**	26.10±0.64	26.47±2.45	**26.71±2.18**
**3b**	34.98±2.92	16.98±6.26	11.50±2.75
**3i**	3.60±1.61	10.49±0.93	4.71±1.06
**3l**	**32.91±1.36**	**38.58±0.59**	25.65±0.61
Positive control	100	84.00±3.59	69.61±2.54
Negative control	0	0	0

Inhibitory effects of compounds at a concentration of 50 μg ml^−1^. Each data is the average of three replicates. Kresoxim-methyl was used as the positive control, Dimethyl sulfoxide was used as negative control.
