# Supplementary material for: A reaction mode of carbene-catalysed aryl aldehyde activation and induced phenol OH functionalization
Source: Nat Commun. 2017 May 25;8:15598. doi: 10.1038/ncomms15598 (PMC5477515; doi:10.1038/ncomms15598)
Supplement: Supplementary Information — Supplementary figures, supplementary tables, supplementary methods and supplementary references. [file ncomms15598-s1.pdf]

Supplementary Figure 1. ^{1}H and ^{13}C NMR spectra for compound 3a

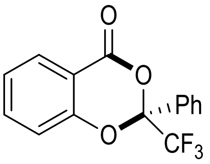

3a: 99%, 94:6 er

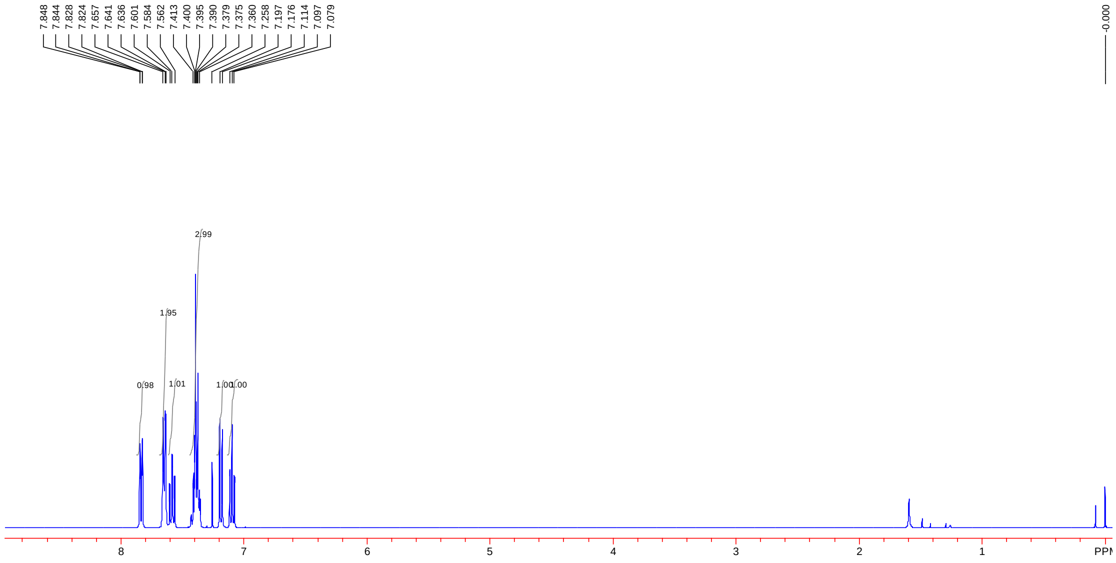

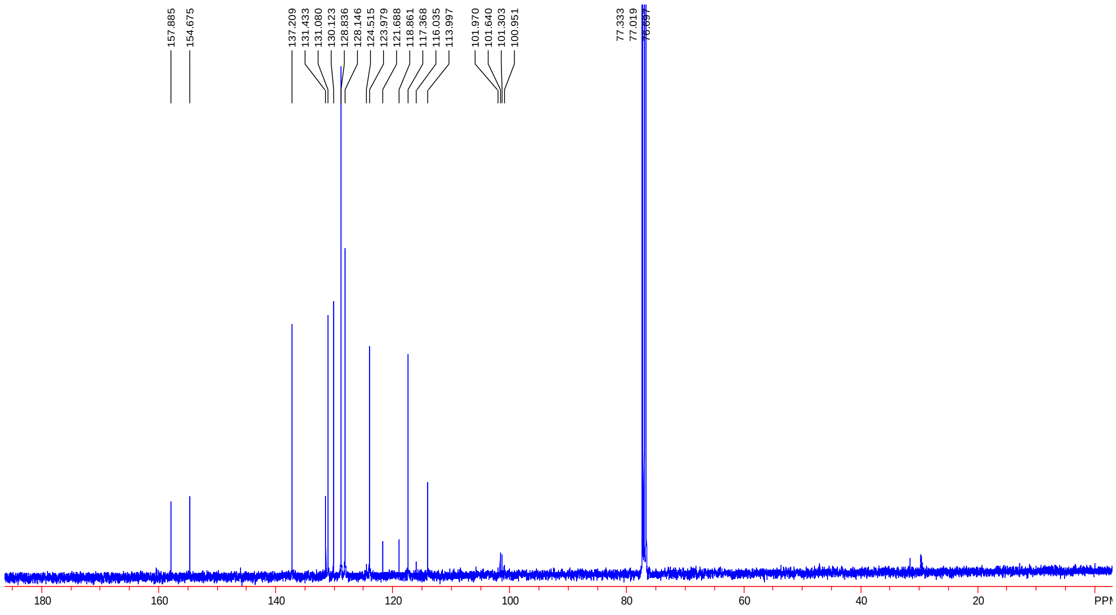

Supplementary Figure 2. ^{19}F and HPLC spectra for compound 3a

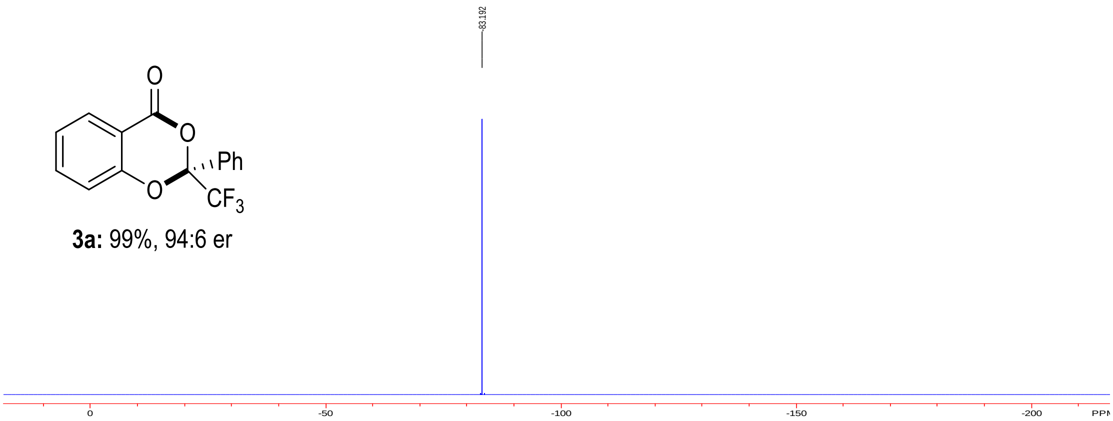

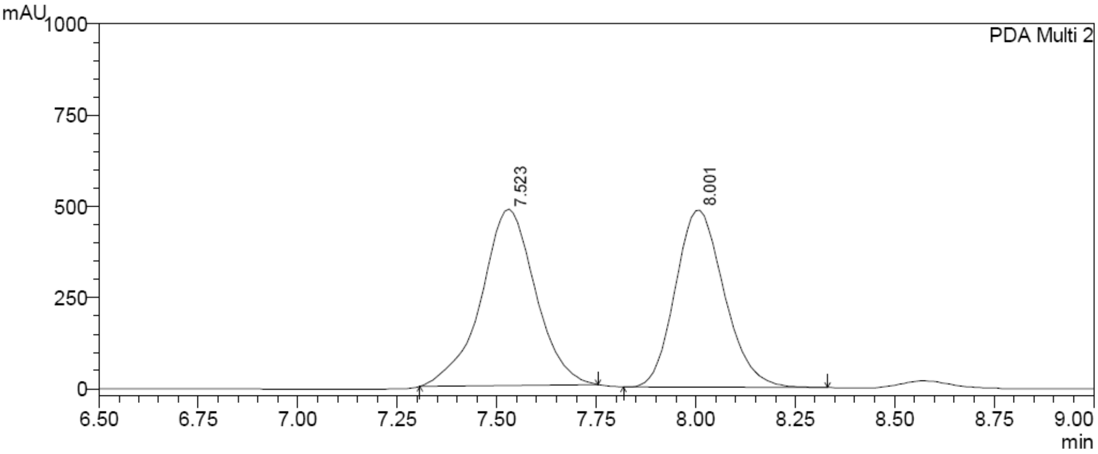

Peak#	Ret. Time	Area	Height	Area %	Height %
1	7.523	4598339	483351	52.449	49.887
2	8.001	4168926	485536	47.551	50.113
Total		8767265	968887	100.000	100.000

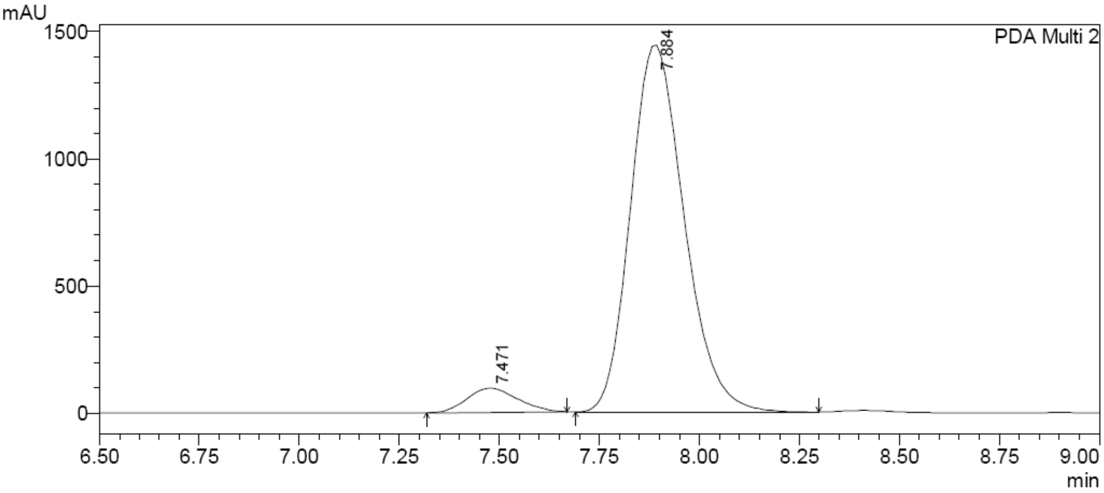

Peak#	Ret. Time	Area	Height	Area %	Height %
1	7.471	843636	95952	5.888	6.241
2	7.884	13485102	1441386	94.112	93.759
Total		14328738	1537338	100.000	100.000

Supplementary Figure 3. ^{1}H and ^{13}C NMR spectra for compound 3b

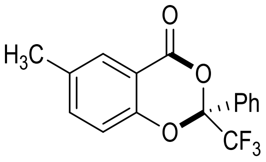

3b: 99%, 96:4 er

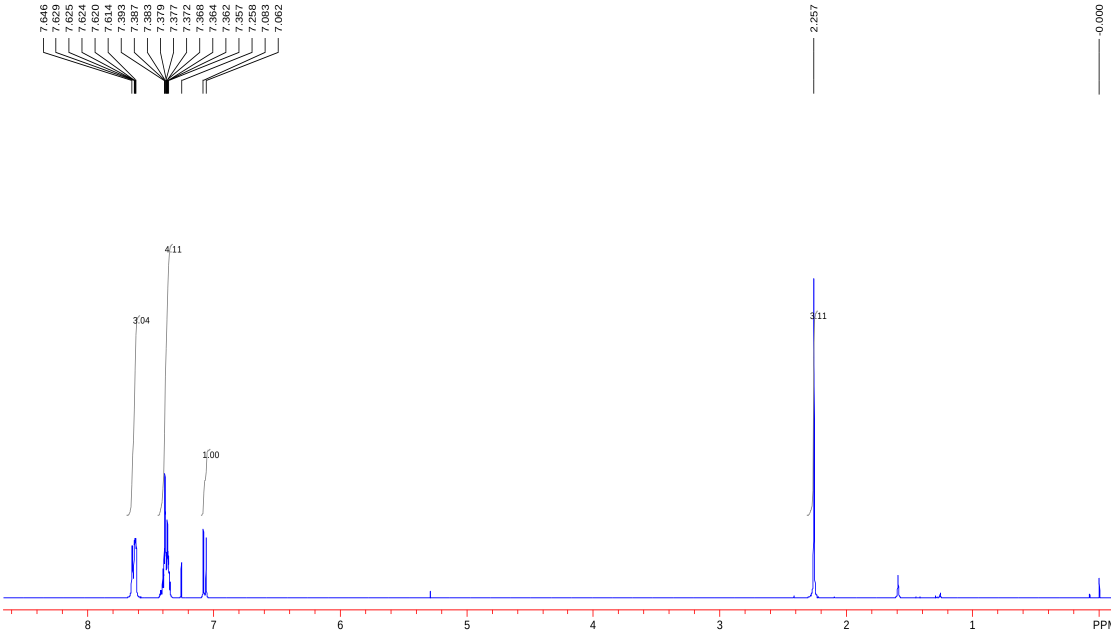

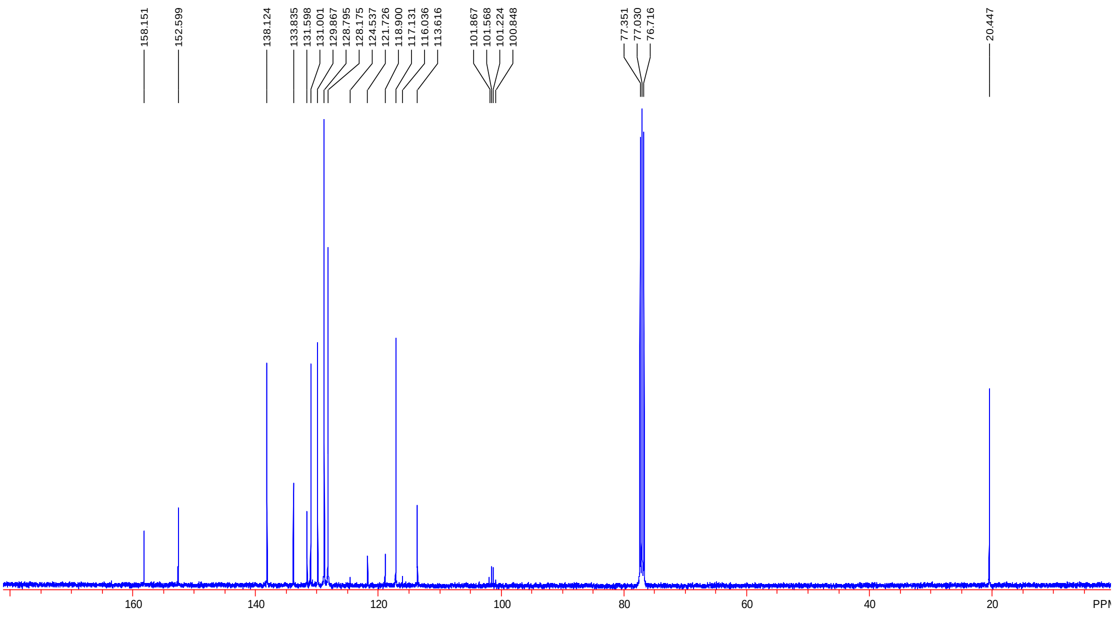

Supplementary Figure 4. ^{19}F and HPLC spectra for compound 3b

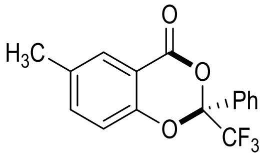

3b: 99%, 96:4 er

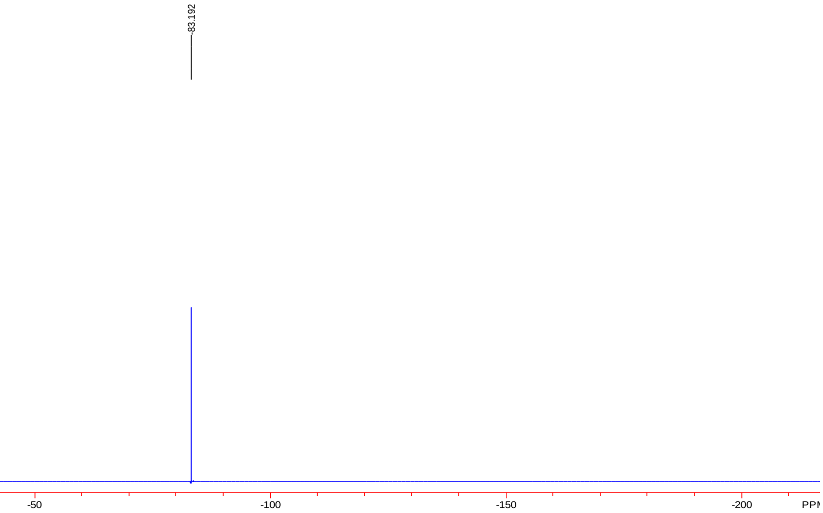

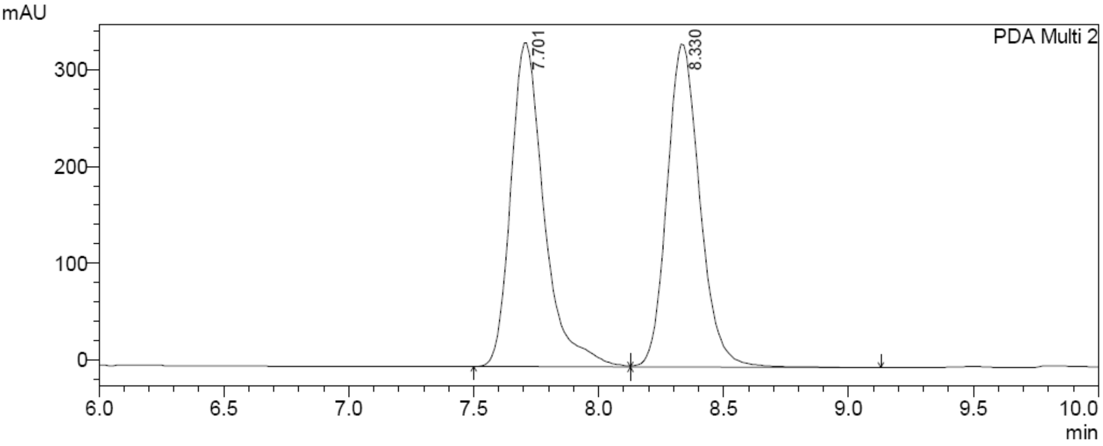

Peak#	Ret. Time	Area	Height	Area %	Height %
1	7.701	380430	41566	50.446	50.134
2	8.330	373701	41344	49.554	49.866
Total		754131	82910	100.000	100.000

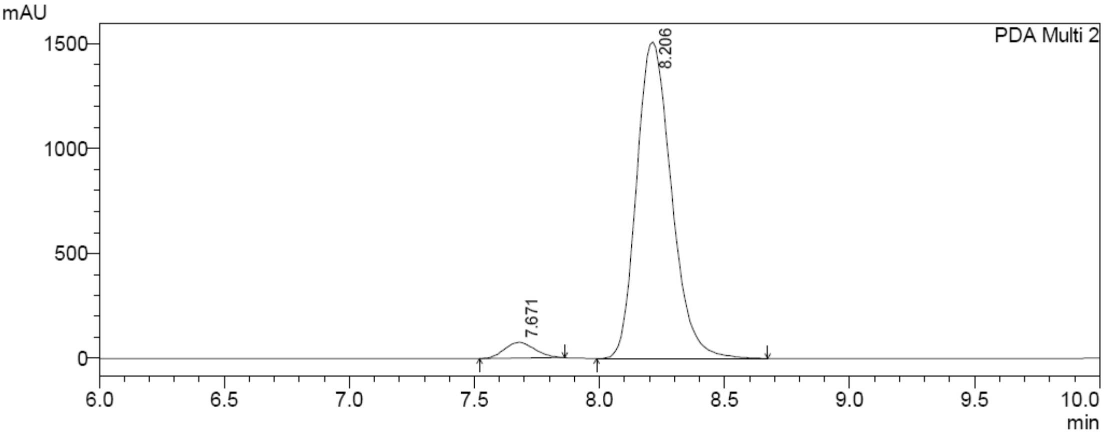

Peak#	Ret. Time	Area	Height	Area %	Height %
1	7.672	78044	9181	3.866	4.203
2	8.206	1940829	209233	96.134	95.797
Total		2018874	218414	100.000	100.000

Supplementary Figure 5. ^{1}H and ^{13}C NMR spectra for compound 3c

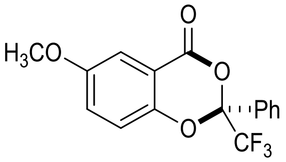

3c: 98%, 96:4 er

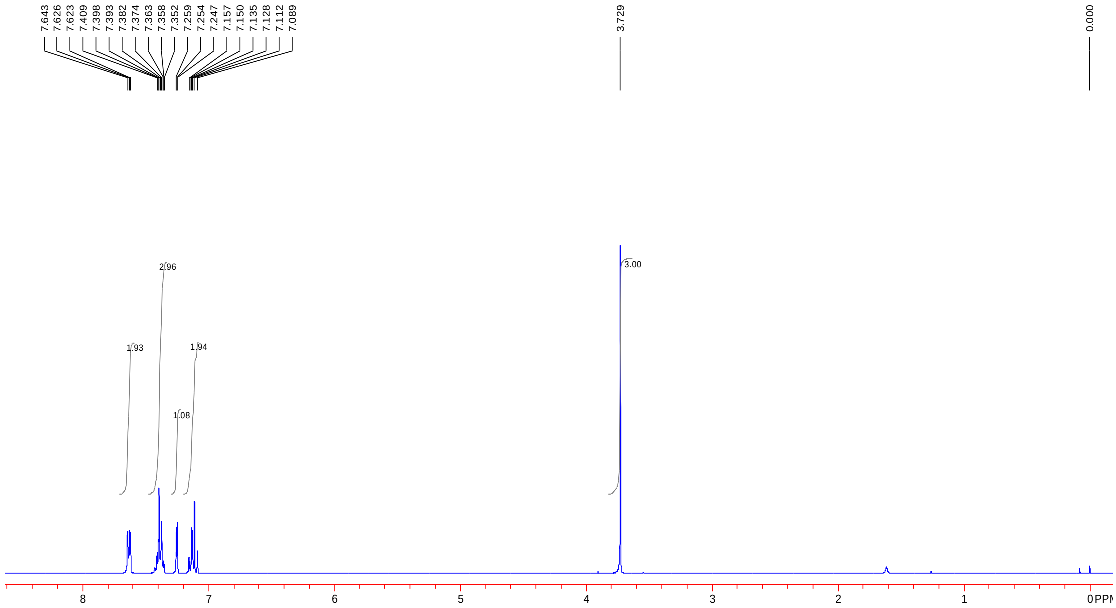

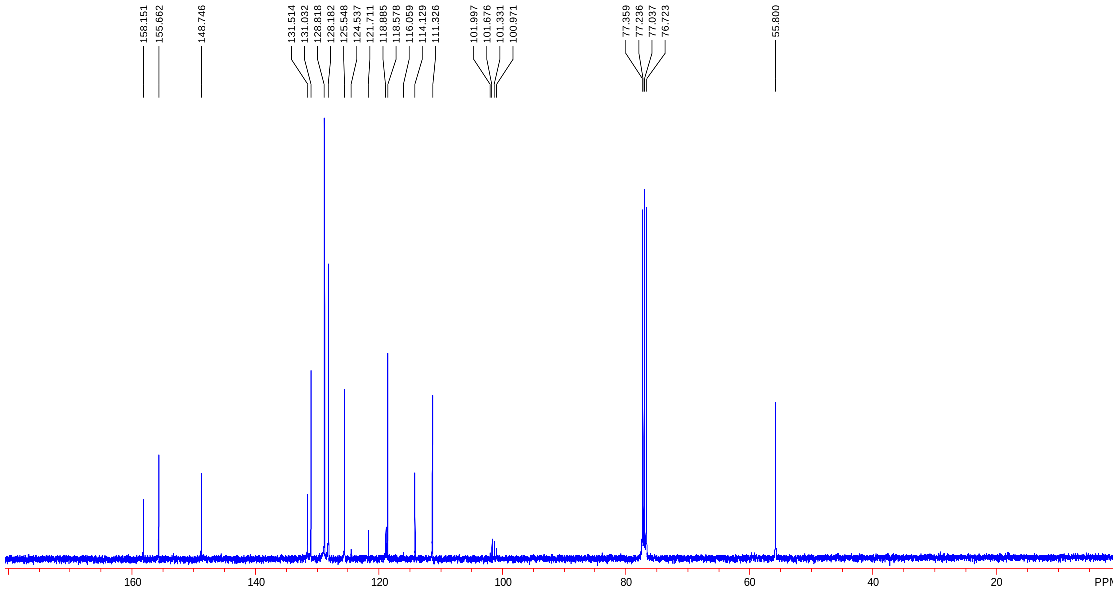

Supplementary Figure 6. ^{19}F and HPLC spectra for compound 3c

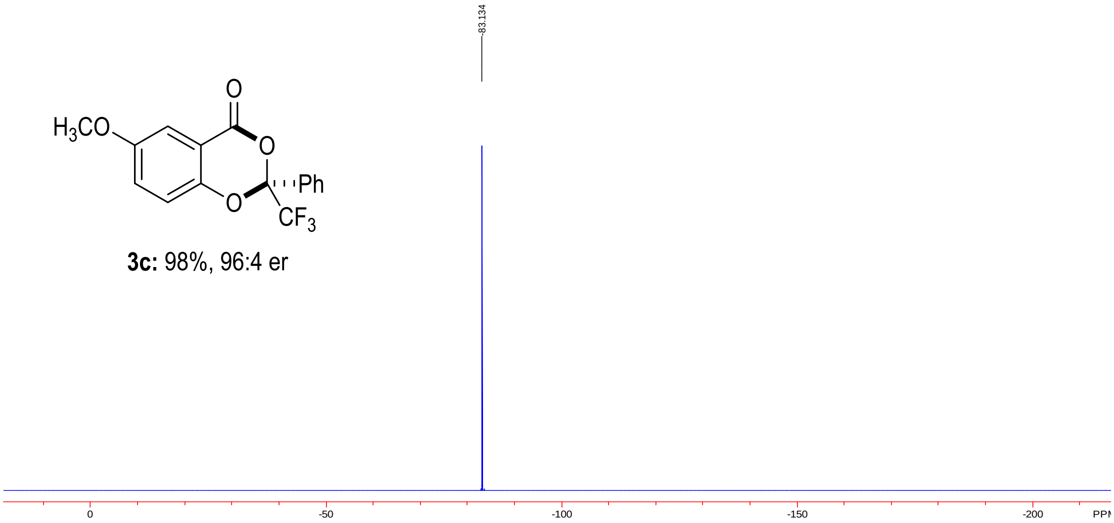

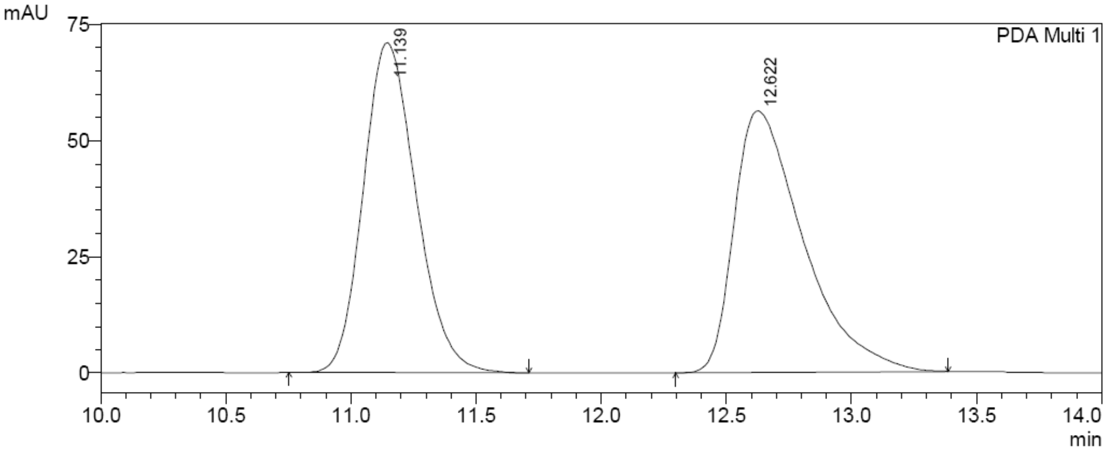

Peak#	Ret. Time	Area	Height	Area %	Height %
1	11.139	1047368	71059	49.111	55.779
2	12.622	1085280	56334	50.889	44.221
Total		2132648	127394	100.000	100.000

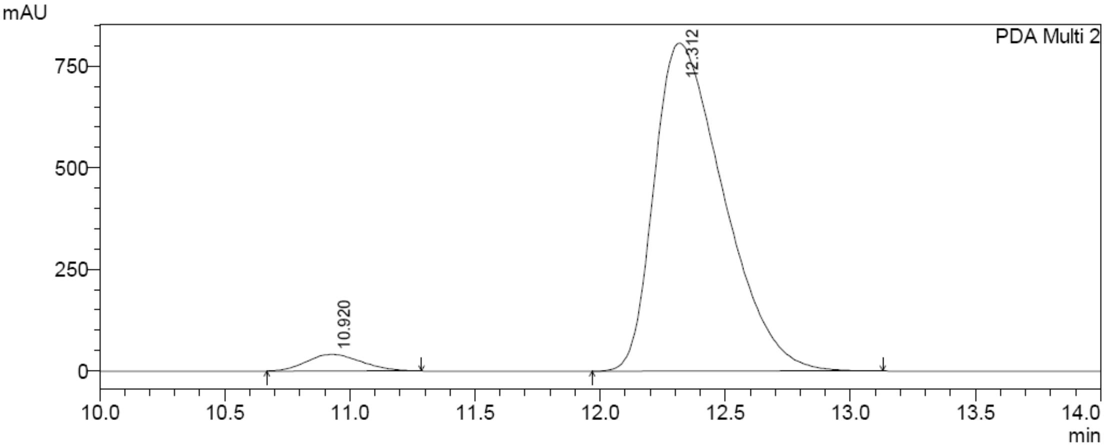

Peak#	Ret. Time	Area	Height	Area %	Height %
1	10.920	50719	3323	3.843	4.795
2	12.312	1269208	65978	96.157	95.205
Total		1319927	69301	100.000	100.000

Supplementary Figure 7. ^{1}H and ^{13}C NMR spectra for compound 3d

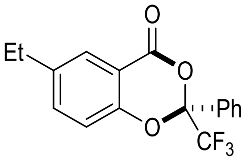

3d: 95%, 96:4 er

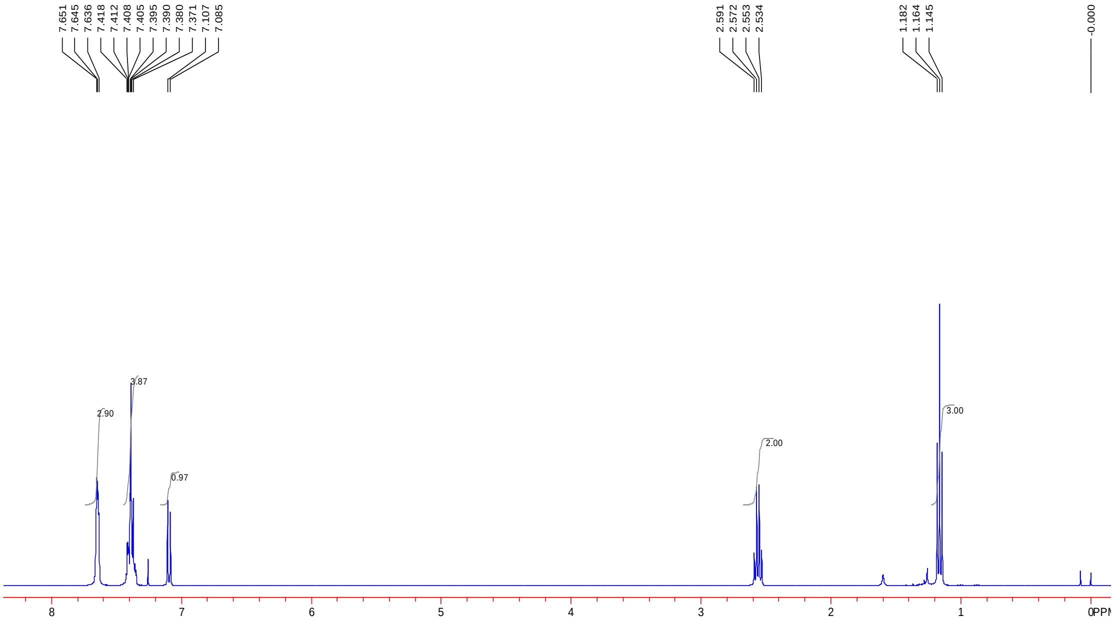

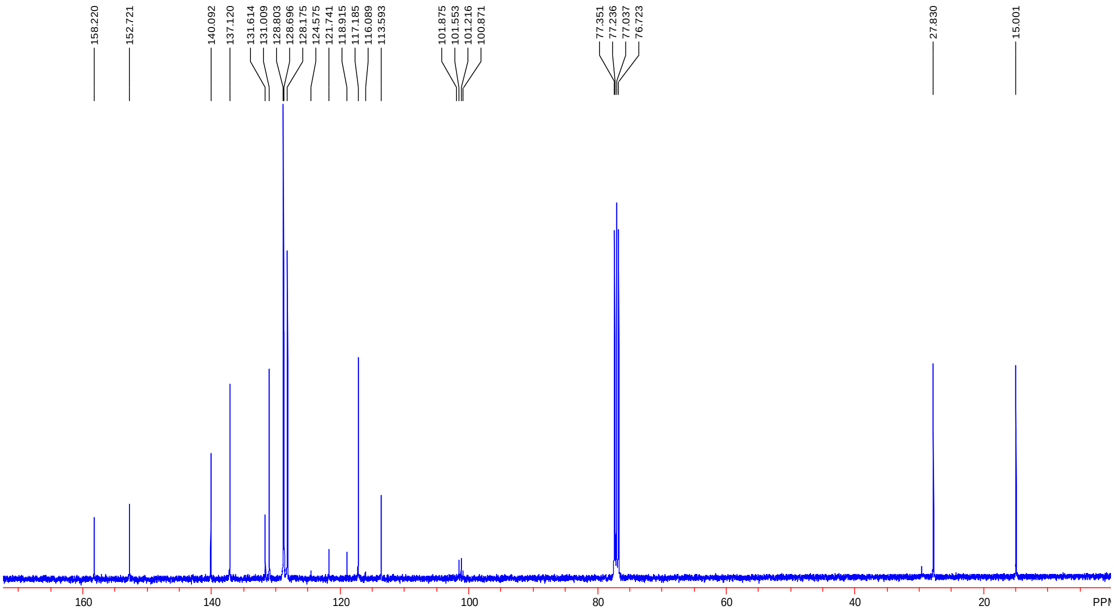

Supplementary Figure 8. ^{19}F and HPLC spectra for compound 3d

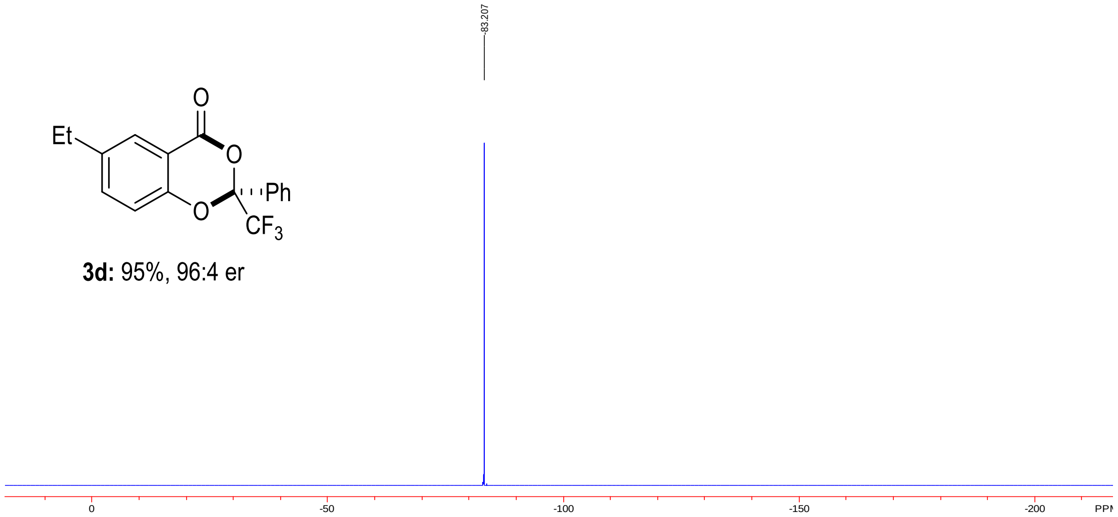

3d: 95%, 96:4 er

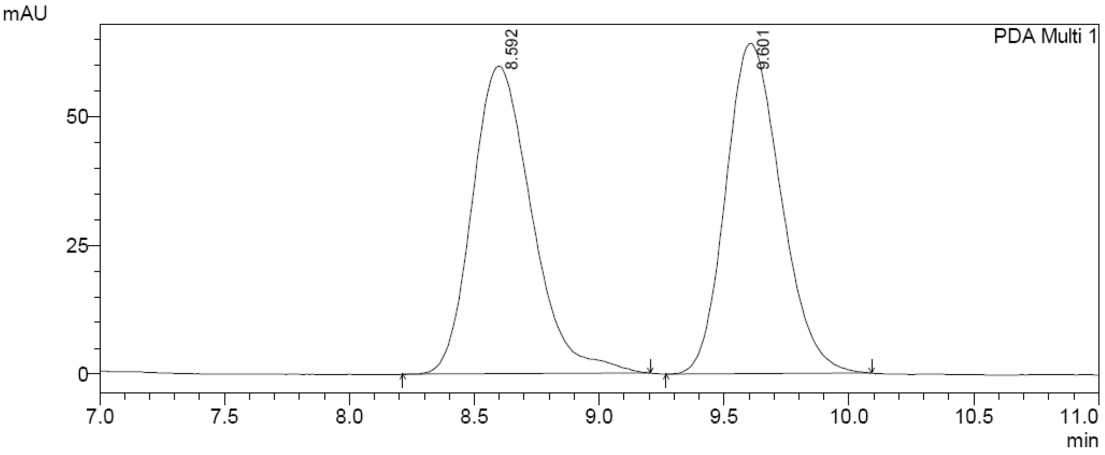

Peak#	Ret. Time	Area	Height	Area %	Height %
1	8.592	989766	59781	50.068	48.251
2	9.601	987081	64114	49.932	51.749
Total		1976847	123895	100.000	100.000

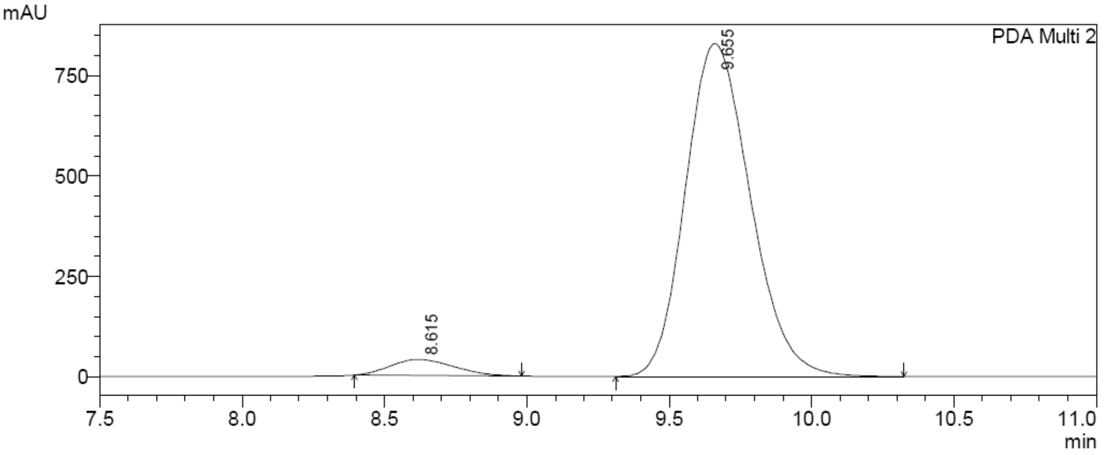

Peak#	Ret. Time	Area	Height	Area %	Height %
1	8.616	64982	4138	4.357	4.305
2	9.655	1426424	91975	95.643	95.695
Total		1491406	96113	100.000	100.000

Supplementary Figure 9. ^{1}H and ^{13}C NMR spectra for compound 3e

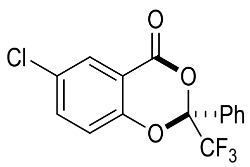

3e: 93%, 91:9 er

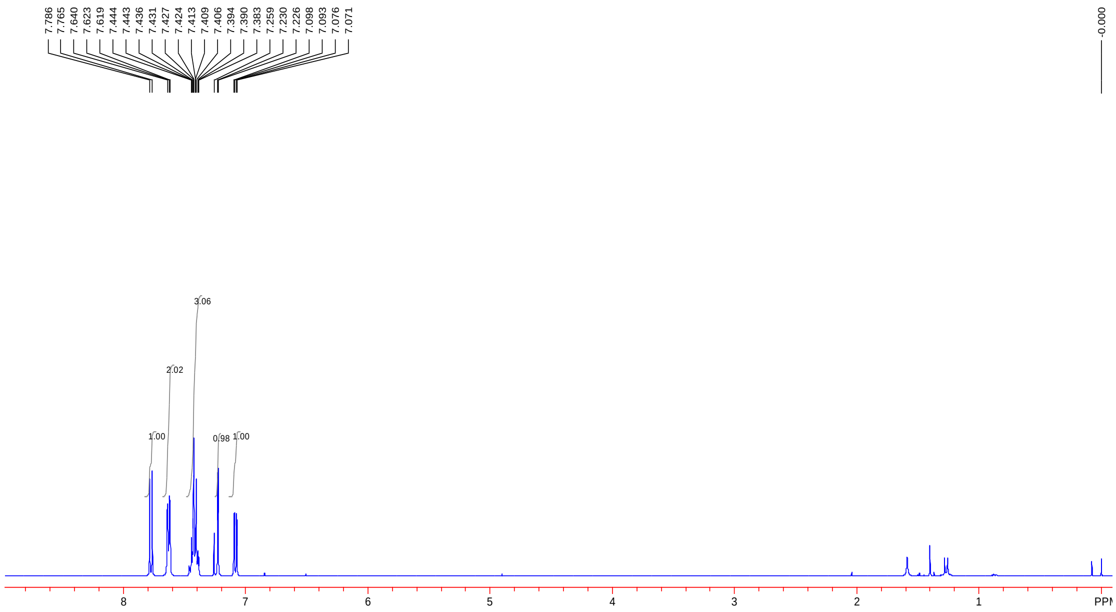

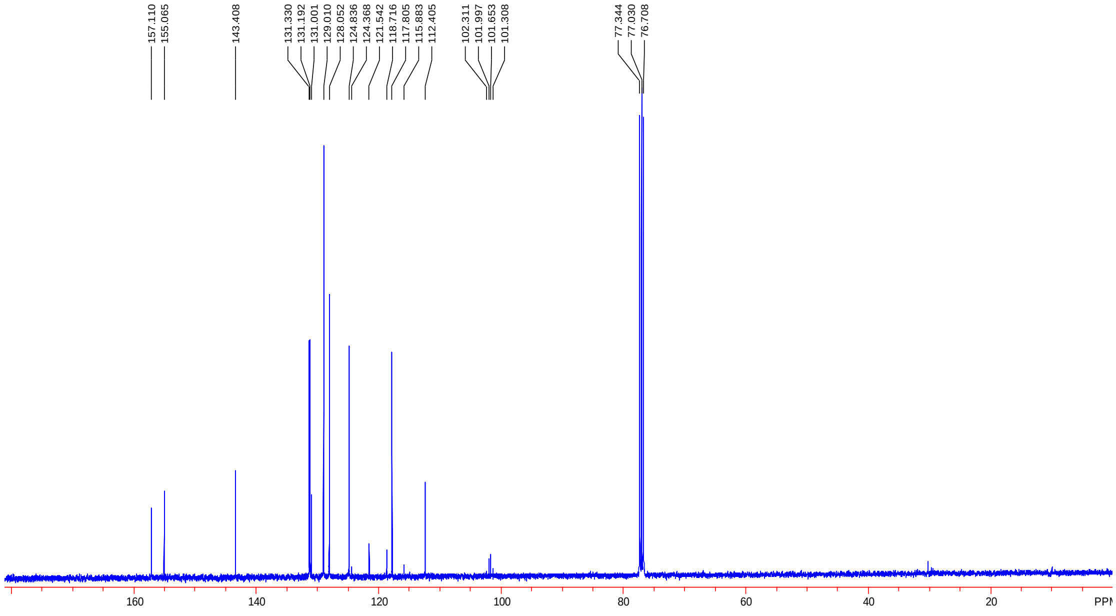

Supplementary Figure 10. ^{19}F and HPLC spectra for compound 3e

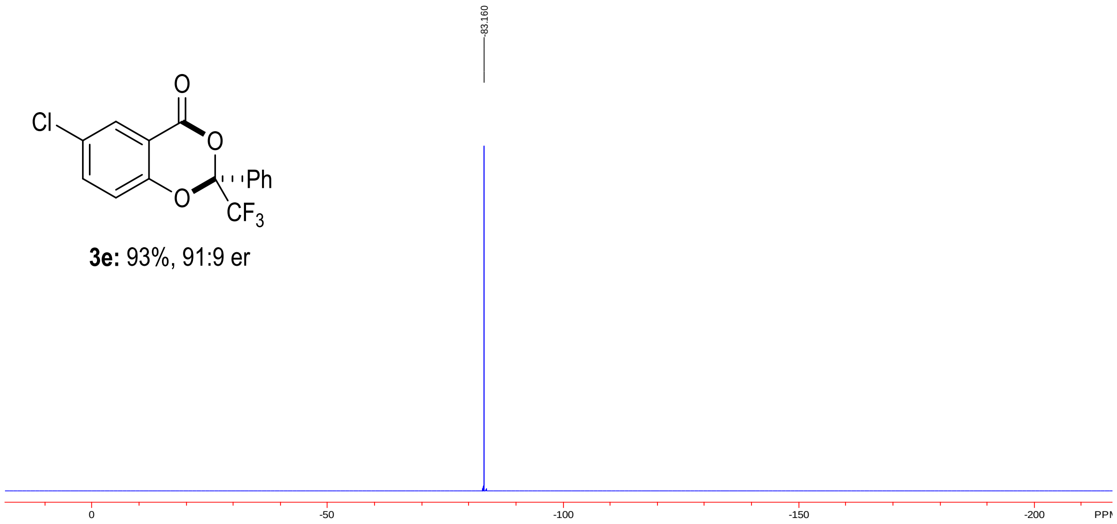

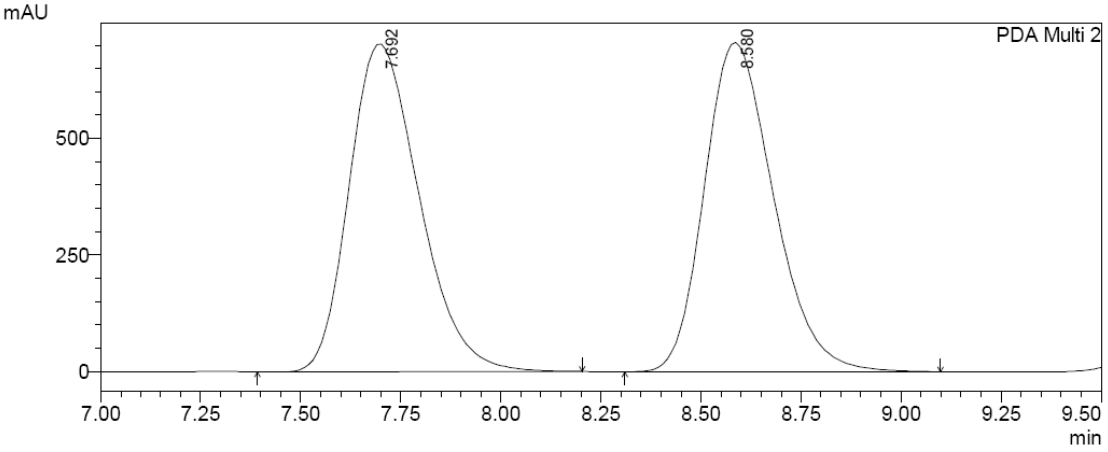

Peak#	Ret. Time	Area	Height	Area %	Height %
1	7.692	8562325	703028	50.131	49.846
2	8.580	8517535	707375	49.869	50.154
Total		17079860	1410403	100.000	100.000

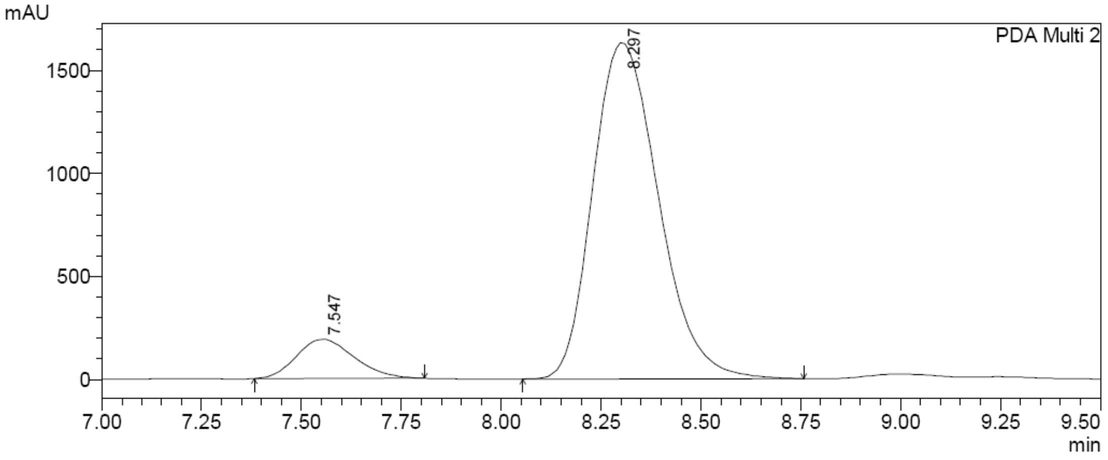

Peak#	Ret. Time	Area	Height	Area %	Height %
1	7.547	1119269	111670	8.695	9.579
2	8.297	11753832	1054062	91.305	90.421
Total		12873101	1165732	100.000	100.000

Supplementary Figure 11. ^{1}H and ^{13}C NMR spectra for compound 3f

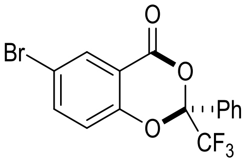

3f: 83%, 92:8 er

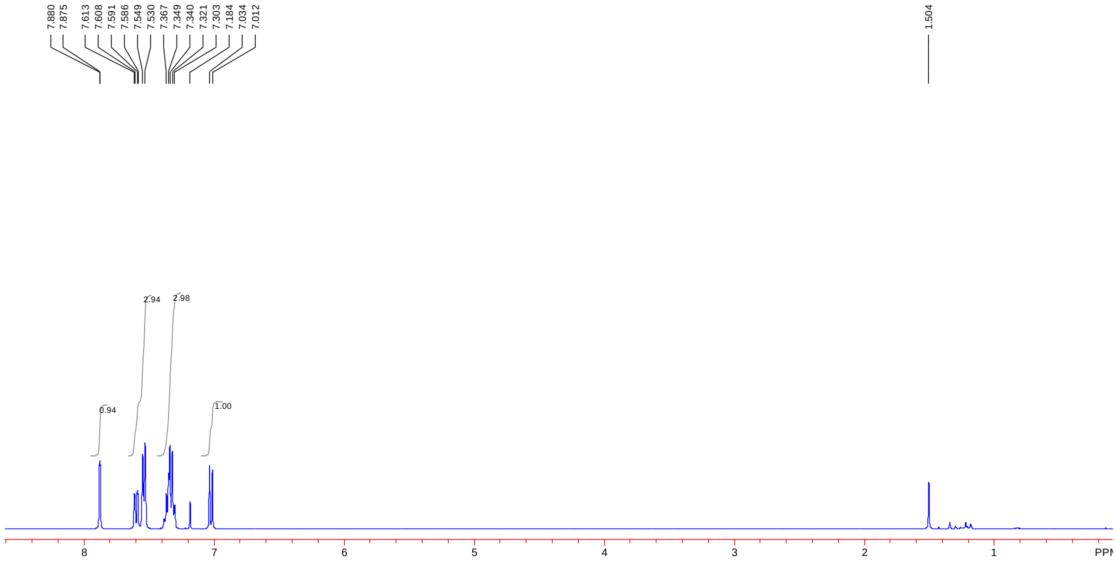

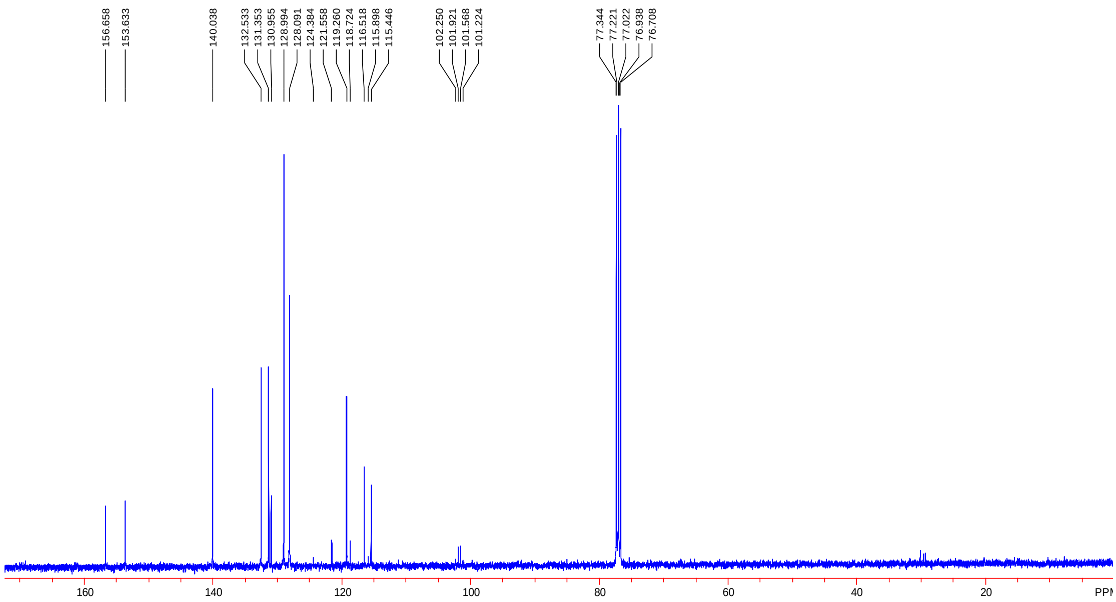

Supplementary Figure 12. ^{19}F and HPLC spectra for compound 3f

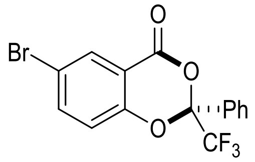

3f: 83%, 92:8 er

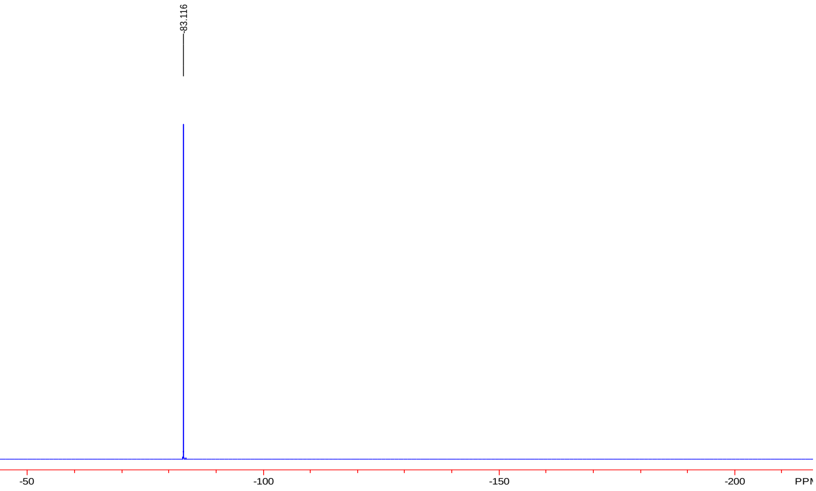

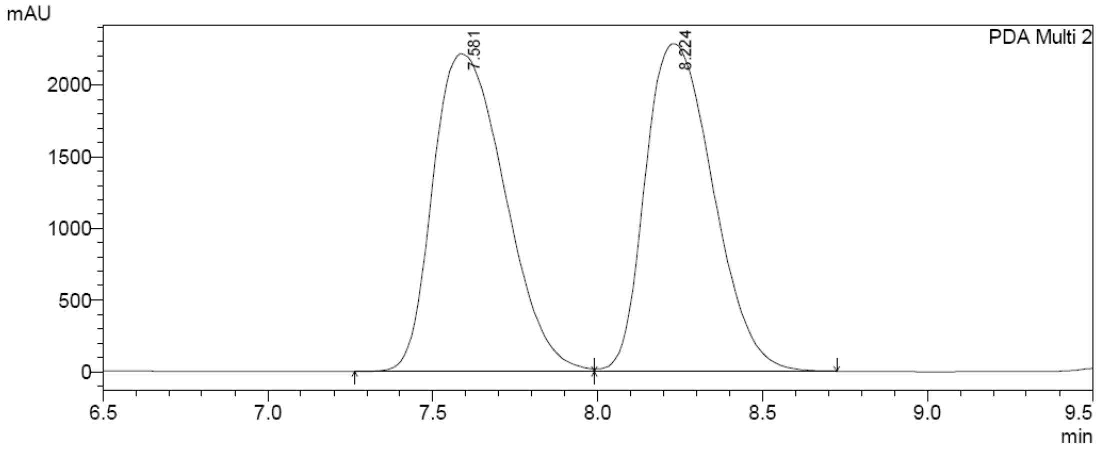

Peak#	Ret. Time	Area	Height	Area %	Height %
1	7.581	33646507	2215602	50.722	49.250
2	8.224	32689208	2283081	49.278	50.750
Total		66335715	4498683	100.000	100.000

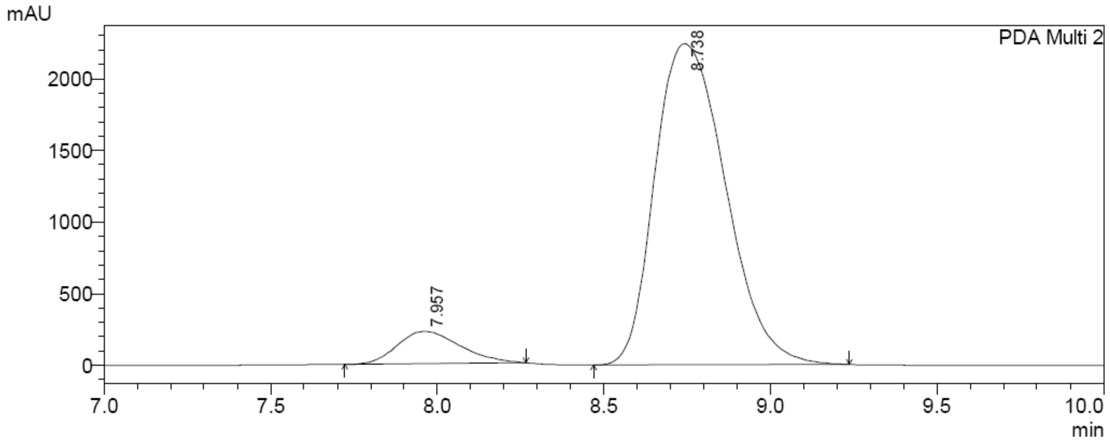

Peak#	Ret. Time	Area	Height	Area %	Height %
1	7.957	2979737	226468	8.230	9.176
2	8.738	33224749	2241646	91.770	90.824
Total		36204486	2468114	100.000	100.000

Supplementary Figure 13. ^{1}H and ^{13}C NMR spectra for compound 3g

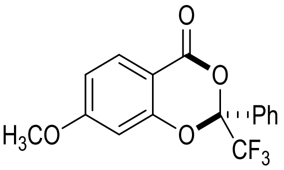

3g: 98%, 94:6 er

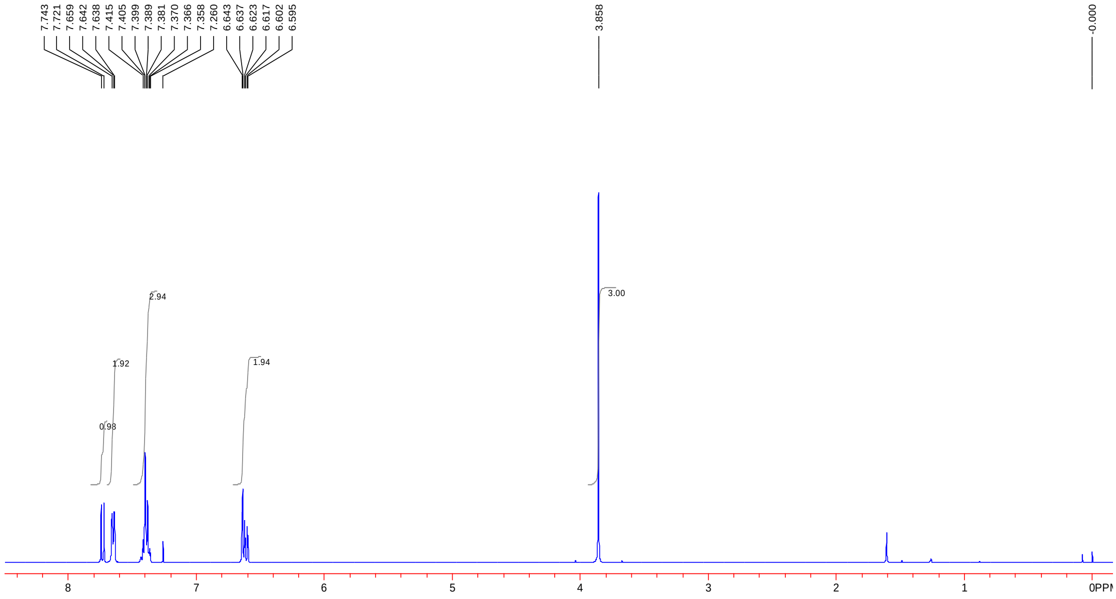

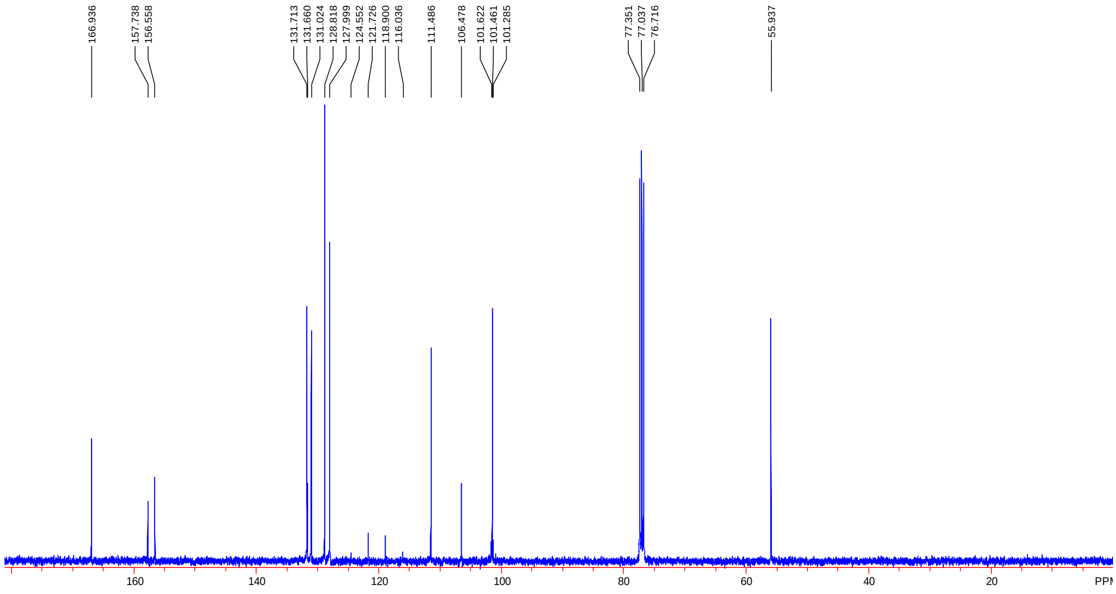

Supplementary Figure 14. ^{19}F and HPLC spectra for compound 3g

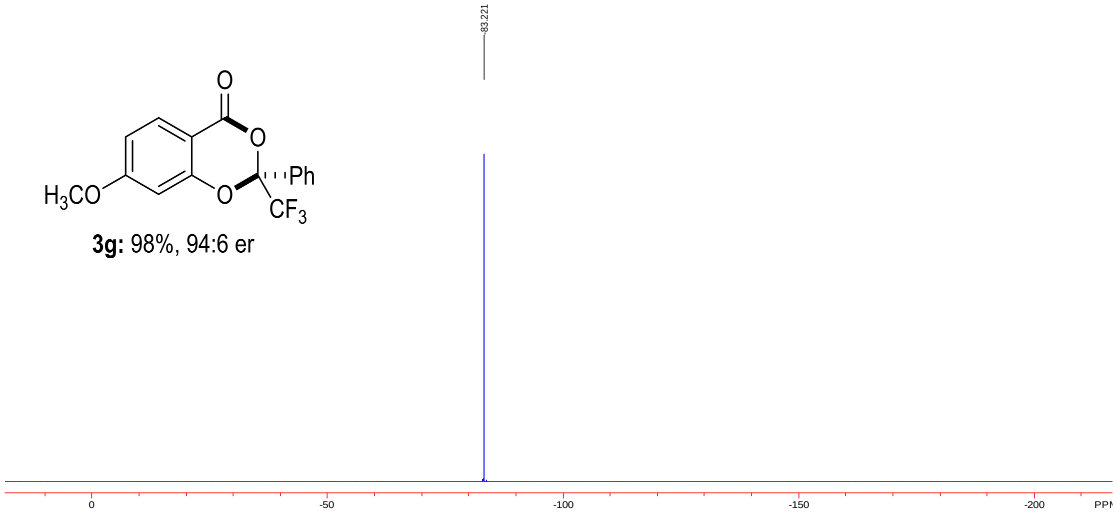

3g: 98%, 94:6 er

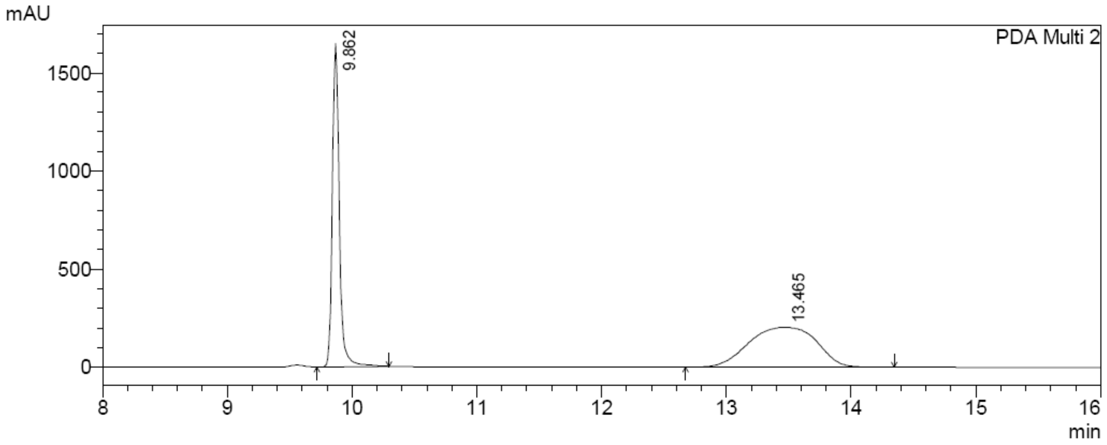

Peak#	Ret. Time	Area	Height	Area %	Height %
1	9.861	4721609	1292823	48.623	90.653
2	13.464	4989073	133304	51.377	9.347
Total		9710682	1426127	100.000	100.000

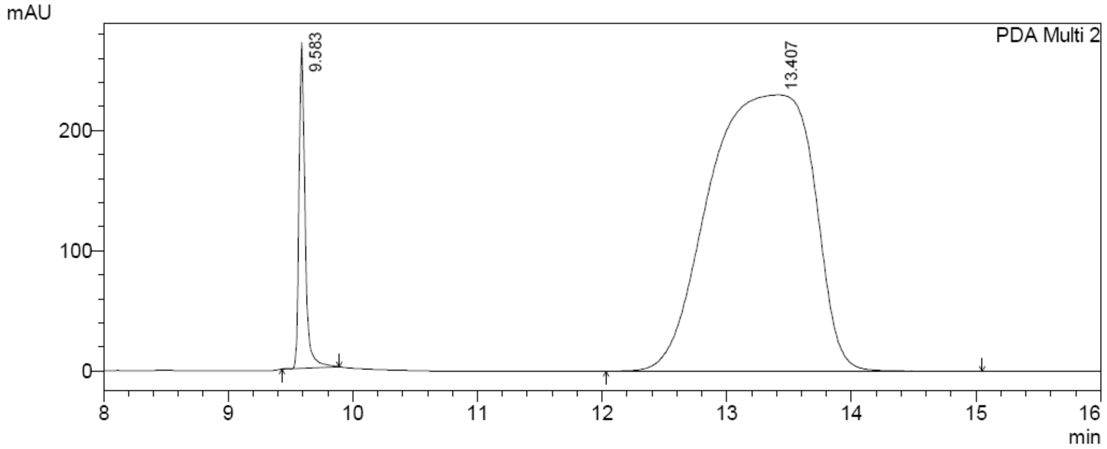

Peak#	Ret. Time	Area	Height	Area %	Height %
1	9.583	588837	175822	6.296	53.846
2	13.405	8764053	150703	93.704	46.154
Total		9352890	326526	100.000	100.000

Supplementary Figure 15. ^{1}H and ^{13}C NMR spectra for compound 3h

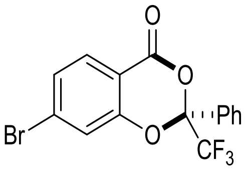

3h:87%, 91:9 er

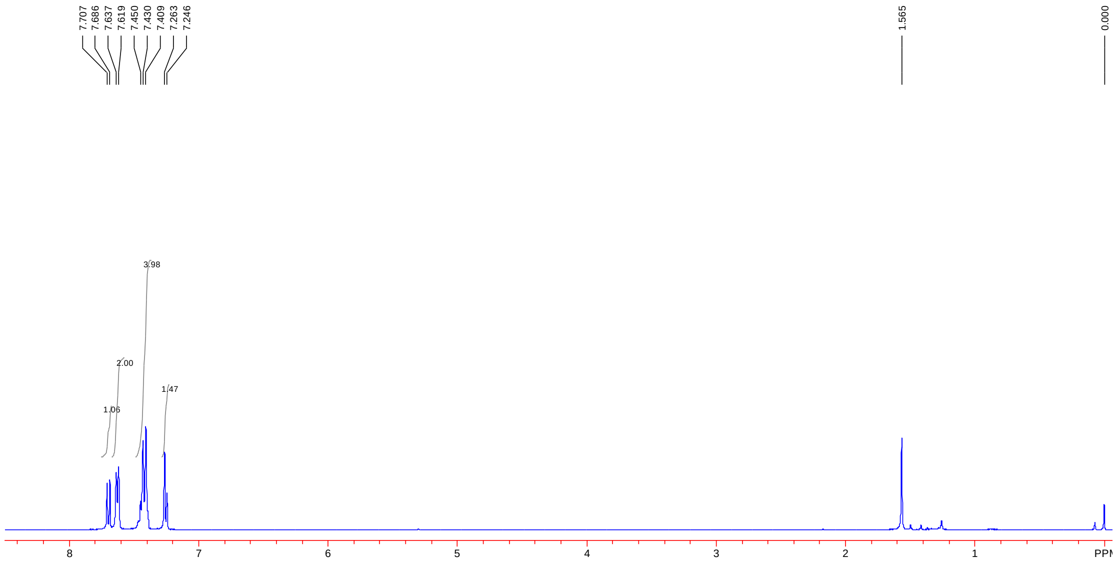

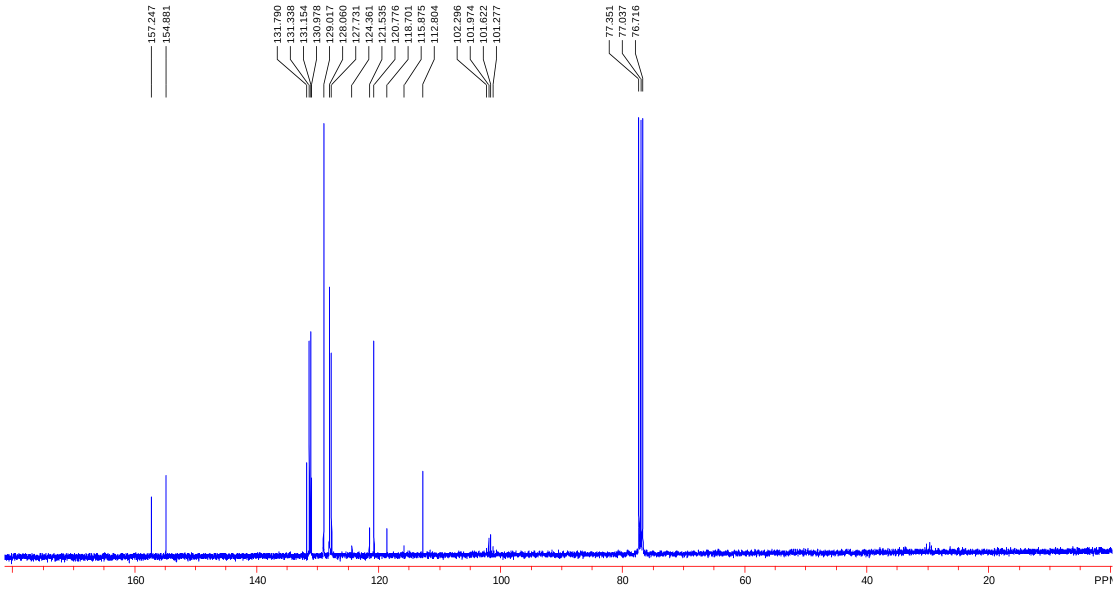

Supplementary Figure 16. ^{19}F and HPLC spectra for compound 3h

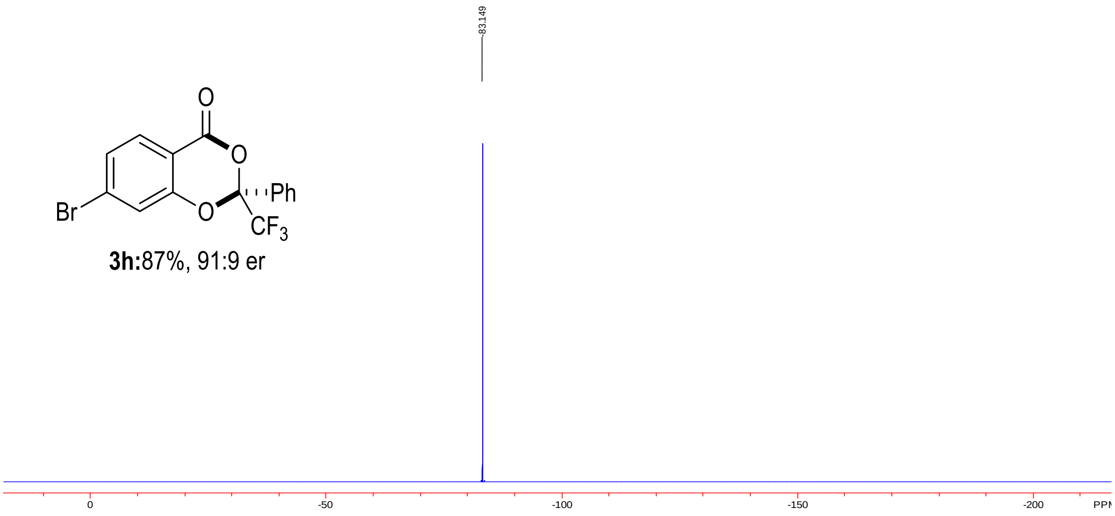

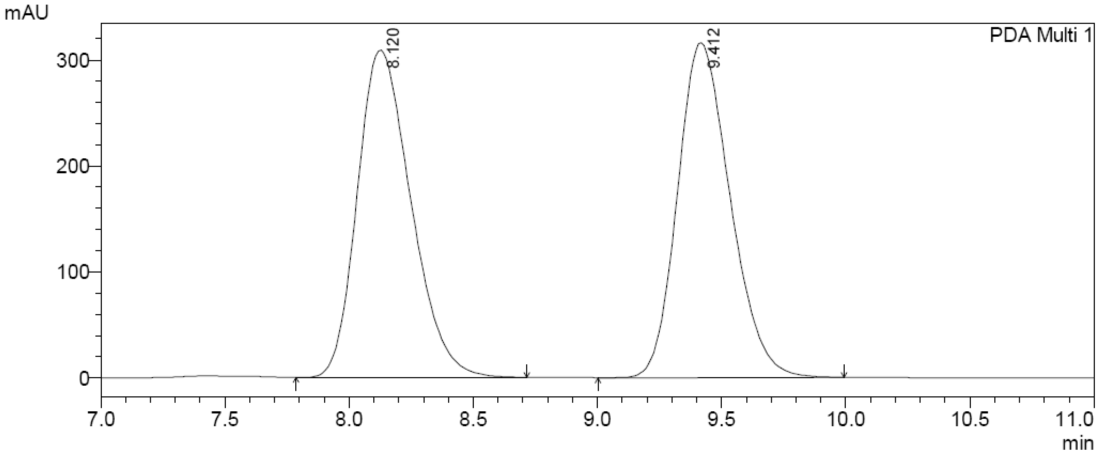

Peak#	Ret. Time	Area	Height	Area %	Height %
1	8.120	4653482	309073	49.914	49.425
2	9.412	4669554	316265	50.086	50.575
Total		9323035	625338	100.000	100.000

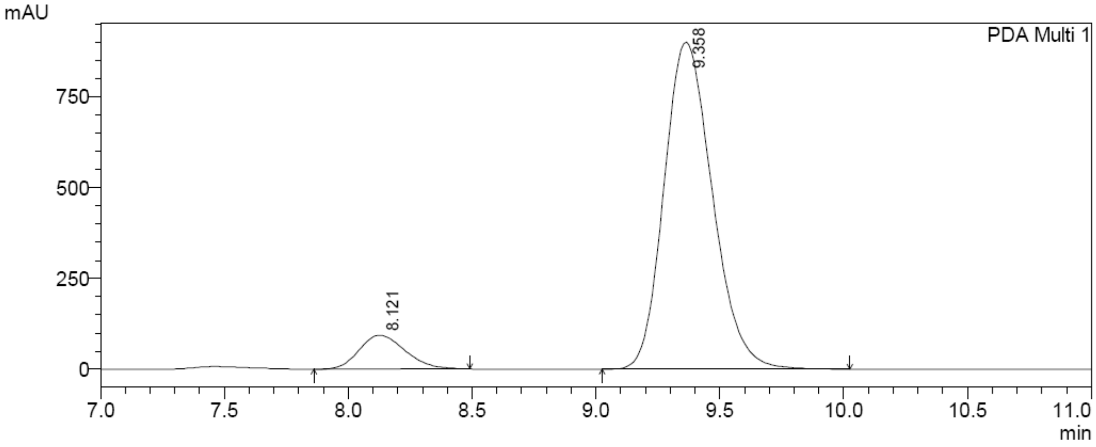

Peak#	Ret. Time	Area	Height	Area %	Height %
1	8.121	1202326	93253	8.948	9.383
2	9.358	12233821	900644	91.052	90.617
Total		13436148	993897	100.000	100.000

Supplementary Figure 17. ^{1}H and ^{13}C NMR spectra for compound 3i

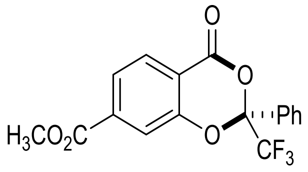

3i: 93%, 90:10 er

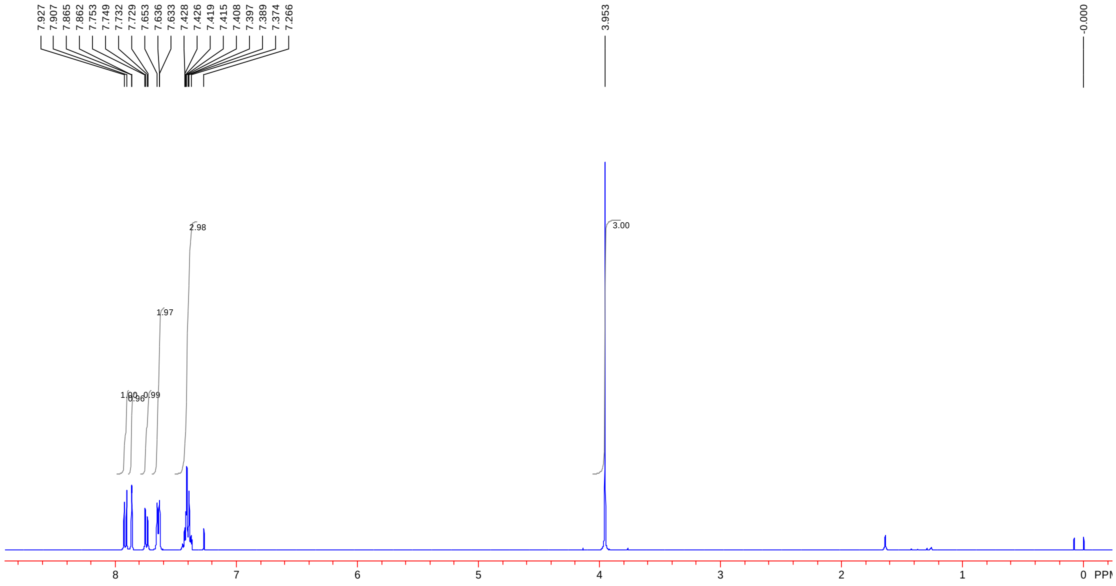

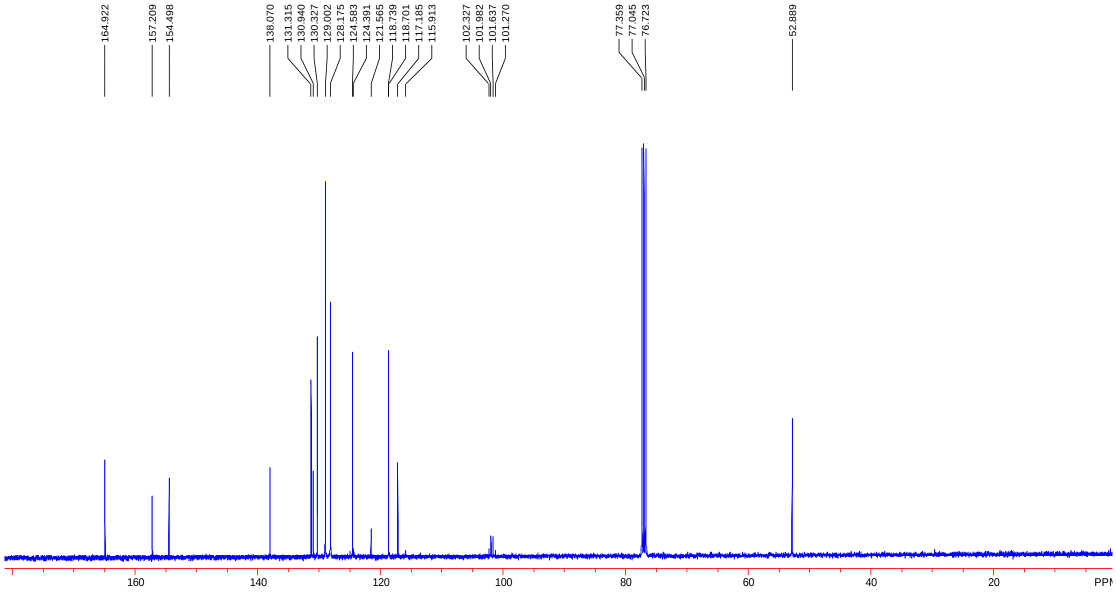

Supplementary Figure 18. ^{19}**F and HPLC spectra for compound 3i**

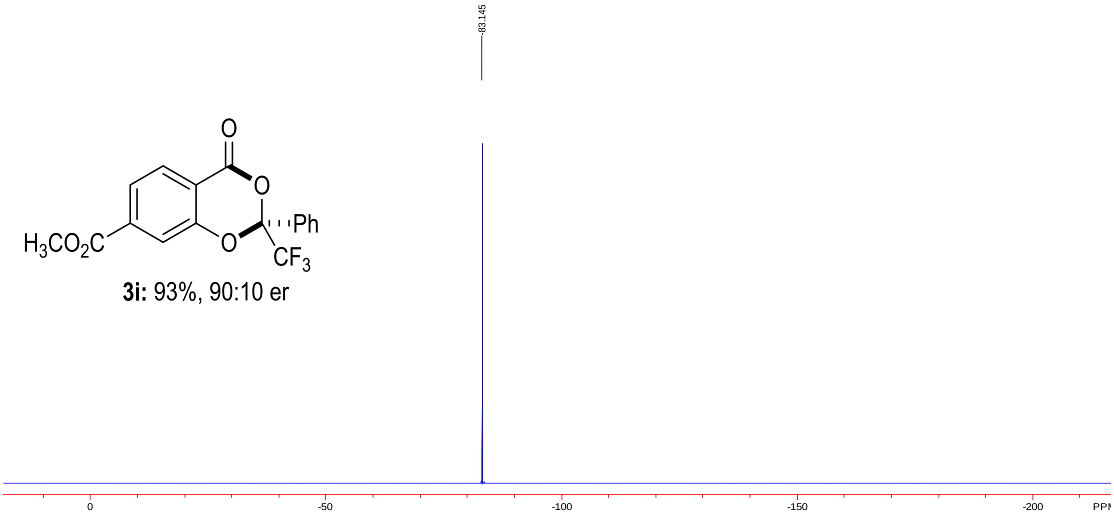

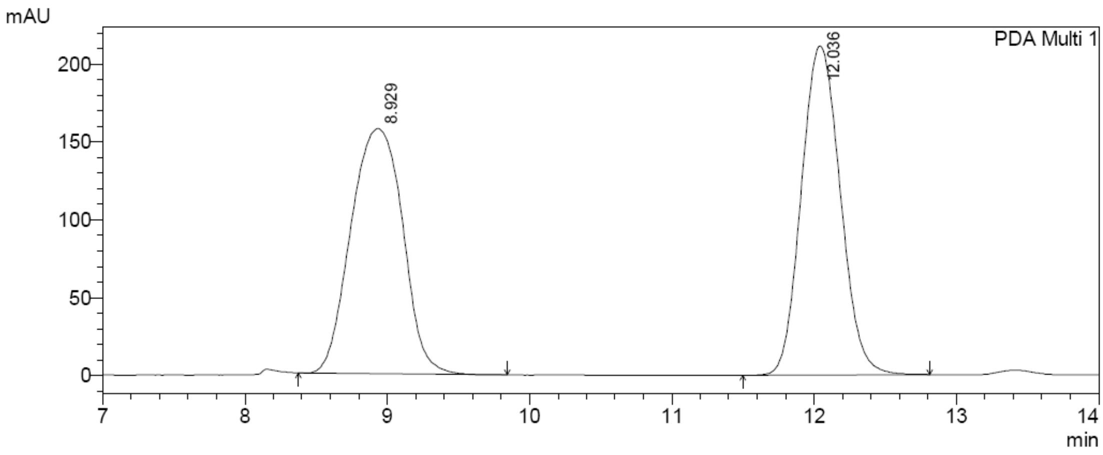

Peak#	Ret. Time	Area	Height	Area %	Height %
1	8.929	3997656	157404	49.833	42.684
2	12.036	4024388	211364	50.167	57.316
Total		8022043	368768	100.000	100.000

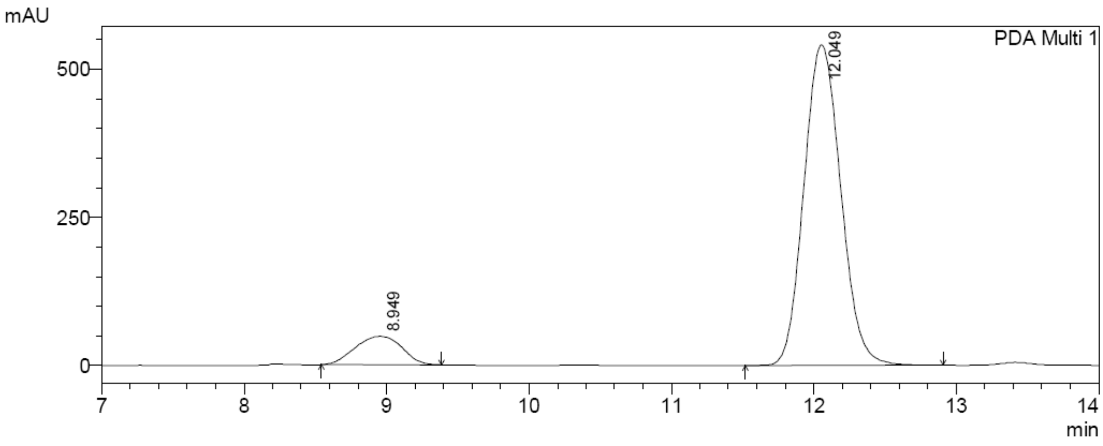

Peak#	Ret. Time	Area	Height	Area %	Height %
1	8.949	1092497	47793	10.087	8.115
2	12.049	9738370	541126	89.913	91.885
Total		10830867	588919	100.000	100.000

Supplementary Figure 19. ^{1}H and ^{13}C NMR spectra for compound 3j

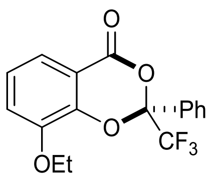

3j: 87%, 97:3 er

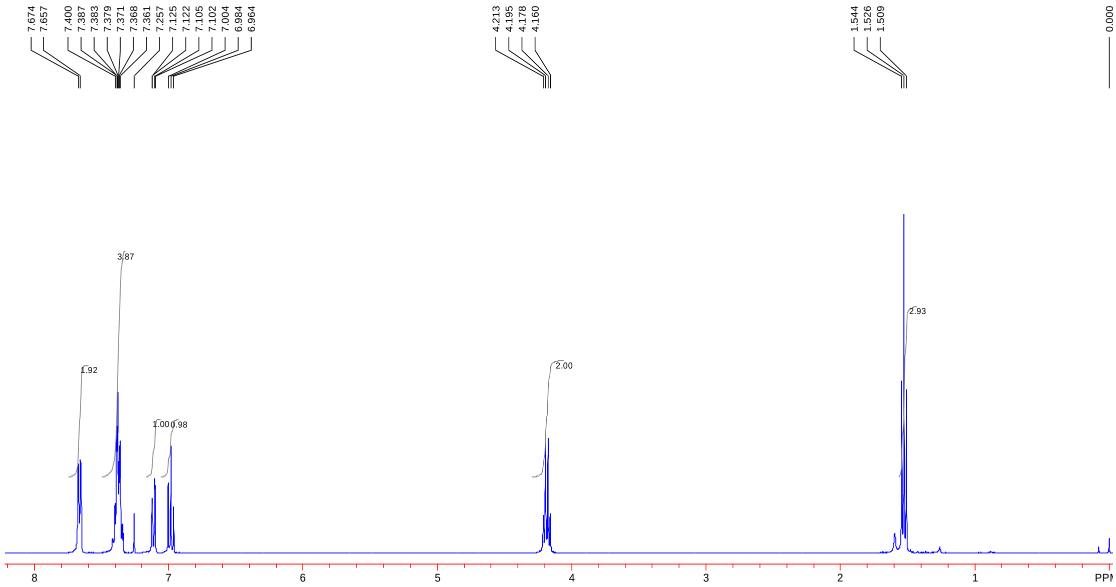

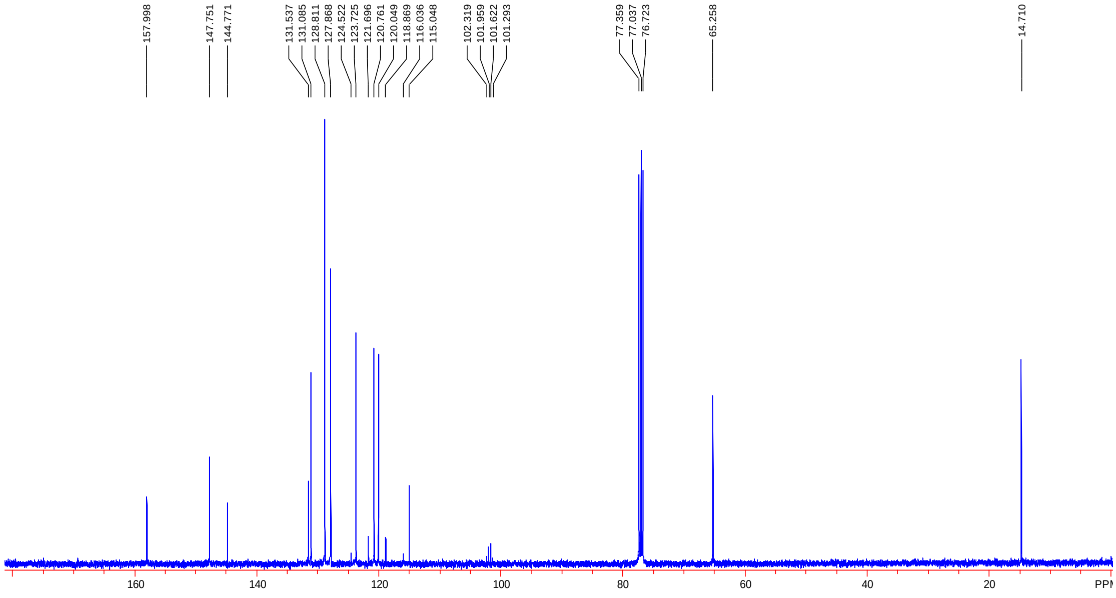

Supplementary Figure 20. ^{19}F and HPLC spectra for compound 3j

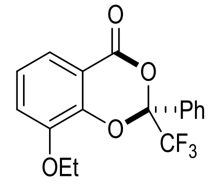

3j: 87%, 97:3 er

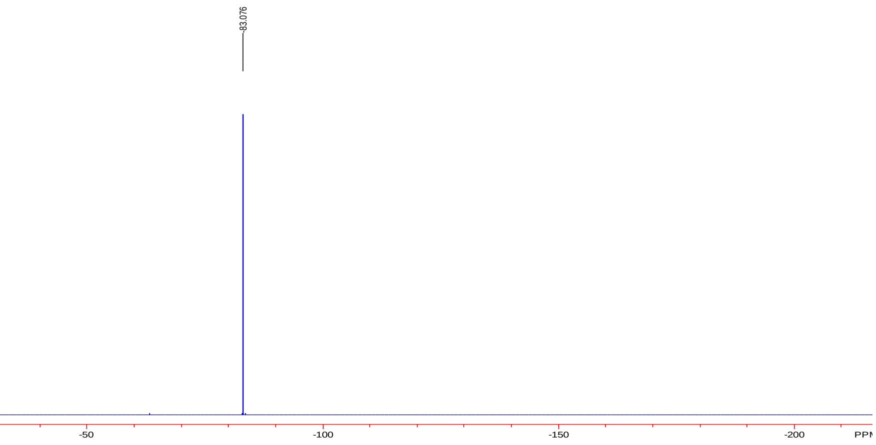

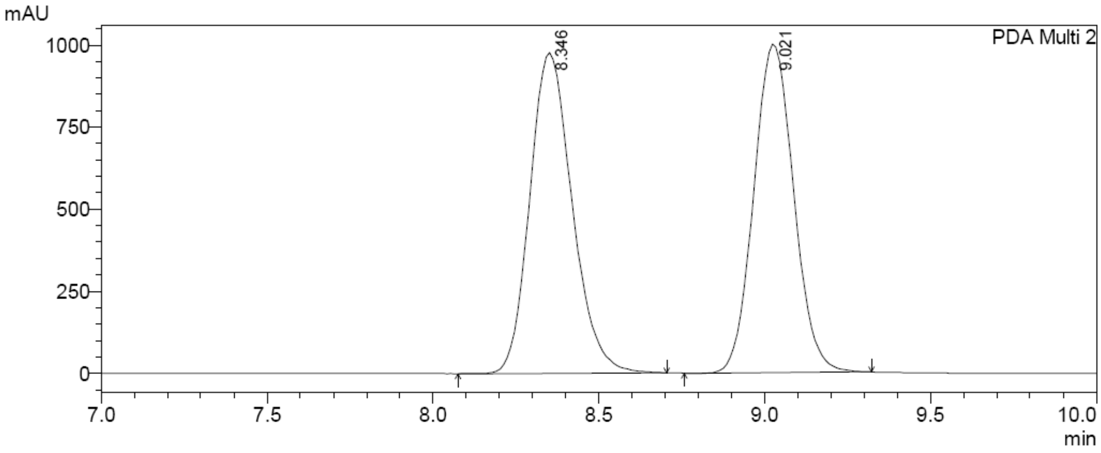

Peak#	Ret. Time	Area	Height	Area %	Height %
1	8.344	1993692	232758	50.084	48.841
2	9.021	1987006	243809	49.916	51.159
Total		3980698	476566	100.000	100.000

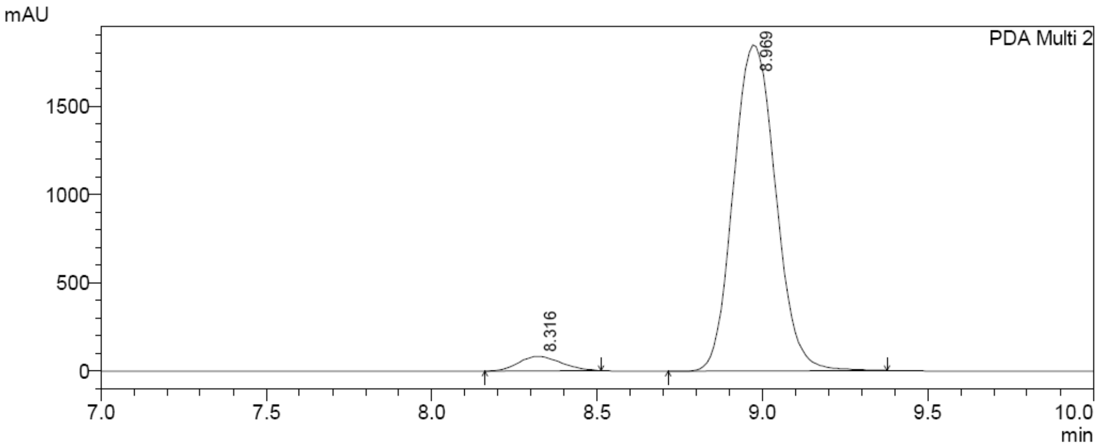

Peak#	Ret. Time	Area	Height	Area %	Height %
1	8.306	130322	15831	2.933	2.900
2	8.969	4312654	530124	97.067	97.100
Total		4442976	545956	100.000	100.000

Supplementary Figure 21. ^{1}**H and** ^{13}**C NMR spectra for compound 3k**

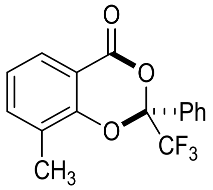

3k: 86%, 93:7 er

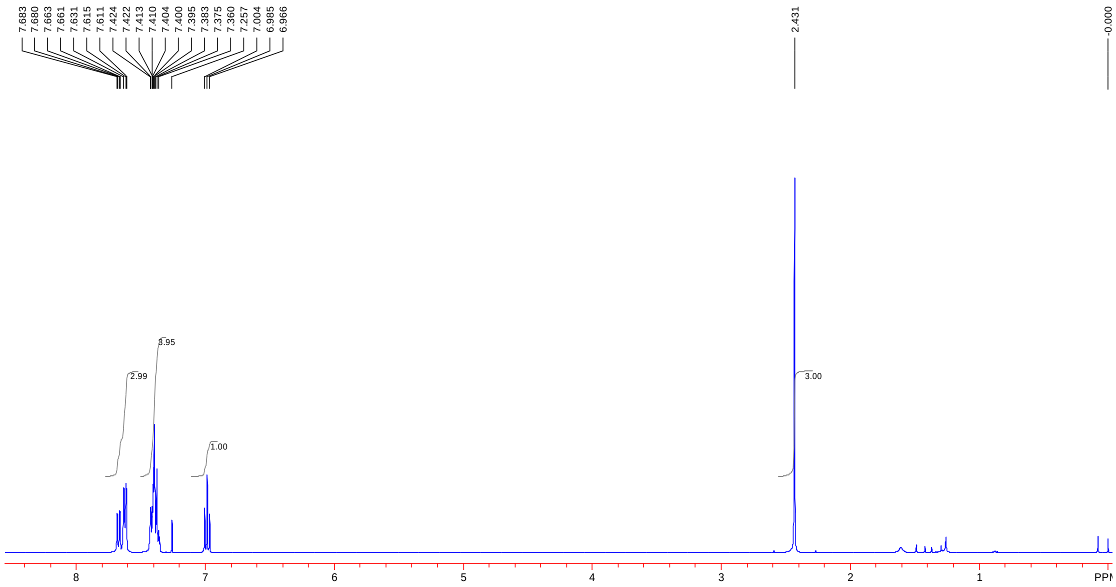

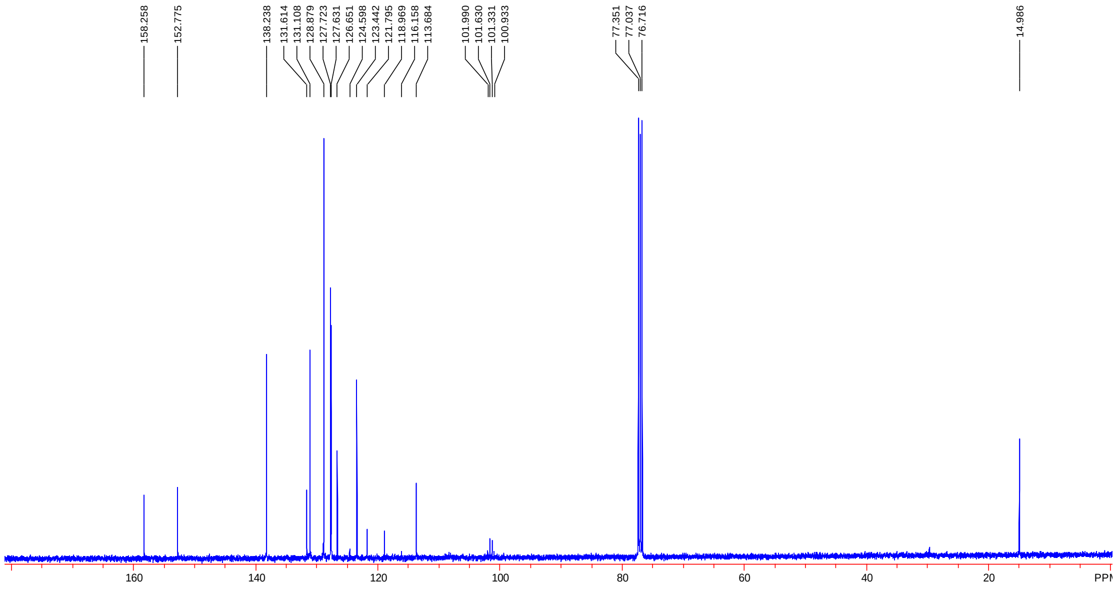

Supplementary Figure 22. ^{19}F and HPLC spectra for compound 3k

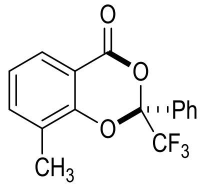

3k: 86%, 93:7 er

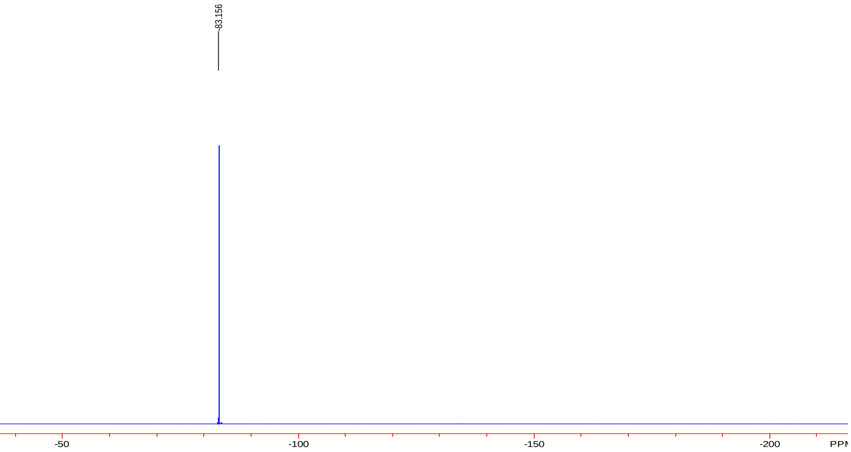

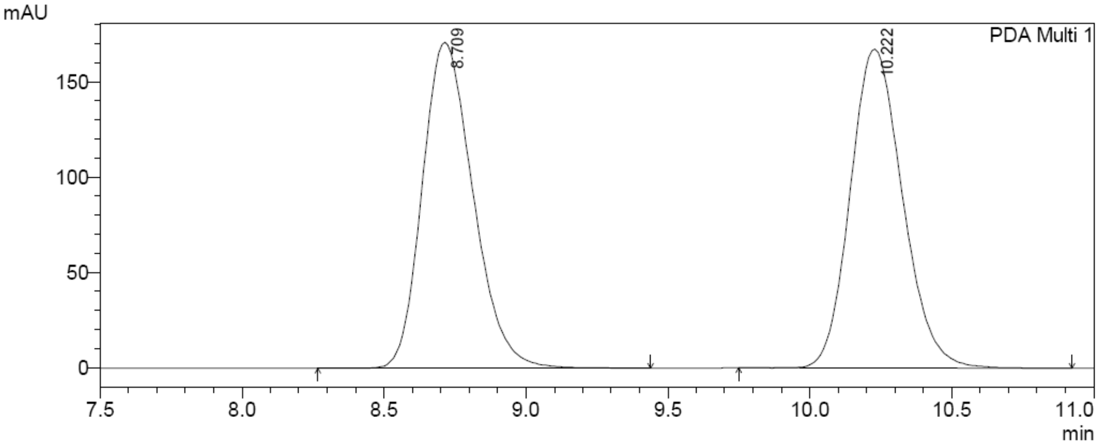

Peak#	Ret. Time	Area	Height	Area %	Height %
1	8.709	2127916	170839	49.835	50.535
2	10.222	2141990	167221	50.165	49.465
Total		4269906	338060	100.000	100.000

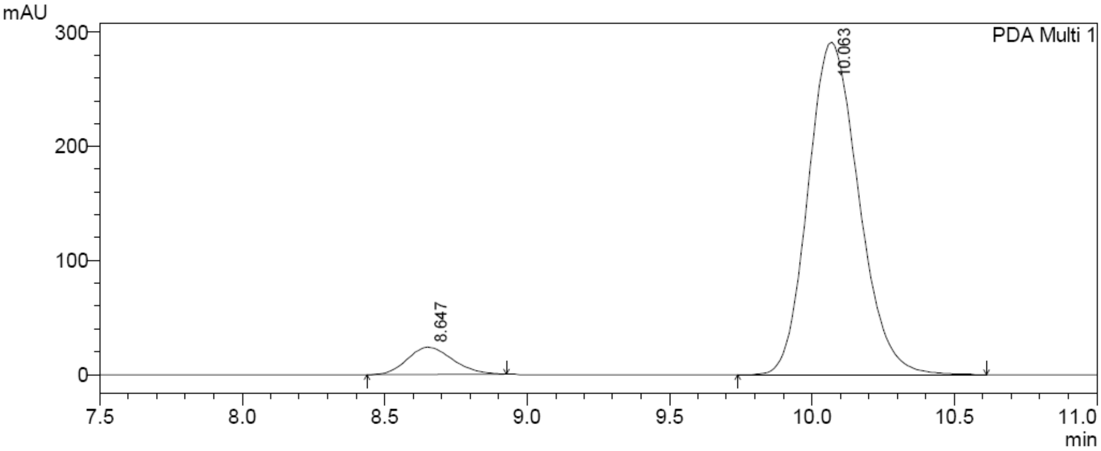

Peak#	Ret. Time	Area	Height	Area %	Height %
1	8.647	264680	23797	6.836	7.554
2	10.063	3607389	291207	93.164	92.446
Total		3872069	315003	100.000	100.000

Supplementary Figure 23. ^{1}H and ^{13}C NMR spectra for compound 3l

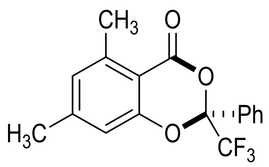

3l: 84%, 98:2 er

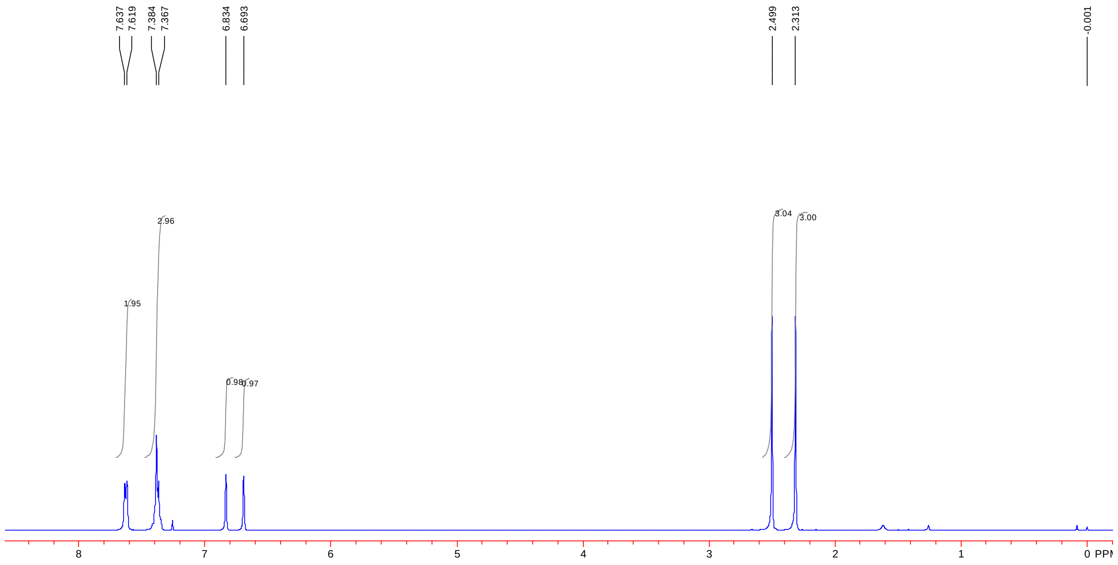

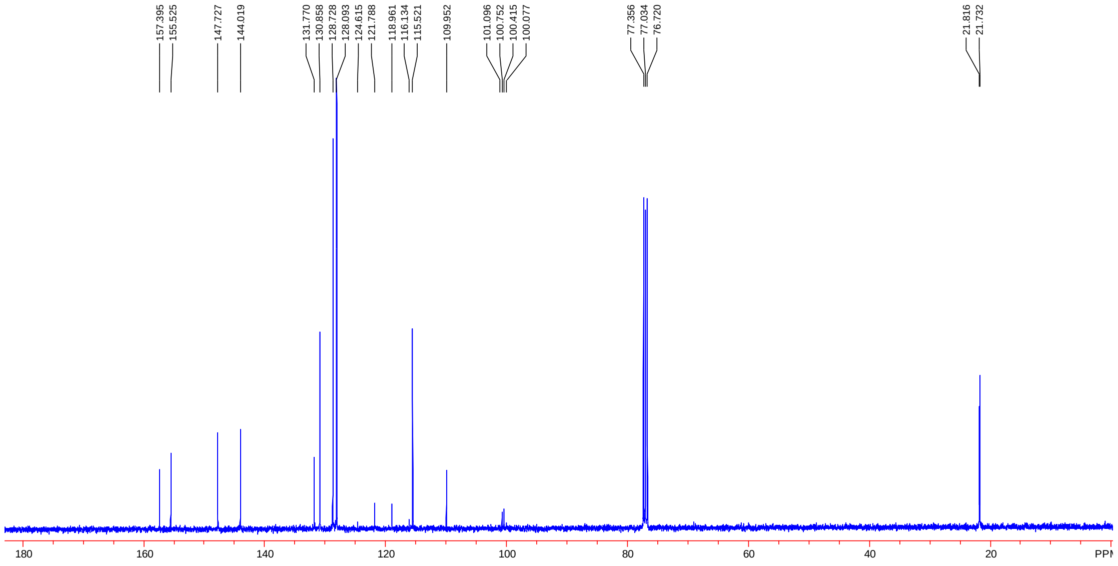

Supplementary Figure 24. ^{19}F and HPLC spectra for compound 3l

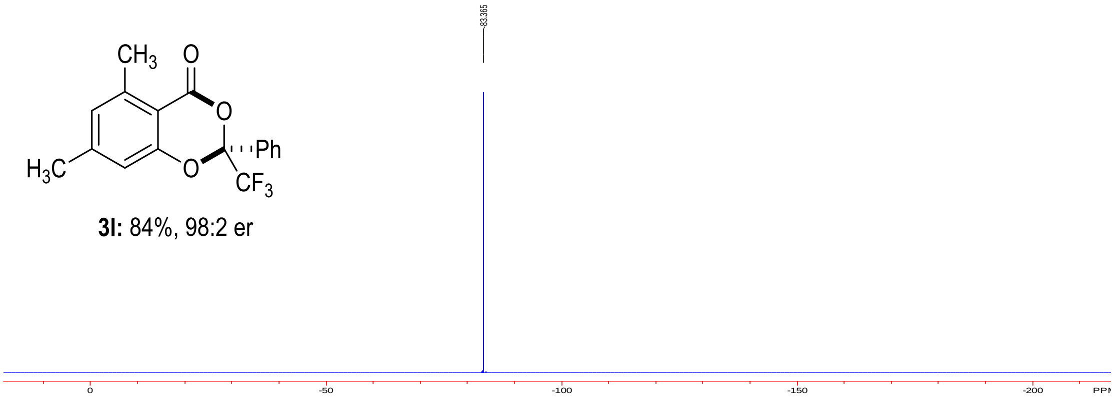

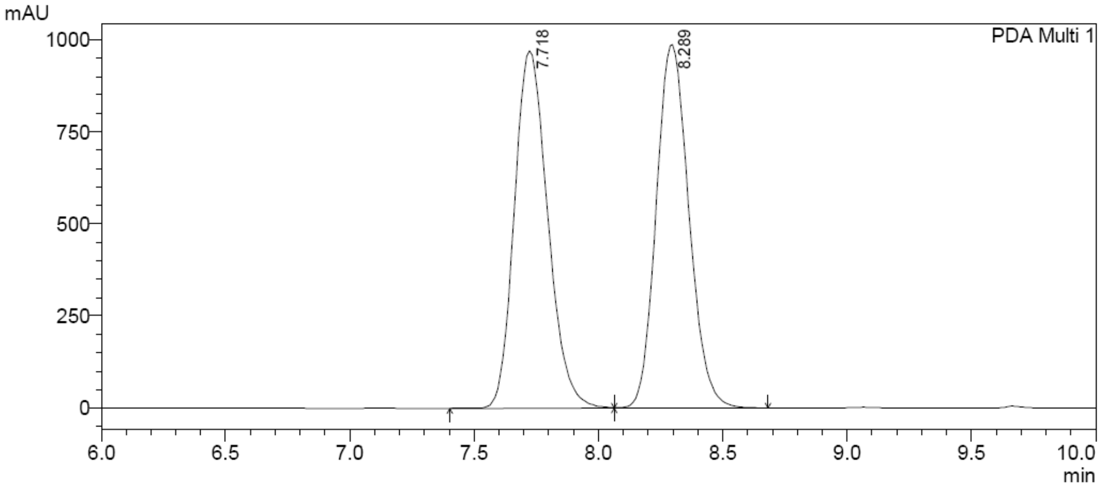

Peak#	Ret. Time	Area	Height	Area %	Height %
1	7.718	16311797	1552748	50.146	49.795
2	8.289	16216627	1565539	49.854	50.205
Total		32528425	3118287	100.000	100.000

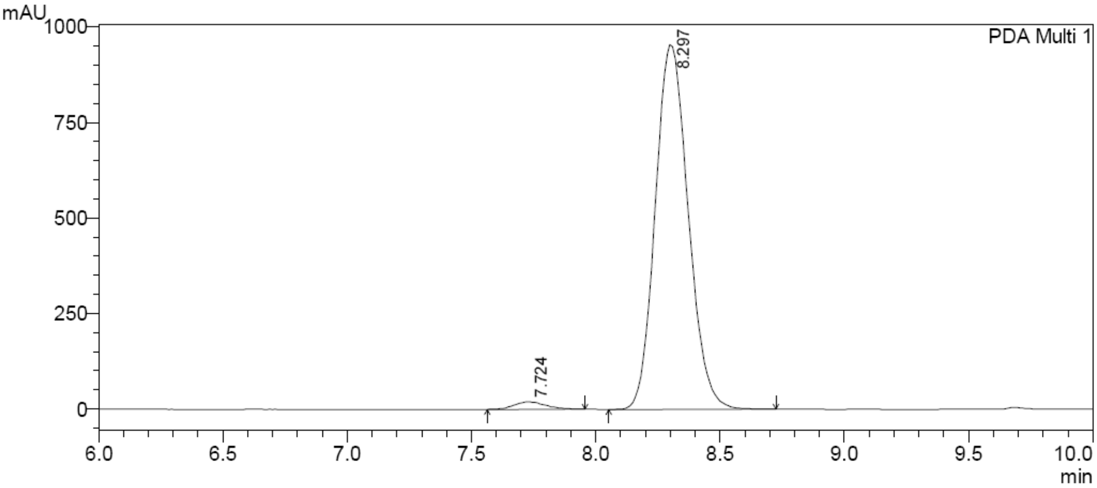

Peak#	Ret. Time	Area	Height	Area %	Height %
1	7.724	172699	19500	1.924	2.005
2	8.297	8804600	953106	98.076	97.995
Total		8977299	972605	100.000	100.000

Supplementary Figure 25. ^{1}H and ^{13}C NMR spectra for compound 3m

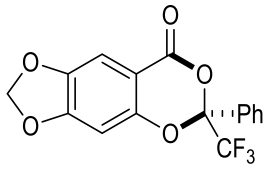

3m: 91%, 97:3 er

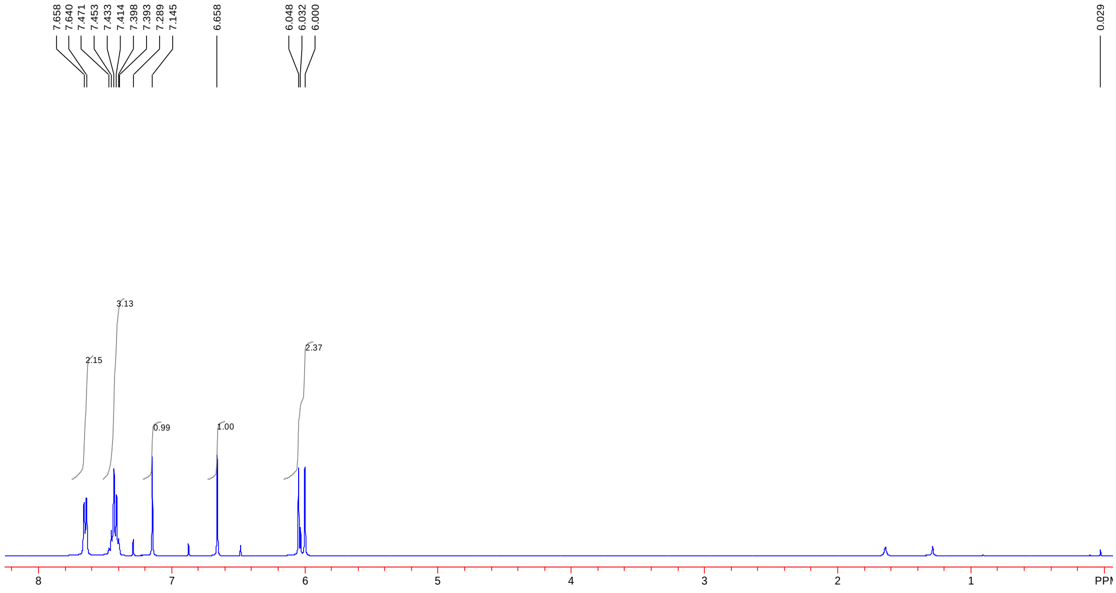

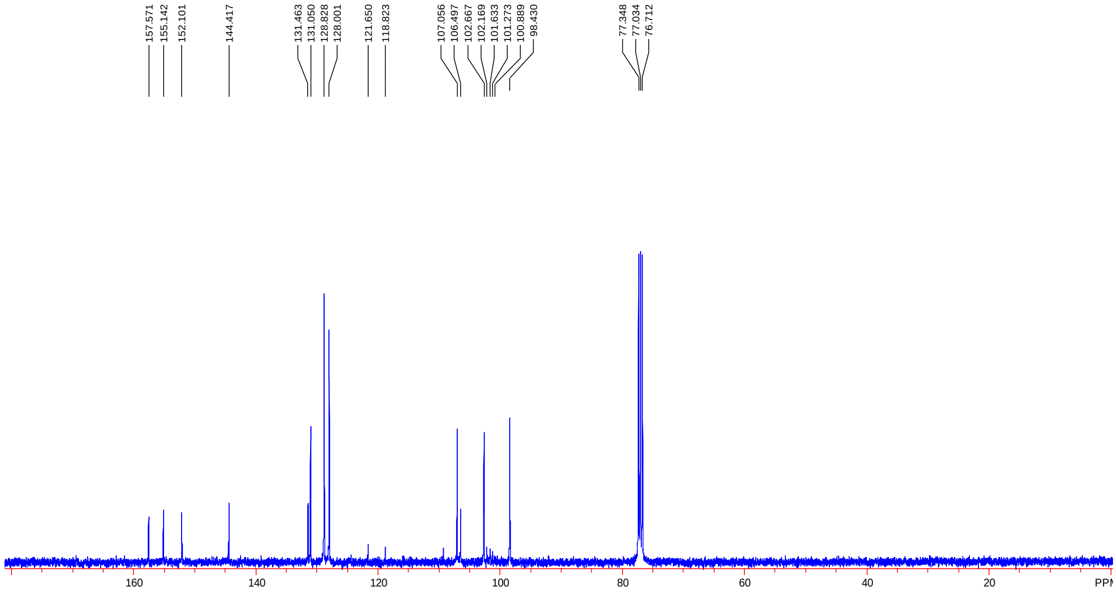

Supplementary Figure 26. ^{19}F and HPLC spectra for compound 3m

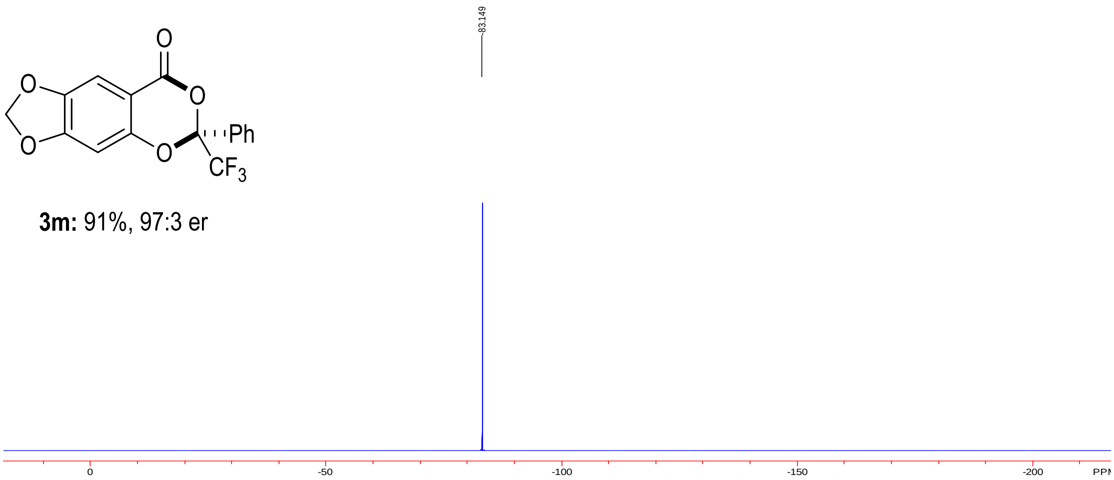

3m: 91%, 97:3 er

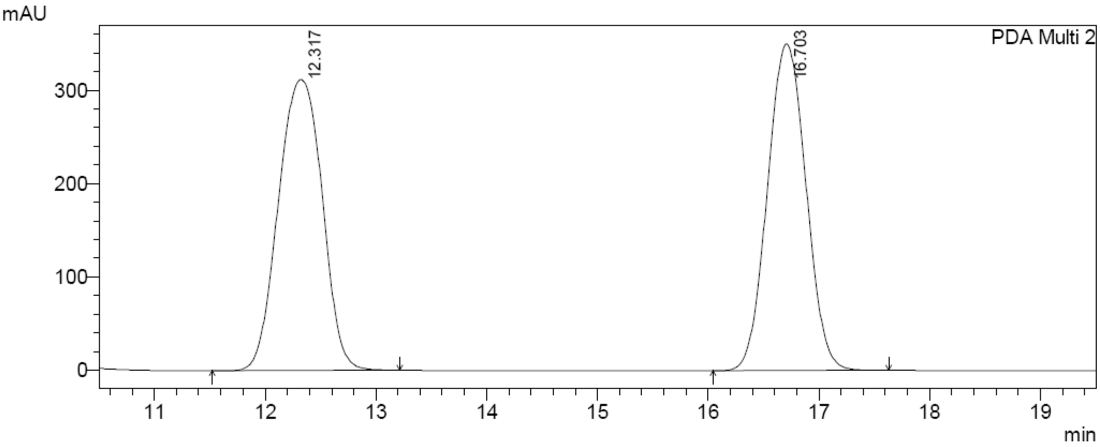

Peak#	Ret. Time	Area	Height	Area %	Height %
1	12.317	2318085	84104	50.354	47.082
2	16.704	2285522	94529	49.646	52.918
Total		4603607	178633	100.000	100.000

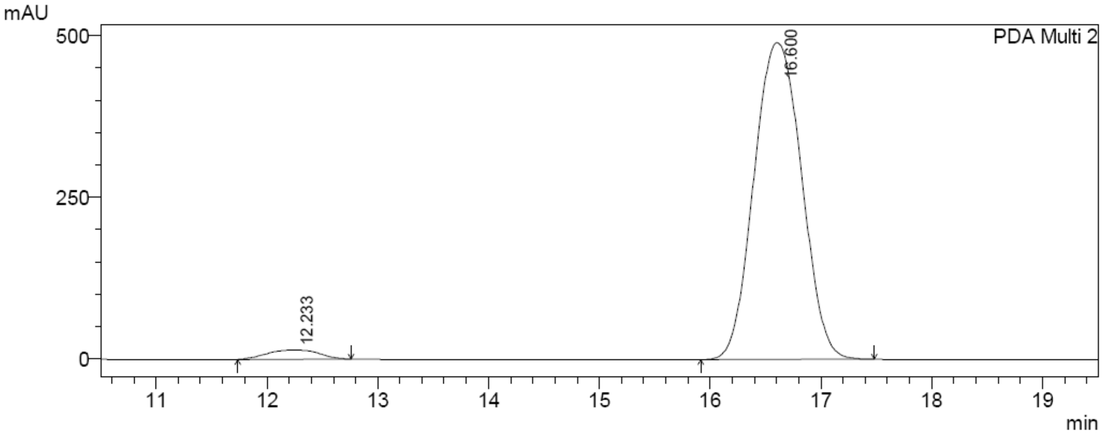

Peak#	Ret. Time	Area	Height	Area %	Height %
1	12.260	121131	3713	2.964	2.720
2	16.600	3965583	132805	97.036	97.280
Total		4086714	136518	100.000	100.000

Supplementary Figure 27. ^{1}H and ^{13}C NMR spectra for compound 3n

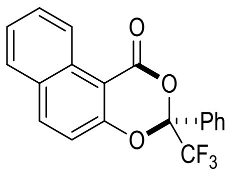

3n: 92%, 98:2 er

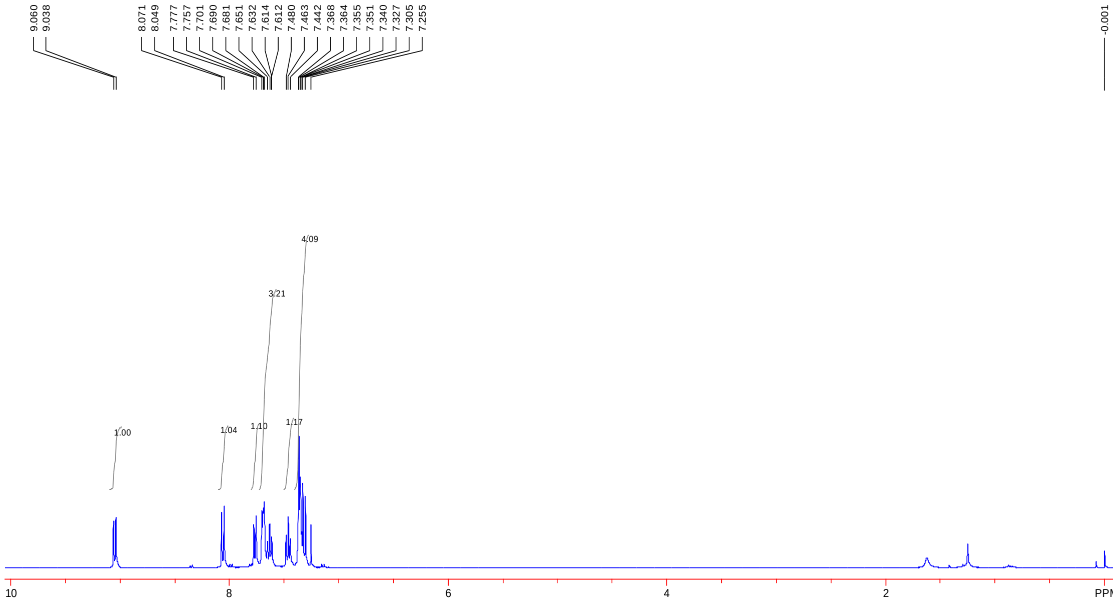

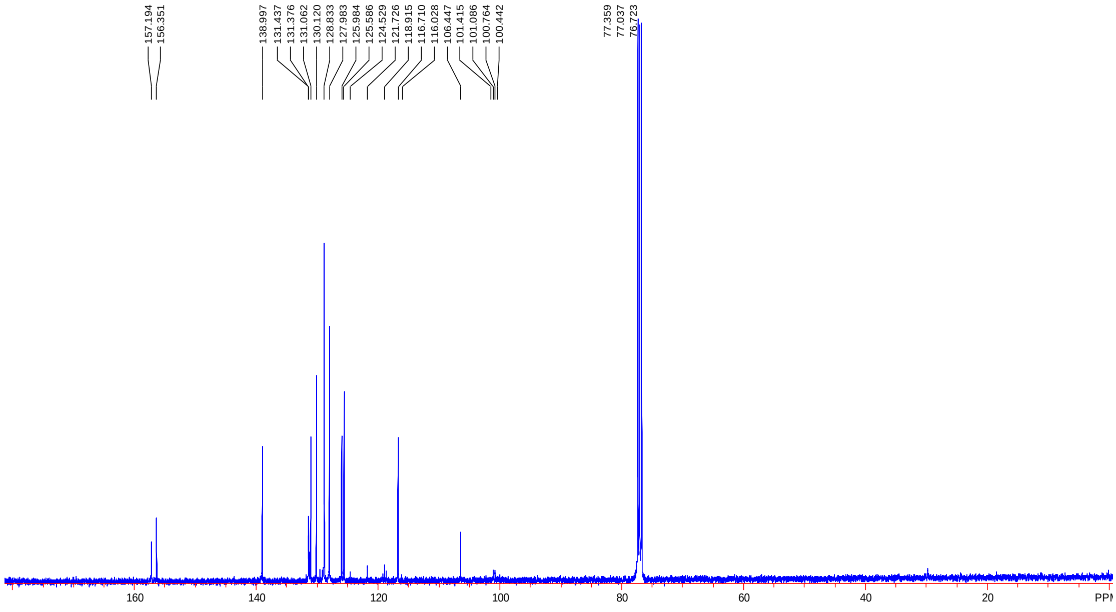

Supplementary Figure 28. ^{19}F and HPLC spectra for compound 3n

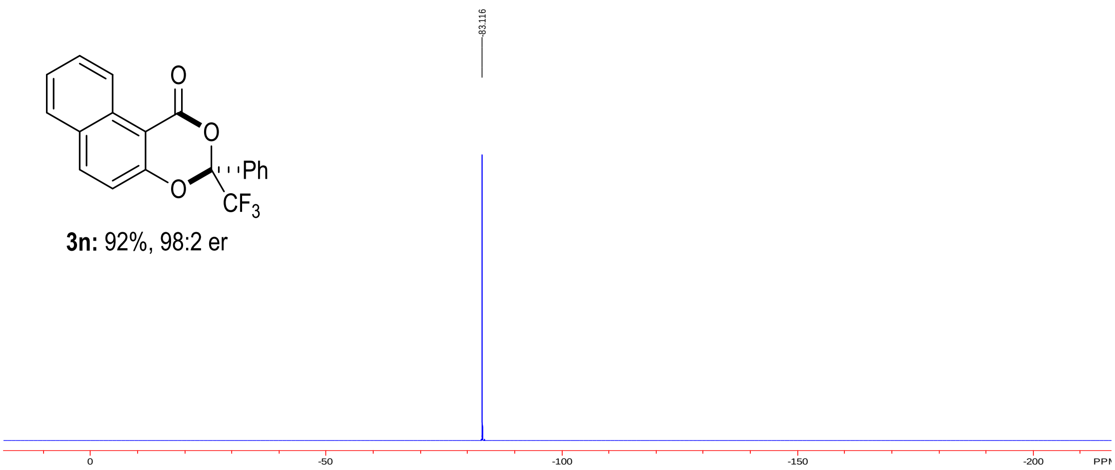

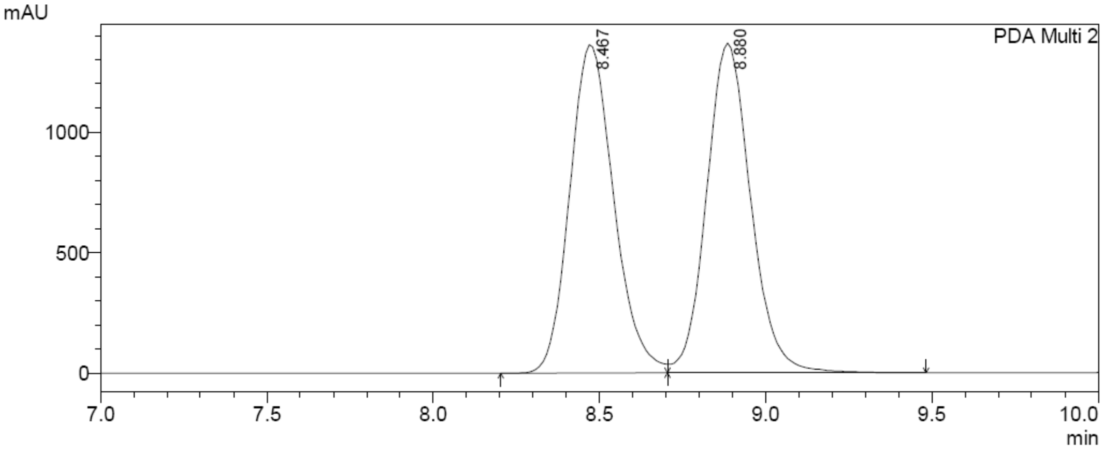

Peak#	Ret. Time	Area	Height	Area %	Height %
1	8.467	12569184	1362438	49.817	49.886
2	8.880	12661640	1368668	50.183	50.114
Total		25230824	2731106	100.000	100.000

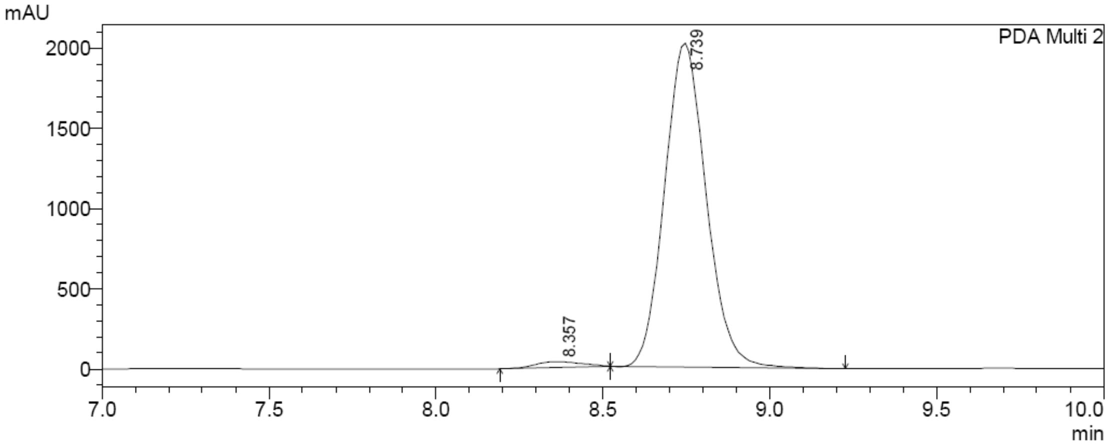

Peak#	Ret. Time	Area	Height	Area %	Height %
1	8.357	357167	37769	1.996	1.836
2	8.739	17541427	2019363	98.004	98.164
Total		17898594	2057132	100.000	100.000

Supplementary Figure 29. ^{1}H and ^{13}C NMR spectra for compound 3o

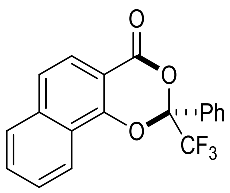

3o: 94%, 95:5 er

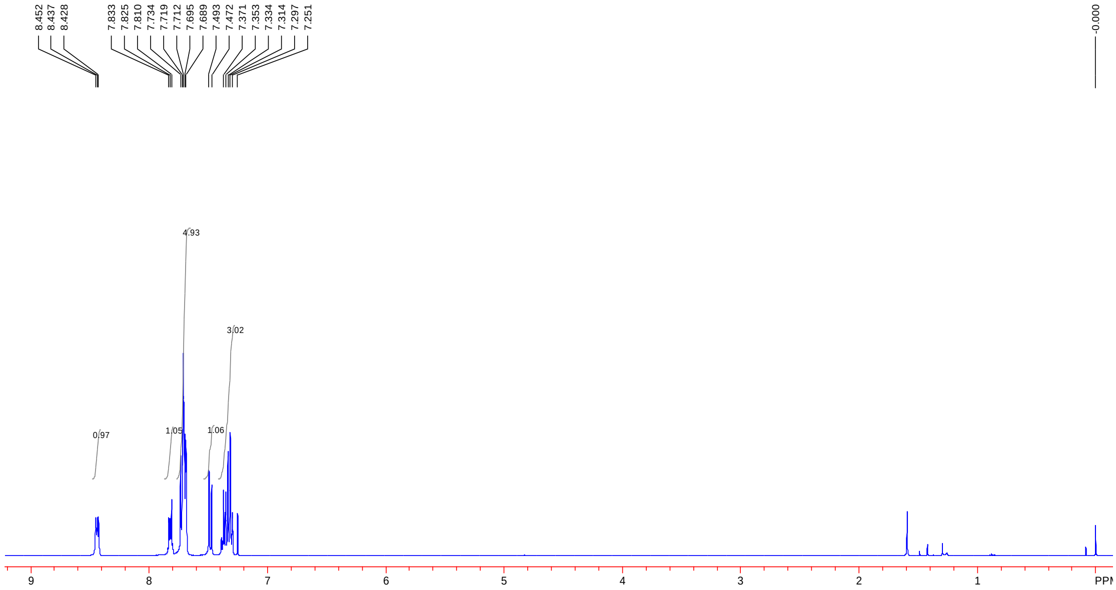

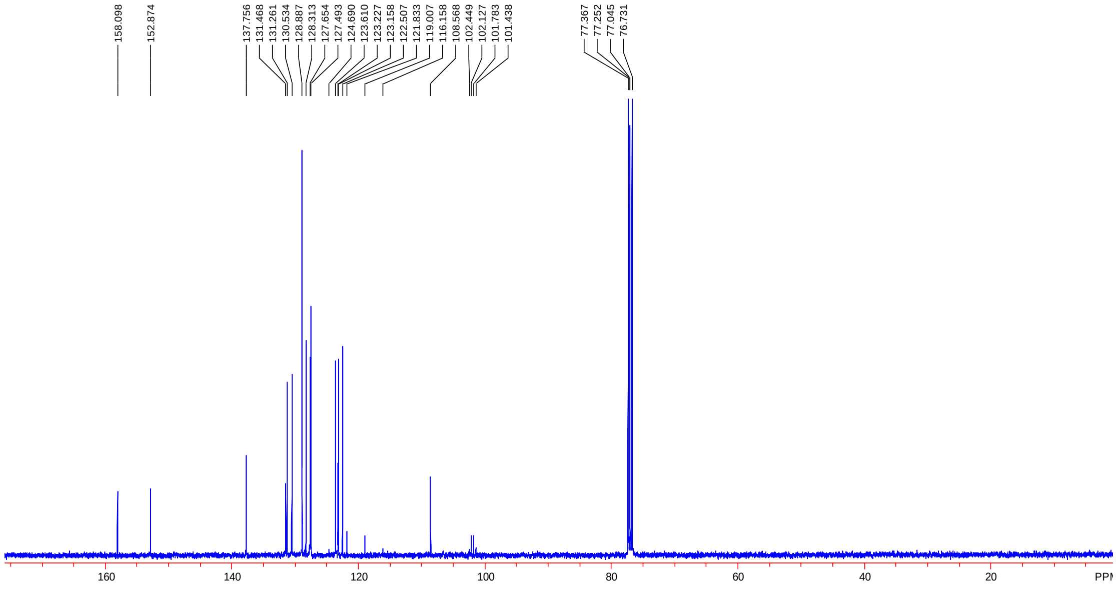

Supplementary Figure 30. ^{19}F and HPLC spectra for compound 3o

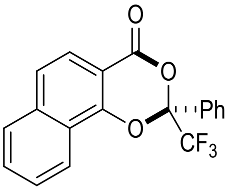

3o: 94%, 95:5 er

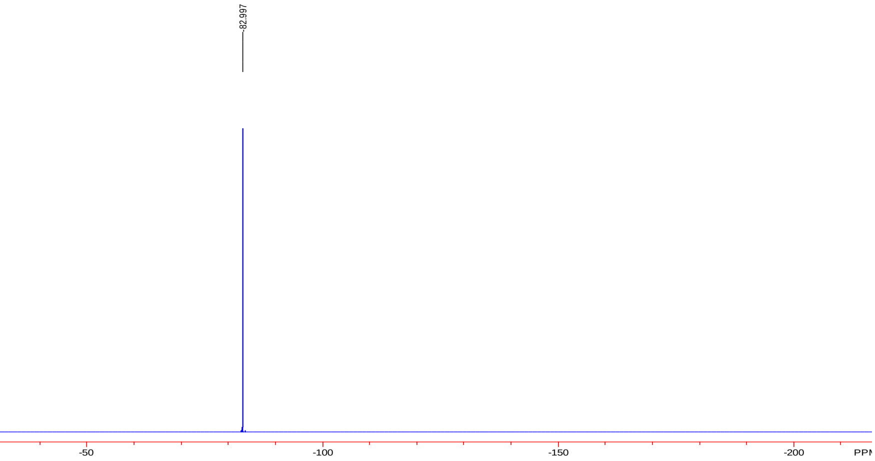

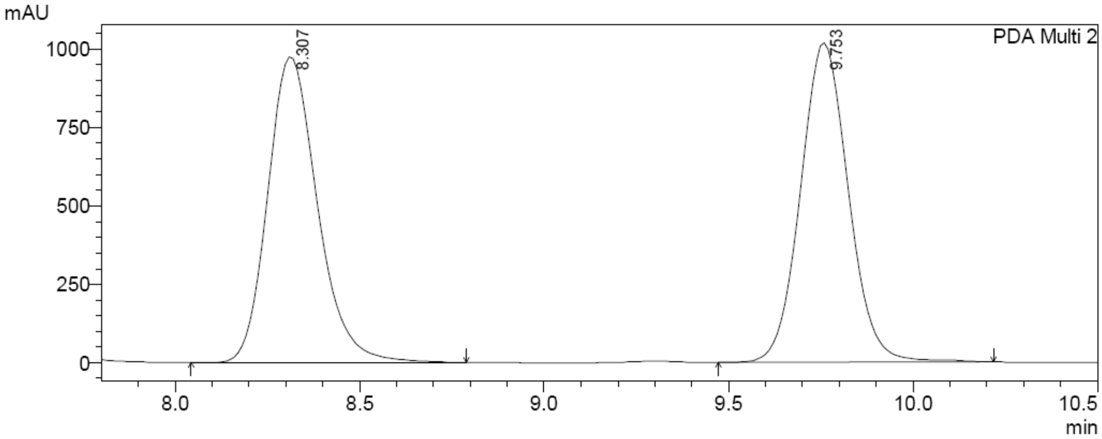

Peak#	Ret. Time	Area	Height	Area %	Height %
1	8.307	9137017	974237	49.762	48.882
2	9.753	9224295	1018809	50.238	51.118
Total		18361312	1993046	100.000	100.000

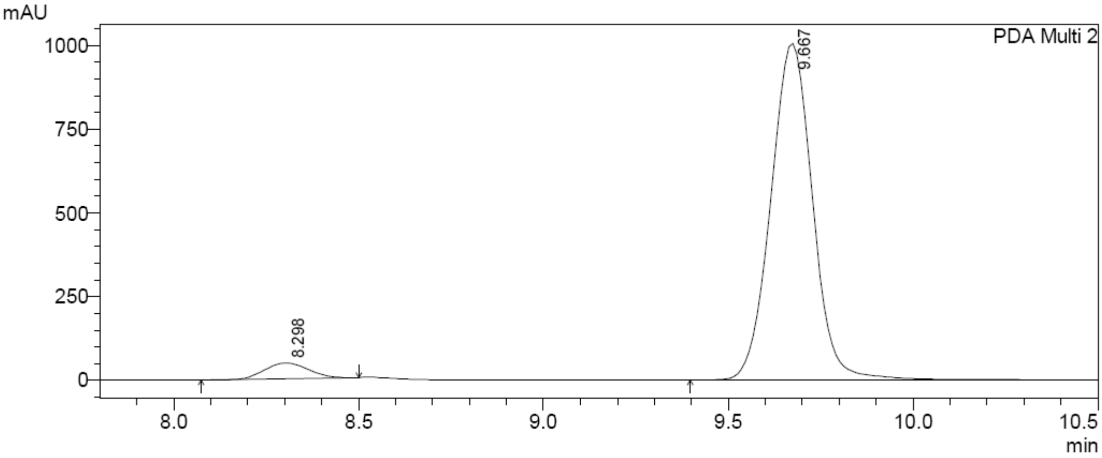

Peak#	Ret. Time	Area	Height	Area %	Height %
1	8.298	385507	47758	4.657	4.530
2	9.667	7891798	1006486	95.343	95.470
Total		8277306	1054244	100.000	100.000

Supplementary Figure 31. ^{1}H and ^{13}C NMR spectra for compound 3p

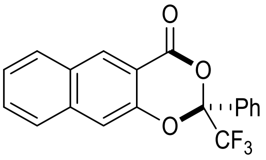

3p: 87%, 94:6 er

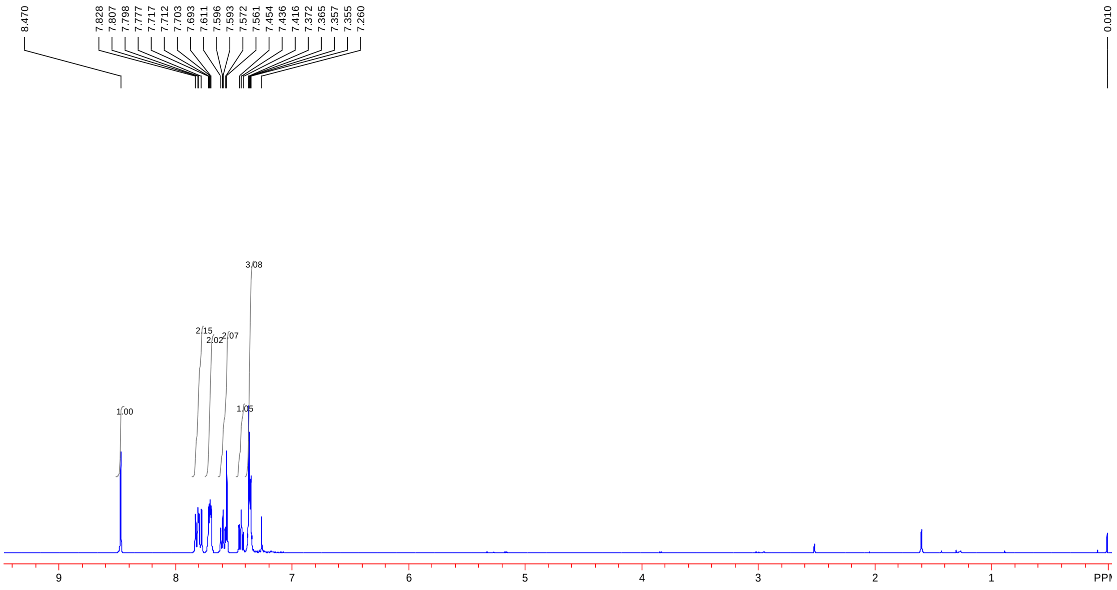

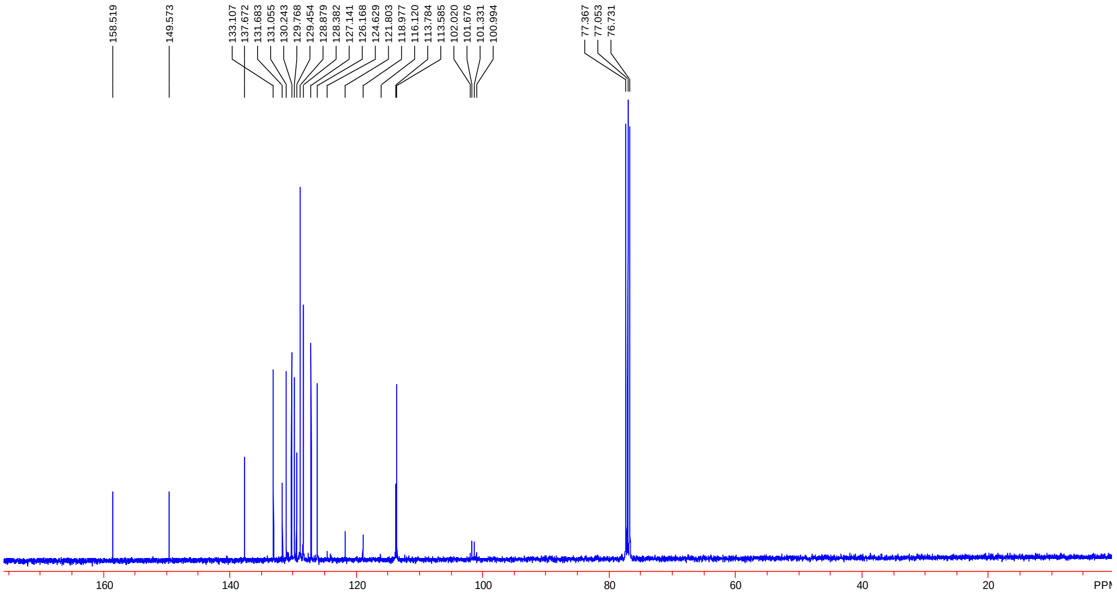

Supplementary Figure 32. ^{19}F and HPLC spectra for compound 3p

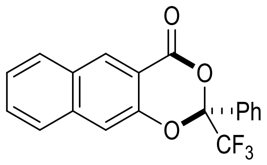

3p: 87%, 94:6 er

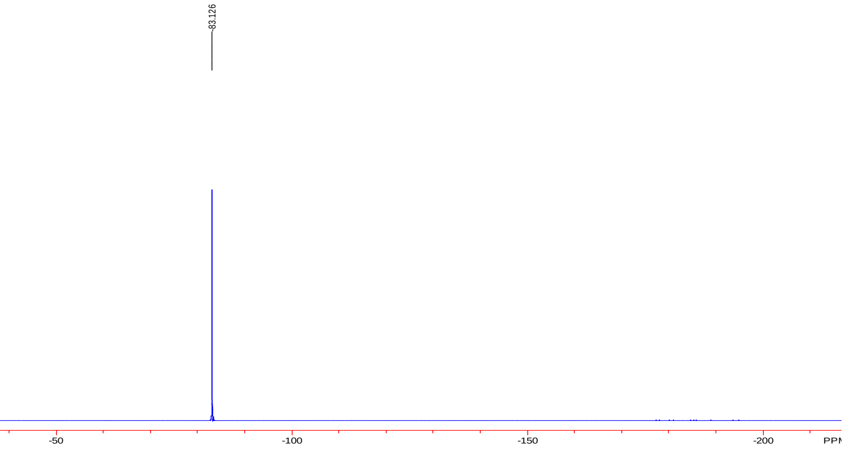

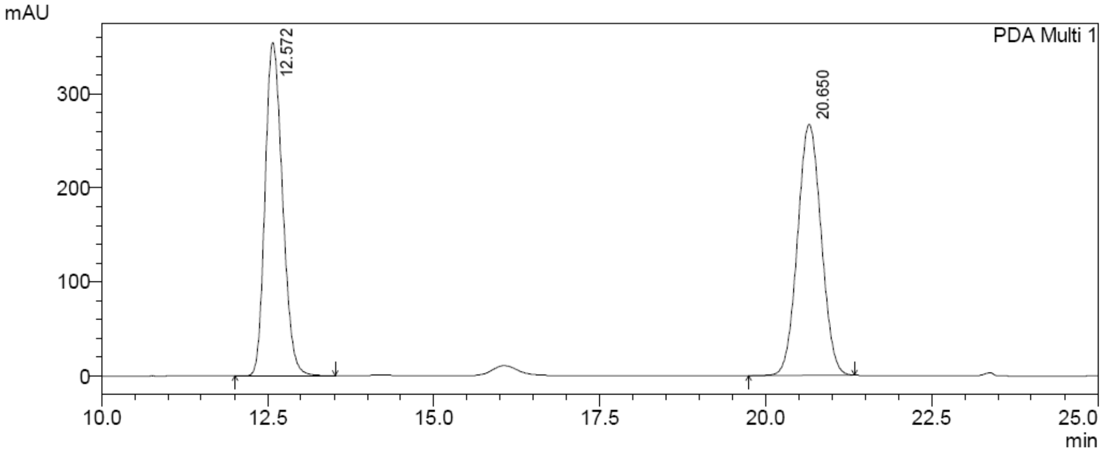

Peak#	Ret. Time	Area	Height	Area %	Height %
1	12.572	7545488	434559	49.807	57.935
2	20.650	7604104	315519	50.193	42.065
Total		15149591	750079	100.000	100.000

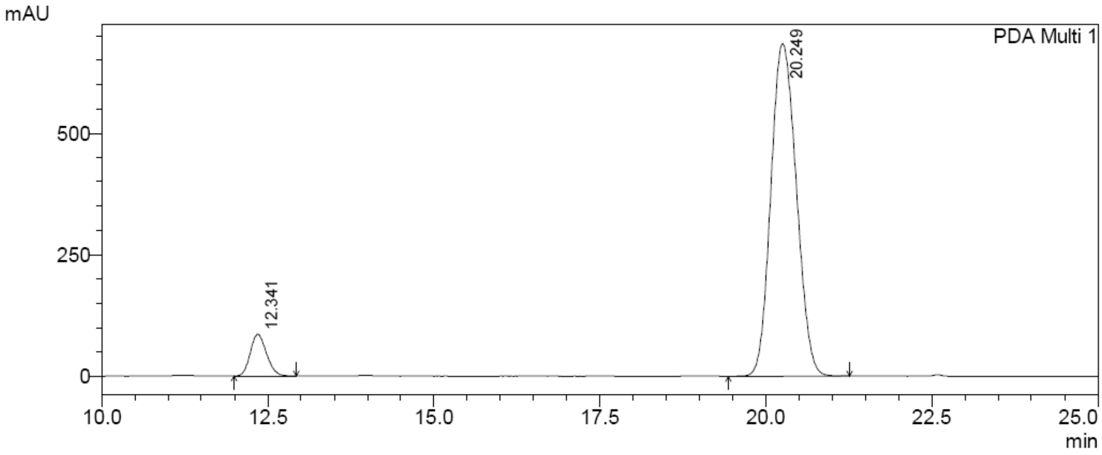

Peak#	Ret. Time	Area	Height	Area %	Height %
1	12.341	1605276	94560	6.153	8.509
2	20.248	24485430	1016698	93.847	91.491
Total		26090706	1111258	100.000	100.000

Supplementary Figure 33. ^{1}H and ^{13}C NMR spectra for compound 3q

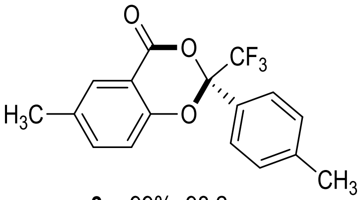

3q: 99%, 98:2 er

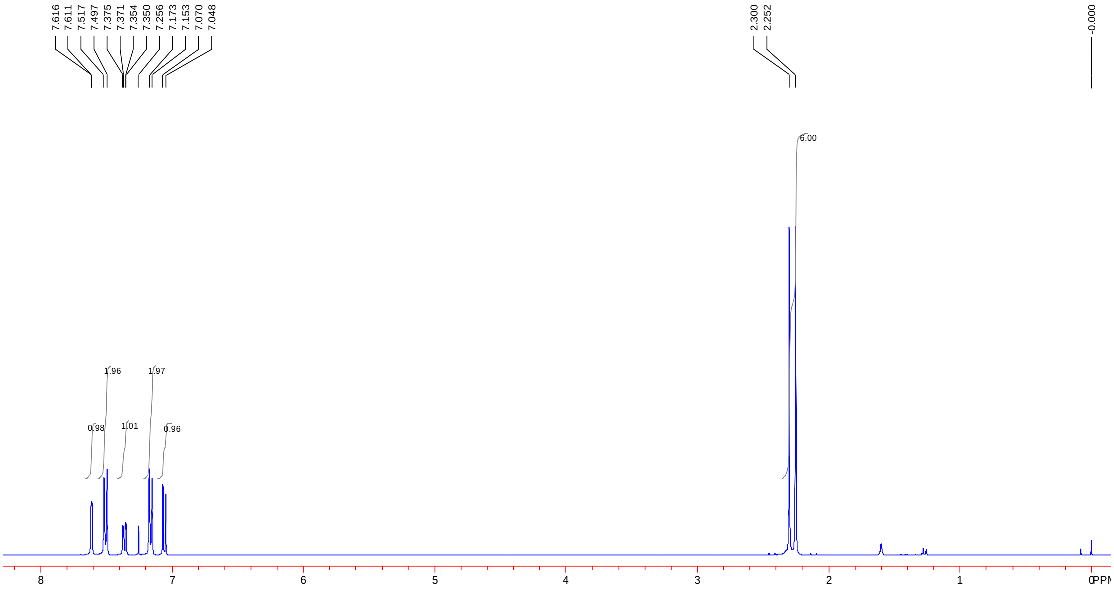

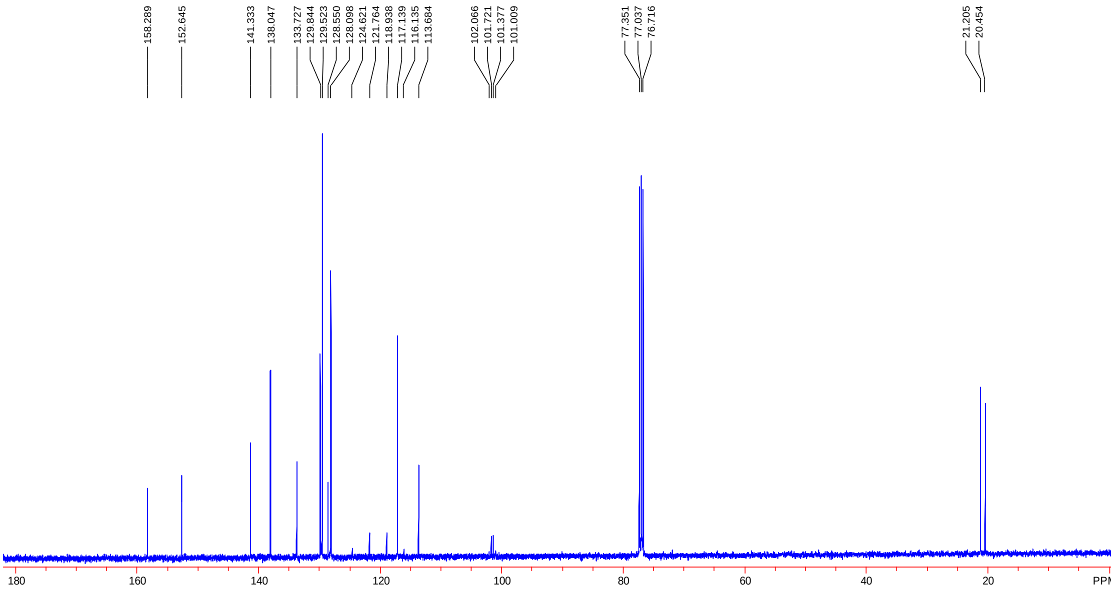

Supplementary Figure 34. ^{19}**F and HPLC spectra for compound 3q**

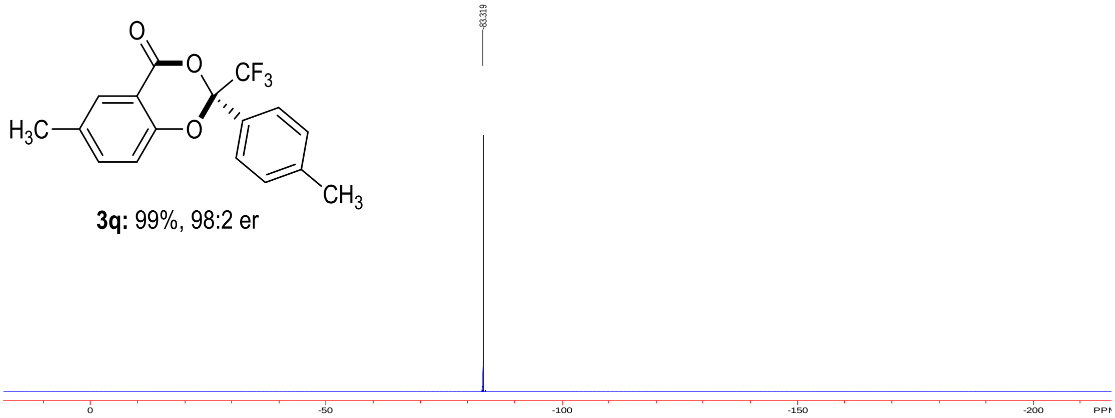

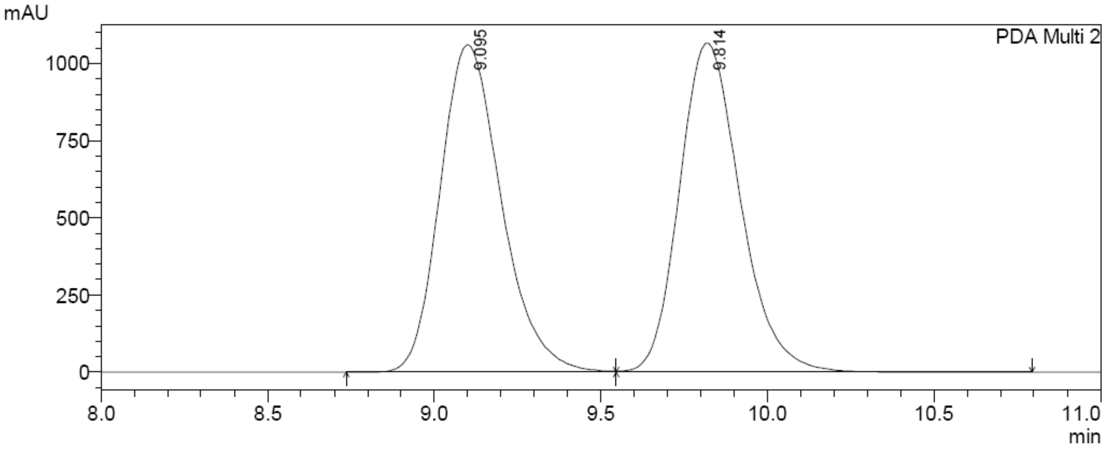

Peak#	Ret. Time	Area	Height	Area %	Height %
1	9.095	1449416	115388	50.064	49.898
2	9.814	1445687	115858	49.936	50.102
Total		2895104	231246	100.000	100.000

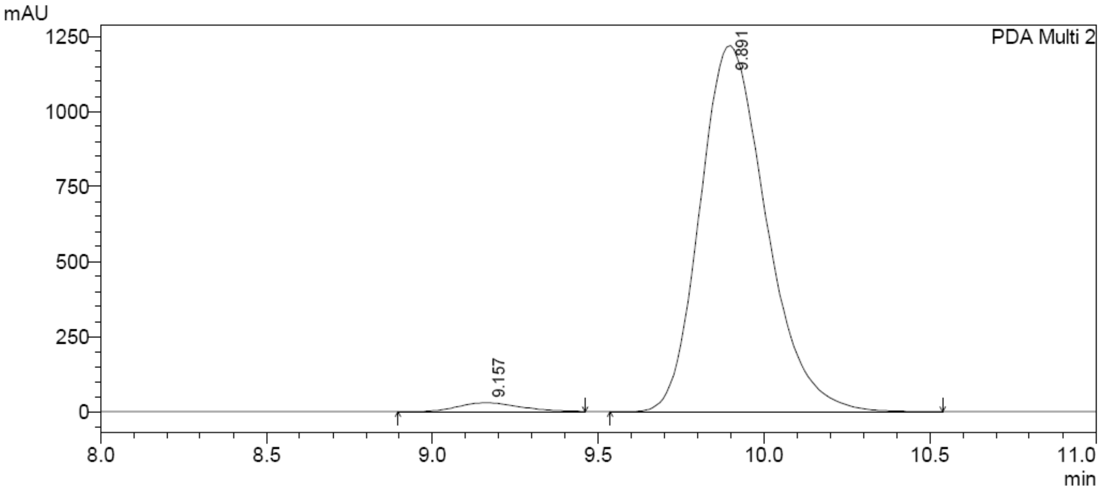

Peak#	Ret. Time	Area	Height	Area %	Height %
1	9.157	40300	3047	2.190	2.228
2	9.891	1799690	133729	97.810	97.772
Total		1839990	136777	100.000	100.000

Supplementary Figure 35. ^{1}H and ^{13}C NMR spectra for compound 3r

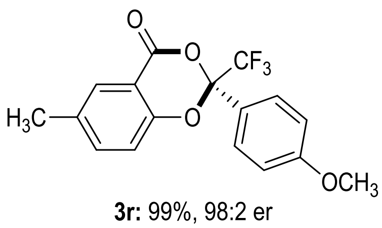

3r: 99%, 98:2 er

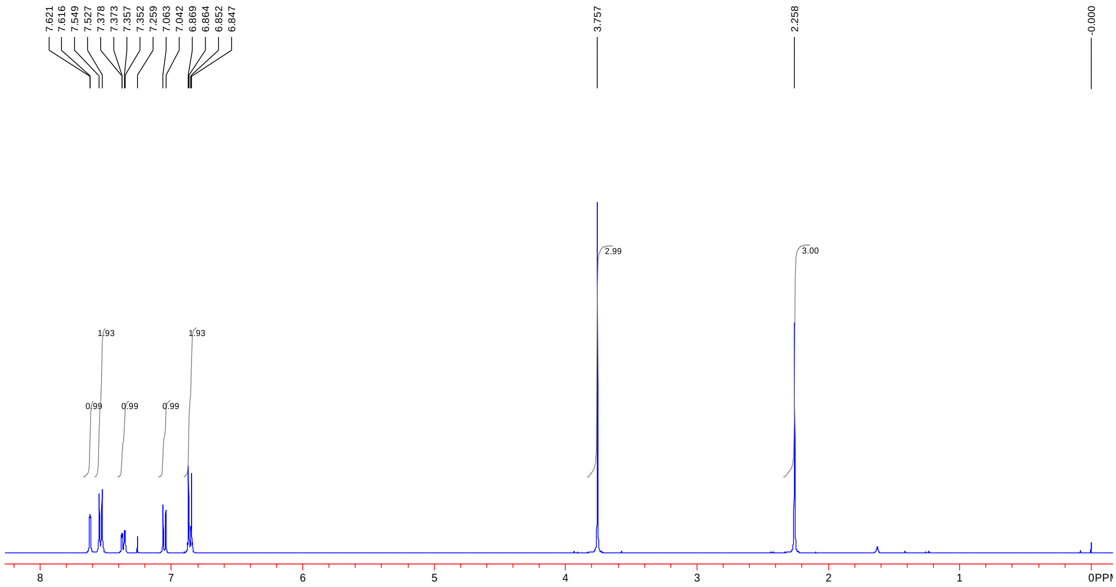

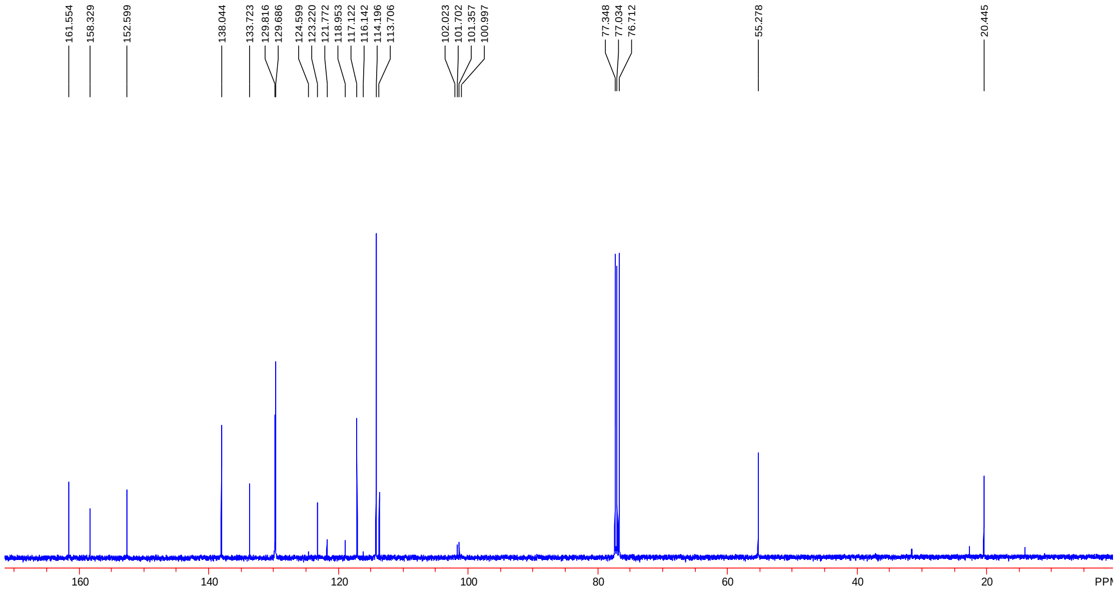

Supplementary Figure 36. ^{19}**F and HPLC spectra for compound 3r**

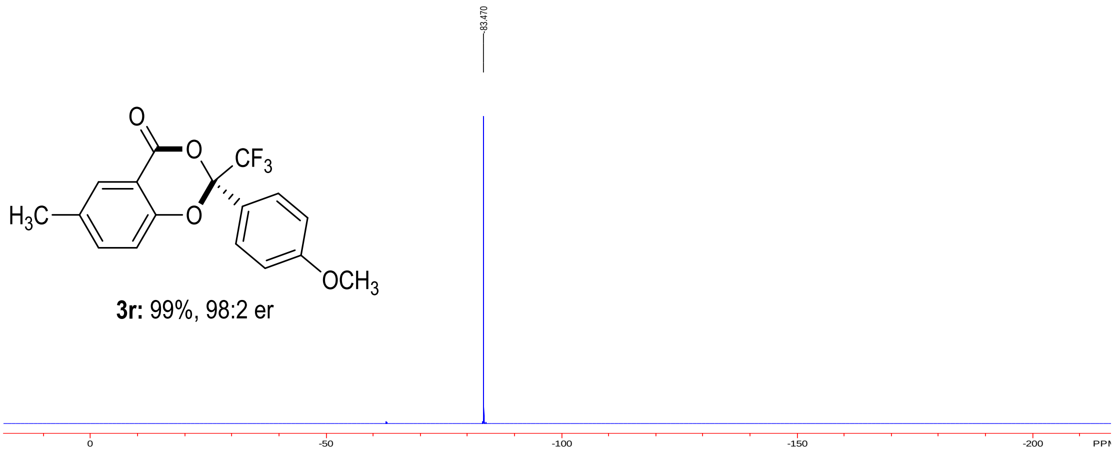

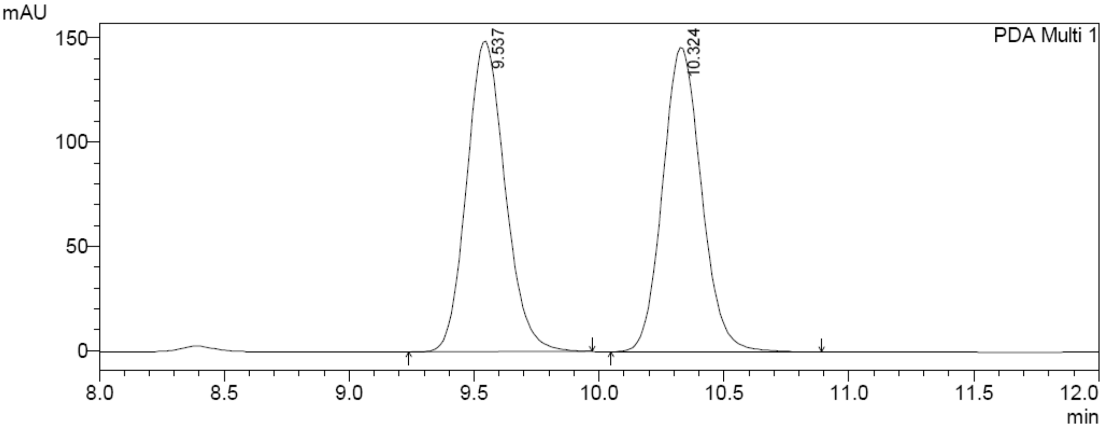

Peak#	Ret. Time	Area	Height	Area %	Height %
1	9.537	1600576	149090	50.355	50.502
2	10.324	1578031	146124	49.645	49.498
Total		3178607	295215	100.000	100.000

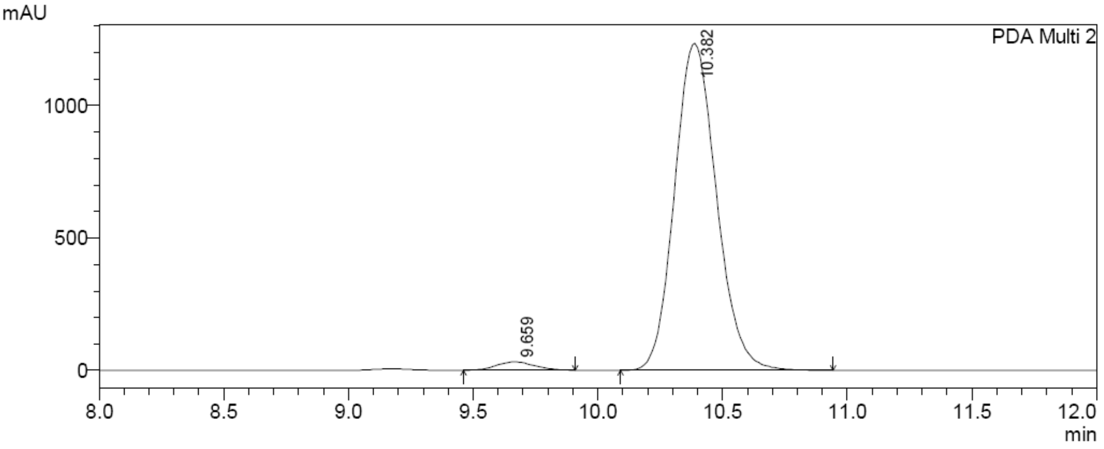

Peak#	Ret. Time	Area	Height	Area %	Height %
1	9.659	69297	6416	2.243	2.412
2	10.382	3020040	259614	97.757	97.588
Total		3089336	266030	100.000	100.000

Supplementary Figure 37. ^{1}H and ^{13}C NMR spectra for compound 3s

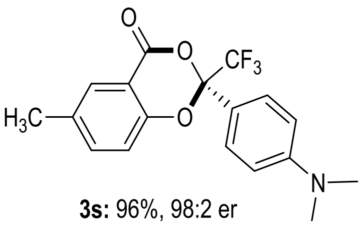

3s: 96%, 98:2 er

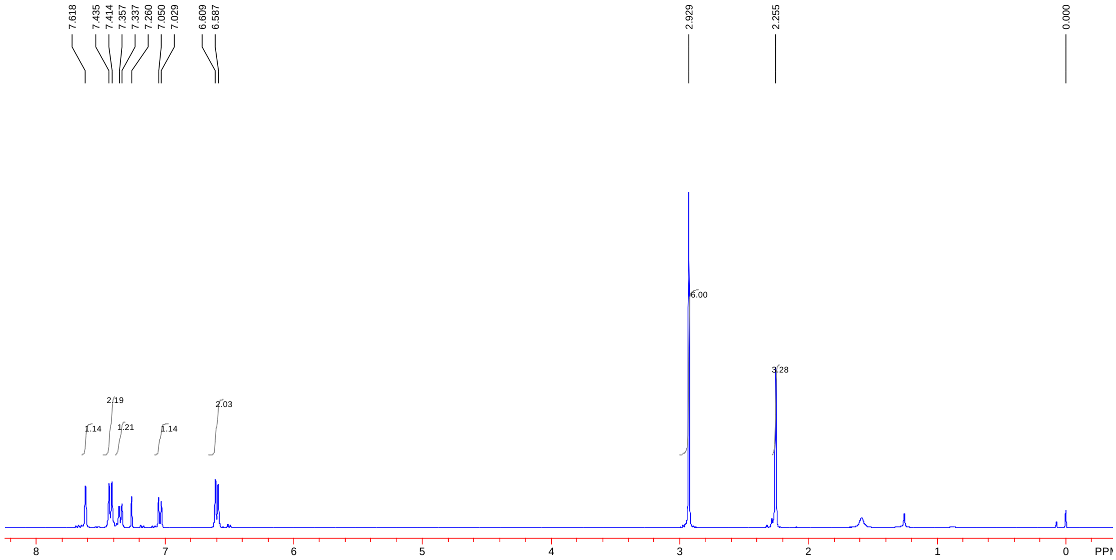

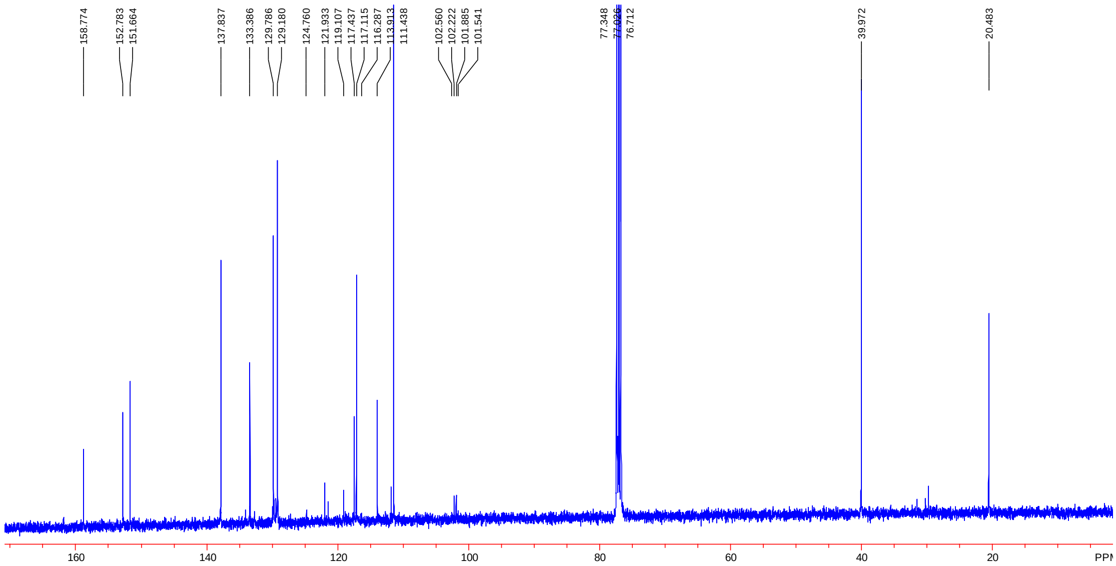

Supplementary Figure 38. ^{19}F and HPLC spectra for compound 3s

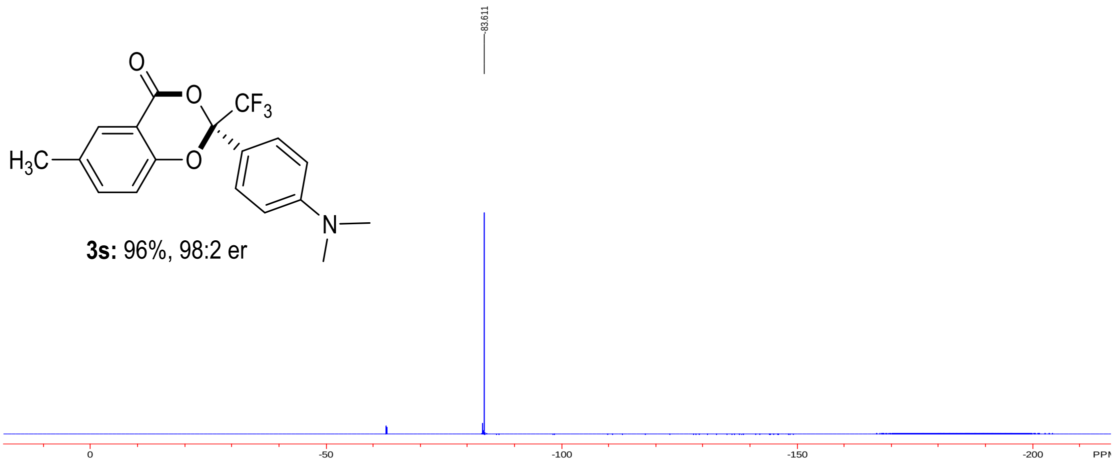

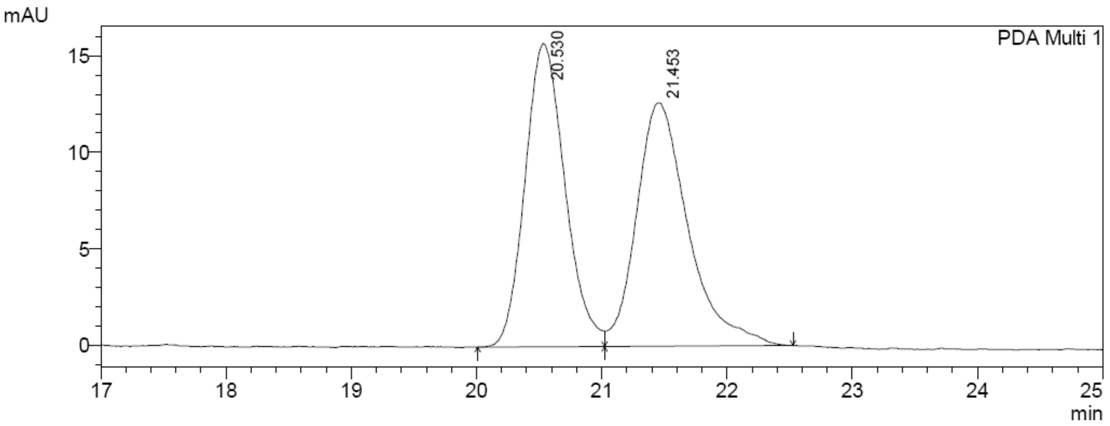

Peak#	Ret. Time	Area	Height	Area %	Height %
1	20.530	350034	15735	49.128	55.478
2	21.453	362453	12628	50.872	44.522
Total		712487	28363	100.000	100.000

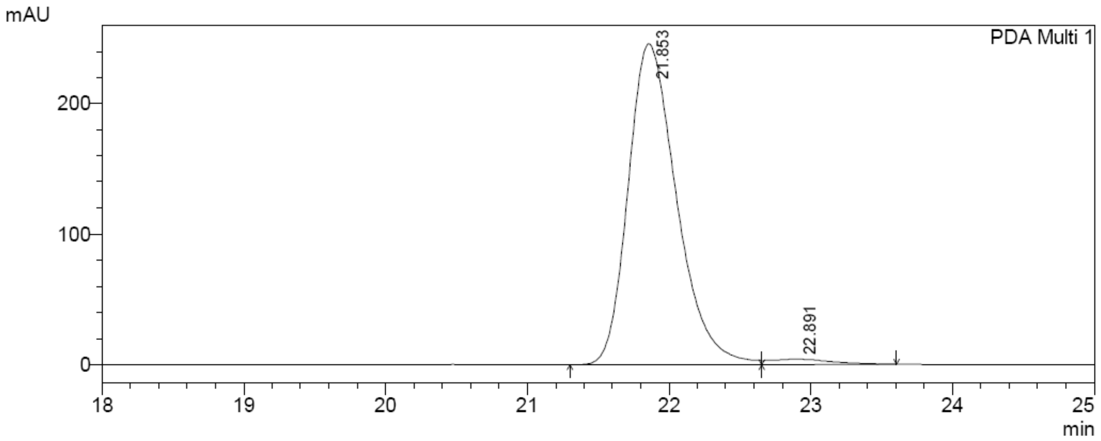

Peak#	Ret. Time	Area	Height	Area %	Height %
1	21.853	7050651	293256	97.947	98.337
2	22.886	147766	4959	2.053	1.663
Total		7198417	298214	100.000	100.000

Supplementary Figure 39. ^{1}H and ^{13}C NMR spectra for compound 3t

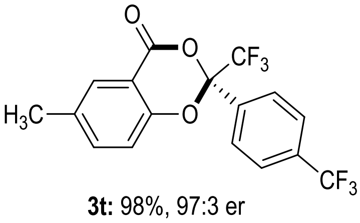

3t: 98%, 97:3 er

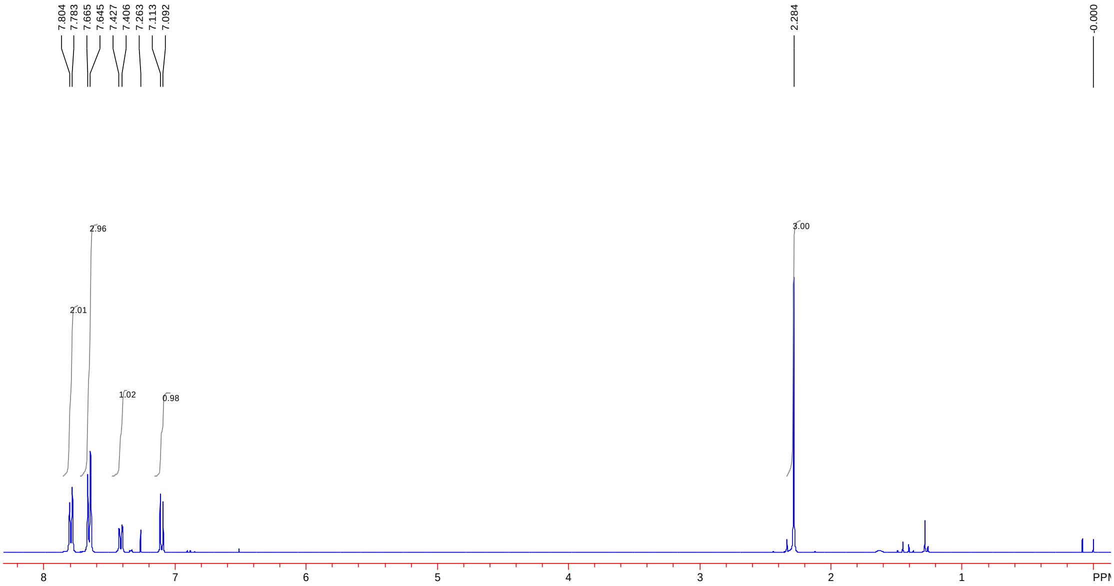

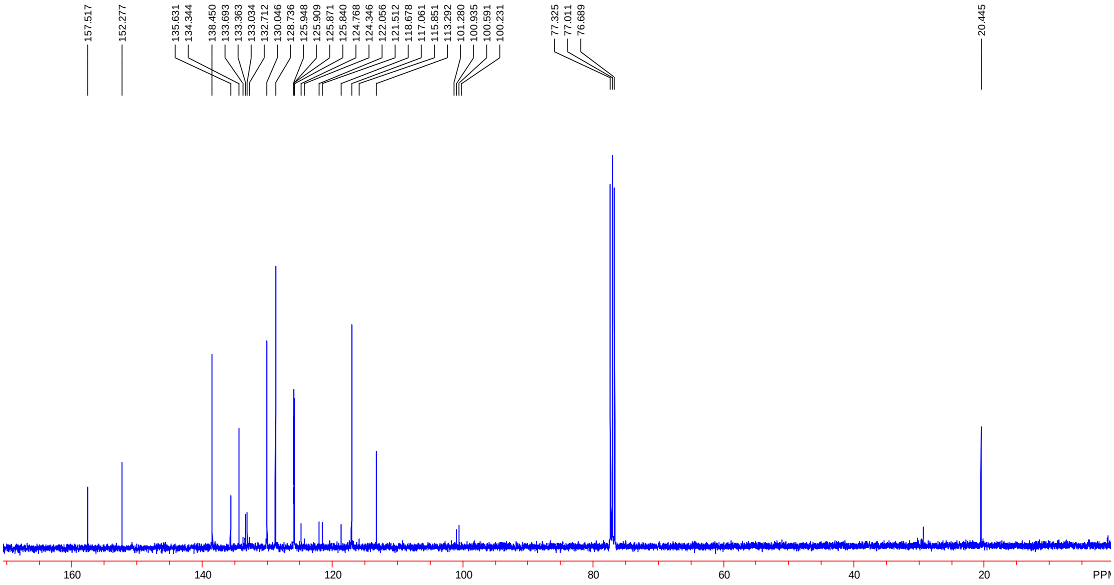

Supplementary Figure 40. **^{19}F and HPLC spectra for compound 3t**

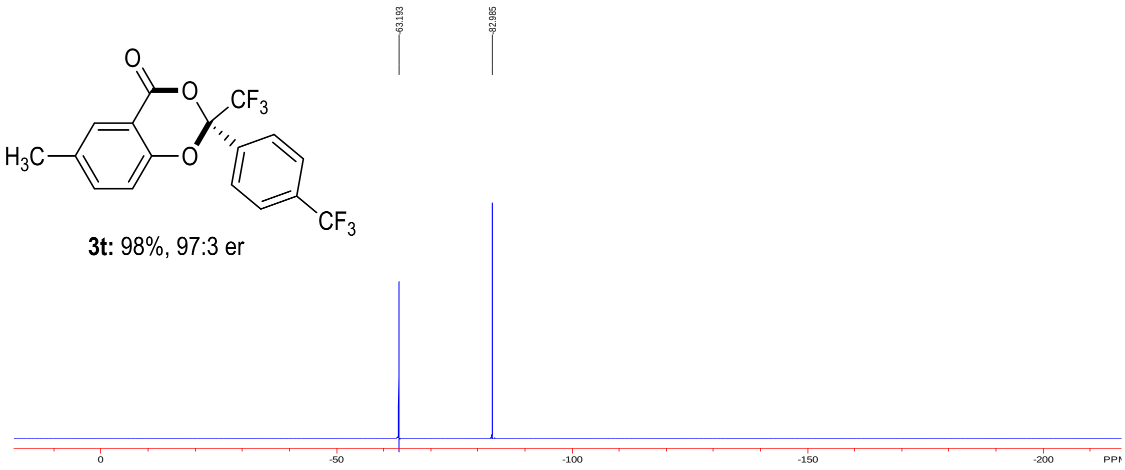

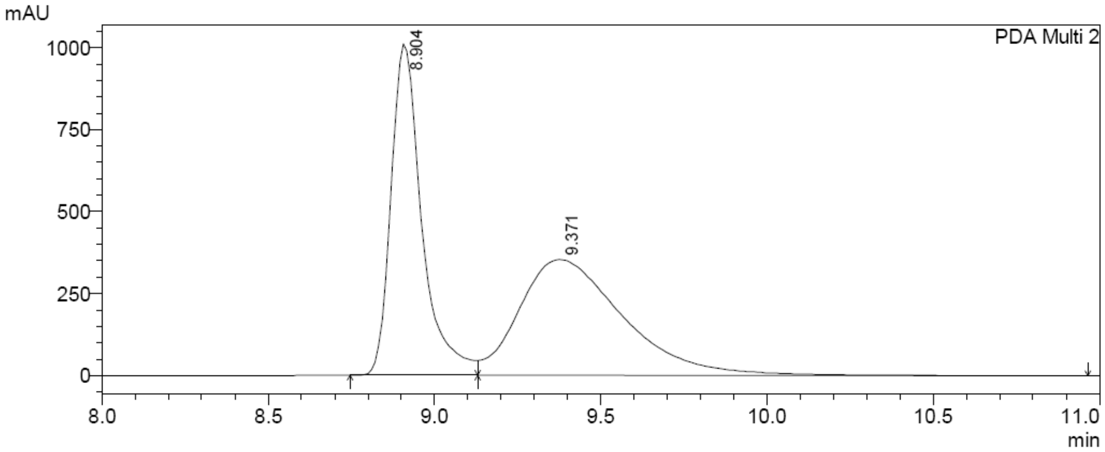

Peak#	Ret. Time	Area	Height	Area %	Height %
1	8.903	916734	147936	48.402	75.688
2	9.372	977273	47518	51.598	24.312
Total		1894007	195454	100.000	100.000

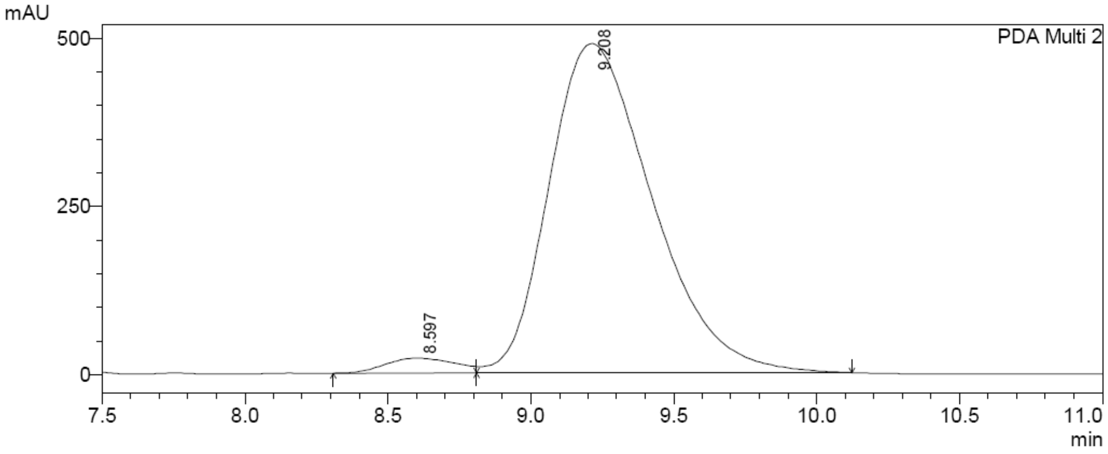

Peak#	Ret. Time	Area	Height	Area %	Height %
1	8.600	50004	2910	2.894	4.107
2	9.206	1677667	67935	97.106	95.893
Total		1727671	70844	100.000	100.000

Supplementary Figure 41. ^{1}H and ^{13}C NMR spectra for compound 3u

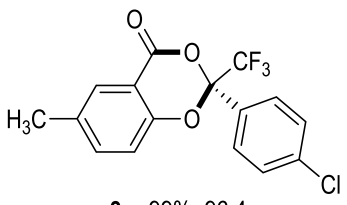

3u: 99%, 96:4 er

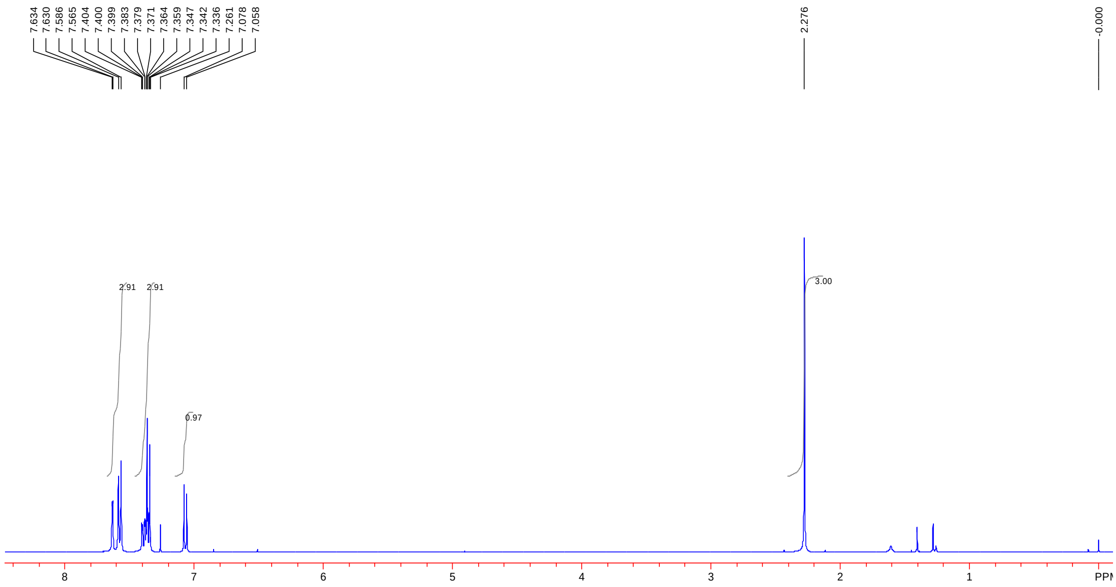

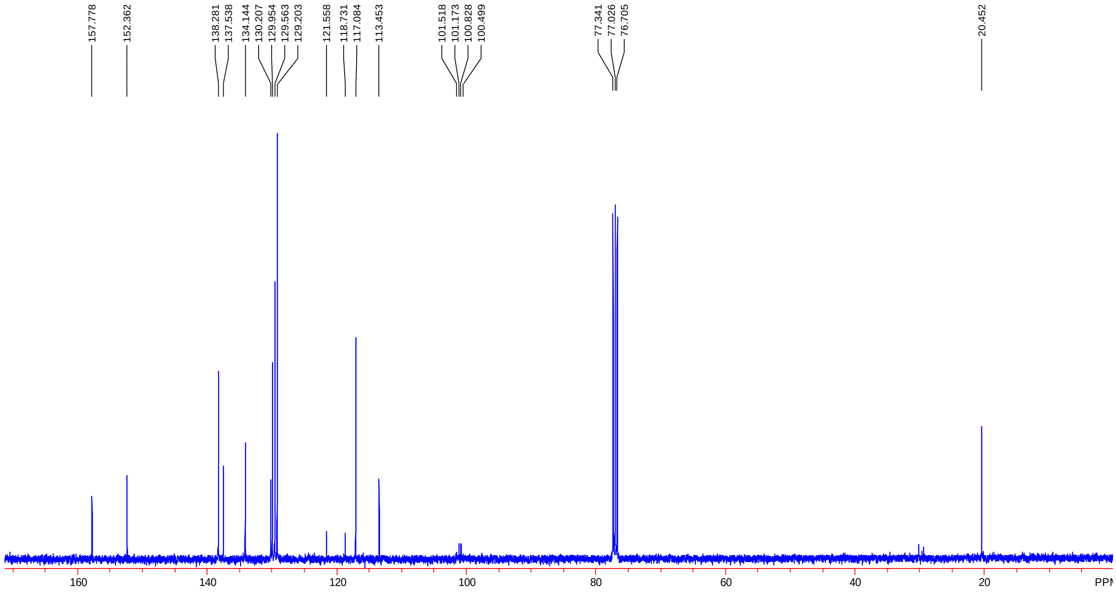

Supplementary Figure 42. ^{19}F and HPLC spectra for compound 3u

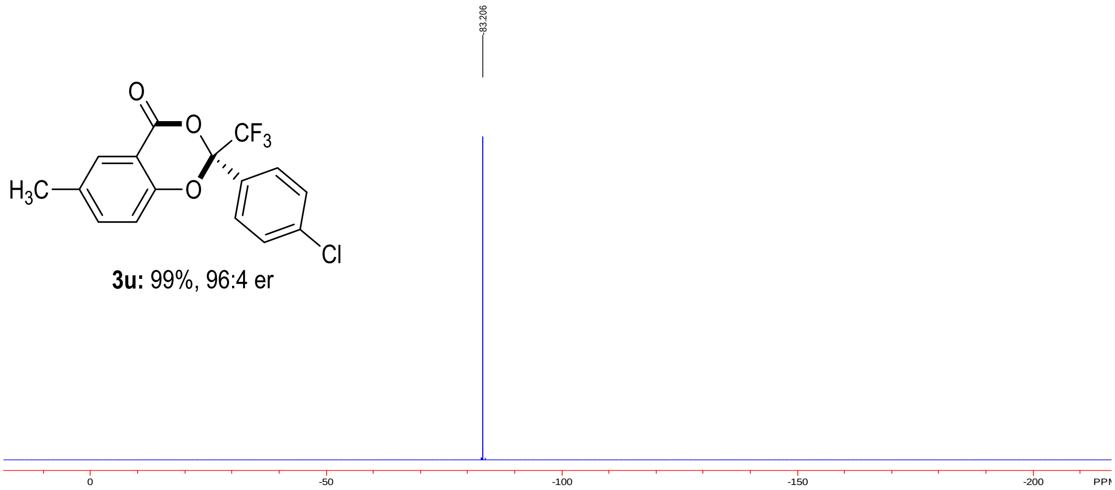

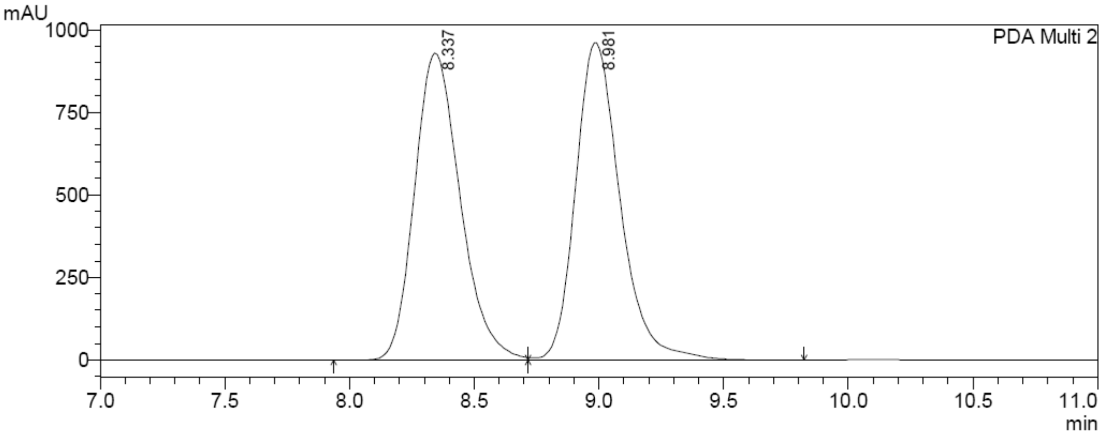

Peak#	Ret. Time	Area	Height	Area %	Height %
1	8.337	1147920	89624	49.922	49.200
2	8.981	1151505	92540	50.078	50.800
Total		2299425	182165	100.000	100.000

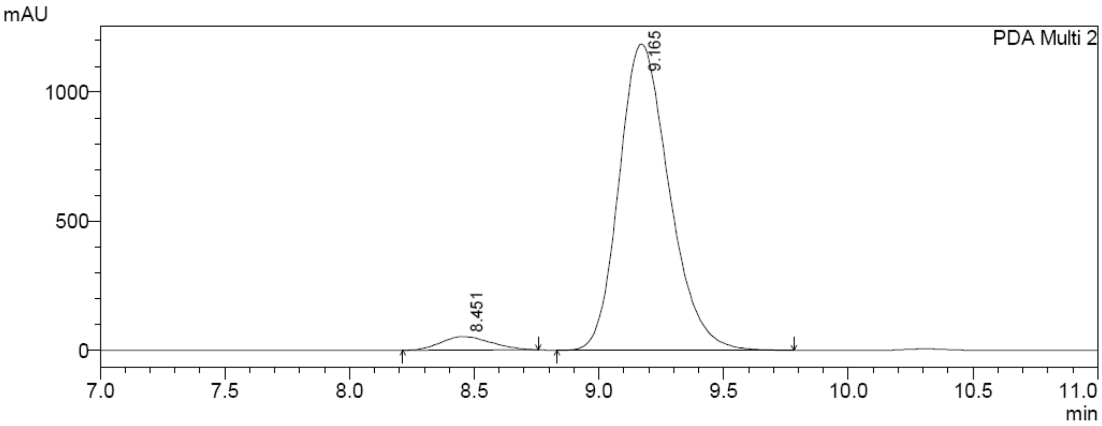

Peak#	Ret. Time	Area	Height	Area %	Height %
1	8.451	732758	51877	4.237	4.188
2	9.165	16563059	1186888	95.763	95.812
Total		17295817	1238765	100.000	100.000

Supplementary Figure 43. ^{1}H and ^{13}C NMR spectra for compound 3v

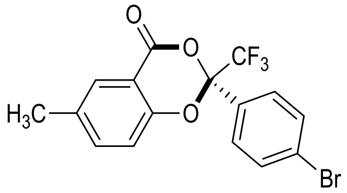

3v: 99%, 95:5 er

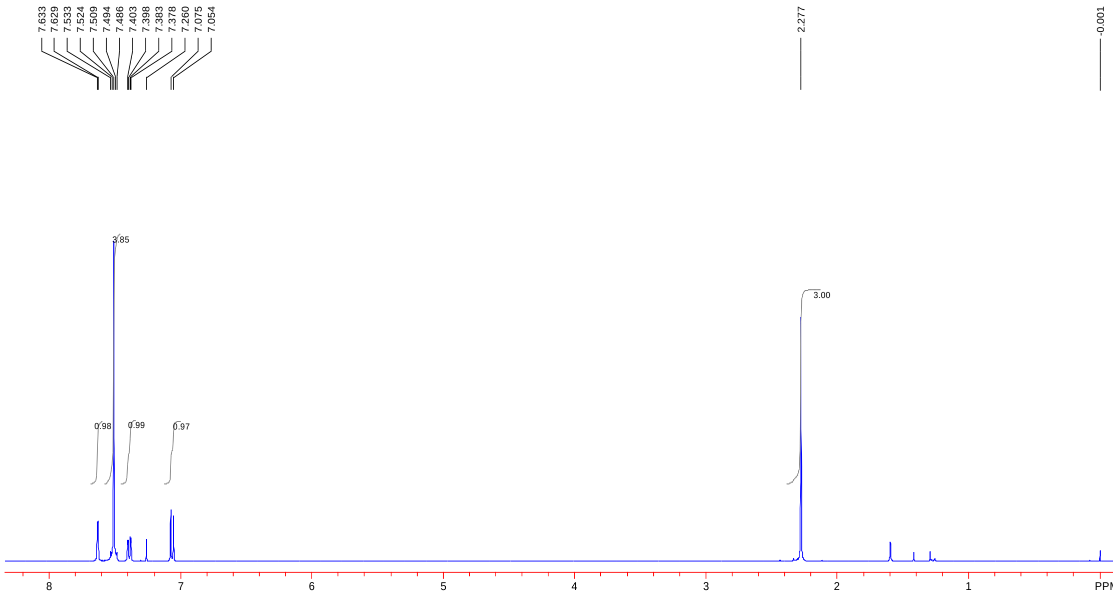

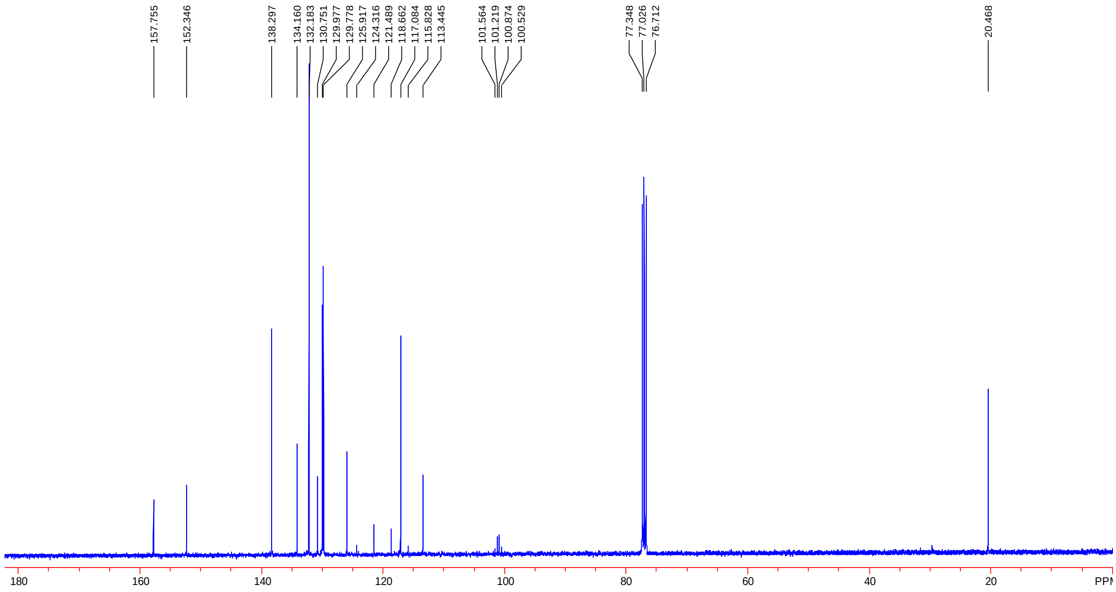

Supplementary Figure 44. ^{19}F and HPLC spectra for compound 3v

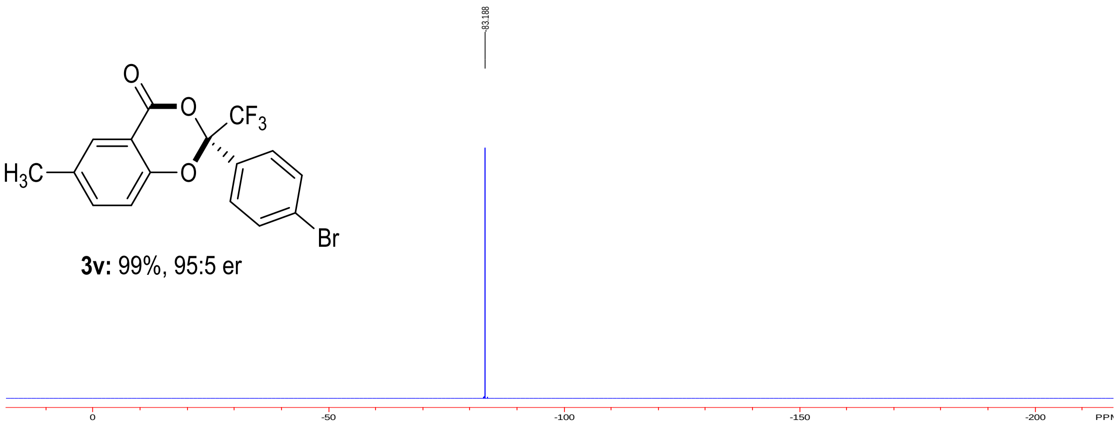

3v: 99%, 95:5 er

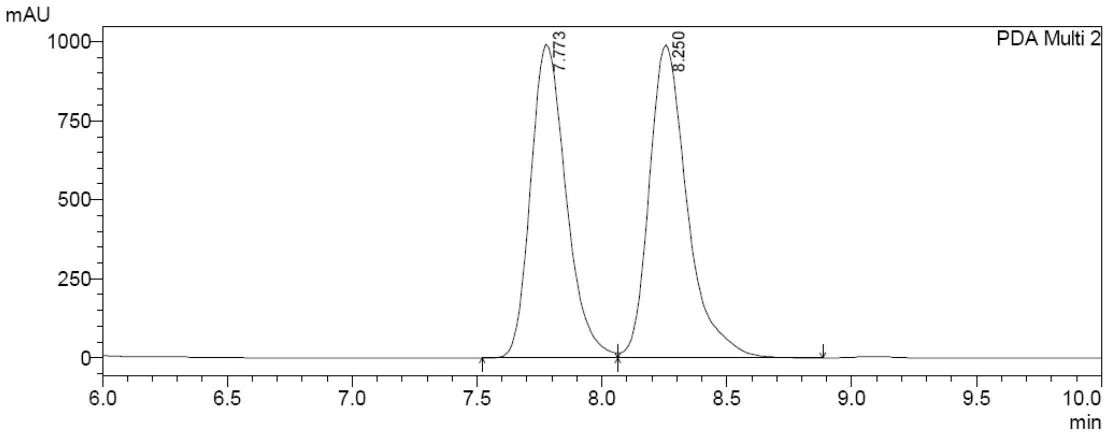

Peak#	Ret. Time	Area	Height	Area %	Height %
1	7.773	9772530	991165	48.296	50.053
2	8.250	10462085	989052	51.704	49.947
Total		20234615	1980217	100.000	100.000

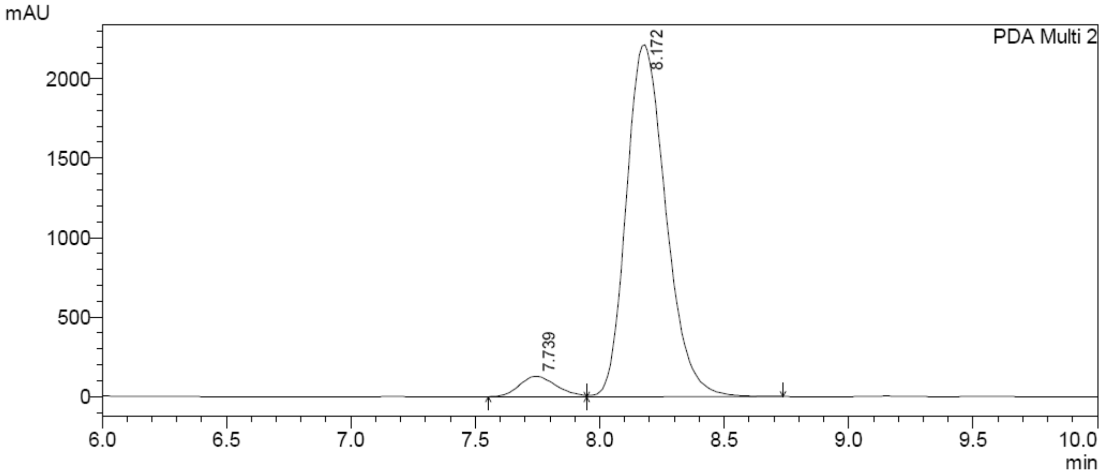

Peak#	Ret. Time	Area	Height	Area %	Height %
1	7.739	1321161	128096	5.119	5.472
2	8.172	24488346	2212766	94.881	94.528
Total		25809507	2340862	100.000	100.000

Supplementary Figure 45. ^{1}H and ^{13}C NMR spectra for compound 3w

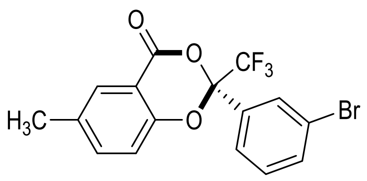

3w: 96%, 93:7 er

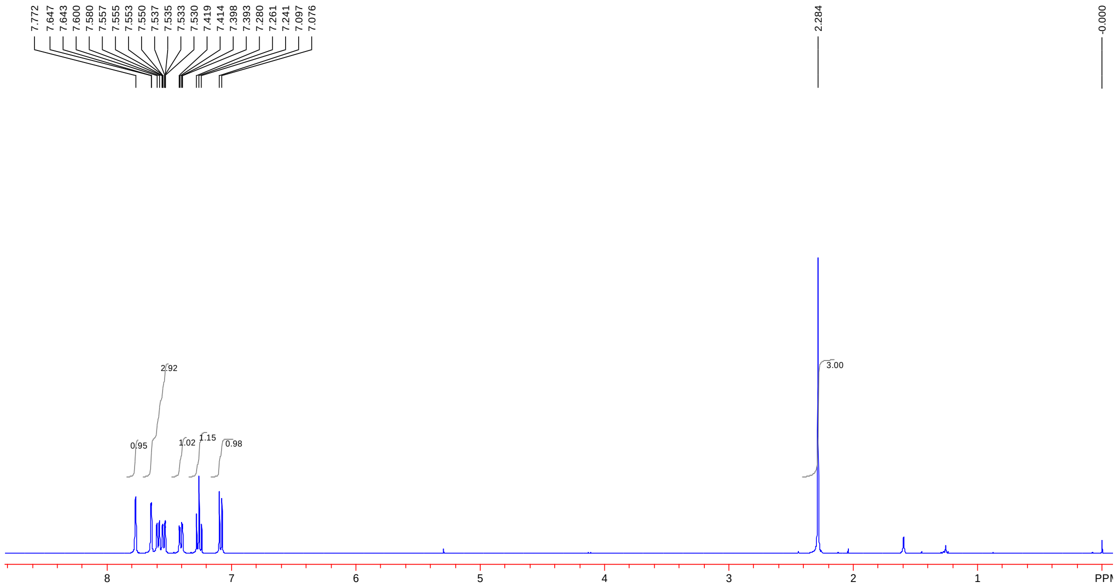

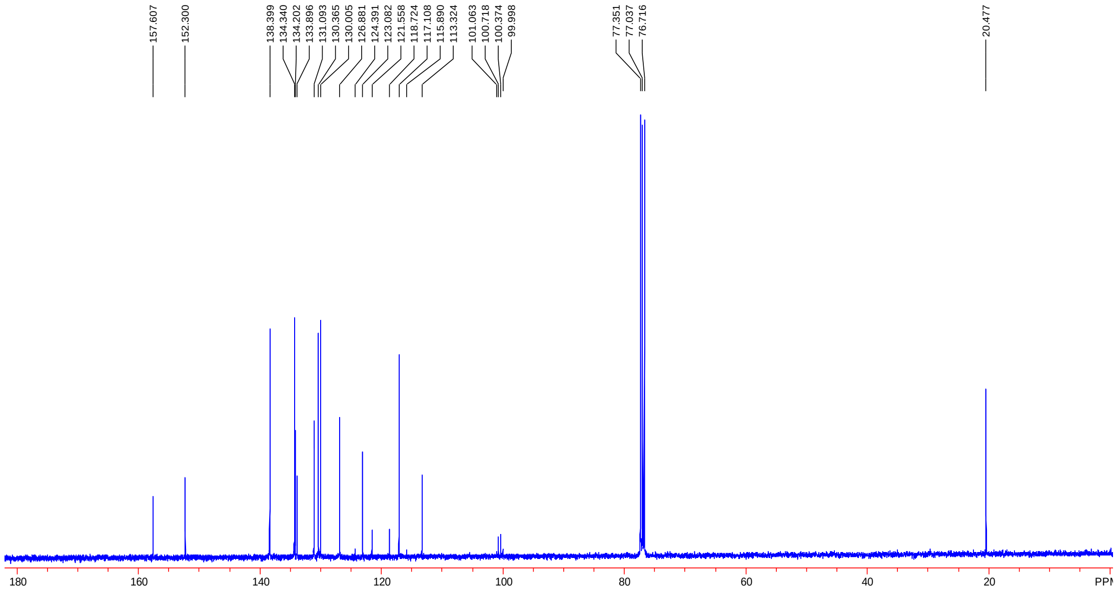

Supplementary Figure 46. ^{19}F and HPLC spectra for compound 3w

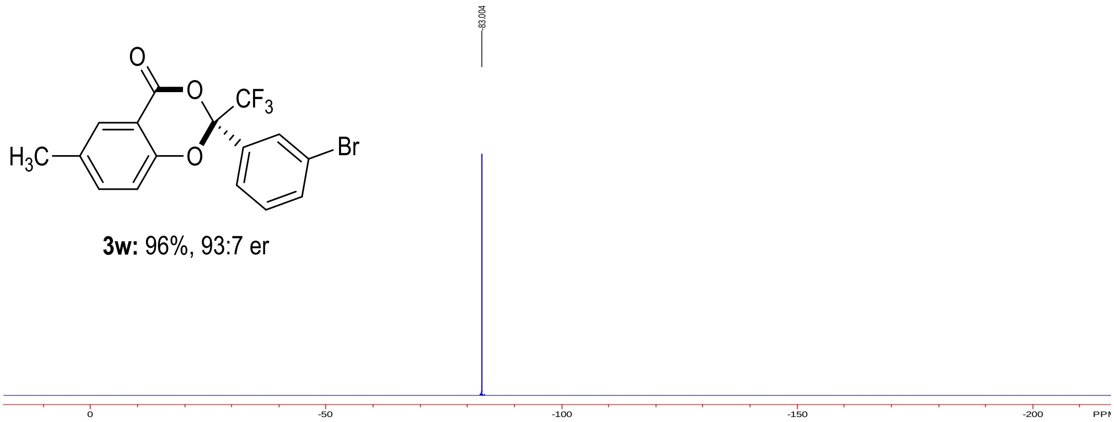

3w: 96%, 93:7 er

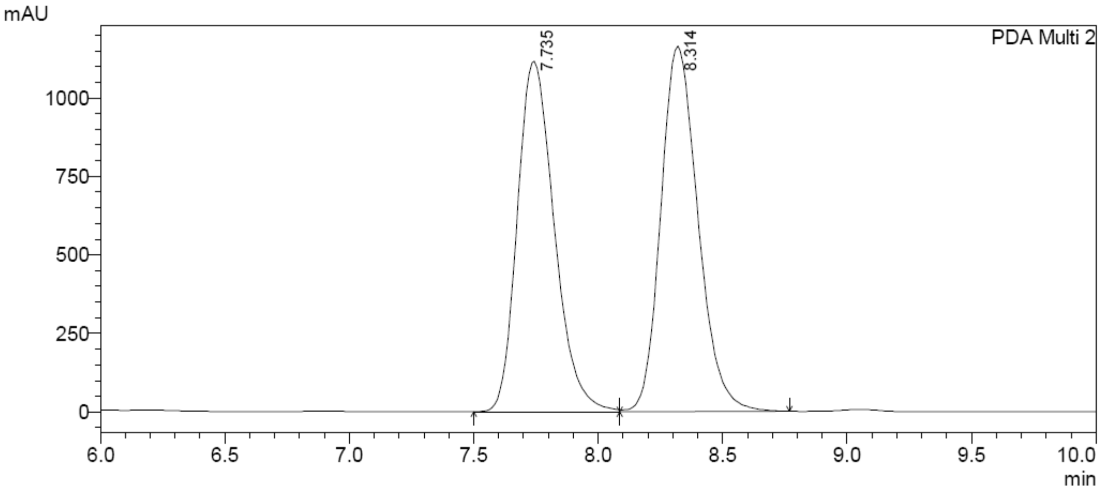

Peak#	Ret. Time	Area	Height	Area %	Height %
1	7.735	1015905	100266	48.483	48.746
2	8.314	1079487	105423	51.517	51.254
Total		2095393	205689	100.000	100.000

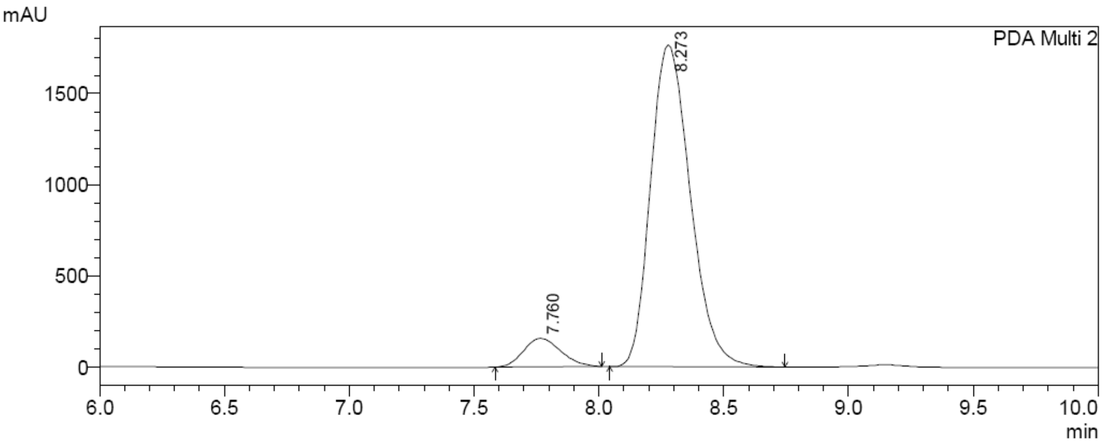

Peak#	Ret. Time	Area	Height	Area %	Height %
1	7.759	142123	13738	7.394	7.657
2	8.273	1780121	165683	92.606	92.343
Total		1922243	179422	100.000	100.000

Supplementary Figure 47. ^{1}H and ^{13}C NMR spectra for compound 3x

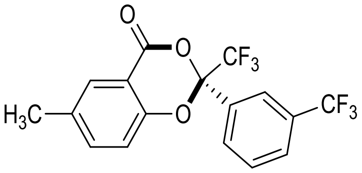

3x: 94%, 96:4 er

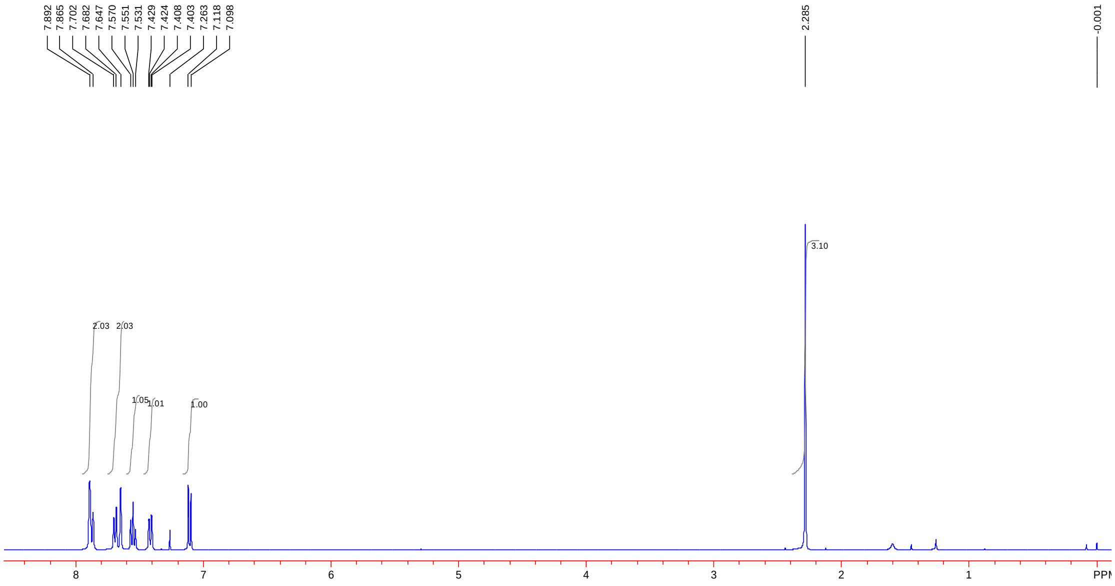

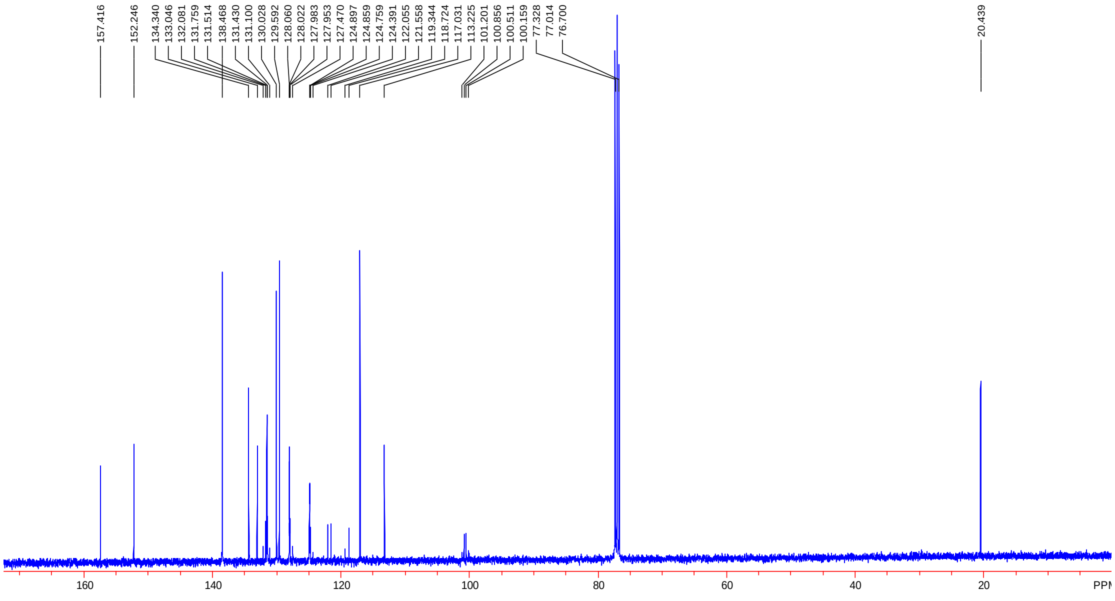

Supplementary Figure 48. ^{19}F and HPLC spectra for compound 3x

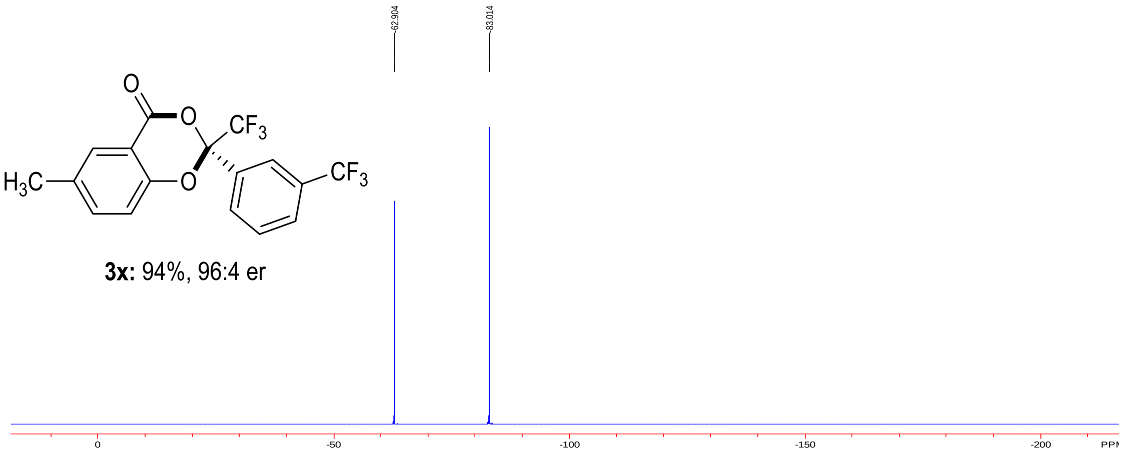

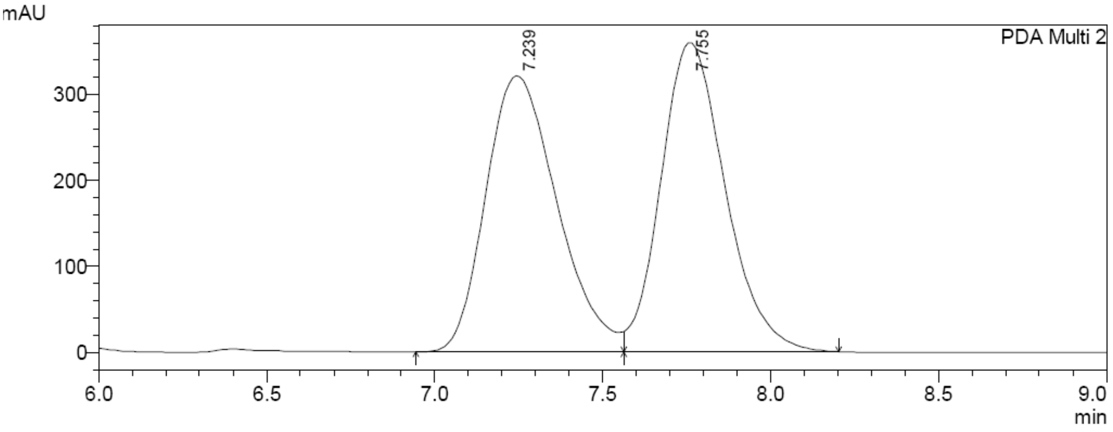

Peak#	Ret. Time	Area	Height	Area %	Height %
1	7.239	4759559	321444	49.843	47.208
2	7.755	4789523	359470	50.157	52.792
Total		9549083	680914	100.000	100.000

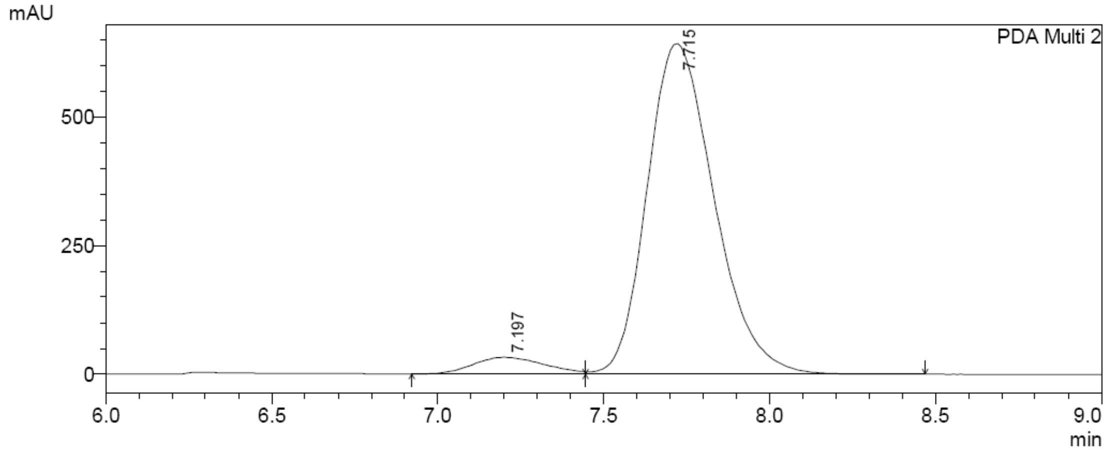

Peak#	Ret. Time	Area	Height	Area %	Height %
1	7.196	49353	3629	4.252	4.293
2	7.715	1111420	80913	95.748	95.707
Total		1160773	84542	100.000	100.000

Supplementary Figure 49. ^{1}H and ^{13}C NMR spectra for compound 3y

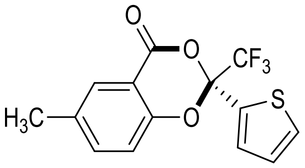

3y: 75%y, 89:11 er

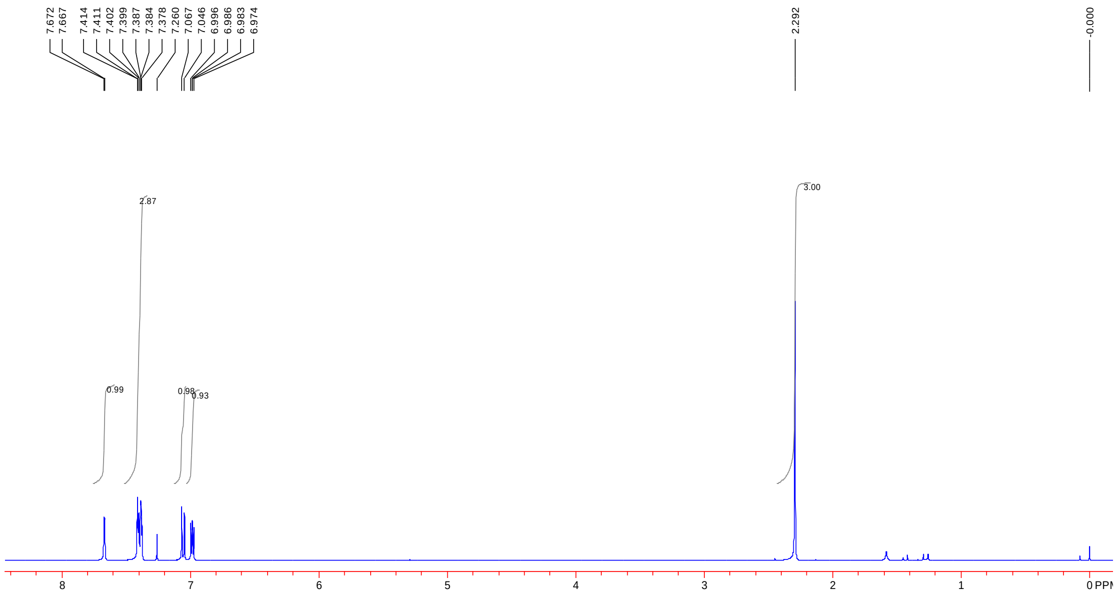

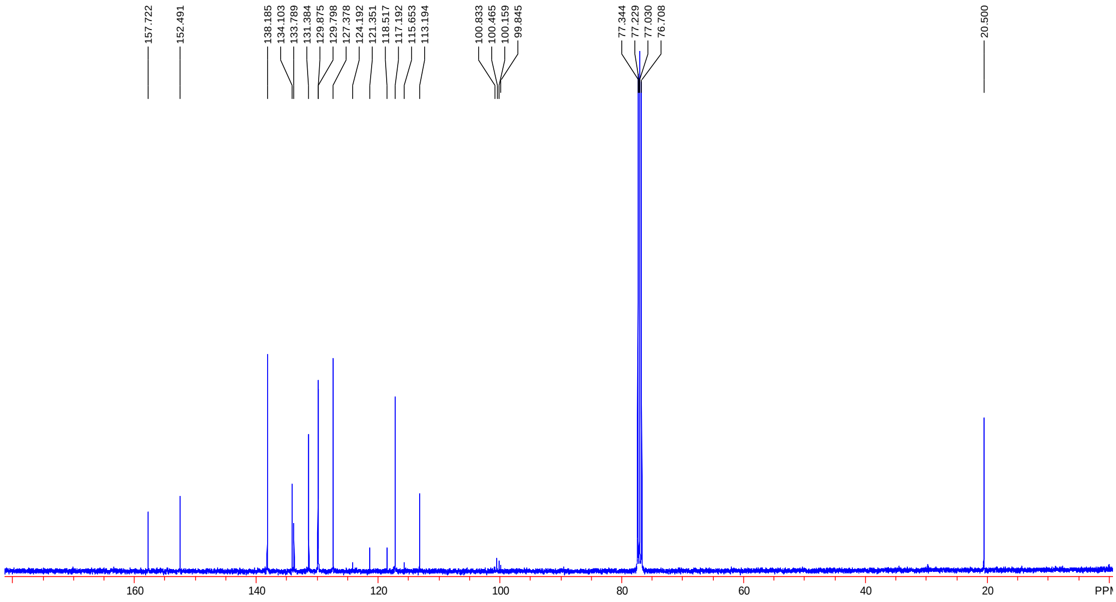

Supplementary Figure 50. ^{19}F and HPLC spectra for compound 3y

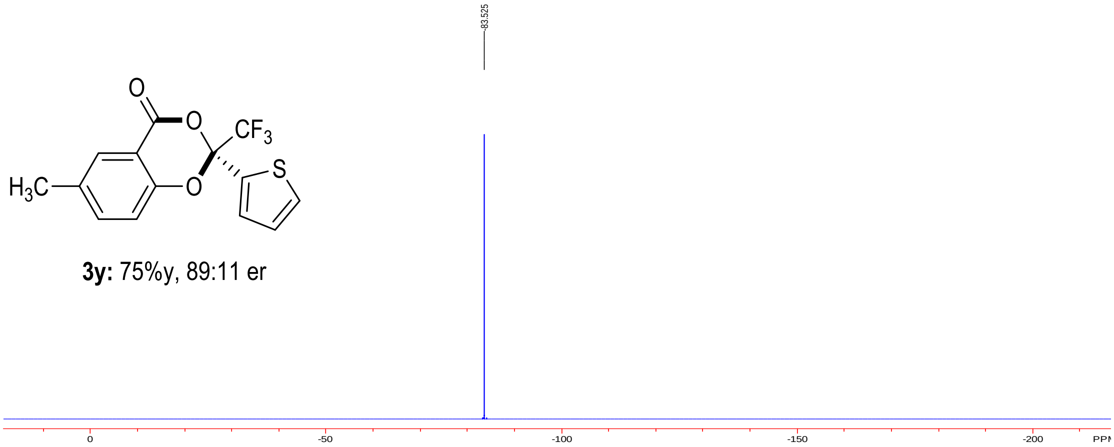

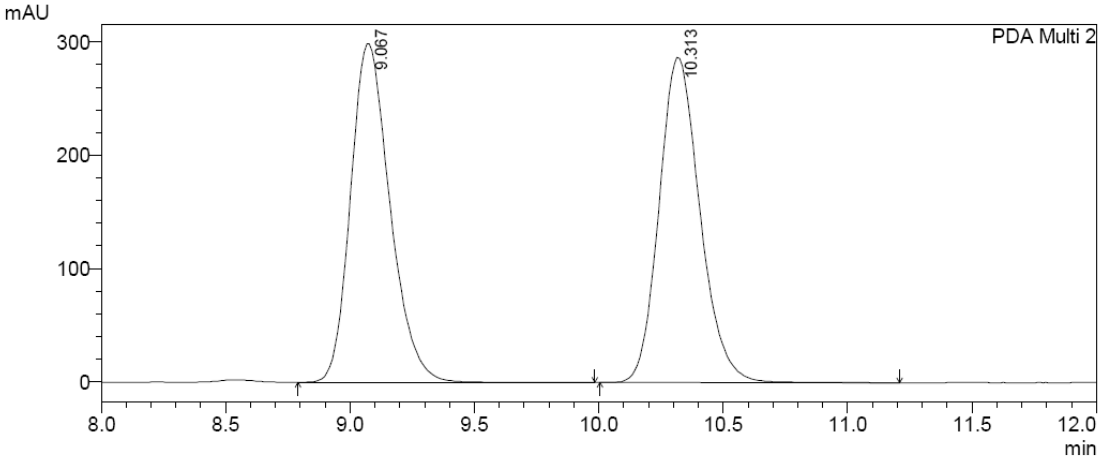

Peak#	Ret. Time	Area	Height	Area %	Height %
1	9.067	3296844	298685	50.073	50.997
2	10.313	3287237	287008	49.927	49.003
Total		6584082	585693	100.000	100.000

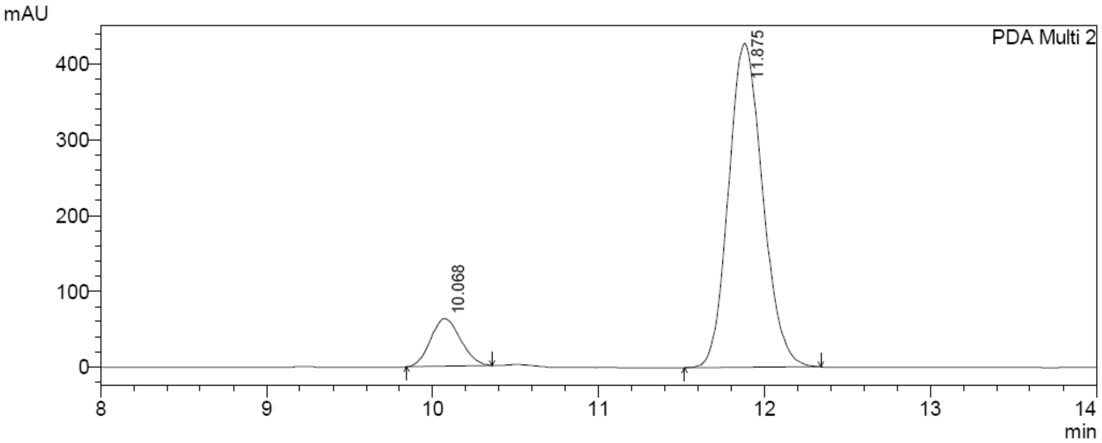

Peak#	Ret. Time	Area	Height	Area %	Height %
1	10.068	790860	62761	11.440	12.806
2	11.875	6122103	427331	88.560	87.194
Total		6912962	490092	100.000	100.000

Supplementary Figure 51. ^{1}H and ^{13}C NMR spectra for compound 3z

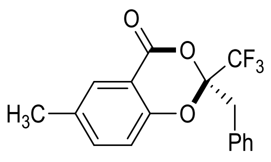

3z: 64%, 90:10 er

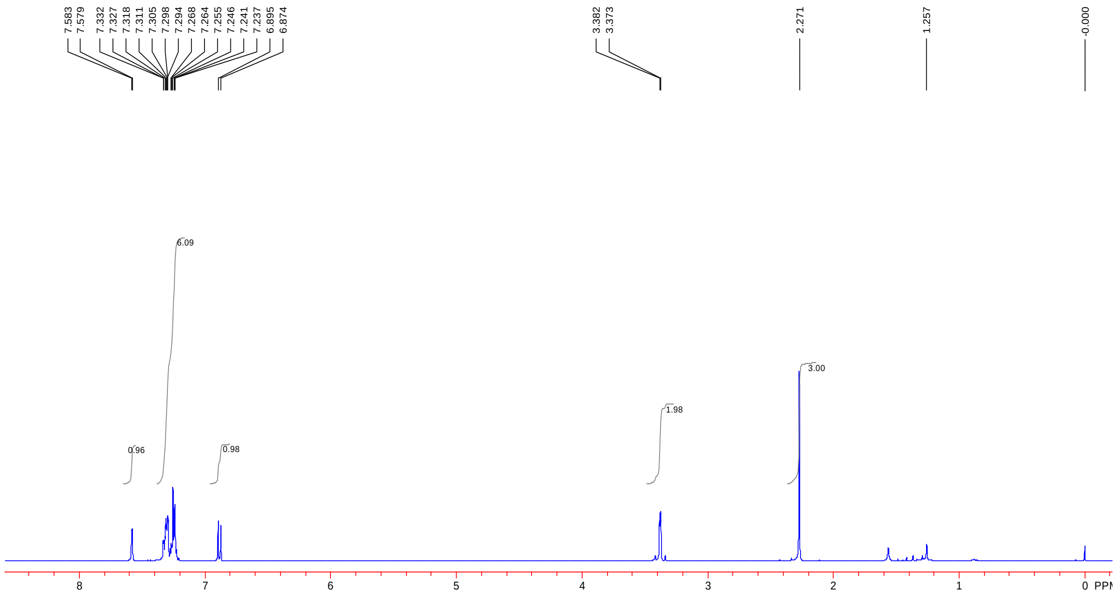

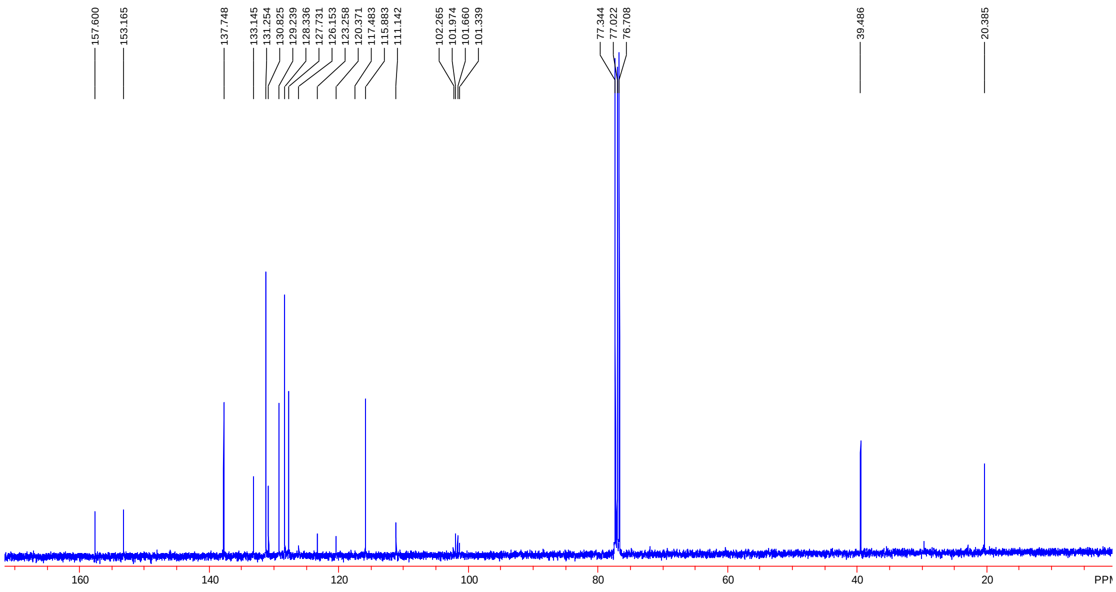

Supplementary Figure 52. ^{19}F and HPLC spectra for compound 3z

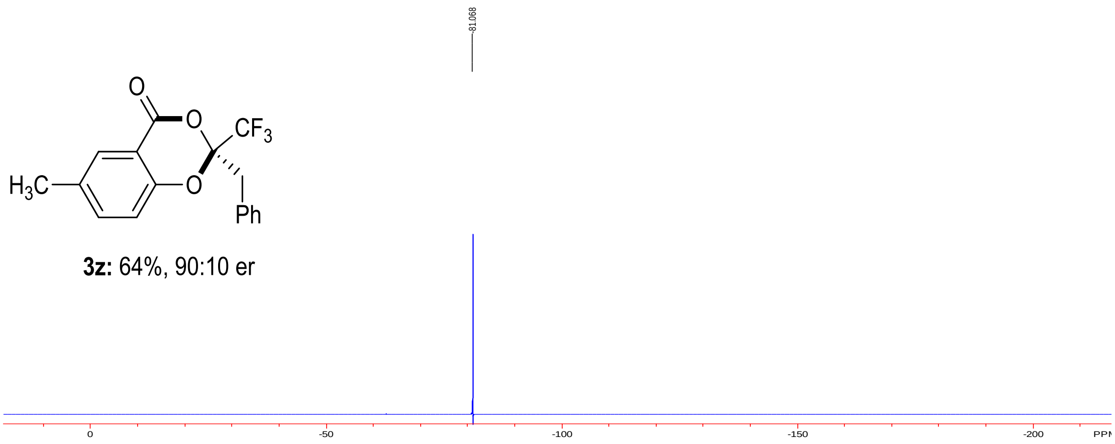

3z: 64%, 90:10 er

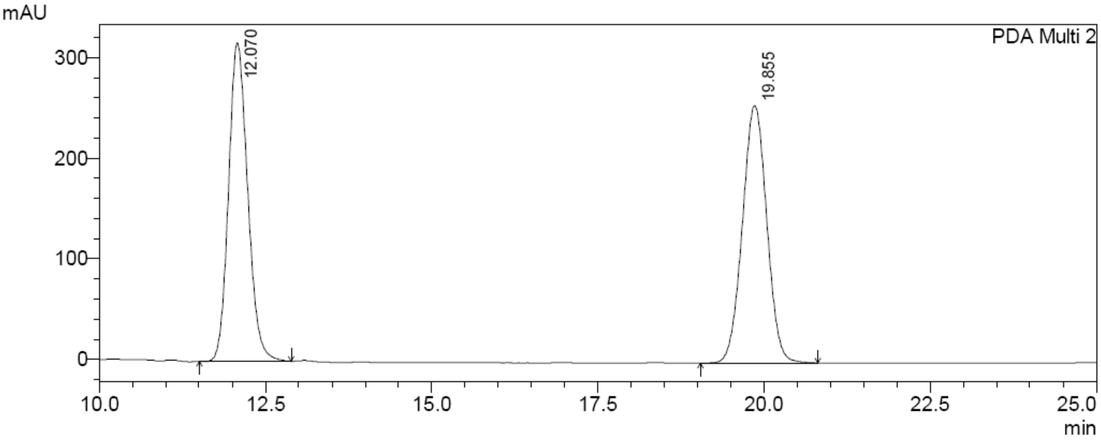

Peak#	Ret. Time	Area	Height	Area %	Height %
1	12.070	6346980	316879	49.310	55.310
2	19.855	6524590	256035	50.690	44.690
Total		12871570	572914	100.000	100.000

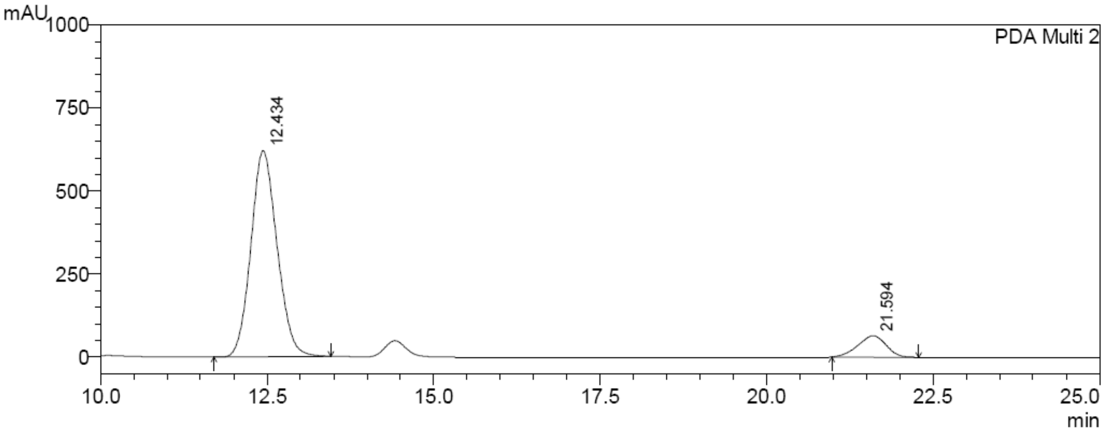

Peak#	Ret. Time	Area	Height	Area %	Height %
1	12.434	16989996	621031	89.860	90.561
2	21.594	1917280	64727	10.140	9.439
Total		18907276	685759	100.000	100.000

Supplementary Figure 53. ^{1}H and ^{13}C NMR spectra for compound 3za

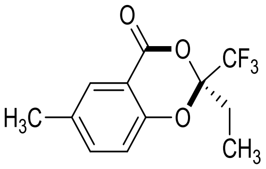

3za: 81%, 82:18 er

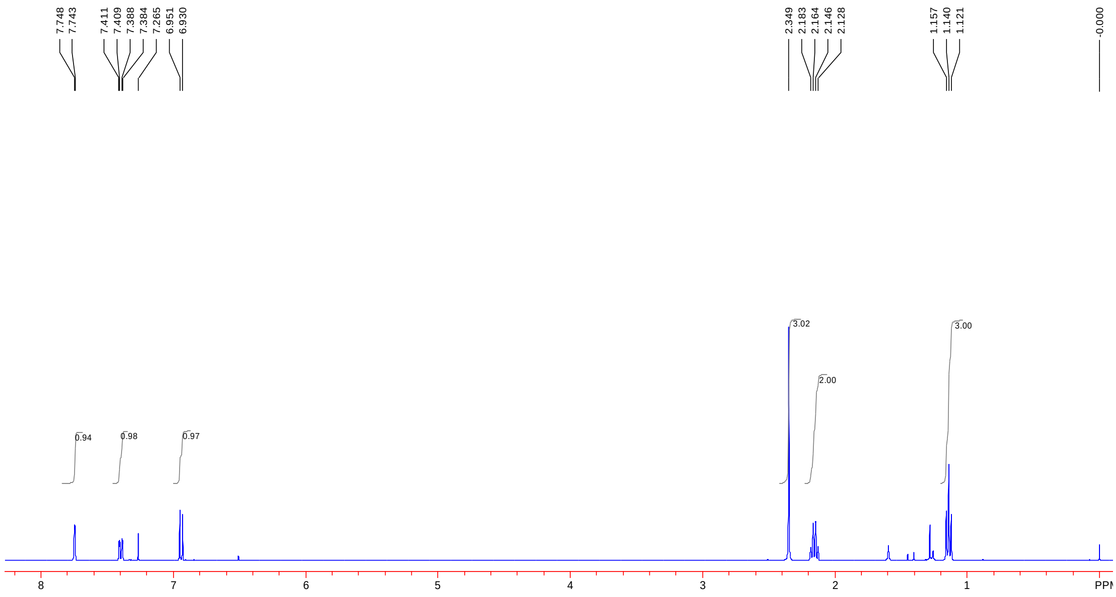

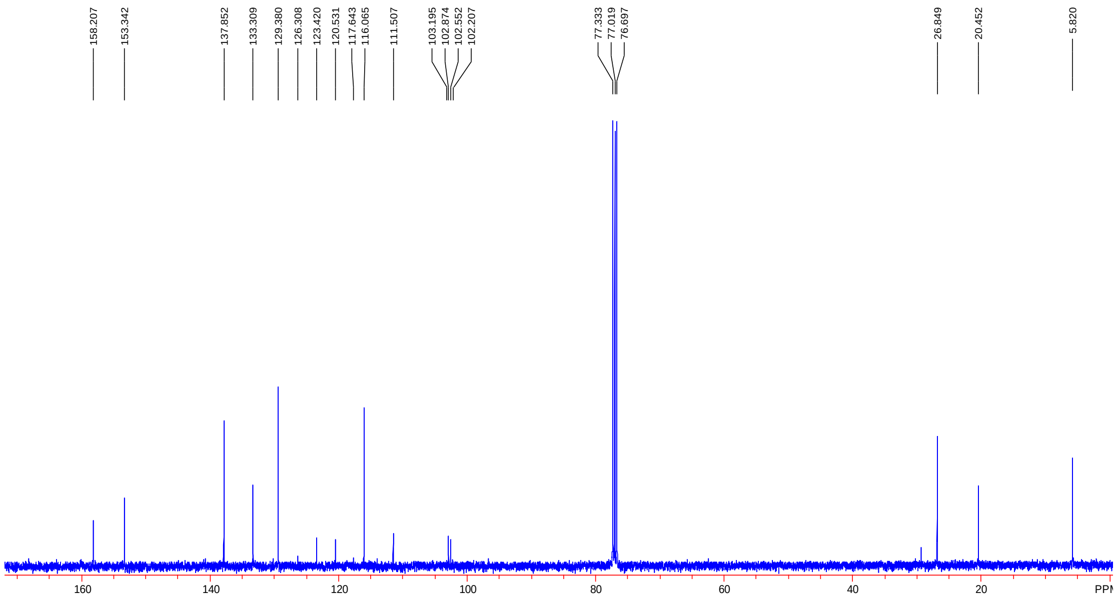

Supplementary Figure 54. ^{19}F and HPLC spectra for compound 3za

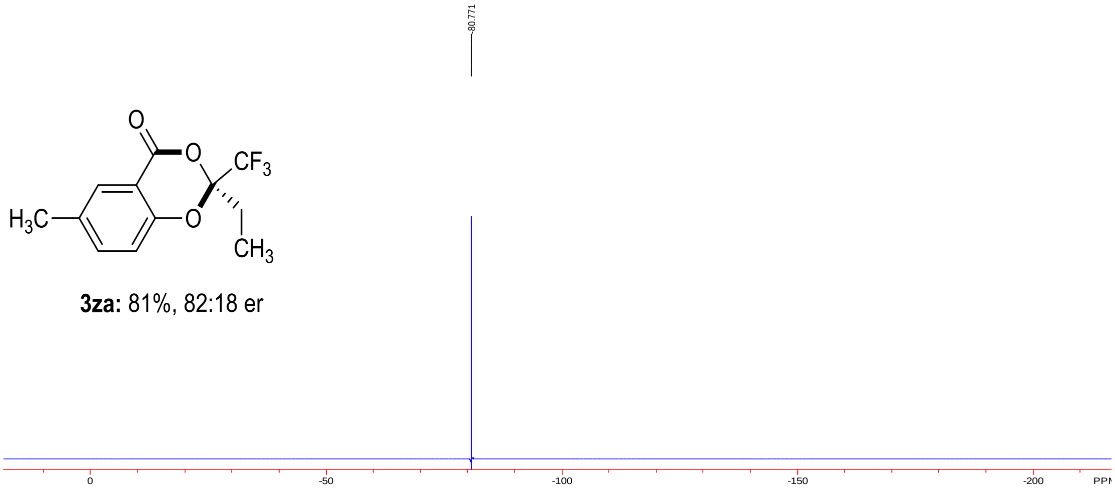

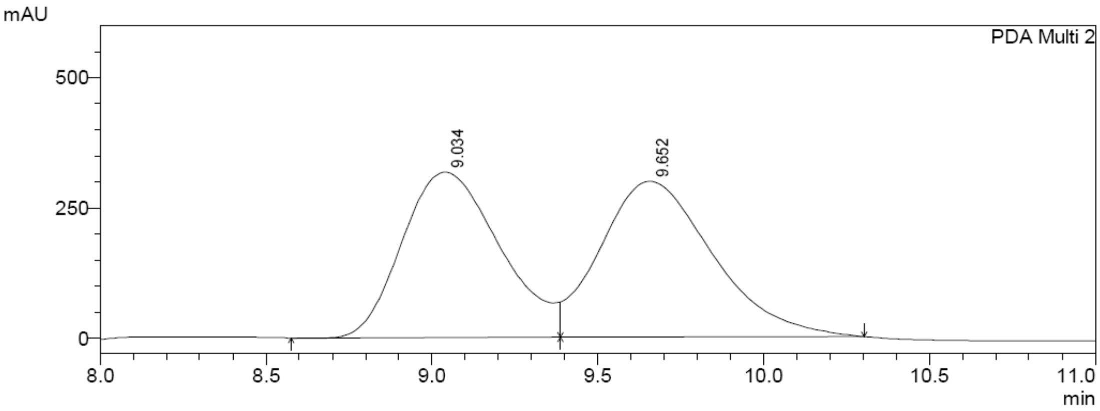

Peak#	Ret. Time	Area	Height	Area %	Height %
1	9.034	6539948	317265	48.196	51.463
2	9.652	7029529	299224	51.804	48.537
Total		13569478	616489	100.000	100.000

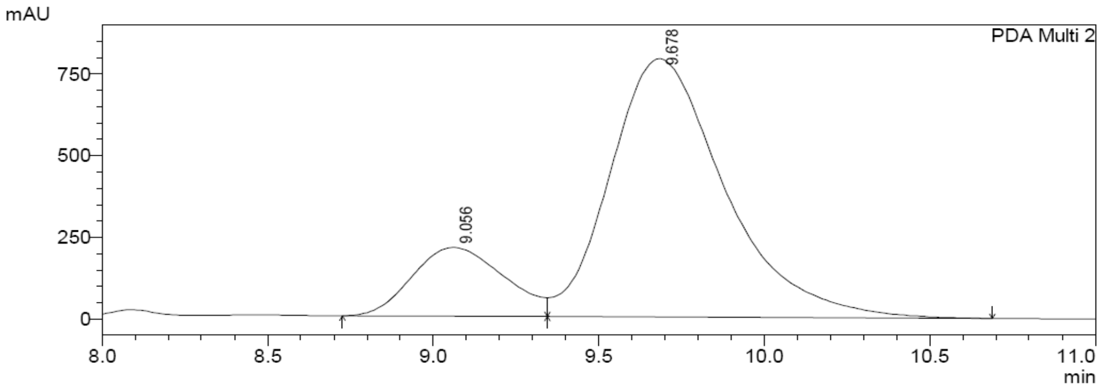

Peak#	Ret. Time	Area	Height	Area %	Height %
1	9.056	4082725	210775	17.662	21.043
2	9.678	19033357	790878	82.338	78.957
Total		23116082	1001653	100.000	100.000

Supplementary Methods

General Information

Commercially available materials purchased from Alfa Aesar or Sigma-Aldrich were used as received. Proton nuclear magnetic resonance (^1H NMR) spectra were recorded on a Bruker BBFO (400 MHz) spectrometer. Chemical shifts were recorded in parts per million (ppm, δ) relative to tetramethylsilane (δ 0.00) or chloroform (δ = 7.26, singlet). ^1H NMR splitting patterns are designated as singlet (s), doublet (d), triplet (t), quartet (q), dd (doublet of doublets); m (multiplets), and etc. All first-order splitting patterns were assigned on the basis of the appearance of the multiplet. Splitting patterns that could not be easily interpreted are designated as multiplet (m) or broad (br). Carbon nuclear magnetic resonance (^{13}C NMR) spectra were recorded on a Bruker BBFO (100 MHz) spectrometer. Fluorine (^{19}F) nuclear magnetic resonance (^{19}F NMR) spectra were recorded on a Bruker BBFO (376 MHz) spectrometer. IR spectra were recorded on a Shimadzu IR Prestige-21FT-IR spectrometer as neat thin films between NaCl plates. High resolution mass spectral analysis (HRMS) was performed on Finnigan MAT 95 XP mass spectrometer (Thermo Electron Corporation). The determination of enantiomeric excess was performed *via* chiral HPLC analysis using Shimadzu LC-20AD HPLC workstation. *X*-ray crystallography analysis was performed on Bruker X8 APEX *X*-ray diffractionmeter. Optical rotations were measured using a 1 mL cell with a 1 dm path length on a Jasco P-1030 polarimeter and are reported as follows: $[\alpha]^{rt}_D$ (*c* in g per 100 mL solvent). Analytical thin-layer chromatography (TLC) was carried out on Merck 60 F254 pre-coated silica gel plate (0.2 mm thickness). Visualization was performed using a UV lamp.

Reaction Conditions Optimization

Supplementary Table 1. Screening of different bases [a]

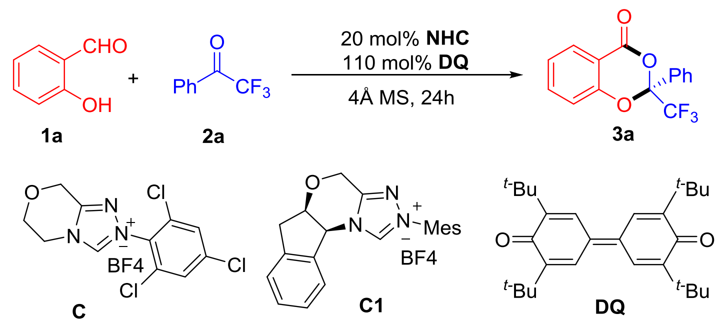

entry	NHC	solvent	Base	yield (%)[b]	er[c]
1	—	THF	DABCO	—	—
2	**C**	THF	DABCO	90	—
3	**C1**	THF	Cs_2CO_3	56	52:48
4	**C1**	THF	TEA	28	52:48
5	**C1**	THF	DMAP	19	52:48
6	**C1**	THF	DABCO	77	52:48
7	**C1**	THF	K_3PO_4	80	52:48
8	**C1**	THF	K_2CO_3	76	52:48

[a] Reaction conditions: **1a** (0.11 mmol), **2a** (0.1 mmol), base (0.1 mmol)，solvent (1 mL), rt, 24 h. [b] Yield determined by NMR analysis with an internal standard. [c] Enantiomeric ratio of **3a**, determined *via* chiral phase HPLC analysis. Mes = 2,4,6-Trimethylphenyl. THF = Tetrahydrofuran. TEA = Triethylamine. DABCO = 1,4-Diazabicyclo[2.2.2]octane. DMAP = 4-dimethylaminopyridine

Supplementary Table 2. Screening of different solvents [a]

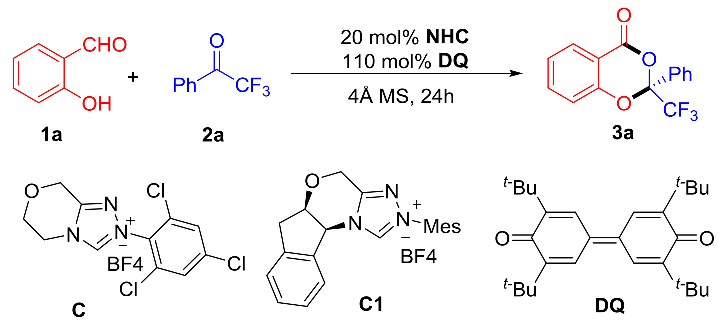

entry	NHC	solvent	Base	yield (%)[b]	er[c]
1	**C1**	THF	DABCO	77	52:48
2	**C1**	CH_2Cl_2	DABCO	72	63:37
3	**C1**	Toluene	DABCO	95	60:40
4	**C1**	MeCN	DABCO	30	50:50
5	**C1**	Ethyl Acetate	DABCO	22	50:50
6	**C1**	Hexane	DABCO	45	64:36
7	**C1**	DCE	DABCO	56	60:40

[a] Reaction conditions: **1a** (0.11 mmol), **2a** (0.1 mmol), base (0.1 mmol)，solvent (1 mL), rt, 24 h. [b] Yield determined by NMR analysis with an internal standard. [c] Enantiomeric ratio of **3a**, determined *via* chiral phase HPLC analysis. Mes = 2,4,6-Trimethylphenyl. DCE = 1,2-Dichloroethane. THF = Tetrahydrofuran. TEA = Triethylamine. DABCO = 1,4-Diazabicyclo[2.2.2]octane.

Supplementary Table 3. Screening of chiral carbene catalysts [a]

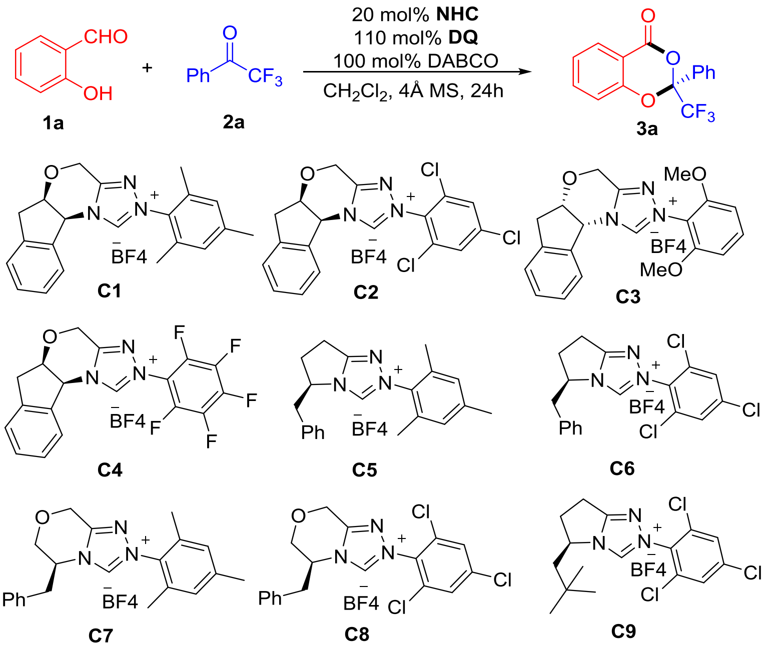

entry	NHC	Additive	yield (%)[b]	er[c]
1	**C1**	—	72	63:37
2	**C2**	—	98	68:32
3	**C3**	—	34	61:39
4	**C4**	—	94	53:47
5	**C5**	—	23	60:40
6	**C6**	—	27	52:48
7	**C7**	—	22	54:46
8	**C8**	—	97	55:45
9	**C9**	—	trace	—
10 [d]	**C2**	—	99	70:30
11 [d,e]	**C2**	—	99	73:27
12 [d,f]	**C2**	—	92	73:27

[a] Reaction conditions: **1a** (0.11 mmol), **2a** (0.1 mmol), CH_2Cl_2 (1 mL), rt, 24 h. [b] Yield determined by NMR analysis with an internal standard. [c] Enantiomeric ratio of **3a**, determined *via* chiral phase HPLC analysis. [d] CH_2Cl_2/Hexane = 1:1 (2 mL). [e] -10 °C. [f] -40 °C, 48 h.

Supplementary Table 4. Screening of chiral bases and additives [a]

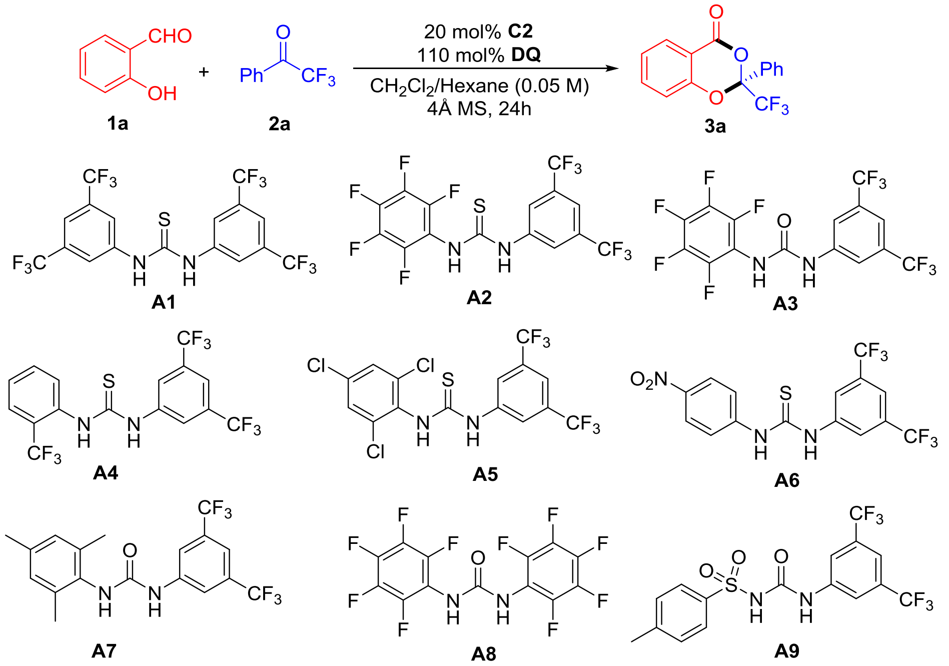

entry	Base (100 mol%)	Additive (20 mol%)	yield (%)[b]	er[c]
1	Quinine	—	99	72:28
2	Quinidine	—	99	71:29
3	DABCO	**A1**	90	85:15
4	DABCO	**A2**	99	92:8
5	DABCO	**A3**	99(99)	94:6
6	DABCO	**A4**	95	92:8
7	DABCO	**A5**	99	93:7
8	DABCO	**A6**	99	86:14
9	DABCO	**A7**	89	87:13
10	DABCO	**A8**	99	89:11
11	DABCO	**A9**	99	87:13
12 [d]	DABCO	**A3**	99	94:6
13	Cs_2CO_3	**A3**	93	94:6

[a] Reaction conditions: **1a** (0.11 mmol), **2a** (0.1 mmol), DCM/Hexane =1:1 (2 mL), -10 ℃, 24 h. [b] Yield determined by NMR analysis with an internal standard. Isolated yield in parentheses based on **2a**. [c] Enantiomeric ratio of **3a**, determined *via* chiral phase HPLC analysis. [d] 5 mol% **C2**. DABCO = 1,4-Diazabicyclo[2.2.2]octane.

General procedure for the catalytic reactions:

General procedure for the catalytic synthesis of products 3:

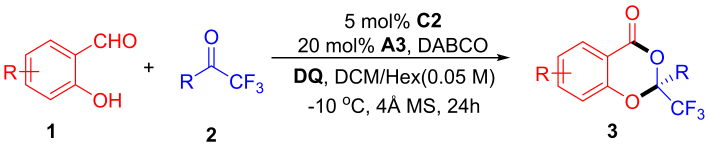

To a 10 mL flame-dry Schlenk reaction tube equipped with a magnetic stir bar, was added chiral NHC pre-catalyst **C2** (0.005 mmol, 5 mol%, 2.4 mg), urea **A3** (0.02 mmol, 20 mol%, 8.76 mg), DABCO (0.1 mmol, 100 mol%, 11.2 mg), oxidant **DQ** (0.11 mmol, 110 mol%, 45 mg), aldehyde (0.11 mmol) and 4 Å molecular sieves. The Schlenk tube was sealed with a septum, evacuated and refilled with nitrogen (3 cycles). Solvent (CH_2Cl_2/Hexane =1:1, 2.0 mL) and trifluoromethyl ketone **2** (0.1 mmol) were then added via syringe. The reaction mixture was allowed to stir for 24 hours at -10℃. After completion of the reaction, monitored by TLC plate, the reaction mixture was concentrated under reduced pressure and the residue was subjected to column chromatography or TLC plate directly using hexane/EtOAc as eluent to afford the desired product **3**.

Procedure for scale-up synthesis of 3b with 1 mol% carbene catalyst:

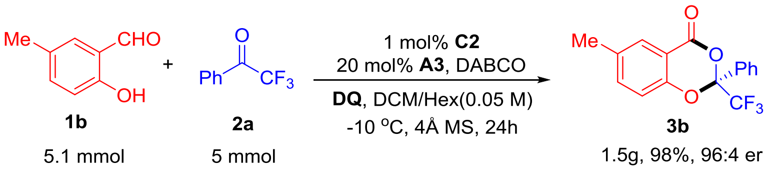

To a 250 mL flame-dry two-neck round bottom flask (RBF) equipped with a magnetic stir bar, was added chiral NHC pre-catalyst **C2** (0.05 mmol, 1 mol%, 24 mg), urea **A3** (1.0 mmol, 20 mol%, 438 mg), DABCO (5.0 mmol, 100 mol%, 560 mg), oxidant **DQ** (5.1 mmol, 102 mol%, 2.08 mg), aldehyde (5.1 mmol, 694mg) and 4 Å molecular sieves. The RBF was sealed with a septum, evacuated and refilled with nitrogen (3 cycles). Solvent (CH_2Cl_2/Hexane =1:1, 100 mL) and trifluoromethyl ketone **2** (5.0 mmol, 870 mg) were then added via syringe. The reaction mixture was allowed to stir for 24 hours at -10℃. After completion of the reaction, monitored by TLC plate, the reaction mixture was concentrated under reduced pressure and the residue was subjected to column chromatography directly using hexane/EtOAc (20/1) as eluent to afford the desired product **3b** 1.5g in 98% yield and 96:4 er.

Procedure for using MnO_2 as terminal oxidant:

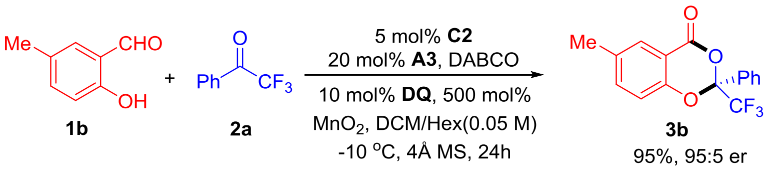

To a 10 mL flame-dry Schlenk reaction tube equipped with a magnetic stir bar, was added chiral NHC pre-catalyst **C2** (0.005 mmol, 5 mol%, 2.4 mg), urea **A3** (0.02 mmol, 20 mol%, 8.76 mg),

DABCO (0.1 mmol, 100 mol%, 11.2 mg), oxidant **DQ** (0.01 mmol, 10 mol%, 4.1 mg), MnO_2 (0.5 mmol, 500 mol%, 43 mg), aldehyde (0.11 mmol) and 4 Å molecular sieves. The Schlenk tube was sealed with a septum, evacuated and refilled with nitrogen (3 cycles). Solvent (CH_2Cl_2/Hexane =1:1, 2.0 mL) and trifluoromethyl ketone **2** (0.1 mmol) were then added via syringe. The reaction mixture was allowed to stir for 24 hours at -10℃. After completion of the reaction, monitored by TLC plate, the reaction mixture was concentrated under reduced pressure and the residue was subjected to column chromatography directly using hexane/EtOAc as eluent to afford the desired product **3b** with 95% yield and 95:5 er.

Note: Racemic samples for the chiral phase HPLC analysis were prepared using **C** as the NHC pre-catalyst.

Stereochemistry determination via *X*-ray crystallographic analysis:

Good quality crystal of **3h** (colorless needle crystal) was obtained by vaporization of a hexane/ethyl acetate solution of compound **3c** (~500mg). CCDC 1486140 contains the supplementary crystallographic data for this paper. These data can be obtained free of charge from The Cambridge Crystallographic Data Centre via www.ccdc.cam.ac.uk/data_request/cif.

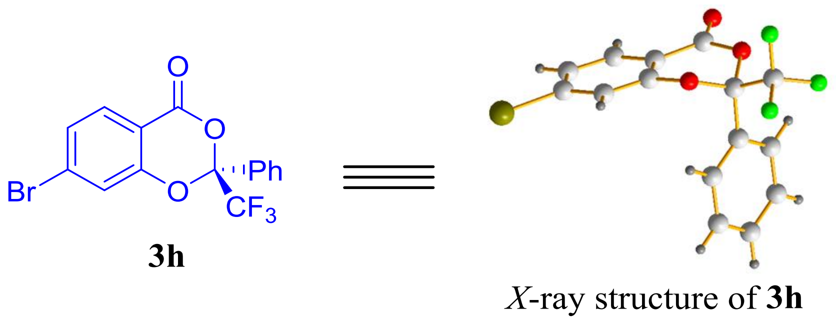

Supplementary Figure 55 *X*-ray structure of product 3h

Proof of mechanism studies:

Supplementary Figure 56. ^{1}H spectra for starting material and blank reaction without carbene

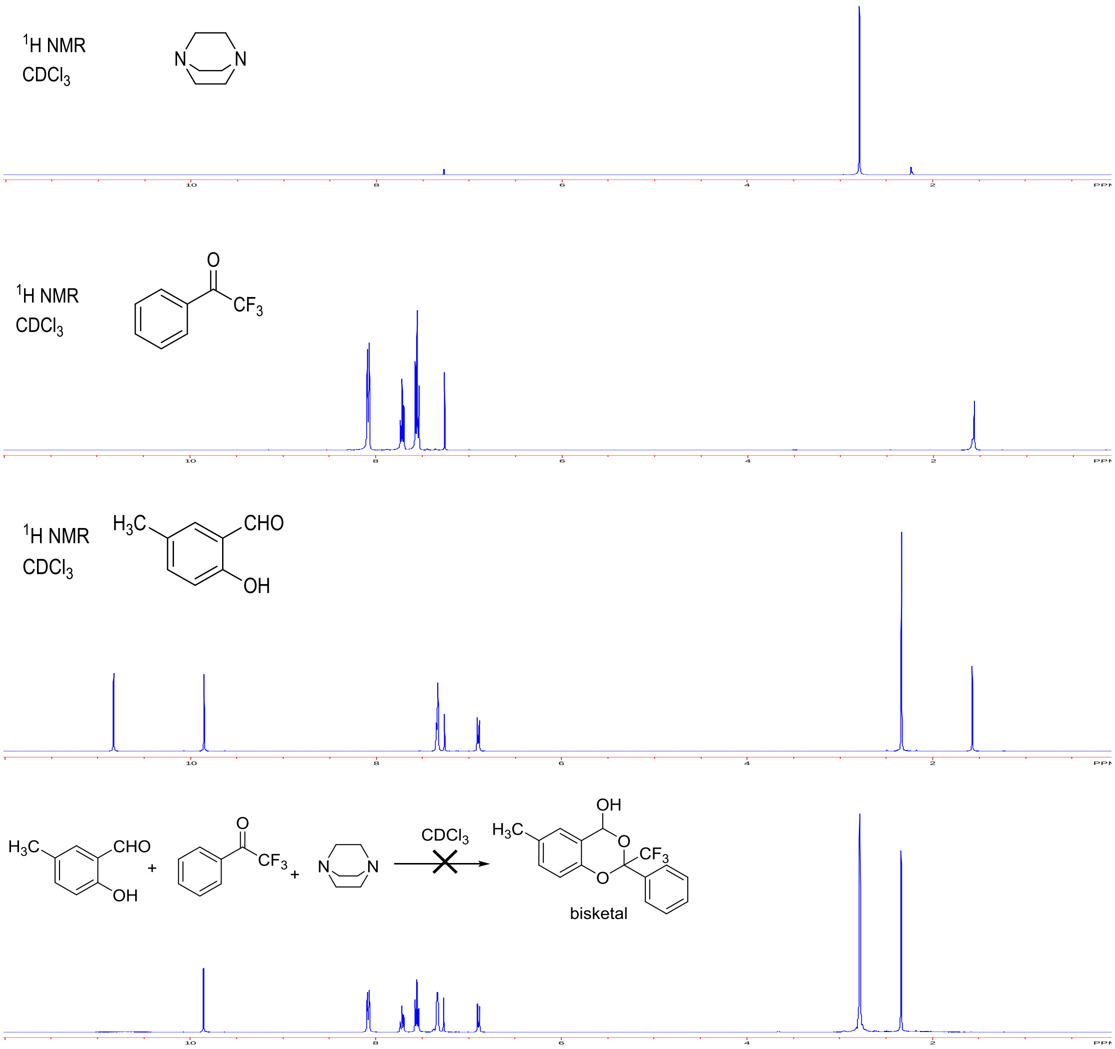

Based on ^{1}H NMR analysis, without carbene catalyst, there almost no background reaction or formation of bisketal intermediate.

Supplementary Figure 57. ^{19}F and ^{1}H spectra for urea and ketone with different solvent

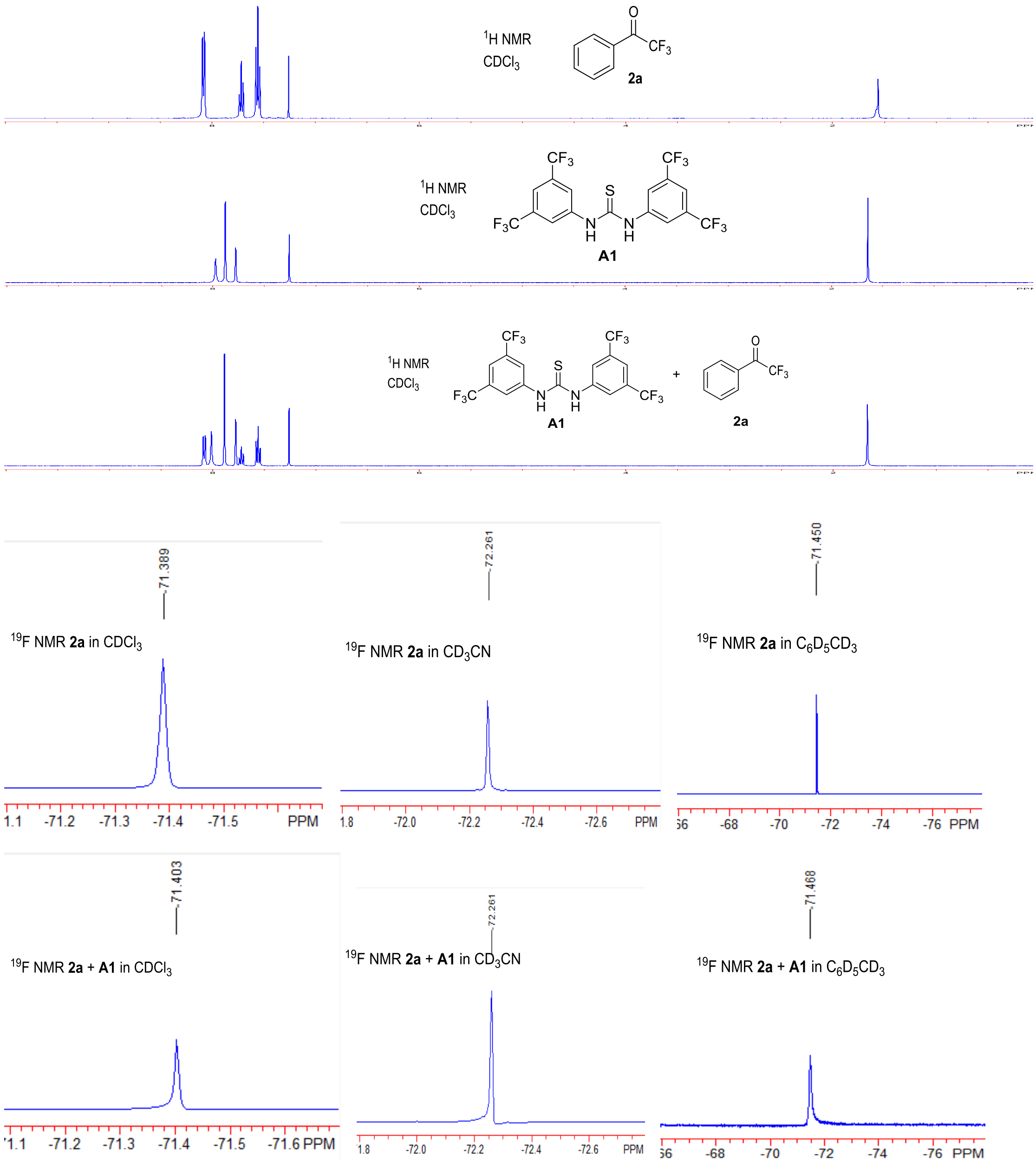

Based on ^{1}H NMR spectra, we can found that thiourea co-catalyst dose not react with ketone substrate.

Based on ^{19}F NMR spectra, we can see that in non-polar solvents such as $CDCl_3$ and d8-toluene, in the presence of thiourea, the chemical shift of ^{19}F was changed compared to no thiourea one (although very small difference, it is also reasonable for weak hydrogen bonding effect). However, in polar solvent such as CD_3CN, the chemical shift of ^{19}F totally same no matter with or without thiourea. Therefore, thiourea co-catalyst can serve as H-bonding donor, which is consistent with our proposal.

Computational method for mechanism studies:

We performed density functional theory (DFT) calculations using Gaussian 09 software[1]. For geometry optimization calculations, the B3LYP functional[2-4] was used in combination with the 6-311G(d) basis set.[5,6] Single-point calculations on the optimized structures were performed using the dispersion-corrected B3LYP (B3LYP-D3)[2-4,7-11] functional and the 6-311+G(d,p) basis set,[5,6,12] with Grimme's D3 correction[7,8] combined with Becke-Johnson (BJ) damping.[9-11] The solvent effect of DCM was taken into account using the IEF-PCM method[13] in single-point calculations. Although a mixed solvent (CH_2Cl_2/hexane = 1:1) was used in the optimized experimental condition, for the sake of simplicity, we examined the effect of CH_2Cl_2 because similar e.r. was observed experimentally with this solvent. Vibrational frequency calculations were performed to confirm that each optimized structure resides at a stationary point on the potential energy surface. Free energy corrections were also obtained from frequency calculations. All relative energies are free energy at -10 ℃ (263.15 K) and 1 atm. Mulliken atomic charges were calculated at the B3LYP/6-311G(d) level.

Conformation of triazolium salt precursor C2

Triazolium salt precursor **C2** has two conformers (A and B in Fig. S58) that differ in the geometry of the oxygen-containing ring. Conformer A is more stable than conformer B by 1.3 kcal/mol. Therefore, in the following calculations, conformer A was used for **C2**.

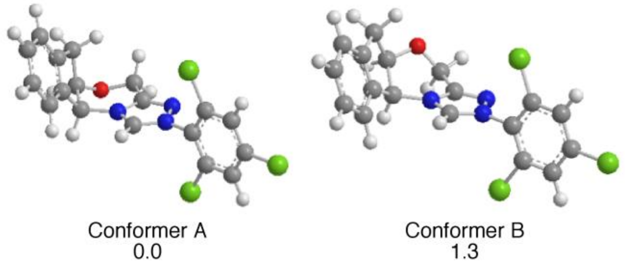

Supplementary Figure 58. Optimized structures of triazolium salt precursor C2. Relative free energies are shown in kcal/mol and are with respect to conformer A.

Optimized structures of conformers of intermediate II

DFT calculations were used to assess the nature of *o*-QM in intermediate **II**. Six conformers of **II** were obtained from DFT calculations, and **II-1** was the most stable one. A weak CH···O hydrogen bond between the phenolate oxygen and hydrogen atom of the indane moiety from the NHC catalyst was formed in **II-1** but not in **II-2**, which makes the intermediate **II-1** more stable by 0.9 kcal/mol. **II-3** and **II-4** are even less stable, probably because the phenolate oxygen points away from the NHC group, leading to weaker electrostatic stabilization. **II-5** and **II-6** (Fig. S59), in which a carbon atom of the azolium ring forms a covalent bond with the phenolate oxygen atom, are less stable than **II-1**. The covalent bond in **II-5** and **II-6** prevents the azolium ring from gaining resonance stabilization that **II-1** can have, and this explains the difference in stability of these intermediates. Key Mulliken atomic charges for **II-2** are given in Supplementary Table 5. The charge distribution in **II-2** is similar to that in **II-1**.

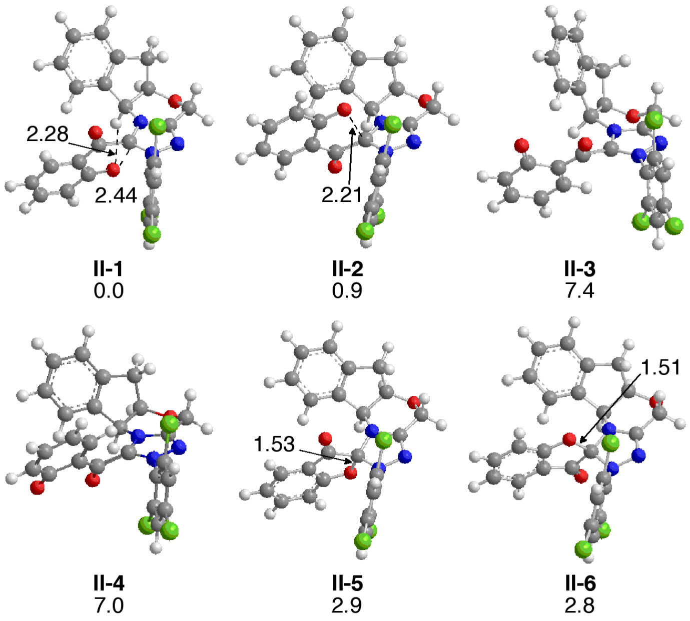

Supplementary Figure 59. Optimized structures of the six conformers of intermediate II. Relative free energies are shown in kcal/mol and are with respect to II-1. Key interatomic distances are indicated in Å.

Supplementary Table 5. Key Mulliken atomic charges for II-2.

Group	Mulliken Charge
+NHC	0.51
C1	0.20
O1	-0.35
O2	-0.45

Mechanisms of [4+2] annulation without urea

The calculations excluding urea suggest that NHC-bound intermediate **II** can react with ketone substrate **2a** in a [4+2] mechanism (Fig. 1c) to afford a ketal-like product, without having too high barriers. Our calculations also suggest that the reaction should consist of two steps: (1) a ring-annulation step and (2) an NHC dissociation step (see Fig. S60). The four conformers (**II-1**, **II-2**, **II-3**, and **II-4**) in Fig S59 can react with **2a**, giving rise to several possible reaction pathways (16 pathways in total). Nevertheless, we here focus on low-energy pathways. When the resultant product does not have the predominant stereochemical pattern observed in the experiment, “minor” is attached to the labels. The *o*-QM moiety has two distinct faces on which **2a** can possibly attack, and thus reaction pathways may be classified into Paths A and B; Path A features the attack of **2a** on **II** from the “indane side” depicted in Fig. S61, whereas Path B involves the attack from the “$PhCl_3$ side” in Fig. S61. For example, Path 1-A corresponds to the attack of **2a** on **II-1** from the indane side. Each of Paths A and B can also have two pathways that differ in the orientation of substrate **2a**.

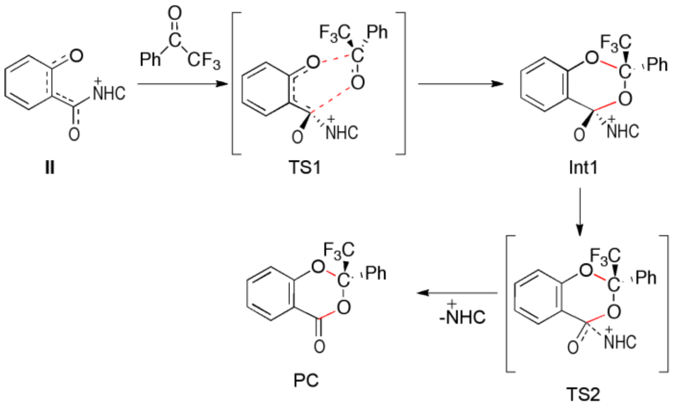

Supplementary Figure 60. Reaction mechanism obtained from DFT calculations

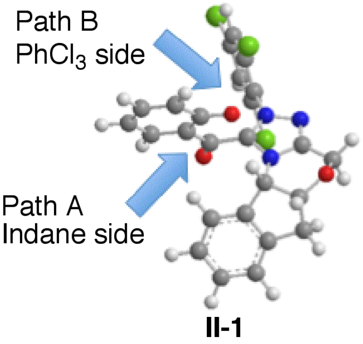

Supplementary Figure 61. The indane and $PhCl_3$ sides of II-1

[4+2] annulation pathways without urea for intermediates II-1 or II-2

Our DFT calculations on the reactions of **II-1** and **II-2** with **2a** without urea showed that this reaction consists of two steps: (1) a ring-annulation step and (2) an NHC dissociation step. It should be noted that Path 1-A proceeds in a concerted fashion and the optimized structure of $TS2_{1\text{-}A}$ is similar to those of several other TS2s for the NHC dissociation step. After the formation of the product (the minor enantiomeric product), the NHC catalyst is recovered and may abstract a proton from H^+-DABCO (protonated base) to regenerate the triazolium salt precursor. The regeneration of the triazolium salt precursor is exothermic by 8.2 kcal/mol (Fig. S62).

Supplementary Table 6. Relative free energies of different species formed in four [4+2] annulation pathways for II-1

	RC	TS1	Int1	TS2	PC
Path 1-A	0.0	-	-	16.8	5.7
Path 1-A-minor	1.1	13.4	13.0	18.3	5.6
Path 1-B	2.1	13.9	13.7	19.5	1.3
Path 1-B-minor	1.9	12.8	12.3	18.4	4.5

*Relative free energies are shown in kcal/mol and are with respect to $RC_{1\text{-}A}$

Supplementary Table 7. Relative free energies of different species formed in four [4+2] annulation pathways for II-2

	RC	TS1	Int1	TS2	PC
Path 2-A	5.1	13.2	12.3	17.7	8.7
Path 2-A-minor	3.3	10.4	10.0	17.4	6.3
Path 2-B	2.3	17.0	14.9	19.5	6.8
Path 2-B-minor	1.3	18.9	14.6	19.9	7.7

*Relative free energies are shown in kcal/mol and are with respect to $RC_{1\text{-}A}$

Supplementary Table 8. Relative free energies of key species in Path 1-A-minor at B3LYP-D3/6-311+G(d,p) or M06-2X/6-311+G(d,p) (ref. 14).

	RC	TS1	Int1	TS2
B3LYP-D3	0.0	12.3	11.9	17.2
M06-2X[1]	0.0	4.5	4.2	4.2

[1]Dispersion corrections were calculated using Grimme's D3 correction combined with zero-damping.

*Relative free energies are shown in kcal/mol and are with respect to $RC_{1\text{-}A\text{-}minor}$.

*Geometry optimization was performed at the B3LYP/6-311G(d) level.

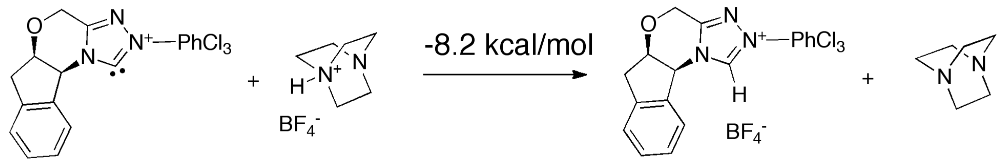

Supplementary Figure 62. Regeneration of the triazolium salt precursor from the NHC catalyst.

[4+2] annulation pathways without urea for intermediates II-3 or II-4

We also examined the reactions of conformers **II-3** and **II-4** with **2a**, and in these conformers, the phenolate oxygen points away from the NHC group. The reaction on Path 3-A can take place via a 6-membered ring intermediate ($Int1_{3\text{-}A}$), which should be structurally equivalent to $Int1_{2\text{-}B}$, as illustrated in Fig. S63. In a similar fashion, Paths 3-B, 4-A, and 4-B also involve 6-membered ring intermediates, which should be structurally equivalent to $Int1_{2\text{-}A}$, $Int1_{1\text{-}B}$, and $Int1_{1\text{-}A}$, respectively. However, as shown in Tables S9 and S10, Int1's on Paths 3-A, 3-B, 4-A, and 4-B are not as stable as Int1's on Paths 2-B, 2-A, 1-B, and 1-A, respectively. It should be noted that these energy differences are small, which therefore suggests that Int1's on Paths 3-A, 3-B, 4-A, and 4-B may be readily converted into the corresponding Int1's (Int1's in Tables S9 and S10) before the NHC dissociation process. Thus, we assumed that TS2's and PC's on Paths 3-A, 3-B, 4-A, and 4-B are equivalent to those on Paths 2-B, 2-A, 1-B, and 1-A, respectively.

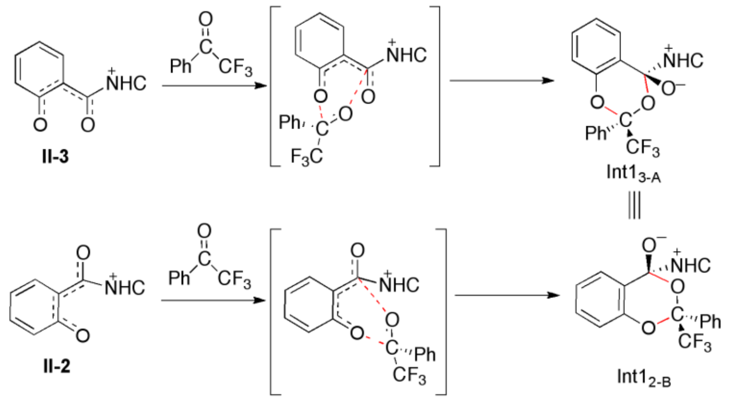

Supplementary Figure 63. The first step of the [4+2] annulation in Paths 3-A and 2-B

Supplementary Table 9. Relative free energies of different species formed in four [4+2] annulation pathways for II-3

	RC	TS1	Int1	Int1'	TS2	PC
Path 3-A	7.9	12.7	11.7	14.9 ($Int1_{2\text{-}B}$)	19.5 ($TS2_{2\text{-}B}$)	6.8 ($PC_{2\text{-}B}$)
Path 3-A-minor	10.0	14.4	14.2	14.6 ($Int1_{2\text{-}B\text{-}minor}$)	19.9 ($TS2_{2\text{-}B\text{-}minor}$)	7.7 ($PC_{2\text{-}B\text{-}minor}$)
Path 3-B	7.4	19.1	12.9	12.3 ($Int1_{2\text{-}A}$)	17.7 ($TS2_{2\text{-}A}$)	8.7 ($PC_{2\text{-}A}$)
Path 3-B-minor	7.3	16.6	14.4	10.0 ($Int1_{2\text{-}A\text{-}minor}$)	17.4 ($TS2_{2\text{-}A\text{-}minor}$)	6.3 ($PC_{2\text{-}A\text{-}minor}$)

*Relative free energies are shown in kcal/mol and are with respect to $RC_{1\text{-}A}$.

Supplementary Table 10. Relative free energies of different species formed in four [4+2] annulation pathways for II-4

	RC	TS1	Int1	Int1'	TS2	PC
Path 4-A	8.2	15.3	14.7	13.7 ($Int1_{1\text{-}B}$)	19.5 ($TS2_{1\text{-}B}$)	1.3 ($PC_{1\text{-}B}$)
Path 4-A-minor	6.4	12.9	12.3	12.3 ($Int1_{1\text{-}B\text{-}minor}$)	18.4 ($TS2_{1\text{-}B\text{-}minor}$)	4.5 ($PC_{1\text{-}B\text{-}minor}$)
Path 4-B	8.7	19.0	15.6	-	16.8 ($TS2_{1\text{-}A}$)	5.7 ($PC_{1\text{-}A}$)
Path 4-B-minor	8.3	21.6	21.9	13.0 ($Int1_{1\text{-}A\text{-}minor}$)	18.3 ($TS2_{1\text{-}A\text{-}minor}$)	5.6 ($PC_{1\text{-}A\text{-}minor}$)

*Relative free energies are shown in kcal/mol and are with respect to $RC_{1\text{-}A}$

Reaction mechanisms with urea co-catalyst

Our DFT calculations without urea showed that step 2 is more important than step 1. We calculated the reaction mechanism in Path 1-A with thiourea **A1** and obtained three transition states, namely A1-$TS1_{1\text{-}A}$, A1-$TS1'_{1\text{-}A}$, and A1-$TS2_{1\text{-}A}$, as shown in Fig. S64. As the reaction without urea, the NHC dissociation step has the highest energy. Overall, the reaction looks endothermic, but after the formation of PC, NHC can be further stabilized by forming a triazolium salt precursor (Fig. S62). Note that **A1** is attached on the label when **A1** is added in the system. Thiourea **A1** interacts with ketone through a hydrogen bond that should make ketone more reactive, and therefore the activation energy for the ring-annulation step should be lowered. In the calculations with **A1**, we examined several binding modes of **A1** to find the most stable $TS1_{1\text{-}A}$ and

$TS2_{1\text{-}A}$. We obtained one binding mode for A1-$TS1_{1\text{-}A}$ (Fig. S64) and two binding mode for A1-$TS2_{1\text{-}A}$ (Fig. S65). A1-$TS2_{1\text{-}A}$, in which **A1** forms hydrogen bonds with the carbonyl oxygen of *o*-QM, is more stable than A1-$TS2_{1\text{-}A'}$, in which A1 forms hydrogen bonds with the CF_3 group of ketone. It should be noted that A3-TS2's, which are involved in the reaction with urea **A3**, should be the rate-determining step because the urea moiety of both **A1** and **A3** should interact with the same atom(s) through hydrogen bond(s) in each key species, and therefore A1-TS1's and A3-TS1's should be stabilized by the same hydrogen bond(s).

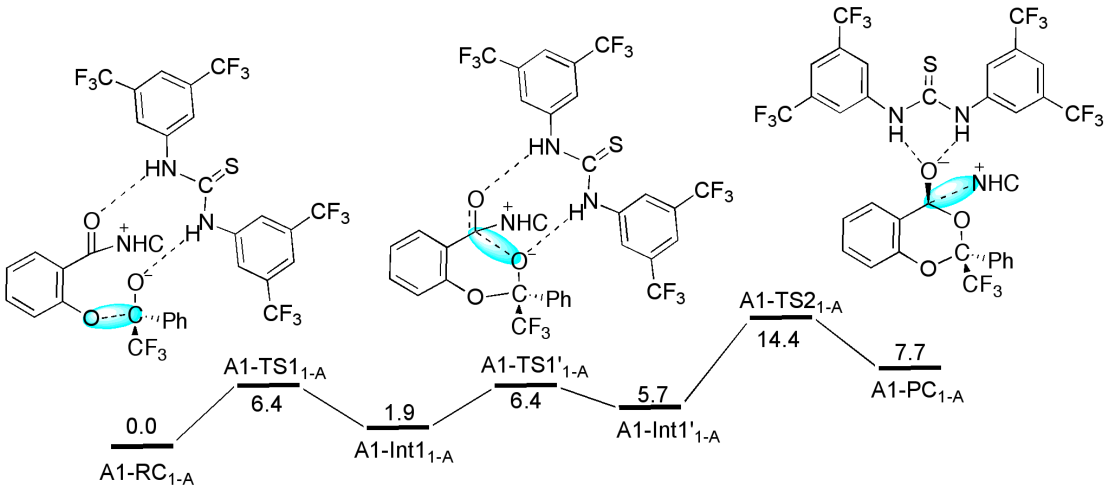

Supplementary Figure 64. Energy diagram of the reaction with urea A1 and structures of key species. Relative free energies are shown in kcal/mol and are with respect to A1-$RC_{1\text{-}A}$.

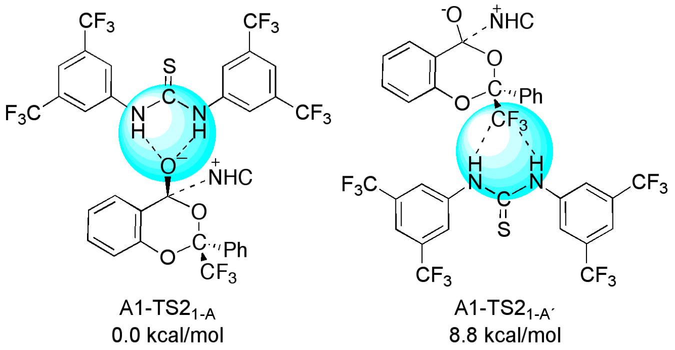

Supplementary Figure 65. Schematic drawing of A1-$TS2_{1\text{-}A}$ and A1-$TS2_{1\text{-}A'}$. Relative free energies are shown in kcal/mol and are with respect to A1-$TS2_{1\text{-}A}$.

Supplementary Table 11. Relative free energies of transition states in Path 1-A with urea A1 at the B3LYP-D3/6-311+G(d,p) and M06-2X/6-311+G(d,p) levels.

	A1-$TS1_{1\text{-}A}$	A1-$TS1'_{1\text{-}A}$	A1-$TS2_{1\text{-}A}$
B3LYP-D3	0.0	0.0	7.7
M06-2X[1]	0.0	0.0	5.8

[1]Dispersion corrections were calculated using Grimme's D3 correction combined with zero-damping.

*Relative free energies are shown in kcal/mol and are with respect to TS1.

*Geometry optimization was performed at the B3LYP/6-311G(d) level.

Energy differences of A3-TS2's

We further investigated each A3-TS2's to identify the key factors that control the enantioselectivity (Table S12). A3-TS2$_{1\text{-A}}$, which is the lowest-energy in A3-TS2, was found to have attractive π-π stacking between the pentafluorophenyl group of urea **A3** and the indane moiety of the catalyst (Fig. 2c). Such π-π stacking was also observed in A3-TS2$_{1\text{-A-minor}}$, but was not found in other A3-TS2's. Therefore, A3-TS2$_{1\text{-A}}$ and A3-TS2$_{1\text{-A-minor}}$ are more stable than the other A3-TS2's. Note that we examined several binding modes between TS2 and urea **A3** in each pathway to obtain the lowest-energy A3-TS2 (Table S13).

Supplementary Table 12. Relative free energies of TS2 with urea A3 calculated at the B3LYP-D3/6-311+G(d,p) and M06-2X/6-311+G(d,p) levels.

	A3-TS2$_{1\text{-A}}$	A3-TS2$_{1\text{-A-minor}}$	A3-TS2$_{1\text{-B}}$	A3-TS2$_{1\text{-B-minor}}$	A3-TS2$_{2\text{-A}}$	A3-TS2$_{2\text{-A-minor}}$	A3-TS2$_{2\text{-B}}$	A3-TS2$_{2\text{-B-minor}}$
B3LYP-D3	0.0	2.8	4.1	3.9	4.2	4.3	4.4	4.7
M06-2X[1]	0.0	2.7	5.8	3.6	4.3	4.0	3.9	5.8

[1]Dispersion corrections were calculated using Grimme's D3 correction combined with zero damping.

*Relative free energies are shown in kcal/mol and are with respect to TS2$_{1\text{-A}}$.

*Geometry optimization was performed at the B3LYP/6-311G(d) level.

Supplementary Table 13. Free energy differences of optimized structures of A3-TS2's

	A3-TS2$_{1\text{-A}}$	A3-TS2$_{1\text{-A-minor}}$	A3-TS2$_{1\text{-B}}$	A3-TS2$_{1\text{-B-minor}}$	A3-TS2$_{2\text{-A}}$	A3-TS2$_{2\text{-A-minor}}$	A3-TS2$_{2\text{-B}}$	A3-TS2$_{2\text{-B-minor}}$
1	0.0	2.8	4.1	3.9	4.2	4.3	4.4	4.7
2	2.9	4.3	4.4	4.2	5.0	5.5	6.5	5.1
3	3.5	6.5	4.8	-	-	6.1	-	-
4	-	-	5.1	-	-	6.6	-	-

*Relative free energies are shown in kcal/mol and are with respect to A3-TS2$_{1\text{-A}}$

In Vitro antibacterial bioassay

Antifungal activities test

The antifungal activities were tested against three pathogenic fungi, *Eggplant verticillium*, *Phytophthora infestans* and *Fusarium oxysporum*, by the poison plate technique. Compounds were dissolved in 1 mL DMSO and diluted with sterile distilled water containing 0.1% Tween-20 (9mL) before mixing with 90 mL potato dextrose agar (PDA). The compounds were tested at a concentration of 50 μg/mL. All kinds of fungi were incubated in PDA at 27±1 ˚C for 4 days to get new mycelium for antifungal assay. Then mycelia dishes of approximately 4 mm diameter were cut from culture medium and one of them was picked up with a sterilized inoculation needle and inoculated in the center of PDA plate aseptically. The inoculated plates were incubated at 27±1 ˚C for 5 days. DMSO in sterile distilled water served as negative control, while Kresoxim-methyl acted as positive controls. For each treatment, three replicates were conducted. The radial growth of the fungal colonies was measured and the data were statistically analyzed. The inhibiting effects of the test compounds *in vitro* on these fungi were calculated by the formula:

$$\text{Inhibition rate (\%)} = [(C\text{-}T)/(C\text{-}0.4)]\times 100$$

"*C*" represents the diameter of fungi growth on untreated PDA, and "*T*" means the diameter of fungi on treated PDA.

Characterization of products 3:

(*S*)-2-phenyl-2-(trifluoromethyl)-4H-benzo[*d*][1,3]dioxin-4-one (3a): colorless oil, 99% yield, 29.2 mg

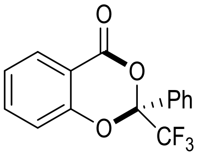

3a: 99%, 94:6 er

^{1}H NMR (400 MHz, $CDCl_3$) δ 7.08 (t, *J* = 7.6 Hz, 1H), 7.11 (d, *J* = 1.6 Hz, 1H), 7.19-7.41 (m, 3H), 7.58 (t, *J* = 8.8 Hz, 1H), 7.64 (t, *J* = 6.4 Hz, 2H), 7.83 (dd, *J* = 1.6, 7.6 Hz, 1H);

^{13}C NMR (100 MHz, $CDCl_3$) δ 101.5 (q, *J* = 34.5 Hz), 113.9, 117.4, 120.3 (q, *J* = 283 Hz), 124.0, 128.1, 128.8, 130.1, 131.1, 131.4, 137.2, 154.6, 157.9;

^{19}F NMR (376 MHz, $CDCl_3$) δ -83.2;

IR ν_{max} (film, cm^{-1}): 1762, 1467, 1300, 1265, 1124, 738; **$[\alpha]^{23}_D$** = 69.5 (*c* = 1.3 in $CHCl_3$);

HRMS (ESI, m/z): calcd. for $C_{15}H_9F_3O_3H^+$ 295.0577, found 295.0574;

HPLC analysis: 94:6 er, [CHIRALPAK IC column; 0.6 mL/min; solvent system: *i*-PrOH/hexane = 5:95; retention times: 7.8 min (major), 7.5 min (minor)].

***(S)*-6-methyl-2-phenyl-2-(trifluoromethyl)-4H-benzo[*d*][1,3]dioxin-4-one (3b):** colorless oil. 99% yield, 30.6 mg

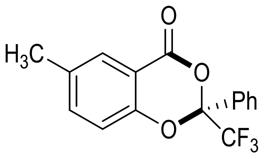

3b: 99%, 96:4 er

^{1}H NMR (400 MHz, $CDCl_3$) δ 2.26 (s, 3H), 7.07 (d, *J* = 8.4 Hz, 1H), 7.35-7.41 (m, 4H), 7.61-7.65 (m, 3H);

^{13}C NMR (100 MHz, $CDCl_3$) δ 20.4, 101.4 (q, *J* = 34.3 Hz), 113.6, 117.1, 120.3 (q, *J* = 282.7 Hz), 128.2, 128.8, 129.9, 130.0, 131.6, 133.8, 138.1, 152.6, 158.1;

^{19}F NMR (376 MHz, $CDCl_3$) δ -83.2;

IR ν_{max} (film, cm^{-1}): 1764, 1494, 1265, 1203, 742; **$[\alpha]^{22}_D$** = 66.8 (*c* = 2.6 in $CHCl_3$);

HRMS (ESI, m/z): calcd. for $C_{16}H_{11}F_3O_3H^+$ 309.0733, found 309.0728;

HPLC analysis: 96:4 er, [CHIRALPAK IC column; 0.6 mL/min; solvent system: i-PrOH/hexane = 5:95; retention times: 8.2 min (major), 7.6 min (minor)].

(*S*)-6-methoxy-2-phenyl-2-(trifluoromethyl)-4H-benzo[*d*][1,3]dioxin-4-one (3c): light yellow oil. 98% yield, 31.7 mg

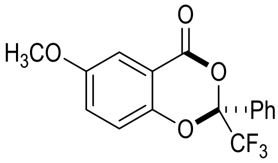

3c: 98%, 96:4 er

^{1}H NMR (400 MHz, $CDCl_3$) δ 3.73 (s, 3H), 7.09-7.16 (m, 2H), 7.25 (d, *J* = 4.8 Hz, 1H), 7.37-7.41 (m, 3H), 7.62 (d, *J* = 7.2 Hz, 2H);

^{13}C NMR (100 MHz, $CDCl_3$) δ 55.8, 101.5 (q, *J* = 35.0 Hz), 111.3, 114.1, 118.6, 120.3 (q, *J* = 282.6 Hz), 125.5, 128.2, 128.8, 131.0, 131.5, 148.7, 155.6, 158.1;

^{19}F NMR (376 MHz, $CDCl_3$) δ -83.1;

IR ν_{max} (film, cm^{-1}): 1766, 1494, 1265, 1232, 1201, 738; **$[\alpha]^{23}_D$** = 44.5 (*c* = 2.3 in $CHCl_3$);

HRMS (ESI, m/z): calcd. for $C_{16}H_{11}F_3O_4H^+$ 325.0682, found 325.0676;

HPLC analysis: 96:4 er, [CHIRALPAK IC column; 0.6 mL/min; solvent system: i-PrOH/hexane = 1:99; retention times: 12.3 min (major), 10.9 min (minor)].

(*S*)-6-ethyl-2-phenyl-2-(trifluoromethyl)-4H-benzo[*d*][1,3]dioxin-4-one (3d): yellowish oil. 95% yield, 30.4 mg

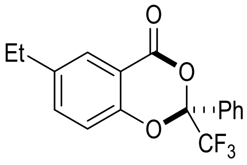

3d: 95%, 96:4 er

^{1}H NMR (400 MHz, $CDCl_3$) δ 1.16 (t, *J* = 7.6 Hz, 3H), 2.56 (q, *J* = 7.6 Hz, 2H), 7.09 (d, *J* = 7.6 Hz, 1H), 7.35-7.42 (m, 4H), 7.64 (d, *J* = 3.6 Hz, 3H);

^{13}C NMR (100 MHz, $CDCl_3$) δ 15.0, 27.8, 101.4 (q, *J* = 34.4 Hz), 113.6, 117.2, 120.3 (q, *J* = 282.9 Hz), 128.2, 128.7, 128.8, 131.0, 131.6, 137.1, 140.1, 152.7, 158.2;

^{19}F NMR (376 MHz, $CDCl_3$) δ -83.2;

IR ν_{max} (film, cm^{-1}):1766, 1496, 1265, 1234, 1203, 738; **$[\alpha]^{22}_{D}$** = 52.6 (*c* = 3.0 in $CHCl_3$);

HRMS (ESI, m/z): calcd. for $C_{17}H_{13}F_3O_3H^+$ 323.0890, found 323.0885;

HPLC analysis: 96:4 er, [CHIRALPAK IC column; 0.6 mL/min; solvent system: i-PrOH/hexane = 1:99; retention times: 9.6 min (major), 8.6 min (minor)].

(*S)*-6-chloro-2-phenyl-2-(trifluoromethyl)-4H-benzo[*d*][1,3]dioxin-4-one (3e): colorless oil. 31.4 mg, 93% yield

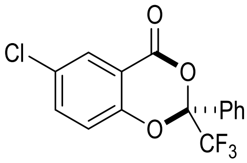

3e: 93%, 91:9 er

^{1}H NMR (400 MHz, $CDCl_3$) δ 7.08 (d, *J* = 7.6 Hz, 1H), 7.23 (s, 1H), 7.38-7.44 (m, 3H), 7.63 (d, *J* = 6.8 Hz, 2H), 7.77 (d, *J* = 8.4 Hz, 1H);

^{13}C NMR (100 MHz, $CDCl_3$) δ 101.8 (q, *J* = 34.5 Hz), 112.4, 117.8, 120.1 (q, *J* = 282.8 Hz), 124.8, 128.0, 129.0, 131.0, 131.2, 131.3, 143.4, 155.0, 157.1;

^{19}F NMR (376 MHz, $CDCl_3$) δ -83.2;

IR ν_{max} (film, cm^{-1}): 1766, 1608, 1423, 1265, 1203, 750; **$[\alpha]^{22}_{D}$** = 153.8 (*c* = 2.9 in $CHCl_3$);

HRMS (ESI, m/z): calcd. for $C_{15}H_8ClF_3O_3H^+$ 329.0187, found 329.0186;

HPLC analysis: 91:9 er, [CHIRALPAK IC column; 0.6 mL/min; solvent system: i-PrOH/hexane = 1:99; retention times: 8.3 min (major), 7.5 min (minor)].

(*S*)-6-bromo-2-phenyl-2-(trifluoromethyl)-4H-benzo[*d*][1,3]dioxin-4-one (3f): light yellow oil. 31 mg, 83% yield

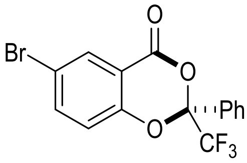

3f: 83%, 92:8 er

^{1}H NMR (400 MHz, $CDCl_3$) δ 7.09 (d, *J* = 8.8 Hz, 1H), 7.38-7.44 (m, 3H), 7.61 (d, *J* = 7.6 Hz, 2H), 6.66 (dd, *J* = 2.4, 8.8 Hz, 1H), 7.95 (d, *J* = 2.4 Hz, 1H);

^{13}C NMR (100 MHz, $CDCl_3$) δ 101.7 (q, *J* = 34.8 Hz), 115.4, 116.5, 119.2, 120.1 (q, *J* = 282.9 Hz), 128.1, 129.0, 131.0, 131.3, 132.5, 140.1, 153.6, 156.6;

^{19}F NMR (376 MHz, $CDCl_3$) δ -83.1;

IR ν_{max} (film, cm^{-1}):1774, 1473, 1265, 1236, 1203, 738; **$[\alpha]^{21}_{D}$** = 44.1 (*c* = 2.9 in $CHCl_3$);

HRMS (ESI, m/z): calcd. for $C_{15}H_8BrF_3O_3H^+$ 372.9682, found 372.8684;

HPLC analysis: 92:8 er, [CHIRALPAK IC column; 0.6 mL/min; solvent system: i-PrOH/hexane = 1:99; retention times: 8.8 min (major), 7.9 min (minor)].

(*S*)-7-methoxy-2-phenyl-2-(trifluoromethyl)-4H-benzo[*d*][1,3]dioxin-4-one (3g): colorless oil. 31.7 mg, 98% yield

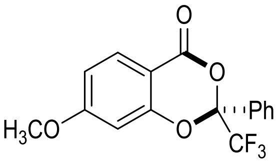

3g: 98%, 94:6 er

^{1}H NMR (400 MHz, $CDCl_3$) δ 3.86 (s, 1H), 6.60-6.64 (m, 2H), 7.38-7.41 (m, 3H), 7.64 (d, *J* = 8.0 Hz, 2H), 7.73 (d, *J* = 8.4 Hz, 1H);
^{13}C NMR (100 MHz, $CDCl_3$) δ 55.9, 101.4, 101.5 (q, *J* = 34.0 Hz), 106.5, 111.5, 120.3 (q, *J* = 282.6 Hz), 128.0, 128.8, 131.0, 131.6, 131.7, 156.5, 157.7, 166.9;
^{19}F NMR (376 MHz, $CDCl_3$) δ -83.2;
IR ν_{max} (film, cm^{-1}): 1759, 1620, 1444, 1265, 1207, 1126, 742; **$[\alpha]^{23}_D$** = 183.5 (*c* = 2.4 in $CHCl_3$);
HRMS (ESI, m/z): calcd. for $C_{16}H_{11}F_3O_4H^+$ 325.0682, found 325.0680;
HPLC analysis: 94:6 er, [CHIRALPAK IC column; 0.6 mL/min; solvent system: i-PrOH/hexane = 5:95; retention times: 13.4 min (major), 9.5 min (minor)].

(*S*)-7-bromo-2-phenyl-2-(trifluoromethyl)-4H-benzo[*d*][1,3]dioxin-4-one (3h): white powder 32.3 mg, 87% yield

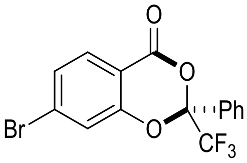

3h:87%, 91:9 er

^{1}H NMR (400 MHz, $CDCl_3$) δ 7.24 (dd, *J* = 1.6, 8.4 Hz, 1H), 7.40-7.44 (m, 4H), 7.62 (d, *J* = 8.0 Hz, 2H), 7.69 (d, *J* = 8.4 Hz, 1H);
^{13}C NMR (100 MHz, $CDCl_3$) δ 101.8 (q, *J* = 34.7 Hz), 112.8, 120.6 (q, *J* = 282.9 Hz), 120.8, 127.7, 128.0, 129.0, 131.0, 131.1, 131.3, 131.8, 154.9, 157.2;
^{19}F NMR (376 MHz, $CDCl_3$) δ -83.1;
IR ν_{max} (film, cm^{-1}):1766, 1602, 1423, 1265, 1203, 1080, 738; **$[\alpha]^{23}_D$** = 173.5 (*c* = 3.0 in $CHCl_3$);
HRMS (ESI, m/z): calcd. for $C_{15}H_8BrF_3O_3H^+$ 372.9682, found 372.9681;
HPLC analysis: 91:9 er, [CHIRALPAK IC column; 0.6 mL/min; solvent system: i-PrOH/hexane = 1:99; retention times: 9.3 min (major), 8.1 min (minor)].

Methyl (*S*)-4-oxo-2-phenyl-2-(trifluoromethyl)-4H-benzo[*d*][1,3]dioxine-7-carboxylate (3i): yellowish oil. 32.7 mg, 93% yield

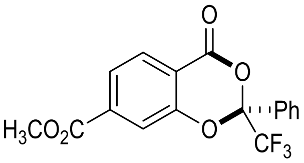

3i: 93%, 90:10 er

^{1}H NMR (400 MHz, $CDCl_3$) δ 3.95 (s, 3H), 7.37-7.43 (m, 3H), 7.63 (d, *J* = 8.0 Hz, 2H), 7.73 (d, *J* = 8.0 Hz, 1H), 7.86 (s, 1H), 7.91 (d, *J* = 8.4 Hz, 1H);
^{13}C NMR (100 MHz, $CDCl_3$) δ 52.9, 101.8 (q, *J* = 34.6 Hz), 117.2, 118.7, 120.6 (q, *J* = 282.6 Hz), 124.5, 128.2, 129.0, 130.3, 130.9, 131.3, 138.1, 154.5, 157.2, 164.9;
^{19}F NMR (376 MHz, $CDCl_3$) δ -83.1;
IR ν_{max} (film, cm^{-1}): 1768, 1730, 1429, 1265, 1203, 738; **$[\alpha]^{22}_D$** = 155.1 (*c* = 2.1 in $CHCl_3$);
HRMS (ESI, m/z): calcd. for $C_{17}H_{11}F_3O_5H^+$ 353.0631, found 353.0628;
HPLC analysis: 90:10 er, [CHIRALPAK IC column; 0.6 mL/min; solvent system: i-PrOH/hexane = 10:90; retention times: 12.0 min (major), 8.9 min (minor)].

(*S*)-8-ethoxy-2-phenyl-2-(trifluoromethyl)-4H-benzo[*d*][1,3]dioxin-4-one (3j): yellowish oil. 29.4 mg, 87% yield

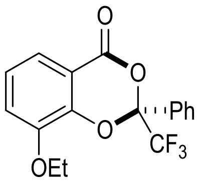

3j: 87%, 97:3 er

^{1}H NMR (400 MHz, $CDCl_3$) δ 1.52 (t, *J* = 7.6 Hz, 3H), 4.18 (q, *J* = 7.6 Hz, 2H), 6.98 (t, *J* = 8.0 Hz, 1H), 7.11 (d, *J* = 8.4 Hz, 1H), 7.34-7.40 (m, 4H), 7.66 (d, *J* = 6.8 Hz, 2H);

^{13}C NMR (100 MHz, $CDCl_3$) δ 14.7, 65.2, 101.8 (q, *J* = 34.4 Hz), 115.0, 120.0, 120.3 (q, *J* = 282.7 Hz), 120.7, 123.7, 127.9, 128.8, 131.1, 131.5, 144.8, 147.7, 158.0;

^{19}F NMR (376 MHz, $CDCl_3$) δ -83.1;

IR ν_{max} (film, cm^{-1}): 1755, 1593, 1475, 1230, 1136, 746; **$[\alpha]^{23}_{D}$** = 204.8 (*c* = 2.9 in $CHCl_3$);

HRMS (ESI, m/z): calcd. for $C_{17}H_{13}F_3O_4H^+$ 339.0839, found 339.0833;

HPLC analysis: 97:3 er, [CHIRALPAK IC column; 0.6 mL/min; solvent system: i-PrOH/hexane = 5:95; retention times: 8.9 min (major), 8.3 min (minor)].

(*S*)-8-methyl-2-phenyl-2-(trifluoromethyl)-4H-benzo[*d*][1,3]dioxin-4-one (3k): yellowish oil. 26.5 mg, 86% yield

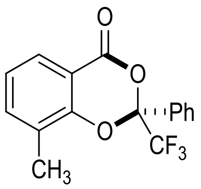

3k: 86%, 93:7 er

^{1}H NMR (400 MHz, $CDCl_3$) δ 2.43 (s, 3H), 6.98 (t, *J* = 7.6 Hz, 1H), 7.35-7.42 (m, 4H), 7.64 (d, *J* = 8.0 Hz, 2H), 7.67 (d, *J* = 8.0 Hz, 1H);

^{13}C NMR (100 MHz, $CDCl_3$) δ 14.9, 101.5 (q, *J* = 34.1 Hz), 113.7, 120.8 (q, *J* = 282.7 Hz), 123.4, 126.6, 127.6, 127.7, 128.9, 131.1, 131.6, 138.2, 152.8, 158.2;

^{19}F NMR (376 MHz, $CDCl_3$) δ -83.1;

IR ν_{max} (film, cm^{-1}):1759, 1483, 1185, 1201, 1132, 750; **$[\alpha]^{21}_{D}$** = 94.4 (*c* = 2.5 in $CHCl_3$);

HRMS (ESI, m/z): calcd. for $C_{16}H_{11}F_3O_3H^+$ 309.0733, found 309.0732;

HPLC analysis: 93:7 er, [CHIRALPAK IC column; 0.6 mL/min; solvent system: i-PrOH/hexane = 1:99; retention times: 10.0 min (major), 8.6 min (minor)].

(*S*)-5,7-dimethyl-2-phenyl-2-(trifluoromethyl)-4H-benzo[d][1,3]dioxin-4-one (3l): yellowish oil. 26.9 mg, 84% yield

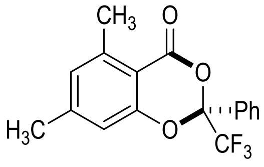

3l: 84%, 98:2 er

^{1}H NMR (400 MHz, $CDCl_3$) δ 2.31 (s, 3H), 2.50 (s, 3H), 6.69 (s, 1H), 6.83 (s, 1H), 7.37 (m, 3H), 7.62 (d, *J* = 6.8 Hz, 2H);

^{13}C NMR (100 MHz, $CDCl_3$) δ 21.7, 21.8, 100.6 (q, *J* = 33.9 Hz), 109.9, 115.5, 120.4 (q, *J* = 282.9 Hz), 128.1, 128.7, 130.8, 131.8, 144.0, 147.7, 155.5, 157.4;

^{19}F NMR (376 MHz, $CDCl_3$) δ -83.3;

IR ν_{max} (film, cm^{-1}):1759, 1484, 1185, 1203, 1132, 750; **$[\alpha]^{21}_{D}$** = 92.7 (*c* = 2.5 in $CHCl_3$);

HRMS (ESI, m/z): calcd. for $C_{17}H_{13}F_3O_3H^+$ 323.0890, found 323.0888;

HPLC analysis: 98:2 er, [CHIRALPAK IC column; 0.6 mL/min; solvent system: i-PrOH/hexane = 5:95; retention times: 8.3 min (major), 7.7 min (minor)].

(*S*)-6-phenyl-6-(trifluoromethyl)-8H-[1,3]dioxolo[4',5':4,5]benzo[1,2-*d*][1,3]dioxin-8-one (3m): yellowish oil. 30.7 mg, 91% yield

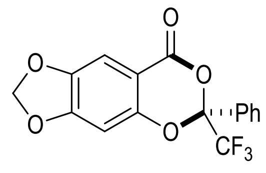

3m: 91%, 97:3 er

^{1}H NMR (400 MHz, $CDCl_3$) δ 5.97 (s, 1H), 6.02 (s, 1H), 6.63 (s, 1H), 7.12 (s, 1H), 7.37-7.42 (m, 3H), 7.62 (d, *J* = 6.8 Hz, 2H);
^{13}C NMR (100 MHz, $CDCl_3$) δ 98.4, 101.4 (q, *J* = 37.0 Hz), 102.7, 106.5, 107.0, 120.7 (q, *J* = 286.1 Hz), 128.0, 128.8, 131.0, 131.4, 144.4, 152.1, 155.1, 157.6;
^{19}F NMR (376 MHz, $CDCl_3$) δ -83.1;
IR ν_{max} (film, cm^{-1}): 1751, 1463, 1265, 1207, 738; **$[\alpha]^{23}_{D}$** = 120.6 (*c* = 2.3 in $CHCl_3$);
HRMS (ESI, m/z): calcd. for $C_{16}H_9F_3O_5H^+$ 339.0475, found 339.0475;
HPLC analysis: 97:3 er, [CHIRALPAK IC column; 0.6 mL/min; solvent system: i-PrOH/hexane = 5:95; retention times: 16.6 min (major), 12.2 min (minor)].

(*S*)-3-phenyl-3-(trifluoromethyl)-1H-naphtho[2,1-*d*][1,3]dioxin-1-one (3n): yellowish oil. 31.6 mg, 92% yield

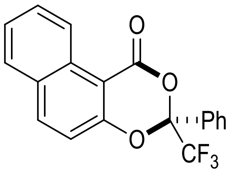

3n: 92%, 98:2 er

^{1}H NMR (400 MHz, $CDCl_3$) δ 7.30-7.37 (m, 4H), 7.46 (t, *J* = 7.6 Hz, 1H), 7.61-7.78 (m, 4H), 8.06 (d, *J* = 8.8 Hz, 1H), 9.05 (d, *J* = 8.4 Hz, 1H);
^{13}C NMR (100 MHz, $CDCl_3$) δ 101.0 (q, *J* = 33.6 Hz), 106.4, 116.7, 120.3 (q, *J* = 283.9 Hz), 125.6, 126.0, 128.0, 128.8, 130.1, 131.1, 131.3, 131.4, 139.0, 156.3, 157.2;
^{19}F NMR (376 MHz, $CDCl_3$) δ -83.2;
IR ν_{max} (film, cm^{-1}):1751, 1517, 1265, 1203, 1130, 734; **$[\alpha]^{21}_{D}$** = -54.4 (*c* = 1.5 in $CHCl_3$);
HRMS (ESI, m/z): calcd. for $C_{19}H_{11}F_3O_3H^+$ 345.0733, found 345.0731;
HPLC analysis: 98:2 er, [CHIRALPAK IC column; 0.6 mL/min; solvent system: i-PrOH/hexane = 5:95; retention times: 8.7 min (major), 8.3 min (minor)].

(*S*)-2-phenyl-2-(trifluoromethyl)-4H-naphtho[1,2-*d*][1,3]dioxin-4-one (3o): white powder. 32.2 mg, 94% yield

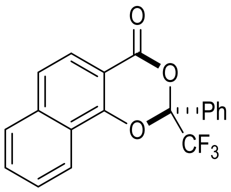

3o: 94%, 95:5 er

^{1}H NMR (400 MHz, $CDCl_3$) δ 7.29-7.37 (m, 3H), 7.48 (d, *J* = 8.4 Hz, 1H), 7.69-7.73 (m, 5H), 8.82 (d, *J* = 7.6 Hz, 1H), 8.44 (d, *J* = 6.4 Hz, 1H);
^{13}C NMR (100 MHz, $CDCl_3$) δ 101.9 (q, *J* = 34.5 Hz), 108.6, 120.4 (q, *J* = 282.7 Hz), 122.5, 123.1, 123.2, 124.7, 127.5, 127.6, 128.3, 128.9, 130.5, 131.2, 131.4, 137.7, 152.9, 158.1;
^{19}F NMR (376 MHz, $CDCl_3$) δ -83.0;
IR ν_{max} (film, cm^{-1}):1759, 1635, 1388, 1265, 1064, 738; **$[\alpha]^{23}_{D}$** = 338.3 (*c* = 2.0 in $CHCl_3$);
HRMS (ESI, m/z): calcd. for $C_{19}H_{11}F_3O_3H^+$ 345.0733, found 345.0730;
HPLC analysis: 95:5 er, [CHIRALPAK IC column; 0.6 mL/min; solvent system: i-PrOH/hexane = 5:95; retention times: 9.7 min (major), 8.3 min (minor)].

(*S*)-2-phenyl-2-(trifluoromethyl)-4H-naphtho[2,3-*d*][1,3]dioxin-4-one (3p): colorless oil. 29.9 mg, 87% yield

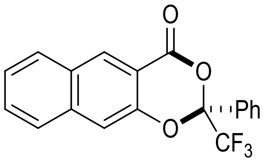

3p: 87%, 94:6 er

^{1}H NMR (400 MHz, $CDCl_3$) δ 7.35-7.38 (m, 3H), 7.43 (t, *J* = 8.0 Hz, 1H), 7.56-7.81 (m, 6H), 8.47 (s, 1H);

^{13}C NMR (100 MHz, $CDCl_3$) δ 101.5 (q, *J* = 34.4 Hz), 113.6, 113.8, 120.9 (q, *J* = 282.7 Hz), 126.2, 127.1, 128.4, 128.9, 129.4, 129.8, 130.2, 131.0, 131.7, 133.1, 137.7, 149.6, 158.5;

^{19}F NMR (376 MHz, $CDCl_3$) δ -83.1;

IR ν_{max} (film, cm^{-1}):1766, 1639, 1265, 1207, 732; **$[\alpha]^{23}_{D}$** = 258.2 (*c* = 2.0 in $CHCl_3$);

HRMS (ESI, m/z): calcd. for $C_{19}H_{11}F_3O_3H^+$ 345.0733, found 345.0733;

HPLC analysis: 94:6 er, [CHIRALPAK IC column; 0.6 mL/min; solvent system: i-PrOH/hexane = 1:99; retention times: 20.2 min (major), 12.3 min (minor)].

(*S*)-6-methyl-2-(p-tolyl)-2-(trifluoromethyl)-4H-benzo[*d*][1,3]dioxin-4-one (3q): colorless oil. 99% yield, 31.7 mg

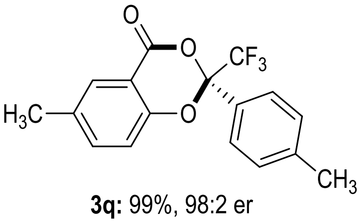

3q: 99%, 98:2 er

^{1}H NMR (400 MHz, $CDCl_3$) δ 2.25 (s, 3H), 2.30 (s, 3H), 7.05 (d, *J* = 8.4 Hz, 1H), 7.16 (d, *J* = 8.0 Hz, 2H), 7.36 (dd, *J* = 2.0, 8.4 Hz, 1H), 7.50 (d, *J* = 8.0 Hz, 2H), 7.61 (s, 1H);

^{13}C NMR (100 MHz, $CDCl_3$) δ 20.5, 21.2, 101.5 (q, *J* = 34.3 Hz), 113.7, 117.1, 120.3 (q, *J* = 282.5 Hz), 128.1, 128.5, 129.5, 129.8, 133.7, 138.0, 141.3, 152.6, 158.3;

^{19}F NMR (376 MHz, $CDCl_3$) δ -83.3;

IR ν_{max} (film, cm^{-1}):1762, 1494, 1265, 1184, 736; **$[\alpha]^{23}_{D}$** = 63.8 (*c* = 2.7 in $CHCl_3$);

HRMS (ESI, m/z): calcd. for $C_{17}H_{13}F_3O_3H^+$ 323.0890, found 323.0888;

HPLC analysis: 98:2 er, [CHIRALPAK IC column; 0.6 mL/min; solvent system: i-PrOH/hexane = 1:99; retention times: 9.8 min (major), 9.1 min (minor)].

(*S*)-2-(4-methoxyphenyl)-6-methyl-2-(trifluoromethyl)-4H-benzo[*d*][1,3]dioxin-4-one (3r): yellowish oil. 99% yield, 33.5 mg

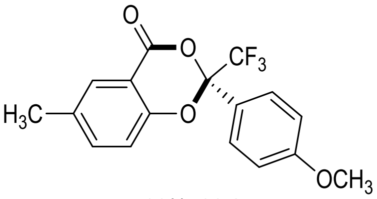

3r: 99%, 98:2 er

^{1}H NMR (400 MHz, $CDCl_3$) δ 2.26 (s, 3H), 3.76 (s, 3H), 6.85 (d, *J* = 6.8 Hz, 2H), 7.05 (d, *J* = 8.4 Hz, 1H), 7.36 (dd, *J* = 2.0, 8.4 Hz, 1H), 7.53 (d, *J* = 8.4 Hz, 2H), 7.61 (s, 1H);

^{13}C NMR (100 MHz, $CDCl_3$) δ 20.4, 55.3, 101.5 (q, *J* = 34.2 Hz), 113.7, 114.2, 117.1, 120.3 (q, *J* = 282.5 Hz), 123.2, 129.7, 129.8, 133.7, 138.0, 152.6, 158.3, 161.5;

^{19}F NMR (376 MHz, $CDCl_3$) δ -83.5;

IR ν_{max} (film, cm^{-1}): 1762, 1494, 1265, 1203, 1087, 740; **$[\alpha]^{21}_{D}$** = 62.8 (*c* = 1.7 in $CHCl_3$);

HRMS (ESI, m/z): calcd. for $C_{17}H_{13}F_3O_4H^+$ 339.0839, found 339.0834;

HPLC analysis: 98:2 er, [CHIRALPAK IC column; 0.6 mL/min; solvent system: i-PrOH/hexane = 5:95; retention times: 10.3 min (major), 9.6 min (minor)].

(*S*)-2-(4-(dimethylamino)phenyl)-6-methyl-2-(trifluoromethyl)-4H-benzo[*d*][1,3]dioxin-4-one (3s): yellowish oil. 96% yield, 33.6 mg

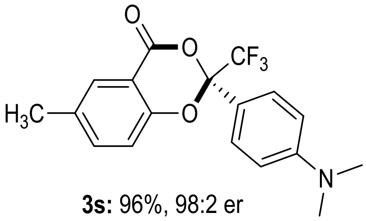

3s: 96%, 98:2 er

^{1}H NMR (400 MHz, $CDCl_3$) δ 2.25 (s, 3H), 2.93 (s, 6H), 6.59 (d, *J* = 8.8 Hz, 2H), 7.03 (d, *J* = 8.4 Hz, 1H), 7.34 (d, *J* = 8.4 Hz, 1H), 7.42 (d, *J* = 8.4 Hz, 2H), 7.62 (s, 1H);

^{13}C NMR (100 MHz, $CDCl_3$) δ 20.5, 39.9, 102.1 (q, *J* = 32.3 Hz), 111.4, 113.9, 117.1, 117.4, 120.5 (q, *J* = 281.3 Hz), 129.2, 129.8, 133.4, 137.8, 151.6, 152.8, 158.8;

^{19}F NMR (376 MHz, $CDCl_3$) δ 83.6;

IR ν_{max} (film, cm^{-1}): 1762, 1612, 1265, 1184, 894, 738; **$[\alpha]^{22}_D$** = 60.1 (*c* = 1.3 in $CHCl_3$);

HRMS (ESI, m/z): calcd. for $C_{18}H_{16}F_3NO_3H^+$ 352.1155, found 352.1154;

HPLC analysis: 98:2 er, [CHIRALPAK ID column; 0.5 mL/min; solvent system: i-PrOH/hexane = 0.5:99.5; retention times: 21.8 min (major), 22.8 min (minor)].

(*S*)-6-methyl-2-(trifluoromethyl)-2-(4-(trifluoromethyl)phenyl)-4H-benzo[*d*][1,3]dioxin-4-one (3t): colorless oil. 98% yield, 36.8 mg

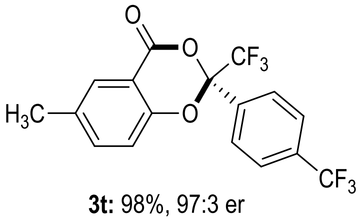

3t: 98%, 97:3 er

^{1}H NMR (400 MHz, $CDCl_3$) δ 2.28 (s, 3H), 7.10 (d, *J* = 8.4 Hz, 1H), 7.41 (d, *J* = 8.8 Hz, 1H), 7.65 (d, *J* = 8.0 Hz, 3H), 7.79 (d, *J* = 8.4 Hz, 2H);

^{13}C NMR (100 MHz, $CDCl_3$) δ 20.4, 100.7 (q, *J* = 34.6 Hz), 113.3, 117.0, 120.1 (q, *J* = 283.2 Hz), 123.3 (q, *J* = 271.1 Hz), 125.8, 128.7, 130.0, 133.2 (q, *J* = 32.8 Hz), 134.3, 135.6, 138.4, 152.3, 157.5;

^{19}F NMR (376 MHz, $CDCl_3$) δ -63.2, -82.9;

IR ν_{max} (film, cm^{-1}):1766, 1327, 1265, 1136, 740; **$[\alpha]^{22}_D$** = 52.8 (*c* = 3.2 in $CHCl_3$);

HRMS (ESI, m/z): calcd. for $C_{17}H_{10}F_6O_3H^+$ 377.0607, found 377.0601;

HPLC analysis: 97:3 er, [CHIRALPAK IC column; 0.6 mL/min; solvent system: i-PrOH/hexane = 1:99; retention times: 9.2 min (major), 8.6 min (minor)].

(*S*)-2-(4-chlorophenyl)-6-methyl-2-(trifluoromethyl)-4H-benzo[*d*][1,3]dioxin-4-one (3u): colorless oil. 99% yield, 33.7 mg

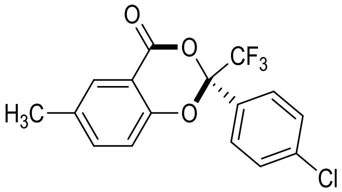

3u: 99%, 96:4 er

^{1}H NMR (400 MHz, $CDCl_3$) δ 2.28 (s, 3H), 7.06 (d, *J* = 8.4 Hz, 1H), 7.34-7.41 (m, 3H), 7.57 (d, *J* = 8.4 Hz, 2H), 7.63 (s, 1H);

^{13}C NMR (100 MHz, $CDCl_3$) δ 20.5, 100.7 (q, *J* = 37.9 Hz), 113.4, 117.1, 120.1 (q, *J* = 283.8 Hz), 129.2, 129.6, 129.9, 130.2, 134.1, 137.5, 138.3, 152.3, 157.8;

^{19}F NMR (376 MHz, $CDCl_3$) δ -83.2;

IR ν_{max} (film, cm^{-1}):1766, 1494, 1265, 1199, 1087, 819; **$[\alpha]^{23}_D$** = 54.2 (*c* = 2.6 in $CHCl_3$);

HRMS (ESI, m/z): calcd. for $C_{17}H_{10}ClF_3O_3H^+$ 343.0343, found 343.0340;

HPLC analysis: 96:4 er, [CHIRALPAK IC column; 0.6 mL/min; solvent system: i-PrOH/hexane = 1:99; retention times: 9.1 min (major), 8.4 min (minor)].

(*S*)-2-(4-bromophenyl)-6-methyl-2-(trifluoromethyl)-4H-benzo[*d*][1,3]dioxin-4-one (3v):

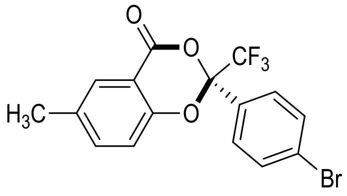

3v: 99%, 95:5 er

colorless oil. 99% yield, 38.1 mg

^{1}H NMR (400 MHz, $CDCl_3$) δ 2.28 (s, 3H), 7.06 (d, *J* = 8.4 Hz, 1H), 7.39 (dd, *J* = 2.0, 8.4 Hz, 1H), 7.49-7.53 (m, 4H), 7.63 (d, *J* = 2.0 Hz, 1H);

^{13}C NMR (100 MHz, $CDCl_3$) δ 20.5, 101.1 (q, *J* = 34.5 Hz), 113.4, 117.1, 120.1 (q, *J* = 282.8 Hz), 125.9, 129.7, 129.9, 130.7, 132.2, 134.1, 138.3, 152.3, 157.7;

^{19}F NMR (376 MHz, $CDCl_3$) δ -83.2;

IR ν_{max} (film, cm^{-1}):1766, 1622, 1496, 1288, 1203, 704; **$[\alpha]^{22}_{D}$** = 45.6 (*c* = 3.2 in $CHCl_3$);

HRMS (ESI, m/z): calcd. for $C_{16}H_{10}BrF_3O_3H^+$ 386.9838, found 386.9838;

HPLC analysis: 95:5 er, [CHIRALPAK IC column; 0.6 mL/min; solvent system: i-PrOH/hexane = 2:98; retention times: 8.1 min (major), 7.7 min (minor)].

(*S*)-2-(3-bromophenyl)-6-methyl-2-(trifluoromethyl)-4H-benzo[*d*][1,3]dioxin-4-one (3w):

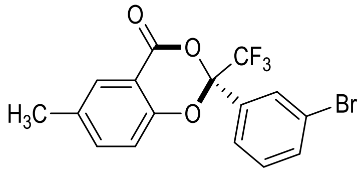

3w: 96%, 93:7 er

colorless oil. 96% yield, 37.1 mg

^{1}H NMR (400 MHz, $CDCl_3$) δ 2.28 (s, 3H), 7.08 (d, *J* = 8.4 Hz, 1H), 7.26 (t, *J* = 8.4 Hz, 1H), 7.40 (dd, *J* = 2.0, 8.4 Hz, 1H), 7.53-7.76 (m, 2H), 7.64 (s, 1H), 7.77 (s, 1H);

^{13}C NMR (100 MHz, $CDCl_3$) δ 20.5, 100.5 (q, *J* = 34.5 Hz), 113.3, 117.1, 120.1 (q, *J* = 283.3 Hz), 123.1, 126.9, 130.0, 130.3, 131.1, 133.9, 134.2, 134.3, 138.4, 152.3, 157.6;

^{19}F NMR (376 MHz, $CDCl_3$) δ -83.0;

IR ν_{max} (film, cm^{-1}):1766, 1265, 1203, 738; **$[\alpha]^{23}_{D}$** = 53.8 (*c* = 2.4 in $CHCl_3$);

HRMS (ESI, m/z): calcd. for $C_{16}H_{10}BrF_3O_3H^+$ 386.9838, found 386.9836;

HPLC analysis: 93:7 er, [CHIRALPAK IC column; 0.6 mL/min; solvent system: i-PrOH/hexane = 2:98; retention times: 8.3 min (major), 7.7 min (minor)].

(*S*)-6-methyl-2-(trifluoromethyl)-2-(3-(trifluoromethyl)phenyl)-4H-benzo[*d*][1,3]dioxin-4-one (3x): white powder. 94% yield, 35.5 mg

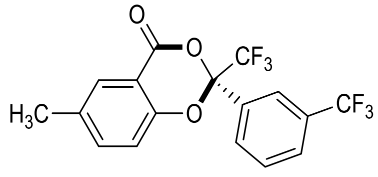

3x: 94%, 96:4 er

^{1}H NMR (400 MHz, $CDCl_3$) δ 2.29 (s, 3H), 7.11 (d, *J* = 8.4 Hz, 1H), 7.41 (dd, *J* = 1.6, 8.4 Hz, 1H), 7.55 (t, *J* = 8.0 Hz, 1H), 7.65 (s, 1H), 7.69 (d, *J* = 8.0 Hz, 1H), 7.87 (d, *J* = 8.4 Hz, 1H), 7.89 (s, 1H);

^{13}C NMR (100 MHz, $CDCl_3$) δ 20.4, 100.7 (q, *J* = 34.4 Hz), 113.2, 117.0, 120.1 (q, *J* = 283.3 Hz), 123.4 (q, *J* = 270.7 Hz), 127.9, 128.0, 129.6, 130.0, 131.4, 131.5 (q, *J* = 32.8 Hz), 133.0, 134.3, 138.5, 152.2, 157.4;

^{19}F NMR (376 MHz, $CDCl_3$) δ -62.9, -83.0;

IR ν_{max} (film, cm^{-1}): 1766, 1494, 1265, 1203, 738; **$[\alpha]^{23}_{D}$** = 42.2 (*c* = 2.2 in $CHCl_3$);

HRMS (ESI, m/z): calcd. for $C_{17}H_{10}F_6O_3H^+$ 377.0607, found 377.0601;

HPLC analysis: 96:4 er, [CHIRALPAK IC column; 0.6 mL/min; solvent system: i-PrOH/hexane = 1:99; retention times: 7.7 min (major), 7.1 min (minor)].

(*R*)-6-methyl-2-(thiophen-2-yl)-2-(trifluoromethyl)-4H-benzo[*d*][1,3]dioxin-4-one (3y): yellowish oil. 75% yield, 23.5 mg

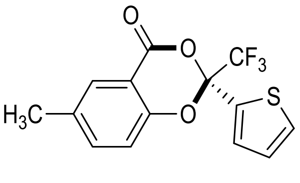

3y: 75%y, 89:11 er

^{1}H NMR (400 MHz, $CDCl_3$) δ 2.31 (s, 3H), 6.98 (dd, *J* = 3.6, 4.8 Hz, 1H), 7.05 (d, *J* = 8.4 Hz, 1H), 7.38-7.41 (m, 3H), 7.67 (d, *J* = 1.6 Hz, 1H);

^{13}C NMR (100 MHz, $CDCl_3$) δ 20.5, 100.3 (q, *J* = 35.6 Hz), 113.2, 117.2, 120.1 (q, *J* = 283.3 Hz), 127.4, 129.8, 129.9, 131.4, 133.8, 134.1, 138.4, 152.5, 157.7;

^{19}F NMR (376 MHz, $CDCl_3$) δ -83.5;

IR ν_{max} (film, cm^{-1}):1766, 1494, 1265, 1108, 734; **$[\alpha]^{23}_D$** = 46.7 (*c* = 1.7 in $CHCl_3$);

HRMS (ESI, m/z): calcd. for $C_{14}H_9F_3O_3SH^+$ 315.0297, found 315.0296;

HPLC analysis: 89:11 er, [CHIRALPAK IC column; 0.6 mL/min; solvent system: i-PrOH/hexane = 1:99; retention times: 11.8 min (major), 10.0 min (minor)].

(*S*)-2-benzyl-6-methyl-2-(trifluoromethyl)-4H-benzo[*d*][1,3]dioxin-4-one (3z): yellowish oil. 64% yield, 20.6 mg

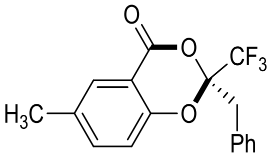

3z: 64%, 90:10 er

^{1}H NMR (400 MHz, $CDCl_3$) δ 2.27 (s, 3H), 3.38 (d, *J* = 3.2 Hz, 2H), 7.88 (d, *J* = 8.4 Hz, 1H), 7.23-7.33 (m, 6H), 7.58 (d, *J* = 1.6 Hz, 1H);

^{13}C NMR (100 MHz, $CDCl_3$) δ 20.4, 39.5, 101.8 (q, *J* = 31.9 Hz), 111.1, 115.9, 121.8 (q, *J* = 284.3 Hz), 127.7, 128.3, 129.2, 130.8, 131.2, 133.1, 137.7, 153.1, 157.6;

^{19}F NMR (376 MHz, $CDCl_3$) δ -81.0;

IR ν_{max} (film, cm^{-1}):1766, 1496, 1265, 1172, 746; **$[\alpha]^{22}_D$** = 9.2 (*c* = 1.4 in $CHCl_3$);

HRMS (ESI, m/z): calcd. for $C_{17}H_{13}F_3O_3H^+$ 323.0890, found 323.0886;

HPLC analysis: 90:10 er, [CHIRALPAK IC column; 0.5 mL/min; solvent system: i-PrOH/hexane = 1:99; retention times: 12.4 min (major), 21.5 min (minor)].

(*S*)-2-ethyl-6-methyl-2-(trifluoromethyl)-4H-benzo[*d*][1,3]dioxin-4-one (3za): colorless oil. 81% yield, 21.1 mg

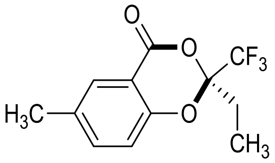

3za: 81%, 82:18 er

^{1}H NMR (400 MHz, $CDCl_3$) δ 1.14 (t, *J* = 7.6 Hz, 3H), 2.14 (q, *J* = 7.6 Hz, 2H), 2.35 (s, 3H), 6.94 (d, *J* = 8.4 Hz, 1H), 7.38 (d, *J* = 8.4 Hz, 1H), 7.74 (s, 1H);

^{13}C NMR (100 MHz, $CDCl_3$) δ 5.8, 20.5, 26.8, 102.7 (q, *J* = 32.2 Hz), 111.5, 116.0, 121.9 (q, *J* = 288.9 Hz), 129.4, 133.3, 137.8, 153.3, 158.2;

^{19}F NMR (376 MHz, $CDCl_3$) δ -80.8;

IR ν_{max} (film, cm^{-1}):1761, 1496, 1265, 1188, 738; **$[\alpha]^{23}_D$** = -5.3 (*c* = 2.2 in $CHCl_3$);

HRMS (ESI, m/z): calcd. for $C_{12}H_{11}F_3O_3H^+$ 261.0733, found 261.0730;

HPLC analysis**:** 82:18 er, [CHIRALPAK OD-H column; 0.5 mL/min; solvent system: i-PrOH/hexane = 1:99; retention times: 9.6 min (major), 9.0 min (minor)].

Supplementary References

1. Frisch, M. J., Trucks, G. W., Schlegel, H. B., Scuseria, G. E., Robb, M. A., Cheeseman, J. R., Scalmani, G., Barone, V., Mennucci, B., Petersson, G. A., Nakatsuji, H., Caricato, M., Li, X., Hratchian, H. P., Izmaylov, A. F., Bloino, J., Zheng, G., Sonnenberg, J. L., Hada, M., Ehara, M., Toyota, K., Fukuda, R., Hasegawa, J., Ishida, M., Nakajima, T., Honda, Y., Kitao, O., Nakai, H., Vreven, T., Montgomery, Jr. J. A., Peralta, J. E., Ogliaro, F., Bearpark, M., Heyd, J. J., Brothers, E., Kudin, K. N., Staroverov, V. N., Keith, T., Kobayashi, R., Normand, J., Raghavachari, K., Rendell, A., Burant, J. C., Iyengar, S. S., Tomasi, J., Cossi, M., Rega, N., Millam, J. M., Klene, M., Knox, J. E., Cross, J. B., Bakken, V., Adamo, C., Jaramillo, J., Gomperts, R., Stratmann, R. E., Yazyev, O., Austin, A. J., Cammi, R., Pomelli, C., Ochterski, J. W., Martin, R. L., Morokuma, K., Zakrzewski, V. G., Voth, G. A., Salvador, P., Dannenberg, J. J., Dapprich, S., Daniels, A. D., Farkas, O., Foresman, J. B., Ortiz, J. V., Cioslowski, J. & Fox, D. J. *Gaussian 09,* Gaussian, Inc., (2009).

2. Becke, A. D. Density-functional thermochemistry. III. The role of exact exchange. *J. Chem. Phys.* **98**, 5648–5652 (1993).

3. Lee, C., Yang, W. & Parr, R. G. Development of the Colle-Salvetti correlation-energy formula into a functional of the electron density. *Phys. Rev. B* **37**, 785–789 (1988).

4. Vosko, S. H., Wilk, L. & Nusair, M. Accurate spin-dependent electron liquid correlation energies for local spin density calculations: a critical analysis. *Can. J. Phys.* **58**, 1200–1211 (1980).

5. Krishnan, R., Binkley, J. S., Seeger, R. & Pople, J. A. Self-consistent molecular orbital methods. XX. A basis set for correlated wave functions. *J. Chem. Phys.* **72**, 650-654 (1980).

6. MacLean, A. D. & Chandler, G. S. Contracted Gaussian basis sets for molecular calculations. I. Second row atoms, Z=11–18. *J. Chem. Phys. 72,* 5639-5648 *(1980)*

7. Grimme, S., Ehrlich, S. & Goerigk, L. Effect of the damping function in dispersion corrected density functional theory. *J. Comput. Chem.* **32**, 1456–1465 (2011).

8. Grimme, S., Antony, J., Ehrlich, S. & Krieg, H. A consistent and accurate ab initio parametrization of density functional dispersion correction (DFT-D) for the 94 elements H-Pu. *J. Chem. Phys.* **132**, 154104 (2010).

9. Johnson, E. R. & Becke, A. D. A post-Hartree-Fock model of intermolecular interactions: Inclusion of higher-order corrections. *J. Chem. Phys.* **124**, 174104 (2006).

10. Becke, A. D. & Johnson, E. R. A density-functional model of the dispersion interaction. *J. Chem. Phys.* **123**, 154101 (2005).

11. Johnson, E. R. & Becke, A. D. A post-Hartree–Fock model of intermolecular interactions. *J. Chem. Phys.* **123**, 024101 (2005).

12. Frisch, M. J., Pople, J. A. & Binkley, J. S. Self-consistent molecular orbital methods 25. Supplementary functions for Gaussian basis sets. *J. Chem. Phys.* **80**, 3265-3269 (1984).

13. Tomasi, J., Mennucci, B. & Cammi, R. Quantum mechanical continuum solvation models. *Chem. Rev.* **105**, 2999–3094 (2005).

14. Zhao, Y. & Truhlar, D. G. The M06 suite of density functionals for main group thermochemistry, thermochemical kinetics, noncovalent interactions, excited states, and transition elements: two new functionals and systematic testing of four M06-class functionals and 12 other functionals. *Theor. Chem. Acc.* **120**, 215–241 (2008).
