# Supplementary material for: A reaction mode of carbene-catalysed aryl aldehyde activation and induced phenol OH functionalization
Source: Nat Commun. 2017 May 25;8:15598. doi: 10.1038/ncomms15598 (PMC5477515; doi:10.1038/ncomms15598)
Supplement: Supplementary data 1 — In DFT study, XYZ coordinates of optimized structures are important parameters to understand the calculation models. For XYZ coordinates of optimized structures in DFT studies on the mechanism, see Supplementary Data 1. [file ncomms15598-s2.docx]

**XYZ coordinates of optimized structures**

Conformer A

C -2.215204 -0.721469 -3.333169

N -1.073041 -1.101467 -2.768290

N -0.247889 -0.028722 -2.545901

C -0.927173 0.996878 -2.994000

N -2.153400 0.605299 -3.489748

C -3.174460 1.532475 -4.095031

C -0.535227 2.446026 -2.967142

C -0.674603 -2.427845 -2.410747

C -0.938815 -2.919851 -1.123522

C -0.527701 -4.197963 -0.759783

C 0.156874 -4.978367 -1.694441

C 0.433624 -4.508350 -2.980306

C 0.017085 -3.228633 -3.332432

Cl -1.794320 -1.935260 0.029826

H -0.734468 -4.576540 0.233739

Cl 0.674935 -6.573286 -1.247226

H 0.966352 -5.125176 -3.693692

Cl 0.353877 -2.628300 -4.931472

O -1.331600 3.189523 -3.867449

H -0.609951 2.822029 -1.936400

H 0.502915 2.557551 -3.289158

C -2.731812 2.988931 -3.767145

C -4.516300 1.427499 -3.419989

C -3.423325 3.324922 -2.419208

C -4.653792 2.445486 -2.463490

C -5.831811 2.547170 -1.726343

C -6.856101 1.623555 -1.954916

C -6.718076 0.617864 -2.918448

C -5.543130 0.515623 -3.666284

H -5.960277 3.336165 -0.990760

H -7.779159 1.697373 -1.387643

H -7.534516 -0.075015 -3.097079

H -5.452338 -0.243227 -4.440676

H -2.787295 3.072810 -1.559717

H -3.648916 4.392379 -2.341407

H -3.153119 3.625707 -4.548420

H -3.172678 1.322144 -5.167455

H -3.037659 -1.366307 -3.602695

Conformer B

C -2.229254 -0.621426 -3.046160

N -1.026357 -1.087623 -2.721536

N -0.075644 -0.098450 -2.775263

C -0.739079 0.958186 -3.150836

N -2.076202 0.671553 -3.331166

C -3.030472 1.716899 -3.808691

C -0.335475 2.394740 -3.247156

C -0.682384 -2.423638 -2.343901

C -0.655233 -2.789170 -0.992200

C -0.289544 -4.074899 -0.618793

C 0.052266 -4.993898 -1.608653

C 0.032888 -4.652618 -2.959479

C -0.333642 -3.364091 -3.321102

Cl -1.082700 -1.634422 0.236601

H -0.269014 -4.353349 0.426086

Cl 0.513283 -6.602053 -1.148311

H 0.302558 -5.375738 -3.717151

Cl -0.353806 -2.926409 -5.006304

O -1.338429 3.123261 -2.556049

H 0.609696 2.562340 -2.735051

H -0.233501 2.716815 -4.292100

C -2.635417 3.093456 -3.144314

C -4.454724 1.494787 -3.384567

C -3.689376 3.344568 -2.047296

C -4.823477 2.413052 -2.397232

C -6.116706 2.388449 -1.881964

C -7.021641 1.443034 -2.362952

C -6.652840 0.538889 -3.362765

C -5.363402 0.564291 -3.889413

H -6.423429 3.099569 -1.122165

H -8.031737 1.420204 -1.968899

H -7.378749 -0.170829 -3.743187

H -5.092049 -0.110093 -4.697131

H -3.252282 3.106198 -1.072703

H -3.994377 4.392235 -2.013402

H -2.693176 3.853781 -3.930907

H -2.901886 1.747177 -4.893057

H -3.148215 -1.183912 -3.064881

**II-1**

C -2.062072 -0.852895 -3.338027

N -0.918003 -1.193635 -2.725944

N -0.166363 -0.090551 -2.372689

C -0.909556 0.909715 -2.731165

N -2.105125 0.498939 -3.280493

C -3.029022 1.411460 -4.015936

C -0.570235 2.359918 -2.613080

C -0.470626 -2.498568 -2.367971

C -0.740225 -3.003952 -1.088977

C -0.316167 -4.274301 -0.719781

C 0.396393 -5.034532 -1.639355

C 0.705026 -4.552140 -2.904192

C 0.275487 -3.277791 -3.260978

Cl -1.596131 -2.042209 0.078843

H -0.539251 -4.657922 0.266004

Cl 0.929748 -6.637261 -1.185364

H 1.272648 -5.148901 -3.604573

Cl 0.689158 -2.660801 -4.827996

O -1.206235 3.100423 -3.637786

H -0.841589 2.733411 -1.616840

H 0.503321 2.492090 -2.739737

C -2.603961 2.886503 -3.735865

C -4.459039 1.402073 -3.517419

C -3.458378 3.344527 -2.528879

C -4.697632 2.499899 -2.680676

C -5.961958 2.709960 -2.140706

C -6.985737 1.813624 -2.446266

C -6.751247 0.731324 -3.295205

C -5.487169 0.521783 -3.844266

H -6.154539 3.563206 -1.497341

H -7.976452 1.966326 -2.030233

H -7.559098 0.049024 -3.536893

H -5.315601 -0.312356 -4.514124

H -2.958397 3.138378 -1.575511

H -3.651419 4.419278 -2.559058

H -2.903250 3.461405 -4.613323

H -2.906775 1.127762 -5.061801

C -3.189810 -1.872408 -3.579650

O -3.716655 -2.267576 -2.541318

C -3.456239 -2.257989 -4.924309

C -2.696468 -1.640532 -5.993700

C -3.006437 -2.096692 -7.326138

C -3.966730 -3.048554 -7.546106

C -4.703182 -3.637026 -6.479871

C -4.442849 -3.243460 -5.193728

O -1.827972 -0.749638 -5.760123

H -2.448266 -1.652586 -8.143994

H -4.173815 -3.367288 -8.564799

H -5.453388 -4.391411 -6.690283

H -4.976092 -3.673625 -4.351567

**II-2**

C -2.419671 -0.656799 -3.058248

N -1.193284 -1.076242 -2.663083

N -0.300769 -0.024281 -2.542348

C -0.978193 1.005086 -2.934454

N -2.267526 0.669855 -3.296839

C -3.199321 1.598184 -3.998868

C -0.496969 2.418408 -3.007115

C -0.800750 -2.376064 -2.241089

C -0.627939 -2.674292 -0.879341

C -0.255843 -3.951307 -0.471793

C -0.037278 -4.930620 -1.431005

C -0.151878 -4.658531 -2.787398

C -0.516534 -3.376716 -3.182316

Cl -0.835433 -1.462272 0.344482

H -0.138800 -4.169564 0.580646

Cl 0.419552 -6.540174 -0.919038

H 0.051519 -5.419288 -3.528122

Cl -0.568643 -3.027561 -4.887172

O -1.230641 3.149040 -3.973949

H -0.563478 2.887144 -2.016610

H 0.547344 2.437116 -3.316453

C -2.639233 3.043381 -3.852579

C -4.564084 1.735771 -3.361466

C -3.281463 3.591121 -2.553854

C -4.607334 2.870973 -2.542858

C -5.784898 3.218451 -1.891136

C -6.916387 2.422200 -2.070526

C -6.876745 1.306090 -2.906850

C -5.699213 0.958056 -3.567335

H -5.829894 4.099455 -1.258139

H -7.841176 2.681667 -1.565164

H -7.768363 0.704953 -3.048535

H -5.674305 0.100725 -4.229040

H -2.690776 3.331470 -1.668092

H -3.369047 4.679659 -2.575232

H -3.026016 3.614118 -4.698150

H -3.235141 1.264722 -5.036229

C -3.495043 -1.512428 -3.759329

O -3.467859 -1.462874 -4.986973

C -4.377361 -2.205835 -2.888086

C -4.323260 -1.786177 -1.508737

C -5.206779 -2.463909 -0.603216

C -6.072910 -3.424080 -1.065853

C -6.132016 -3.798466 -2.436067

C -5.292923 -3.186666 -3.334089

O -3.535070 -0.849189 -1.164731

H -5.180022 -2.178182 0.442938

H -6.740599 -3.914690 -0.361986

H -6.830124 -4.563239 -2.758222

H -5.304256 -3.446582 -4.388449

**II-3**

C -1.998305 -1.111576 -3.343707

N -0.923109 -1.334934 -2.575298

N -0.315996 -0.167439 -2.169964

C -1.064385 0.761220 -2.685425

N -2.111445 0.232075 -3.405347

C -3.158942 1.052328 -4.080600

C -0.871957 2.239642 -2.667495

C -0.424737 -2.598927 -2.143660

C -0.801983 -3.114605 -0.897737

C -0.334110 -4.351756 -0.473677

C 0.521652 -5.067146 -1.303264

C 0.927628 -4.573158 -2.537031

C 0.452181 -3.333677 -2.946986

Cl -1.853195 -2.212476 0.147063

H -0.639103 -4.748679 0.484591

Cl 1.108316 -6.626215 -0.777976

H 1.599644 -5.136591 -3.169224

Cl 0.979044 -2.700997 -4.482948

O -1.282789 2.747488 -3.925301

H -1.427724 2.694467 -1.837478

H 0.183009 2.479336 -2.544728

C -2.664898 2.547913 -4.214994

C -4.458045 1.151650 -3.304049

C -3.620319 3.411787 -3.352396

C -4.705810 2.468170 -2.906274

C -5.867907 2.783315 -2.206265

C -6.775345 1.769080 -1.910013

C -6.532873 0.457770 -2.324341

C -5.377212 0.134902 -3.032204

H -6.068029 3.805864 -1.900181

H -7.684232 2.002220 -1.364482

H -7.253655 -0.323212 -2.108971

H -5.234329 -0.888171 -3.361374

H -3.087928 3.861539 -2.507945

H -4.012873 4.247182 -3.938409

H -2.745920 2.825539 -5.264619

H -3.296518 0.595070 -5.059513

C -3.040898 -2.158109 -3.771838

O -3.465848 -2.779704 -2.798995

C -3.409458 -2.162722 -5.142588

C -4.722072 -2.707733 -5.549630

C -4.944726 -2.740811 -6.993589

C -4.046547 -2.240508 -7.887002

C -2.812956 -1.660348 -7.460861

C -2.519266 -1.633158 -6.124946

O -5.597597 -3.051619 -4.744463

H -5.887100 -3.172587 -7.315271

H -4.268052 -2.278423 -8.951059

H -2.106298 -1.282978 -8.192127

H -1.550320 -1.248296 -5.810164

**II-4**

C -2.423548 -0.638464 -3.057134

N -1.257706 -1.042277 -2.521743

N -0.352374 -0.006548 -2.397430

C -0.998635 1.023777 -2.853954

N -2.267824 0.689679 -3.264044

C -3.172073 1.615777 -4.022845

C -0.498881 2.430645 -2.945888

C -0.853097 -2.360748 -2.156094

C -0.709753 -2.710997 -0.807767

C -0.295556 -3.983374 -0.438373

C -0.016946 -4.911327 -1.434987

C -0.125196 -4.589075 -2.781693

C -0.530074 -3.307733 -3.137191

Cl -1.040921 -1.550183 0.450290

H -0.198525 -4.243109 0.606562

Cl 0.494909 -6.517988 -0.981362

H 0.107958 -5.315415 -3.547879

Cl -0.605770 -2.894597 -4.822062

O -1.179075 3.135036 -3.965227

H -0.602072 2.926419 -1.971571

H 0.558794 2.426891 -3.207366

C -2.596252 3.055511 -3.904072

C -4.550758 1.777361 -3.424468

C -3.284269 3.668483 -2.660173

C -4.609814 2.945034 -2.651913

C -5.800996 3.318229 -2.039552

C -6.927709 2.513442 -2.211951

C -6.869968 1.363718 -3.001247

C -5.680725 0.985968 -3.624463

H -5.860402 4.225330 -1.445771

H -7.863727 2.792890 -1.738889

H -7.758074 0.757047 -3.139839

H -5.645093 0.104447 -4.254247

H -2.719317 3.468038 -1.742107

H -3.375183 4.753548 -2.745183

H -2.932329 3.595581 -4.789992

H -3.179841 1.230345 -5.041001

C -3.568126 -1.441696 -3.701617

O -3.591360 -1.186920 -4.912380

C -4.389022 -2.255890 -2.893547

C -5.507117 -3.007604 -3.514980

C -6.238778 -3.884575 -2.599355

C -5.974703 -3.936320 -1.266390

C -4.947719 -3.132111 -0.679180

C -4.190738 -2.327975 -1.480096

O -5.827487 -2.902670 -4.703389

H -7.031062 -4.474915 -3.048516

H -6.561448 -4.589481 -0.624549

H -4.782853 -3.158070 0.392672

H -3.440556 -1.694733 -1.016188

**II-5**

C -1.888002 -0.721416 -3.749494

N -0.687298 -1.110433 -3.088017

N -0.005545 0.001283 -2.547458

C -0.803599 0.985418 -2.732713

N -2.015865 0.637034 -3.320715

C -2.872891 1.639793 -3.988455

C -0.550109 2.394472 -2.307058

C -0.352730 -2.392590 -2.605322

C -0.547975 -2.751339 -1.256661

C -0.195393 -4.004598 -0.769364

C 0.359920 -4.930776 -1.641362

C 0.588038 -4.623405 -2.974745

C 0.245304 -3.356786 -3.436368

Cl -1.251159 -1.622612 -0.132871

H -0.363812 -4.251134 0.269858

Cl 0.794822 -6.519306 -1.041505

H 1.042043 -5.342705 -3.642195

Cl 0.624300 -2.978375 -5.097800

O -1.198266 3.316987 -3.170853

H -0.879225 2.536339 -1.268815

H 0.516655 2.606529 -2.353286

C -2.568398 3.043363 -3.382227

C -4.350103 1.505100 -3.669999

C -3.512363 3.162127 -2.158610

C -4.705015 2.362353 -2.619877

C -6.021569 2.421436 -2.176924

C -6.982792 1.620952 -2.795480

C -6.633976 0.788139 -3.858499

C -5.314339 0.731717 -4.307932

H -6.304173 3.087799 -1.367386

H -8.013261 1.658256 -2.456687

H -7.394182 0.185579 -4.344653

H -5.055402 0.095913 -5.147517

H -3.068029 2.718260 -1.261143

H -3.744021 4.203779 -1.924280

H -2.881301 3.777772 -4.127081

H -2.659203 1.614374 -5.059640

C -3.112161 -1.696428 -3.547543

O -3.602419 -1.918881 -2.468379

C -3.434365 -2.222839 -4.863277

C -2.586994 -1.609666 -5.795754

C -2.683813 -1.912992 -7.154365

C -3.644495 -2.842901 -7.538715

C -4.497210 -3.465757 -6.609843

C -4.395077 -3.156032 -5.262089

O -1.715193 -0.726034 -5.273425

H -2.026667 -1.438677 -7.873736

H -3.735754 -3.096535 -8.590319

H -5.229507 -4.187811 -6.953220

H -5.038262 -3.616691 -4.519608

**II-6**

C -2.561409 -0.750609 -2.993360

N -1.189803 -1.196742 -2.881148

N -0.298894 -0.103132 -2.803762

C -1.024429 0.924602 -3.034691

N -2.376169 0.646209 -3.191264

C -3.304509 1.566712 -3.879971

C -0.539960 2.335400 -3.105447

C -0.799739 -2.392750 -2.236439

C -0.524565 -2.462233 -0.856254

C -0.108198 -3.639845 -0.245113

C 0.030209 -4.784583 -1.017486

C -0.216448 -4.768991 -2.382158

C -0.612993 -3.574999 -2.975500

Cl -0.686428 -1.055855 0.163315

H 0.095758 -3.658560 0.816627

Cl 0.539186 -6.277928 -0.253380

H -0.086002 -5.659006 -2.981999

Cl -0.843921 -3.572936 -4.704870

O -1.318589 3.094807 -4.020218

H -0.557605 2.792016 -2.106517

H 0.486491 2.355002 -3.467235

C -2.713576 3.008044 -3.807446

C -4.630114 1.761480 -3.167909

C -3.259392 3.599636 -2.482232

C -4.595228 2.909970 -2.366524

C -5.718015 3.293379 -1.641211

C -6.878067 2.522449 -1.726935

C -6.919714 1.393222 -2.544944

C -5.795847 1.010650 -3.278150

H -5.700118 4.187049 -1.024430

H -7.760514 2.813562 -1.165926

H -7.833982 0.813785 -2.620350

H -5.842752 0.145270 -3.930160

H -2.614690 3.348091 -1.633064

H -3.327978 4.689248 -2.523496

H -3.150799 3.566144 -4.637662

H -3.419252 1.232124 -4.913398

C -3.423052 -1.497795 -4.080265

O -3.269749 -1.328964 -5.267049

C -4.382772 -2.303565 -3.346188

C -4.265550 -1.963611 -1.991602

C -5.092774 -2.546383 -1.033252

C -6.030044 -3.474581 -1.475820

C -6.157175 -3.822837 -2.832425

C -5.333126 -3.232457 -3.778695

O -3.320480 -1.036221 -1.721497

H -5.001890 -2.276714 0.012049

H -6.684671 -3.944265 -0.748282

H -6.899989 -4.553810 -3.131453

H -5.407503 -3.477858 -4.832903

RC_1-A_

C -2.072958 -1.033654 -2.922412

N -0.751762 -0.804987 -2.911095

N -0.445629 0.499644 -3.231886

C -1.607923 1.053513 -3.393648

N -2.638952 0.165540 -3.177017

C -4.064059 0.463552 -3.510874

C -1.874324 2.472499 -3.777516

C 0.290771 -1.713236 -2.563134

C 0.784510 -1.749816 -1.252588

C 1.798232 -2.631647 -0.898573

C 2.325922 -3.470099 -1.873217

C 1.878304 -3.437853 -3.187268

C 0.863809 -2.549563 -3.529168

Cl 0.156232 -0.675017 -0.040383

H 2.165411 -2.659223 0.117857

Cl 3.598886 -4.586984 -1.434389

H 2.308243 -4.085532 -3.938591

Cl 0.325087 -2.474558 -5.175007

O -3.092569 2.577898 -4.482664

H -1.864759 3.109523 -2.882221

H -1.085825 2.818834 -4.444165

C -4.207008 1.984171 -3.839670

C -5.032364 0.296558 -2.358209

C -4.662833 2.660440 -2.524146

C -5.373440 1.539511 -1.810516

C -6.288953 1.619737 -0.766037

C -6.859822 0.446660 -0.273924

C -6.532610 -0.789644 -0.832375

C -5.622456 -0.872864 -1.884793

H -6.562604 2.581709 -0.343062

H -7.573898 0.496582 0.541976

H -6.993911 -1.694250 -0.450814

H -5.386081 -1.834411 -2.324680

H -3.809884 3.020825 -1.937094

H -5.298207 3.527319 -2.720131

H -5.006227 2.051660 -4.577340

H -4.276113 -0.161536 -4.376835

C -2.673599 -2.319196 -2.333311

O -2.542388 -2.380946 -1.111563

C -3.242604 -3.287568 -3.211845

C -3.336475 -3.020147 -4.634716

C -3.878425 -4.090840 -5.435820

C -4.298408 -5.265850 -4.870879

C -4.216213 -5.497390 -3.470207

C -3.692620 -4.520302 -2.665771

O -2.976928 -1.912574 -5.132305

H -3.945854 -3.924172 -6.505345

H -4.705464 -6.045628 -5.510149

H -4.554855 -6.439571 -3.053195

H -3.599594 -4.662386 -1.593752

C -3.879911 -0.473798 -7.474356

C -2.359169 -0.146349 -7.555605

C -4.440441 -1.512257 -8.378446

O -4.561501 0.206711 -6.744488

F -1.966611 0.609164 -6.530396

F -1.565167 -1.229928 -7.611703

F -2.139574 0.562308 -8.695267

C -5.796081 -1.833095 -8.210804

C -6.403522 -2.773682 -9.029580

C -5.667184 -3.399306 -10.037077

C -4.323779 -3.078857 -10.219336

C -3.707760 -2.142569 -9.394473

H -6.352315 -1.333861 -7.426806

H -7.449759 -3.021672 -8.885189

H -6.141323 -4.134356 -10.679603

H -3.751318 -3.560050 -11.005183

H -2.663624 -1.909011 -9.551164

TS2_1-A_

C -2.074477 -1.149858 -3.278365

N -0.991202 -1.308958 -2.494833

N -0.559533 -0.135476 -1.878147

C -1.425391 0.734874 -2.290813

N -2.359307 0.166959 -3.126388

C -3.434918 0.997907 -3.687170

C -1.457640 2.216286 -2.127814

C -0.240808 -2.489652 -2.235282

C -0.525089 -3.280695 -1.116775

C 0.246203 -4.393572 -0.806247

C 1.319428 -4.709932 -1.629522

C 1.636323 -3.944710 -2.744101

C 0.854514 -2.832723 -3.035835

Cl -1.868838 -2.878309 -0.084558

H 0.011689 -4.997215 0.059486

Cl 2.297895 -6.111078 -1.251566

H 2.476238 -4.200619 -3.374891

Cl 1.262691 -1.860645 -4.419025

O -1.659423 2.772858 -3.423158

H -2.238484 2.523464 -1.421977

H -0.495842 2.579775 -1.770036

C -2.870201 2.406028 -4.102077

C -4.542497 1.325548 -2.698934

C -4.018730 3.391842 -3.790588

C -4.863909 2.682708 -2.759435

C -5.875602 3.197978 -1.953125

C -6.555992 2.338936 -1.090620

C -6.239809 0.979060 -1.047241

C -5.232668 0.456137 -1.858037

H -6.131676 4.252763 -1.991408

H -7.342644 2.729573 -0.452647

H -6.788742 0.319692 -0.382702

H -5.006910 -0.603849 -1.864932

H -3.613113 4.350206 -3.457009

H -4.600413 3.589085 -4.698956

H -2.591166 2.407759 -5.154817

H -3.820658 0.469935 -4.556401

C -3.392130 -2.322605 -4.061057

O -4.372259 -2.237443 -3.330538

C -2.669482 -3.619495 -4.294804

C -1.832323 -3.767231 -5.399699

C -1.135029 -4.950252 -5.622352

C -1.294549 -6.004981 -4.727942

C -2.160032 -5.886549 -3.638214

C -2.848842 -4.696239 -3.427605

O -1.680849 -2.720943 -6.278592

H -0.492916 -5.032881 -6.491779

H -0.756333 -6.932386 -4.895195

H -2.298203 -6.722169 -2.960463

H -3.534161 -4.575348 -2.596517

C -2.888351 -1.991792 -6.514177

C -2.389294 -0.689751 -7.201347

C -3.836937 -2.780491 -7.417794

O -3.523559 -1.565085 -5.347094

F -1.730635 -0.937644 -8.344929

F -3.425551 0.119777 -7.486973

F -1.554523 -0.003030 -6.394639

C -5.206437 -2.797340 -7.150631

C -6.070184 -3.512613 -7.977708

C -5.573241 -4.210060 -9.075422

C -4.205295 -4.194247 -9.344492

C -3.339405 -3.482749 -8.520366

H -5.583101 -2.264474 -6.286866

H -7.132976 -3.526568 -7.758707

H -6.247462 -4.767556 -9.717953

H -3.811291 -4.739572 -10.196126

H -2.276249 -3.475440 -8.727487

PC_1-A_

C -1.890037 -1.124793 -2.915650

N -0.714924 -1.096536 -2.242751

N -0.236198 0.170409 -1.882545

C -1.170639 0.941252 -2.338892

N -2.164034 0.214968 -2.957619

C -3.335147 0.878764 -3.520671

C -1.263266 2.427377 -2.393583

C 0.066683 -2.230154 -1.910336

C -0.031665 -2.829997 -0.649467

C 0.737862 -3.936199 -0.306047

C 1.622823 -4.446957 -1.246286

C 1.754598 -3.881114 -2.507384

C 0.975811 -2.773707 -2.826166

Cl -1.146835 -2.195394 0.531674

H 0.644777 -4.385347 0.673084

Cl 2.599671 -5.842604 -0.830179

H 2.451380 -4.285355 -3.228569

Cl 1.149579 -2.062069 -4.407089

O -1.681636 2.783273 -3.709407

H -1.956377 2.810536 -1.633039

H -0.287108 2.883148 -2.236170

C -2.956451 2.282899 -4.128822

C -4.407618 1.194752 -2.494456

C -4.117725 3.227826 -3.725599

C -4.841393 2.514934 -2.608497

C -5.838513 2.997294 -1.762828

C -6.390637 2.141893 -0.810366

C -5.959208 0.816554 -0.709007

C -4.963667 0.331303 -1.554971

H -6.181279 4.025167 -1.839575

H -7.165058 2.507677 -0.143314

H -6.405074 0.159808 0.030971

H -4.632499 -0.699749 -1.492285

H -3.722617 4.207432 -3.442940

H -4.784889 3.392115 -4.579708

H -2.856163 2.203817 -5.210576

H -3.724027 0.222249 -4.298789

C -4.249509 -2.827740 -4.644617

O -5.127437 -2.607448 -3.861769

C -3.388497 -4.017096 -4.691713

C -2.300346 -4.048832 -5.567350

C -1.402302 -5.111290 -5.553254

C -1.613498 -6.157591 -4.662708

C -2.708460 -6.151326 -3.791811

C -3.587864 -5.079958 -3.804256

O -2.076857 -3.016711 -6.439176

H -0.559480 -5.102781 -6.234233

H -0.918881 -6.991114 -4.650345

H -2.861244 -6.974966 -3.103704

H -4.430288 -5.030308 -3.124495

C -3.219362 -2.241775 -6.750748

C -2.639456 -0.905441 -7.291423

C -4.108436 -2.929238 -7.782592

O -3.958089 -1.862955 -5.602391

F -1.905920 -1.119677 -8.393632

F -3.633767 -0.057573 -7.605728

F -1.858495 -0.302713 -6.384098

C -5.485058 -2.691979 -7.793490

C -6.288592 -3.297443 -8.756121

C -5.723221 -4.133807 -9.715534

C -4.348509 -4.362742 -9.712420

C -3.541268 -3.760044 -8.752671

H -5.925139 -2.041945 -7.047406

H -7.357976 -3.114653 -8.753672

H -6.351299 -4.605884 -10.464034

H -3.902596 -5.012211 -10.458440

H -2.472521 -3.935871 -8.752540

RC_1-A-minor_

C -1.973216 -1.131472 -2.840285

N -0.654187 -0.886490 -2.801545

N -0.352654 0.408067 -3.170144

C -1.516601 0.941352 -3.378285

N -2.545252 0.053710 -3.151694

C -3.960175 0.309070 -3.559139

C -1.790227 2.337244 -3.829184

C 0.386475 -1.762834 -2.376131

C 0.843843 -1.717975 -1.052227

C 1.855034 -2.566318 -0.618326

C 2.417680 -3.454995 -1.526486

C 2.006592 -3.505035 -2.851790

C 0.994397 -2.649072 -3.273979

Cl 0.170754 -0.582001 0.077496

H 2.192949 -2.530326 0.407962

Cl 3.687665 -4.530445 -0.987467

H 2.462865 -4.191941 -3.550969

Cl 0.503577 -2.685086 -4.936896

O -2.966596 2.374424 -4.614112

H -1.861332 3.008956 -2.963922

H -0.969129 2.684431 -4.454913

C -4.111664 1.802763 -3.990465

C -4.973669 0.200491 -2.439020

C -4.641251 2.556610 -2.747705

C -5.361495 1.469342 -1.991923

C -6.322739 1.599789 -0.994707

C -6.892228 0.450049 -0.449079

C -6.518160 -0.813513 -0.908649

C -5.562317 -0.947374 -1.914130

H -6.633085 2.581635 -0.649938

H -7.642415 0.538849 0.330279

H -6.979742 -1.700272 -0.487693

H -5.290751 -1.930332 -2.279993

H -3.826904 2.977346 -2.146477

H -5.285588 3.391558 -3.032760

H -4.869324 1.809233 -4.771842

H -4.131317 -0.376341 -4.389076

C -2.578478 -2.385450 -2.189099

O -2.492241 -2.369125 -0.961473

C -3.100269 -3.401721 -3.036357

C -3.123274 -3.191291 -4.471723

C -3.617586 -4.297851 -5.254645

C -4.056313 -5.451449 -4.661215

C -4.042648 -5.626435 -3.248999

C -3.567665 -4.613819 -2.459560

O -2.740571 -2.101352 -4.991683

H -3.635149 -4.176070 -6.331054

H -4.426940 -6.259996 -5.286646

H -4.396372 -6.554164 -2.812537

H -3.527087 -4.710546 -1.379067

C -4.029562 -0.323801 -7.710359

C -4.679760 -1.653340 -8.195046

C -2.848071 0.201826 -8.436625

O -4.554798 0.240376 -6.779612

F -5.624921 -2.061046 -7.354699

F -5.253299 -1.462387 -9.409532

F -3.777373 -2.646519 -8.332870

C -2.228345 1.344343 -7.904911

C -1.132308 1.905318 -8.544818

C -0.644147 1.338560 -9.723249

C -1.255704 0.207204 -10.258760

C -2.352144 -0.363579 -9.621083

H -2.615080 1.770137 -6.986304

H -0.655092 2.786163 -8.127963

H 0.212735 1.778714 -10.223245

H -0.878561 -0.233379 -11.175410

H -2.814526 -1.240856 -10.052901

TS1_1-A-minor_

C -2.143932 -0.817189 -3.265141

N -0.812849 -0.878007 -3.072451

N -0.241324 0.367797 -3.015719

C -1.244853 1.177723 -3.165958

N -2.439537 0.505796 -3.302356

C -3.710114 1.222997 -3.715353

C -1.153085 2.670953 -3.170843

C 0.038193 -2.012475 -2.893506

C 0.057807 -2.709142 -1.678724

C 0.897007 -3.799696 -1.491531

C 1.747907 -4.171856 -2.524516

C 1.790674 -3.469089 -3.721495

C 0.942565 -2.381829 -3.899081

Cl -0.952953 -2.205036 -0.352744

H 0.890160 -4.337247 -0.553646

Cl 2.807035 -5.543912 -2.302960

H 2.474013 -3.754997 -4.509049

Cl 1.050694 -1.481192 -5.375973

O -2.219318 3.241537 -3.893619

H -1.121513 3.037847 -2.135765

H -0.227814 2.972484 -3.660721

C -3.497010 2.751609 -3.523581

C -4.939757 0.997870 -2.868307

C -3.984022 3.049645 -2.085011

C -5.097716 2.039521 -1.946428

C -6.218083 2.081130 -1.122620

C -7.180239 1.079003 -1.246231

C -7.037376 0.065680 -2.196851

C -5.917207 0.018878 -3.023485

H -6.353229 2.887330 -0.407783

H -8.060580 1.099680 -0.611326

H -7.807852 -0.691562 -2.296321

H -5.795958 -0.755192 -3.771036

H -3.194847 2.879809 -1.342661

H -4.311073 4.085960 -1.975045

H -4.181188 3.234838 -4.222619

H -3.846836 0.913995 -4.747090

C -3.107419 -1.972250 -2.991710

O -3.888718 -1.728768 -2.091207

C -2.868626 -3.310031 -3.537729

C -2.316867 -3.568797 -4.816377

C -2.063349 -4.907713 -5.176764

C -2.367813 -5.947370 -4.317964

C -2.965143 -5.693789 -3.073792

C -3.217884 -4.389408 -2.699158

O -2.037677 -2.600256 -5.686638

H -1.650392 -5.092700 -6.161554

H -2.162000 -6.969808 -4.620361

H -3.220290 -6.512977 -2.410324

H -3.672145 -4.162660 -1.741712

C -3.329777 -1.689283 -6.251092

C -4.160910 -2.759121 -7.035391

C -2.615048 -0.750783 -7.228449

O -3.978047 -1.173878 -5.285496

F -4.800648 -3.605313 -6.209938

F -5.106784 -2.131660 -7.771788

F -3.430950 -3.508425 -7.893557

C -3.205984 0.492297 -7.470335

C -2.631763 1.395144 -8.362423

C -1.456271 1.061125 -9.030617

C -0.870459 -0.184174 -8.809405

C -1.449273 -1.088850 -7.921613

H -4.136212 0.734432 -6.969022

H -3.106494 2.355210 -8.540285

H -1.004634 1.761781 -9.726160

H 0.039685 -0.456342 -9.335619

H -0.993854 -2.054242 -7.750892

Int1_1-A-minor_

C -2.143257 -0.821659 -3.269776

N -0.814155 -0.901995 -3.068116

N -0.228257 0.336883 -2.973153

C -1.222754 1.160443 -3.105583

N -2.423734 0.506700 -3.269331

C -3.675145 1.246763 -3.698101

C -1.114950 2.651853 -3.073570

C 0.024273 -2.045397 -2.889202

C 0.036466 -2.740044 -1.673331

C 0.874320 -3.830273 -1.478974

C 1.730579 -4.205116 -2.506739

C 1.777533 -3.507381 -3.706619

C 0.930363 -2.420526 -3.890538

Cl -0.987416 -2.234852 -0.357749

H 0.862095 -4.366089 -0.540137

Cl 2.789018 -5.576117 -2.275747

H 2.463012 -3.796997 -4.490932

Cl 1.036369 -1.526904 -5.372446

O -2.152831 3.247985 -3.817194

H -1.112770 2.996463 -2.030359

H -0.171737 2.953434 -3.527675

C -3.446376 2.771273 -3.486474

C -4.926785 1.030320 -2.881085

C -3.966359 3.064581 -2.058901

C -5.094910 2.067186 -1.955612

C -6.234390 2.116776 -1.158792

C -7.205015 1.127129 -1.312438

C -7.050760 0.118312 -2.266154

C -5.911635 0.063870 -3.065934

H -6.377101 2.919801 -0.441833

H -8.100202 1.154058 -0.698855

H -7.826952 -0.629607 -2.389127

H -5.778777 -0.708002 -3.813493

H -3.198213 2.880209 -1.298189

H -4.283712 4.104024 -1.949713

H -4.105189 3.269762 -4.199075

H -3.792822 0.952051 -4.736226

C -3.121902 -1.973981 -3.061285

O -3.940862 -1.757532 -2.188952

C -2.837963 -3.322677 -3.588746

C -2.323393 -3.601313 -4.872636

C -2.051018 -4.933002 -5.222397

C -2.294127 -5.965713 -4.331937

C -2.851716 -5.699816 -3.075930

C -3.128158 -4.391918 -2.720249

O -2.075618 -2.644471 -5.787246

H -1.671507 -5.125200 -6.219267

H -2.072627 -6.987918 -4.622907

H -3.064103 -6.509560 -2.386267

H -3.562257 -4.158385 -1.755297

C -3.269740 -1.727080 -6.217351

C -4.233827 -2.698171 -6.984922

C -2.612706 -0.771714 -7.228020

O -3.861412 -1.181769 -5.200433

F -4.850451 -3.558403 -6.153227

F -5.200460 -1.989511 -7.608337

F -3.611750 -3.437249 -7.933523

C -3.229599 0.461655 -7.451892

C -2.695856 1.373023 -8.359980

C -1.534643 1.058417 -9.062387

C -0.923488 -0.177151 -8.859208

C -1.463128 -1.090931 -7.955399

H -4.146817 0.690477 -6.921671

H -3.190457 2.325694 -8.523125

H -1.114277 1.766769 -9.769622

H -0.025077 -0.435520 -9.411904

H -0.988846 -2.050923 -7.803626

TS2_1-A-minor_

C -2.282577 -1.015113 -3.431005

N -1.063006 -1.071476 -2.862400

N -0.634181 0.140966 -2.325405

C -1.635304 0.927255 -2.566276

N -2.651851 0.270549 -3.219127

C -3.887583 0.990123 -3.561549

C -1.771542 2.401491 -2.391332

C -0.176273 -2.180118 -2.759267

C -0.264576 -3.068553 -1.682072

C 0.628565 -4.123813 -1.543553

C 1.633300 -4.274466 -2.490546

C 1.768009 -3.397078 -3.558846

C 0.862026 -2.350454 -3.682583

Cl -1.493117 -2.849727 -0.467342

H 0.540594 -4.807563 -0.710834

Cl 2.764457 -5.599479 -2.328438

H 2.561710 -3.519558 -4.282704

Cl 1.046653 -1.235738 -5.005040

O -2.262306 2.921321 -3.623576

H -2.431679 2.644464 -1.550536

H -0.797277 2.855426 -2.218562

C -3.538419 2.437065 -4.070445

C -4.808002 1.236508 -2.376310

C -4.693492 3.320534 -3.550098

C -5.253605 2.559522 -2.372898

C -6.128177 3.002385 -1.384138

C -6.547374 2.105383 -0.402444

C -6.109591 0.779028 -0.419635

C -5.240466 0.329027 -1.412784

H -6.478330 4.030389 -1.374183

H -7.224856 2.438792 0.377404

H -6.457298 0.087860 0.341285

H -4.930815 -0.707322 -1.464199

H -4.323920 4.319514 -3.305946

H -5.454977 3.442942 -4.329991

H -3.454908 2.451482 -5.156602

H -4.385387 0.411050 -4.336902

C -3.611298 -2.203026 -4.069922

O -4.591821 -2.005175 -3.359504

C -2.948240 -3.546580 -4.166364

C -2.136624 -3.844477 -5.256875

C -1.515988 -5.082984 -5.380036

C -1.726119 -6.041298 -4.391803

C -2.565981 -5.770900 -3.309710

C -3.180765 -4.526718 -3.204503

O -1.923883 -2.893235 -6.229644

H -0.902232 -5.286235 -6.250200

H -1.249019 -7.011956 -4.480465

H -2.746955 -6.531664 -2.557834

H -3.852368 -4.293211 -2.387039

C -3.029072 -2.053295 -6.552475

C -4.016274 -2.888608 -7.436173

C -2.490239 -0.881316 -7.369252

O -3.744353 -1.589470 -5.445835

F -4.537026 -3.930264 -6.765944

F -5.041614 -2.129941 -7.862124

F -3.388622 -3.379792 -8.523188

C -3.260364 0.277431 -7.497649

C -2.796298 1.347644 -8.257526

C -1.559431 1.270116 -8.895370

C -0.794156 0.113097 -8.775575

C -1.259178 -0.962627 -8.021549

H -4.226525 0.330155 -7.011778

H -3.404613 2.241080 -8.357255

H -1.197397 2.105210 -9.486497

H 0.167368 0.042680 -9.274039

H -0.665290 -1.862207 -7.929910

PC_1-A-minor_

C -1.674469 -0.988608 -3.232350

N -0.744025 -1.147171 -2.260011

N -0.398096 0.003615 -1.539118

C -1.164083 0.893653 -2.083277

N -1.931974 0.347341 -3.088163

C -2.892837 1.163911 -3.820600

C -1.237769 2.368450 -1.882240

C -0.085243 -2.360027 -1.938599

C -0.586006 -3.212518 -0.947664

C 0.059564 -4.398611 -0.613985

C 1.227362 -4.732150 -1.286717

C 1.762018 -3.912411 -2.271743

C 1.100235 -2.730039 -2.584466

Cl -2.054077 -2.800789 -0.105392

H -0.343324 -5.042583 0.155636

Cl 2.048971 -6.227498 -0.881641

H 2.675254 -4.180780 -2.784644

Cl 1.783497 -1.696367 -3.811210

O -1.251209 2.973695 -3.174827

H -2.123812 2.647436 -1.297309

H -0.350639 2.735727 -1.369135

C -2.350106 2.630118 -4.025941

C -4.216330 1.356143 -3.099252

C -3.573840 3.554559 -3.803520

C -4.592988 2.699308 -3.090838

C -5.792135 3.085752 -2.495438

C -6.604478 2.111942 -1.916115

C -6.227280 0.766770 -1.938368

C -5.028272 0.375872 -2.533065

H -6.091255 4.129910 -2.478286

H -7.539264 2.401144 -1.445961

H -6.872857 0.016918 -1.493108

H -4.745918 -0.670907 -2.560783

H -3.273169 4.444506 -3.243762

H -3.962383 3.904975 -4.766872

H -1.940184 2.740388 -5.029642

H -3.046627 0.676355 -4.784665

C -4.101678 -3.134291 -4.372605

O -4.714352 -3.047985 -3.347021

C -3.033795 -4.095080 -4.673094

C -2.330933 -3.987890 -5.873596

C -1.274315 -4.843916 -6.163270

C -0.931739 -5.824054 -5.238138

C -1.632670 -5.953529 -4.034976

C -2.679176 -5.087091 -3.754646

O -2.634151 -3.007770 -6.784040

H -0.742913 -4.734564 -7.101202

H -0.111214 -6.498365 -5.460378

H -1.355646 -6.723456 -3.324049

H -3.234365 -5.148503 -2.826142

C -3.905807 -2.400142 -6.703046

C -4.929600 -3.312736 -7.458103

C -3.833481 -1.035225 -7.369943

O -4.381871 -2.224724 -5.381289

F -5.051291 -4.517883 -6.866748

F -6.146469 -2.750574 -7.483008

F -4.532973 -3.518171 -8.723556

C -4.765050 -0.049961 -7.035121

C -4.715579 1.193525 -7.659989

C -3.742088 1.457964 -8.621726

C -2.816918 0.472604 -8.958040

C -2.863330 -0.773932 -8.338769

H -5.516804 -0.252619 -6.283236

H -5.442665 1.954140 -7.395180

H -3.706842 2.427026 -9.109136

H -2.055847 0.671661 -9.705282

H -2.141429 -1.537519 -8.597151

RC_1-B_

C -2.695631 -1.966750 -2.487023

N -1.926754 -2.499602 -1.521418

N -0.850922 -1.686131 -1.194116

C -1.033313 -0.637440 -1.942977

N -2.156911 -0.753950 -2.719170

C -2.575457 0.290423 -3.652206

C -0.190865 0.578143 -2.147399

C -2.166039 -3.690769 -0.776856

C -2.812059 -3.621222 0.468125

C -3.053993 -4.772076 1.213016

C -2.633843 -5.998867 0.702738

C -1.971112 -6.098772 -0.516833

C -1.731248 -4.937539 -1.251733

Cl -3.309199 -2.087326 1.122976

H -3.559738 -4.708514 2.168117

Cl -2.937814 -7.451743 1.628795

H -1.637578 -7.057446 -0.893720

Cl -0.871764 -5.046592 -2.749530

O -0.099109 0.808843 -3.549517

H -0.617126 1.442792 -1.617581

H 0.822492 0.404474 -1.782622

C -1.323655 1.025752 -4.263205

C -3.372891 1.392922 -2.977652

C -1.722747 2.524609 -4.302549

C -2.888338 2.650196 -3.348883

C -3.496302 3.805237 -2.855686

C -4.584057 3.680338 -1.988613

C -5.062415 2.417443 -1.620097

C -4.461485 1.257961 -2.114607

H -3.127254 4.788743 -3.136005

H -5.062209 4.572903 -1.594073

H -5.908887 2.336631 -0.944070

H -4.828643 0.276489 -1.824544

H -0.863296 3.150226 -4.037181

H -2.017172 2.806256 -5.322698

H -1.113629 0.629352 -5.256507

H -3.135892 -0.209994 -4.445239

C -4.120235 -2.416746 -2.828845

O -4.904919 -2.277957 -1.875966

C -4.388090 -2.875698 -4.145648

C -3.298124 -2.957915 -5.107242

C -3.665271 -3.413602 -6.428734

C -4.959647 -3.753373 -6.731228

C -6.008691 -3.673070 -5.768942

C -5.717581 -3.240182 -4.500785

O -2.110966 -2.631254 -4.797657

H -2.872219 -3.485560 -7.164889

H -5.196502 -4.097776 -7.736564

H -7.021163 -3.952183 -6.044868

H -6.486073 -3.162462 -3.736249

C 0.822938 -3.059420 -5.869649

C 0.836537 -1.948287 -6.859070

O 1.121970 -2.924769 -4.701954

C 0.513996 -4.494337 -6.377586

C 1.137203 -0.665395 -6.370682

C 1.206113 0.418321 -7.240230

C 0.978807 0.234141 -8.607997

C 0.679394 -1.037217 -9.099850

C 0.607255 -2.126938 -8.233168

H 1.299332 -0.539350 -5.305540

H 1.442376 1.406209 -6.853965

H 1.036724 1.079740 -9.288355

H 0.503071 -1.183217 -10.161752

H 0.377818 -3.106842 -8.633500

F 1.487604 -4.883070 -7.238341

F 0.487545 -5.373174 -5.371566

F -0.658570 -4.573324 -7.038024

TS1_1-B_

C -2.205050 -2.037358 -2.857464

N -1.476033 -2.314515 -1.752508

N -0.707278 -1.246451 -1.355176

C -1.014628 -0.310021 -2.195846

N -1.937431 -0.735627 -3.121472

C -2.459871 0.229115 -4.106904

C -0.471156 1.072198 -2.318922

C -1.472751 -3.462980 -0.897156

C -2.405461 -3.551818 0.143377

C -2.398712 -4.615347 1.031365

C -1.423777 -5.596434 0.884229

C -0.460740 -5.519563 -0.110166

C -0.480055 -4.441344 -0.991923

Cl -3.615173 -2.309882 0.343783

H -3.136790 -4.675725 1.818962

Cl -1.404717 -6.949910 1.992247

H 0.303196 -6.278891 -0.204020

Cl 0.765800 -4.342662 -2.193766

O -0.210667 1.315596 -3.690111

H -1.170176 1.803826 -1.892890

H 0.476703 1.146304 -1.788173

C -1.329913 1.220229 -4.577919

C -3.542452 1.128821 -3.541232

C -2.058100 2.576497 -4.740612

C -3.310158 2.455916 -3.906500

C -4.201272 3.452873 -3.517744

C -5.319175 3.106241 -2.760047

C -5.550418 1.776242 -2.402336

C -4.663411 0.775284 -2.795496

H -4.027966 4.488083 -3.795830

H -6.016950 3.875951 -2.446606

H -6.427445 1.518965 -1.817947

H -4.854305 -0.258158 -2.524984

H -1.399739 3.395047 -4.439132

H -2.308471 2.741463 -5.794726

H -0.894326 0.865392 -5.509645

H -2.783989 -0.339753 -4.973306

C -3.261766 -3.015253 -3.366757

O -3.487862 -3.927460 -2.592265

C -4.129508 -2.782524 -4.543330

C -3.729659 -2.353323 -5.836815

C -4.705652 -2.306874 -6.856297

C -6.022733 -2.643447 -6.615699

C -6.416840 -3.084850 -5.345420

C -5.473265 -3.173703 -4.342404

O -2.499992 -1.955386 -6.152913

H -4.373115 -1.997874 -7.840634

H -6.749315 -2.580916 -7.420182

H -7.446774 -3.364455 -5.152664

H -5.749960 -3.548982 -3.364468

C -1.143207 -2.975563 -5.796082

C -0.027145 -2.019483 -6.206997

O -1.188018 -3.380574 -4.598410

C -1.389508 -4.118627 -6.830903

C 1.052672 -1.877390 -5.333935

C 2.109247 -1.023121 -5.641919

C 2.096536 -0.298867 -6.830744

C 1.021674 -0.435890 -7.709436

C -0.033496 -1.291464 -7.403186

H 1.049394 -2.446931 -4.412979

H 2.941738 -0.923538 -4.952387

H 2.918744 0.366934 -7.074231

H 1.005936 0.124235 -8.639535

H -0.867837 -1.388108 -8.083971

F -0.297522 -4.913561 -6.872510

F -2.433521 -4.883777 -6.476348

F -1.614622 -3.698705 -8.097954

Int1_1-B_

C -2.074340 -2.056398 -2.587446

N -1.729795 -2.325555 -1.318032

N -1.327816 -1.201162 -0.628634

C -1.459618 -0.247459 -1.499417

N -1.922852 -0.720696 -2.703216

C -2.078579 0.198212 -3.839730

C -1.036596 1.180650 -1.439031

C -1.706051 -3.580090 -0.632048

C -2.808742 -3.995715 0.115030

C -2.782557 -5.181136 0.837402

C -1.623753 -5.946788 0.813420

C -0.501801 -5.551571 0.094657

C -0.547884 -4.362538 -0.620141

Cl -4.262487 -3.027305 0.145373

H -3.647925 -5.496491 1.403569

Cl -1.574115 -7.444424 1.716212

H 0.393117 -6.158086 0.083383

Cl 0.850040 -3.867037 -1.523470

O -0.253915 1.428286 -2.601690

H -1.901411 1.851197 -1.371463

H -0.392563 1.345570 -0.577066

C -0.889534 1.226084 -3.873634

C -3.318184 1.072554 -3.758549

C -1.529178 2.526790 -4.407033

C -2.998571 2.391749 -4.086659

C -3.992721 3.366027 -4.109222

C -5.302855 3.004597 -3.796553

C -5.617732 1.682487 -3.476394

C -4.626196 0.702204 -3.460707

H -3.753317 4.394690 -4.361361

H -6.085084 3.756799 -3.804713

H -6.642442 1.411989 -3.244739

H -4.880111 -0.327166 -3.232566

H -1.050194 3.397174 -3.952422

H -1.372538 2.601629 -5.489297

H -0.091099 0.857150 -4.515465

H -2.060962 -0.401572 -4.746404

C -2.516446 -3.174625 -3.579914

O -2.391803 -4.307399 -3.114140

C -3.736506 -2.821239 -4.412472

C -3.704976 -2.309084 -5.716739

C -4.900468 -2.058123 -6.399901

C -6.122846 -2.328094 -5.803352

C -6.170283 -2.874464 -4.519525

C -4.982252 -3.126444 -3.846642

O -2.562422 -1.992763 -6.398897

H -4.839797 -1.666058 -7.408820

H -7.039805 -2.130942 -6.349479

H -7.121248 -3.117149 -4.057531

H -4.999098 -3.583900 -2.863685

C -1.307022 -2.632208 -6.000492

C -0.200408 -1.795822 -6.656228

O -1.146948 -2.682137 -4.663903

C -1.317586 -4.061908 -6.638681

C 1.032955 -1.691620 -6.010331

C 2.058861 -0.934009 -6.571359

C 1.860004 -0.274362 -7.782917

C 0.631726 -0.381167 -8.432658

C -0.393816 -1.142774 -7.876216

H 1.171225 -2.205462 -5.067423

H 3.015098 -0.862118 -6.062530

H 2.659097 0.315811 -8.220716

H 0.470960 0.126257 -9.378837

H -1.346431 -1.224819 -8.383604

F -0.150416 -4.685719 -6.401592

F -2.302483 -4.834627 -6.150976

F -1.484742 -4.009547 -7.980359

TS2_1-B_

C -2.045977 -1.923864 -2.581178

N -1.714762 -2.166916 -1.302738

N -1.276938 -1.042358 -0.610234

C -1.369481 -0.103660 -1.499101

N -1.831932 -0.592961 -2.699959

C -2.011089 0.300461 -3.845460

C -0.937572 1.322492 -1.460442

C -1.717730 -3.418662 -0.621163

C -2.847970 -3.849542 0.075394

C -2.858367 -5.057800 0.760458

C -1.708739 -5.836887 0.746155

C -0.560748 -5.433691 0.075752

C -0.572955 -4.221248 -0.600458

Cl -4.290807 -2.864243 0.100721

H -3.743648 -5.379810 1.290920

Cl -1.704737 -7.364279 1.601247

H 0.326663 -6.051088 0.071715

Cl 0.866534 -3.713655 -1.433401

O -0.185572 1.566638 -2.646075

H -1.798534 1.997159 -1.376159

H -0.270588 1.497276 -0.618063

C -0.859360 1.371596 -3.896909

C -3.282880 1.127916 -3.779856

C -1.550741 2.664074 -4.392101

C -3.016495 2.461925 -4.091106

C -4.051639 3.393387 -4.112687

C -5.347757 2.972531 -3.816822

C -5.608754 1.634824 -3.511318

C -4.575761 0.698722 -3.494437

H -3.854834 4.434452 -4.350778

H -6.161500 3.690645 -3.824676

H -6.622720 1.319380 -3.289137

H -4.783222 -0.342002 -3.268617

H -1.112141 3.537085 -3.902449

H -1.387645 2.786347 -5.469116

H -0.069602 1.042159 -4.570723

H -1.980960 -0.313338 -4.744675

C -2.361280 -3.352238 -3.822550

O -2.182601 -4.401457 -3.230756

C -3.679716 -2.995906 -4.462615

C -3.742243 -2.393071 -5.719130

C -4.968102 -2.080060 -6.305913

C -6.145172 -2.390345 -5.637067

C -6.102656 -3.027324 -4.394200

C -4.873935 -3.335333 -3.822753

O -2.608679 -2.061906 -6.427536

H -4.977026 -1.615106 -7.285234

H -7.099063 -2.150495 -6.095506

H -7.022185 -3.293623 -3.884072

H -4.816505 -3.851018 -2.871246

C -1.397462 -2.744018 -6.076940

C -0.242616 -1.922379 -6.645791

O -1.221901 -2.861389 -4.709748

C -1.421251 -4.148616 -6.769922

C 0.999025 -1.950145 -6.007249

C 2.061341 -1.205438 -6.513984

C 1.891890 -0.431565 -7.660524

C 0.654996 -0.408616 -8.301845

C -0.409118 -1.155242 -7.801093

H 1.120289 -2.549830 -5.114428

H 3.023131 -1.232190 -6.012031

H 2.720875 0.147722 -8.054706

H 0.517133 0.187825 -9.198092

H -1.369246 -1.138059 -8.300500

F -0.275735 -4.806130 -6.533278

F -2.434814 -4.921005 -6.345223

F -1.554650 -4.017975 -8.106566

PC_1-B_

C -2.048922 -1.681025 -2.644786

N -1.670455 -1.898783 -1.363273

N -1.090620 -0.812222 -0.696309

C -1.136136 0.109170 -1.604429

N -1.693013 -0.366675 -2.770955

C -1.870480 0.504321 -3.923083

C -0.603681 1.501201 -1.612931

C -1.784746 -3.137100 -0.681489

C -2.945532 -3.466562 0.026257

C -3.078273 -4.684815 0.681976

C -2.022633 -5.585799 0.626921

C -0.849697 -5.295160 -0.055824

C -0.740478 -4.068632 -0.700151

Cl -4.272744 -2.333754 0.103124

H -3.984433 -4.920680 1.222551

Cl -2.176505 -7.129234 1.443456

H -0.037297 -6.007141 -0.095536

Cl 0.737873 -3.702935 -1.544540

O 0.080205 1.698547 -2.850216

H -1.409560 2.234604 -1.478145

H 0.130246 1.641320 -0.821146

C -0.686062 1.533533 -4.049386

C -3.111603 1.375511 -3.847274

C -1.350693 2.857041 -4.507441

C -2.816390 2.697057 -4.181135

C -3.826656 3.656576 -4.191445

C -5.126727 3.275512 -3.861401

C -5.416223 1.950008 -3.527357

C -4.407611 0.987565 -3.519431

H -3.607322 4.689623 -4.445189

H -5.920443 4.015912 -3.859705

H -6.432092 1.668837 -3.269174

H -4.624575 -0.042693 -3.256048

H -0.877618 3.707029 -4.008667

H -1.203210 2.999833 -5.584401

H 0.043403 1.175383 -4.775016

H -1.899872 -0.141389 -4.802720

C -2.299519 -4.534052 -4.462666

O -1.984526 -5.282839 -3.584145

C -3.666751 -4.124051 -4.811513

C -3.865010 -3.139926 -5.779194

C -5.142097 -2.688610 -6.092056

C -6.231549 -3.248919 -5.432774

C -6.052726 -4.246719 -4.470134

C -4.771149 -4.678664 -4.159932

O -2.797588 -2.552478 -6.412869

H -5.265433 -1.917784 -6.843636

H -7.231662 -2.906173 -5.677224

H -6.910283 -4.676788 -3.965302

H -4.595589 -5.439656 -3.408857

C -1.568077 -3.256074 -6.420920

C -0.457082 -2.245094 -6.660568

O -1.294626 -3.961807 -5.229919

C -1.605130 -4.302530 -7.585038

C 0.775022 -2.387515 -6.021299

C 1.791381 -1.465047 -6.259013

C 1.585676 -0.403410 -7.137094

C 0.355823 -0.264170 -7.778778

C -0.664127 -1.182475 -7.544590

H 0.927904 -3.208996 -5.333985

H 2.745013 -1.577837 -5.754385

H 2.380809 0.311424 -7.322976

H 0.190069 0.557544 -8.467959

H -1.618977 -1.072288 -8.042724

F -0.418296 -4.910164 -7.712547

F -2.531912 -5.255699 -7.365993

F -1.904116 -3.709388 -8.753358

RC_1-B-minor_

C -2.676668 -1.503090 -2.345159

N -1.605986 -1.686704 -1.557151

N -0.682420 -0.661250 -1.680607

C -1.251517 0.156686 -2.511069

N -2.478320 -0.301873 -2.917710

C -3.308518 0.432868 -3.872476

C -0.750989 1.409591 -3.144804

C -1.389958 -2.727158 -0.606092

C -1.762251 -2.541777 0.731983

C -1.568555 -3.546622 1.671414

C -0.982321 -4.738423 1.261688

C -0.572566 -4.940384 -0.049097

C -0.769655 -3.925878 -0.981887

Cl -2.450813 -1.034074 1.259041

H -1.868290 -3.397103 2.699263

Cl -0.739591 -6.012022 2.437079

H -0.100695 -5.865900 -0.348861

Cl -0.216335 -4.145254 -2.605809

O -1.038559 1.334426 -4.534573

H -1.207727 2.292424 -2.678352

H 0.330592 1.477962 -3.044907

C -2.411590 1.203055 -4.915850

C -4.144174 1.516947 -3.215669

C -3.105219 2.576379 -5.097889

C -4.026992 2.719883 -3.911794

C -4.733809 3.842643 -3.486610

C -5.549345 3.742915 -2.360629

C -5.661716 2.534804 -1.667884

C -4.960015 1.407212 -2.090969

H -4.648530 4.784846 -4.019709

H -6.100970 4.612316 -2.017402

H -6.300075 2.471581 -0.792950

H -5.045672 0.470398 -1.549159

H -2.354899 3.367845 -5.172157

H -3.670846 2.589692 -6.036486

H -2.363976 0.638762 -5.843572

H -3.919774 -0.308380 -4.388309

C -4.008649 -2.252597 -2.215244

O -4.602190 -1.984022 -1.165706

C -4.409615 -3.110325 -3.272707

C -3.560808 -3.244826 -4.446209

C -4.050510 -4.140624 -5.467902

C -5.238296 -4.805666 -5.324449

C -6.053623 -4.654330 -4.167036

C -5.638364 -3.818323 -3.166635

O -2.478446 -2.603439 -4.561683

H -3.439883 -4.261393 -6.356286

H -5.571677 -5.470933 -6.117414

H -6.988603 -5.197887 -4.086642

H -6.229139 -3.675896 -2.267354

C -0.001268 -3.375204 -6.431548

C -0.559682 -3.918904 -7.698220

O 0.403280 -4.071736 -5.533594

C 0.149940 -1.832408 -6.280581

C -0.831219 -5.295120 -7.732182

C -1.333190 -5.884095 -8.883853

C -1.558739 -5.107666 -10.021801

C -1.280945 -3.742690 -10.000962

C -0.787228 -3.145503 -8.845241

H -0.644546 -5.880837 -6.840158

H -1.548887 -6.947208 -8.898095

H -1.949140 -5.567535 -10.924052

H -1.449726 -3.139679 -10.886825

H -0.574968 -2.084989 -8.848073

F 1.176515 -1.414317 -7.067934

F -0.946460 -1.155768 -6.678889

F 0.431301 -1.488019 -5.029153

TS1_1-B-minor_

C -2.284523 -1.583627 -2.810486

N -1.358571 -1.686038 -1.832372

N -0.732880 -0.488797 -1.580959

C -1.320796 0.345684 -2.378332

N -2.289881 -0.270599 -3.133579

C -3.118466 0.550620 -4.028539

C -1.049455 1.791310 -2.623343

C -0.974558 -2.812642 -1.040173

C -1.705957 -3.140411 0.108010

C -1.343249 -4.210338 0.911213

C -0.211061 -4.942234 0.569972

C 0.569166 -4.607777 -0.526312

C 0.194362 -3.528076 -1.323150

Cl -3.082945 -2.180541 0.581472

H -1.928032 -4.461600 1.784968

Cl 0.254560 -6.302161 1.568002

H 1.464578 -5.165668 -0.762792

Cl 1.229088 -3.077101 -2.635612

O -1.063737 2.015253 -4.024005

H -1.781416 2.417800 -2.097540

H -0.052570 2.045694 -2.266972

C -2.254204 1.654866 -4.733169

C -4.199104 1.329756 -3.301215

C -3.224040 2.851721 -4.870830

C -4.261411 2.634610 -3.794026

C -5.211704 3.521876 -3.296359

C -6.089046 3.088771 -2.302266

C -6.023421 1.781848 -1.814998

C -5.076901 0.888734 -2.316057

H -5.267448 4.539806 -3.669990

H -6.829240 3.774075 -1.902195

H -6.713539 1.458128 -1.043144

H -5.032855 -0.127877 -1.937990

H -2.677086 3.792937 -4.779606

H -3.688251 2.844714 -5.864024

H -1.895077 1.287839 -5.692534

H -3.532293 -0.099976 -4.789782

C -3.251207 -2.708654 -3.163897

O -3.381813 -3.547714 -2.291415

C -4.159753 -2.654633 -4.334791

C -3.813867 -2.232485 -5.644660

C -4.796846 -2.300361 -6.651666

C -6.076153 -2.752088 -6.381072

C -6.411348 -3.193573 -5.095700

C -5.454743 -3.159814 -4.098551

O -2.611245 -1.752227 -5.967106

H -4.509807 -1.986477 -7.648528

H -6.815438 -2.779807 -7.175773

H -7.408943 -3.561799 -4.882347

H -5.684382 -3.520764 -3.102995

C -1.332803 -2.814040 -5.665139

C -1.485736 -3.896652 -6.731857

O -1.351444 -3.193582 -4.449530

C -0.192005 -1.793375 -5.970920

C -1.501511 -5.223574 -6.298394

C -1.644922 -6.265053 -7.211793

C -1.775365 -5.989661 -8.570760

C -1.766822 -4.666440 -9.009977

C -1.625501 -3.624314 -8.097826

H -1.401142 -5.415843 -5.237326

H -1.654292 -7.292412 -6.860807

H -1.885633 -6.800238 -9.284691

H -1.874349 -4.444067 -10.067308

H -1.632213 -2.600174 -8.446747

F 0.997840 -2.429250 -5.954201

F -0.282538 -1.157532 -7.159318

F -0.133037 -0.824857 -5.025429

Int1_1-B-minor_

C -2.073863 -1.598939 -2.782425

N -1.599285 -1.841114 -1.550929

N -1.326338 -0.684325 -0.850834

C -1.666238 0.258178 -1.675989

N -2.135153 -0.253593 -2.862137

C -2.507048 0.655100 -3.955594

C -1.467993 1.733352 -1.595713

C -1.301546 -3.089806 -0.919466

C -2.224928 -3.682889 -0.058327

C -1.923142 -4.854168 0.624331

C -0.668697 -5.421932 0.444810

C 0.281981 -4.844284 -0.388674

C -0.039223 -3.673854 -1.061628

Cl -3.799027 -2.961342 0.172062

H -2.652810 -5.309005 1.279737

Cl -0.271928 -6.898848 1.295994

H 1.255630 -5.296243 -0.517926

Cl 1.145025 -2.949621 -2.106627

O -0.815655 2.129231 -2.797118

H -2.420754 2.255952 -1.450886

H -0.799980 1.979178 -0.772110

C -1.487388 1.847610 -4.035661

C -3.851160 1.337137 -3.763067

C -2.343888 3.043923 -4.503754

C -3.751074 2.692862 -4.083204

C -4.873668 3.512490 -4.009427

C -6.089976 2.960823 -3.607595

C -6.185220 1.603671 -3.293913

C -5.064563 0.777628 -3.374601

H -4.804982 4.567956 -4.254981

H -6.970568 3.591525 -3.539832

H -7.138874 1.184843 -2.990573

H -5.147963 -0.279223 -3.146592

H -1.965856 3.972575 -4.070031

H -2.277218 3.145254 -5.593266

H -0.683858 1.608595 -4.728964

H -2.465294 0.083729 -4.879751

C -2.404569 -2.756564 -3.794837

O -2.095220 -3.871884 -3.333203

C -3.782132 -2.587248 -4.437718

C -3.994723 -2.055630 -5.715257

C -5.288823 -1.941161 -6.230616

C -6.375066 -2.375041 -5.483950

C -6.177658 -2.950136 -4.226244

C -4.888265 -3.061410 -3.721809

O -2.971603 -1.617980 -6.514589

H -5.414506 -1.523660 -7.223380

H -7.376180 -2.285543 -5.893408

H -7.021042 -3.320333 -3.653039

H -4.711589 -3.533532 -2.761774

C -1.691655 -2.259367 -6.251360

C -1.688805 -3.685873 -6.808045

O -1.335354 -2.142655 -4.939243

C -0.696236 -1.366703 -7.050391

C -0.969338 -4.684270 -6.150209

C -0.924580 -5.973195 -6.676274

C -1.591986 -6.272910 -7.861133

C -2.310160 -5.277282 -8.521268

C -2.355864 -3.988063 -7.999643

H -0.480879 -4.451890 -5.214546

H -0.372549 -6.746561 -6.151741

H -1.557799 -7.279258 -8.267158

H -2.836380 -5.504300 -9.443163

H -2.912909 -3.214944 -8.514713

F 0.556636 -1.822516 -6.924015

F -0.989915 -1.316339 -8.361953

F -0.707310 -0.087051 -6.599902

TS2_1-B-minor_

C -2.041398 -1.482413 -2.731305

N -1.553278 -1.679456 -1.495266

N -1.218588 -0.506142 -0.826364

C -1.535839 0.412947 -1.683161

N -2.038002 -0.133479 -2.842822

C -2.460538 0.731204 -3.945348

C -1.318669 1.887488 -1.657029

C -1.311013 -2.918077 -0.832801

C -2.288882 -3.496411 -0.021809

C -2.057564 -4.689052 0.651832

C -0.819000 -5.300548 0.508473

C 0.182542 -4.747625 -0.279399

C -0.070817 -3.554214 -0.942594

Cl -3.844573 -2.722805 0.163290

H -2.827263 -5.126962 1.272024

Cl -0.510433 -6.806020 1.347843

H 1.141934 -5.235327 -0.382094

Cl 1.181220 -2.861827 -1.929895

O -0.734169 2.251392 -2.904265

H -2.258044 2.423487 -1.473150

H -0.604218 2.154556 -0.880268

C -1.485925 1.957122 -4.090281

C -3.822836 1.368862 -3.733206

C -2.400367 3.134448 -4.502035

C -3.782871 2.727127 -4.051849

C -4.935001 3.502491 -3.948664

C -6.119127 2.902751 -3.520894

C -6.154003 1.542139 -3.207795

C -5.003478 0.762011 -3.315477

H -4.913589 4.560802 -4.191082

H -7.021837 3.498500 -3.430520

H -7.083002 1.086785 -2.881115

H -5.035636 -0.296623 -3.080812

H -2.042093 4.064665 -4.054339

H -2.369542 3.270093 -5.589415

H -0.724191 1.743779 -4.837806

H -2.433169 0.133870 -4.855055

C -2.294467 -2.970964 -3.951502

O -1.955227 -3.982214 -3.356419

C -3.701406 -2.775523 -4.464377

C -3.953153 -2.152047 -5.688493

C -5.257568 -1.958636 -6.142071

C -6.321393 -2.413221 -5.372961

C -6.086618 -3.076452 -4.165143

C -4.782154 -3.261278 -3.724121

O -2.923959 -1.703718 -6.484767

H -5.414538 -1.468730 -7.096488

H -7.336979 -2.266724 -5.726117

H -6.917620 -3.454483 -3.579217

H -4.573225 -3.785067 -2.798328

C -1.687966 -2.407559 -6.272948

C -1.734760 -3.800538 -6.900631

O -1.315117 -2.391614 -4.935700

C -0.634164 -1.518465 -6.991111

C -1.020359 -4.850889 -6.321400

C -1.024601 -6.108234 -6.920473

C -1.733201 -6.323031 -8.099869

C -2.442921 -5.274184 -8.682078

C -2.441368 -4.016195 -8.087785

H -0.494383 -4.686366 -5.390826

H -0.476559 -6.922730 -6.458081

H -1.736174 -7.304862 -8.562848

H -2.999282 -5.435016 -9.599948

H -2.991452 -3.200922 -8.541658

F 0.588424 -2.052389 -6.890046

F -0.920409 -1.369933 -8.295678

F -0.583122 -0.278427 -6.453697

PC_1-B-minor_

C -2.220806 -1.296828 -2.562927

N -1.626894 -1.379189 -1.349325

N -1.101920 -0.187016 -0.834748

C -1.406745 0.658402 -1.766361

N -2.065392 0.040270 -2.806054

C -2.532143 0.802746 -3.954501

C -1.075758 2.103248 -1.920238

C -1.467756 -2.572810 -0.600213

C -2.428267 -2.971205 0.335268

C -2.290039 -4.144285 1.067700

C -1.163714 -4.928728 0.855325

C -0.183397 -4.567218 -0.058054

C -0.342214 -3.386675 -0.774784

Cl -3.845141 -1.985646 0.605525

H -3.044758 -4.435203 1.785118

Cl -0.976246 -6.414620 1.767495

H 0.686039 -5.189410 -0.217561

Cl 0.893009 -2.930739 -1.911917

O -0.624938 2.308417 -3.258566

H -1.943389 2.735023 -1.687989

H -0.254243 2.385681 -1.264087

C -1.537486 1.970892 -4.308861

C -3.859124 1.503187 -3.723934

C -2.443346 3.165086 -4.706140

C -3.806707 2.830073 -4.149987

C -4.932809 3.643289 -4.039676

C -6.102495 3.111550 -3.497904

C -6.148699 1.781325 -3.072688

C -5.024066 0.965131 -3.184492

H -4.901898 4.679614 -4.363406

H -6.983864 3.737743 -3.401166

H -7.064932 1.382544 -2.649223

H -5.050059 -0.067185 -2.850523

H -2.025246 4.097988 -4.318826

H -2.480213 3.264965 -5.797291

H -0.885760 1.668472 -5.127829

H -2.596305 0.103023 -4.788855

C -2.214654 -4.232119 -4.288167

O -1.849399 -4.923142 -3.381172

C -3.609740 -3.934062 -4.651415

C -3.878499 -2.964344 -5.619351

C -5.183372 -2.581605 -5.908405

C -6.229044 -3.198368 -5.229471

C -5.979852 -4.186041 -4.271334

C -4.672375 -4.546209 -3.980407

O -2.847693 -2.339191 -6.276830

H -5.362632 -1.817186 -6.655471

H -7.250321 -2.908686 -5.454213

H -6.804579 -4.660413 -3.751545

H -4.443627 -5.288249 -3.224604

C -1.650633 -3.098652 -6.338472

C -1.733255 -4.200428 -7.390631

O -1.262347 -3.610060 -5.078988

C -0.551946 -2.057703 -6.688608

C -1.009789 -5.383859 -7.225798

C -1.063271 -6.374221 -8.203309

C -1.829452 -6.185888 -9.350950

C -2.542225 -5.000536 -9.522036

C -2.492153 -4.008072 -8.548149

H -0.411831 -5.528611 -6.334479

H -0.504421 -7.293808 -8.065405

H -1.870656 -6.959662 -10.110668

H -3.139471 -4.847675 -10.414868

H -3.044321 -3.086024 -8.682473

F 0.643554 -2.649992 -6.789213

F -0.827088 -1.460120 -7.861424

F -0.464090 -1.098271 -5.753702

RC_2-A_

C 0.830786 -1.364619 0.353710

N 1.732083 -1.126752 -0.618489

N 1.394070 -1.758451 -1.799539

C 0.311968 -2.406715 -1.506846

N -0.058784 -2.225582 -0.190567

C -1.059686 -3.082237 0.513535

C -0.478326 -3.299600 -2.402423

C 2.943552 -0.379088 -0.545685

C 3.049578 0.892797 -1.129326

C 4.239617 1.610702 -1.046884

C 5.329615 1.045059 -0.400771

C 5.273244 -0.232784 0.140369

C 4.083061 -0.943472 0.047184

Cl 1.725818 1.594783 -1.992295

H 4.304726 2.595545 -1.488328

Cl 6.821995 1.951657 -0.288681

H 6.138170 -0.678075 0.611866

Cl 4.054870 -2.578563 0.646092

O -1.015134 -4.376060 -1.649659

H -1.263868 -2.725355 -2.908547

H 0.171592 -3.730289 -3.162298

C -1.796817 -3.980984 -0.534586

C -2.212201 -2.358799 1.177095

C -3.126652 -3.256688 -0.850697

C -3.380601 -2.483507 0.416911

C -4.579875 -1.954552 0.883419

C -4.597687 -1.300144 2.115187

C -3.433598 -1.189360 2.877828

C -2.232925 -1.727094 2.418002

H -5.492338 -2.058305 0.304031

H -5.529255 -0.890220 2.492996

H -3.462205 -0.690005 3.840261

H -1.338773 -1.659272 3.024815

H -3.028311 -2.577783 -1.702977

H -3.917174 -3.969279 -1.096962

H -2.024565 -4.917819 -0.023876

H -0.476054 -3.673659 1.219286

C 1.035993 -1.181175 1.863187

O 1.332182 -2.223085 2.452394

C 0.848862 0.128237 2.379642

C 0.179929 1.086046 1.521054

C 0.020343 2.410568 2.065293

C 0.431673 2.702081 3.339997

C 1.044940 1.731058 4.180516

C 1.241833 0.462346 3.700186

O -0.250650 0.741517 0.381439

H -0.458575 3.156427 1.440352

H 0.282493 3.707662 3.725905

H 1.358238 2.001986 5.182819

H 1.713427 -0.305219 4.306425

C -1.942918 2.254728 -1.880251

C -2.282118 0.826663 -2.410463

C -2.809055 2.861600 -0.839917

O -1.047029 2.844196 -2.433999

F -2.315844 -0.125416 -1.455249

F -3.505814 0.845904 -2.991918

F -1.399320 0.433632 -3.332357

C -2.676785 4.244418 -0.639614

C -3.455342 4.895122 0.306376

C -4.371446 4.169967 1.070210

C -4.501979 2.795815 0.883219

C -3.728054 2.138678 -0.067704

H -1.956880 4.787642 -1.240169

H -3.350792 5.965291 0.450756

H -4.979937 4.676355 1.812723

H -5.203679 2.229205 1.485735

H -3.827338 1.067880 -0.180713

TS1_2-A_

C 0.438509 -0.905687 -0.128164

N 1.411816 -1.078084 -1.046290

N 1.077003 -2.017401 -1.990377

C -0.104190 -2.415681 -1.635014

N -0.543366 -1.761181 -0.504875

C -1.739149 -2.292092 0.183136

C -0.860813 -3.624647 -2.063588

C 2.749704 -0.567621 -1.060789

C 3.120341 0.499181 -1.885456

C 4.430779 0.971872 -1.880722

C 5.372784 0.345757 -1.079045

C 5.050733 -0.764998 -0.307440

C 3.742304 -1.222964 -0.318900

Cl 1.993061 1.227296 -2.976293

H 4.701633 1.810550 -2.506902

Cl 7.016853 0.944291 -1.063483

H 5.800579 -1.269586 0.285436

Cl 3.371094 -2.672268 0.575680

O -1.006860 -4.439267 -0.896607

H -1.826138 -3.372189 -2.511151

H -0.270758 -4.199274 -2.774675

C -1.623732 -3.852996 0.262583

C -3.086714 -2.091988 -0.504843

C -3.083517 -4.317774 0.387275

C -3.852950 -3.253889 -0.358134

C -5.146691 -3.312179 -0.863966

C -5.664406 -2.198632 -1.525797

C -4.897141 -1.042314 -1.668316

C -3.602328 -0.974461 -1.152336

H -5.742149 -4.213552 -0.754563

H -6.669839 -2.233012 -1.933013

H -5.307702 -0.179506 -2.181961

H -3.024391 -0.068155 -1.237088

H -3.198359 -5.324019 -0.020548

H -3.380311 -4.354742 1.443137

H -1.001691 -4.161926 1.104655

H -1.746908 -1.868715 1.187348

C 0.637617 -0.178930 1.203001

O 1.782629 -0.177527 1.612949

C -0.517269 0.169698 2.059246

C -1.710176 0.786837 1.604542

C -2.728606 1.045252 2.543636

C -2.576072 0.709581 3.877319

C -1.380684 0.139115 4.333666

C -0.361671 -0.107649 3.432102

O -1.905937 1.132812 0.335249

H -3.631608 1.523390 2.182516

H -3.382202 0.910875 4.576348

H -1.252570 -0.104442 5.382790

H 0.578823 -0.533888 3.761971

C -0.733845 2.168988 -0.301595

C -1.307484 2.177929 -1.754073

C -0.939423 3.486407 0.443144

O 0.416732 1.630261 -0.221612

F -2.623652 2.477751 -1.859707

F -0.643365 3.078570 -2.504386

F -1.147601 0.969662 -2.347300

C 0.183002 4.062130 1.041610

C 0.075159 5.259205 1.744915

C -1.159260 5.893898 1.858588

C -2.285393 5.322180 1.267947

C -2.179296 4.124769 0.565780

H 1.132938 3.551318 0.943438

H 0.956746 5.695835 2.204119

H -1.245634 6.827705 2.405774

H -3.252036 5.808526 1.357582

H -3.061426 3.683561 0.120797

Int1_2-A_

C 0.278087 -0.936469 -0.011965

N 1.360752 -1.250248 -0.744653

N 1.284206 -2.509291 -1.300195

C 0.135263 -2.951267 -0.898866

N -0.517338 -2.027494 -0.109792

C -1.862004 -2.350746 0.400716

C -0.476851 -4.303806 -1.036427

C 2.534397 -0.472329 -0.986196

C 2.614502 0.356967 -2.107304

C 3.762284 1.102664 -2.358868

C 4.837361 0.986998 -1.489424

C 4.803104 0.134838 -0.390883

C 3.650444 -0.598537 -0.152535

Cl 1.290853 0.463248 -3.228330

H 3.809257 1.753560 -3.220818

Cl 6.284701 1.924167 -1.793058

H 5.653733 0.044071 0.270037

Cl 3.620838 -1.692593 1.198827

O -0.933584 -4.688895 0.256600

H -1.285822 -4.304586 -1.776163

H 0.278036 -5.027479 -1.338737

C -1.928334 -3.852923 0.859852

C -2.949279 -2.240425 -0.651769

C -3.363647 -4.300157 0.496439

C -3.801133 -3.343958 -0.587361

C -4.892094 -3.430351 -1.447736

C -5.110589 -2.404364 -2.367579

C -4.254106 -1.302670 -2.423203

C -3.163410 -1.209237 -1.559649

H -5.561404 -4.284620 -1.410365

H -5.955050 -2.463208 -3.046738

H -4.436673 -0.507376 -3.137711

H -2.508148 -0.348501 -1.597983

H -3.364447 -5.350338 0.194533

H -4.014529 -4.218823 1.374924

H -1.728296 -3.935019 1.927937

H -2.058058 -1.697124 1.247432

C 0.218847 0.388432 0.851980

O 1.347860 0.686589 1.285550

C -0.968803 0.409616 1.810822

C -2.195863 1.022601 1.522840

C -3.258691 0.931625 2.427306

C -3.092233 0.260433 3.631447

C -1.854227 -0.297938 3.961418

C -0.803649 -0.207625 3.056290

O -2.416784 1.728392 0.374710

H -4.197359 1.410171 2.171134

H -3.922335 0.198458 4.327982

H -1.709649 -0.785583 4.919615

H 0.173652 -0.612519 3.296988

C -1.206863 2.280109 -0.212962

C -1.687411 2.780964 -1.607010

C -0.712436 3.465333 0.625605

O -0.282057 1.305926 -0.451760

F -2.705420 3.656399 -1.509840

F -0.684125 3.387311 -2.259682

F -2.116696 1.764864 -2.387874

C 0.655349 3.716203 0.738527

C 1.106418 4.815426 1.465465

C 0.197671 5.673182 2.079767

C -1.169620 5.427177 1.966765

C -1.623464 4.330747 1.240128

H 1.356411 3.029143 0.286715

H 2.172814 4.994852 1.557999

H 0.552029 6.527465 2.648413

H -1.884513 6.088965 2.445656

H -2.686351 4.141568 1.152280

TS2_2-A_

C 0.253986 -0.932674 -0.037526

N 1.346763 -1.303295 -0.727307

N 1.298983 -2.606529 -1.203583

C 0.142037 -3.021333 -0.797050

N -0.522695 -2.043858 -0.086212

C -1.876450 -2.301255 0.418887

C -0.482828 -4.372807 -0.868949

C 2.506840 -0.523255 -1.004949

C 2.555686 0.301959 -2.131272

C 3.683169 1.066827 -2.410424

C 4.772963 0.982266 -1.554883

C 4.768251 0.149381 -0.442409

C 3.632375 -0.603384 -0.179151

Cl 1.203657 0.382264 -3.223813

H 3.704424 1.709825 -3.279253

Cl 6.198065 1.942908 -1.892713

H 5.626673 0.089290 0.211911

Cl 3.633107 -1.658467 1.204213

O -0.999560 -4.668376 0.426029

H -1.263820 -4.410897 -1.638294

H 0.268143 -5.128136 -1.093702

C -2.007600 -3.785166 0.929438

C -2.951236 -2.179467 -0.645761

C -3.432767 -4.220933 0.509881

C -3.826358 -3.264360 -0.589666

C -4.903342 -3.336181 -1.469726

C -5.086428 -2.313273 -2.399926

C -4.208802 -1.227511 -2.446316

C -3.131840 -1.150268 -1.564494

H -5.589632 -4.177235 -1.438434

H -5.919436 -2.360865 -3.094026

H -4.364864 -0.433924 -3.169177

H -2.459454 -0.301452 -1.595951

H -3.430052 -5.271275 0.207549

H -4.116942 -4.137578 1.362359

H -1.870337 -3.825158 2.009655

H -2.053329 -1.611484 1.242699

C 0.277531 0.724837 0.910010

O 1.426461 0.894426 1.295830

C -0.878715 0.599390 1.874821

C -2.131766 1.149187 1.588645

C -3.208508 0.958385 2.456542

C -3.021885 0.237993 3.630539

C -1.760616 -0.270297 3.956929

C -0.697022 -0.078008 3.082881

O -2.345184 1.888204 0.454821

H -4.169470 1.391580 2.202576

H -3.858787 0.093435 4.306220

H -1.610608 -0.802390 4.890244

H 0.295193 -0.450927 3.312908

C -1.146509 2.478094 -0.080241

C -1.569179 2.940592 -1.502959

C -0.708872 3.676043 0.765373

O -0.160401 1.527727 -0.289495

F -2.600860 3.801867 -1.453315

F -0.549776 3.548835 -2.124899

F -1.954289 1.902994 -2.273801

C 0.646315 3.986271 0.891035

C 1.039980 5.102222 1.625601

C 0.087326 5.916874 2.232120

C -1.266656 5.611986 2.103597

C -1.664452 4.498468 1.370385

H 1.384254 3.334768 0.443518

H 2.096139 5.329167 1.728659

H 0.397675 6.784311 2.806275

H -2.014849 6.241192 2.575073

H -2.716835 4.263155 1.268843

PC_2-A_

C 1.369338 0.997832 0.568969

N 1.204721 2.252348 0.087615

N 2.368400 2.914980 -0.322043

C 3.275315 2.019409 -0.097978

N 2.725847 0.874284 0.435060

C 3.550841 -0.287512 0.737707

C 4.758440 2.082566 -0.225411

C -0.034113 2.938583 0.000951

C -0.781265 2.930330 -1.181728

C -2.003080 3.586108 -1.274360

C -2.477858 4.265450 -0.160966

C -1.764881 4.305997 1.029064

C -0.546127 3.642746 1.095695

Cl -0.190174 2.074257 -2.580471

H -2.568757 3.560358 -2.195277

Cl -4.019846 5.094936 -0.260792

H -2.147468 4.836160 1.889956

Cl 0.348436 3.701397 2.592653

O 5.319748 1.507433 0.954299

H 5.102240 1.560368 -1.128205

H 5.101866 3.114662 -0.268513

C 4.994999 0.138494 1.210278

C 3.799561 -1.185937 -0.459586

C 5.966073 -0.844818 0.503658

C 5.152411 -1.496436 -0.587772

C 5.581863 -2.326723 -1.621539

C 4.641819 -2.835307 -2.517022

C 3.286684 -2.521445 -2.381181

C 2.854666 -1.692362 -1.347736

H 6.633922 -2.571960 -1.734845

H 4.965844 -3.478736 -3.329033

H 2.563669 -2.921585 -3.084354

H 1.805054 -1.443315 -1.241373

H 6.842366 -0.303834 0.135958

H 6.335726 -1.590713 1.216764

H 5.070095 0.059811 2.294819

H 3.038342 -0.831402 1.533924

C -1.833471 -0.280068 1.104746

O -2.239497 0.796071 1.436290

C -1.163402 -1.267694 1.965194

C -0.605809 -2.414276 1.394570

C 0.133582 -3.309211 2.160877

C 0.294021 -3.056181 3.518983

C -0.273711 -1.922898 4.111039

C -0.993344 -1.029275 3.332386

O -0.749547 -2.663103 0.053606

H 0.563663 -4.184728 1.688572

H 0.862270 -3.754820 4.124528

H -0.142307 -1.739123 5.171401

H -1.418791 -0.125955 3.753078

C -1.871274 -2.032674 -0.541580

C -1.570191 -2.068846 -2.064841

C -3.171789 -2.759069 -0.211196

O -1.949071 -0.655549 -0.225753

F -1.444979 -3.339112 -2.487290

F -2.563056 -1.492823 -2.754080

F -0.430779 -1.426378 -2.360429

C -4.375188 -2.051961 -0.155189

C -5.565147 -2.723095 0.114429

C -5.561840 -4.100447 0.320629

C -4.363485 -4.808762 0.253366

C -3.171974 -4.142863 -0.016413

H -4.377713 -0.980964 -0.316066

H -6.494931 -2.166298 0.163702

H -6.490143 -4.621188 0.532013

H -4.355310 -5.882262 0.410758

H -2.241475 -4.694411 -0.072709

RC_2-A-minor_

C 0.662450 -1.087563 0.035605

N 1.564823 -0.772607 -0.916036

N 1.195090 -1.260972 -2.158976

C 0.105324 -1.919155 -1.919337

N -0.242611 -1.880093 -0.585942

C -1.292880 -2.742659 0.027916

C -0.736287 -2.655932 -2.910106

C 2.835482 -0.148018 -0.756017

C 3.028052 1.215170 -1.024184

C 4.280450 1.800804 -0.861570

C 5.347455 1.013675 -0.451980

C 5.202311 -0.348356 -0.221586

C 3.947985 -0.921823 -0.389849

Cl 1.723474 2.209192 -1.579105

H 4.411462 2.855967 -1.057665

Cl 6.920236 1.750217 -0.240372

H 6.045519 -0.957755 0.072383

Cl 3.793439 -2.641420 -0.167109

O -1.429379 -3.719127 -2.278876

H -1.427391 -1.960278 -3.402155

H -0.100956 -3.098881 -3.676172

C -2.156336 -3.345724 -1.119715

C -2.336014 -2.016628 0.847050

C -3.323538 -2.346464 -1.309847

C -3.491999 -1.803137 0.087031

C -4.603822 -1.198977 0.661755

C -4.546605 -0.812586 2.000789

C -3.401214 -1.048530 2.761741

C -2.287256 -1.662729 2.191605

H -5.506267 -1.031402 0.081937

H -5.406004 -0.333370 2.458617

H -3.375321 -0.753979 3.805256

H -1.405053 -1.860165 2.788448

H -3.063305 -1.544193 -2.008943

H -4.214898 -2.840745 -1.703276

H -2.561597 -4.285819 -0.742314

H -0.757042 -3.506421 0.592960

C 0.926168 -1.076199 1.549761

O 1.310693 -2.151828 2.008752

C 0.679032 0.162459 2.199043

C -0.062904 1.140399 1.433918

C -0.290141 2.405334 2.077942

C 0.132046 2.618074 3.365960

C 0.818712 1.623538 4.116406

C 1.082271 0.410331 3.533463

O -0.489819 0.851575 0.272903

H -0.821199 3.168698 1.521662

H -0.065904 3.580003 3.832416

H 1.135637 1.833916 5.132053

H 1.612761 -0.372247 4.067824

C -2.087033 2.735372 -2.187157

C -3.158740 2.920619 -1.073845

C -2.371241 1.774858 -3.289653

O -1.084282 3.401038 -2.124861

F -2.720816 3.721906 -0.106827

F -4.274127 3.490460 -1.604206

F -3.527949 1.749140 -0.529419

C -1.318279 1.481865 -4.169228

C -1.516321 0.622290 -5.241510

C -2.772548 0.051819 -5.455584

C -3.825787 0.340832 -4.589502

C -3.629523 1.194905 -3.507909

H -0.350885 1.935086 -3.991867

H -0.694438 0.399716 -5.913708

H -2.931026 -0.612260 -6.299309

H -4.805022 -0.093869 -4.759796

H -4.461312 1.416339 -2.852669

TS1_2-A-minor_

C 0.609502 -0.952375 0.117421

N 1.602115 -1.073116 -0.791156

N 1.276790 -1.935688 -1.808736

C 0.080669 -2.337571 -1.511227

N -0.375714 -1.757401 -0.349153

C -1.658672 -2.235637 0.201575

C -0.697078 -3.484479 -2.062229

C 2.940637 -0.564469 -0.768685

C 3.307636 0.538782 -1.542753

C 4.614638 1.017884 -1.523419

C 5.560645 0.359023 -0.752629

C 5.242572 -0.786303 -0.030547

C 3.936291 -1.248504 -0.058849

Cl 2.171020 1.300888 -2.609562

H 4.881075 1.884659 -2.112076

Cl 7.202022 0.960340 -0.714239

H 5.995096 -1.312436 0.539918

Cl 3.563888 -2.727403 0.784134

O -0.965425 -4.351228 -0.958604

H -1.611904 -3.160096 -2.566002

H -0.080144 -4.046099 -2.761140

C -1.686895 -3.802251 0.157114

C -2.896799 -1.852488 -0.598189

C -3.186797 -4.136000 0.064734

C -3.771696 -2.941895 -0.651308

C -4.992592 -2.828374 -1.307412

C -5.320082 -1.617621 -1.918522

C -4.440033 -0.535713 -1.866656

C -3.219737 -0.641559 -1.198467

H -5.675995 -3.670876 -1.354441

H -6.266372 -1.517460 -2.440575

H -4.703014 0.403726 -2.339907

H -2.555731 0.208415 -1.134483

H -3.332835 -5.086076 -0.453449

H -3.611561 -4.242899 1.070697

H -1.206998 -4.232474 1.037267

H -1.723873 -1.889195 1.231632

C 0.822629 -0.337887 1.503403

O 1.988300 -0.206000 1.818994

C -0.277592 -0.260476 2.487224

C -1.541352 0.363207 2.281173

C -2.458420 0.353090 3.359623

C -2.147310 -0.229526 4.572859

C -0.883719 -0.798839 4.785996

C 0.038766 -0.786806 3.758043

O -1.905431 0.946073 1.155422

H -3.414866 0.836290 3.195729

H -2.881144 -0.223949 5.373526

H -0.629142 -1.236151 5.745151

H 1.030231 -1.199716 3.905243

C -0.815423 2.145032 0.401919

C -0.639297 3.168125 1.573124

C -1.765454 2.665779 -0.671209

O 0.247107 1.568259 0.057569

F -1.784823 3.511452 2.205185

F 0.212254 2.722065 2.508575

F -0.115985 4.308806 1.066768

C -1.250476 2.817739 -1.959733

C -2.044954 3.315879 -2.989960

C -3.369391 3.667413 -2.739580

C -3.890726 3.519343 -1.454129

C -3.095412 3.024373 -0.423767

H -0.216574 2.548832 -2.130217

H -1.628082 3.435558 -3.985356

H -3.990941 4.060488 -3.538402

H -4.921918 3.792529 -1.251820

H -3.507674 2.900030 0.567615

Int1_2-A-minor_

C 0.530769 -1.025722 0.111880

N 1.617616 -1.387219 -0.586203

N 1.427931 -2.550682 -1.301956

C 0.202053 -2.877962 -1.031915

N -0.386003 -1.973415 -0.178454

C -1.792776 -2.148423 0.214831

C -0.570509 -4.108673 -1.366878

C 2.892813 -0.744246 -0.661132

C 3.145628 0.212786 -1.645218

C 4.397190 0.803857 -1.762250

C 5.404401 0.410645 -0.890964

C 5.192672 -0.562011 0.079536

C 3.935813 -1.139271 0.179937

Cl 1.885957 0.685193 -2.752939

H 4.576987 1.555156 -2.518592

Cl 6.985134 1.149386 -1.025086

H 5.986478 -0.862192 0.749291

Cl 3.668571 -2.363615 1.391494

O -1.170020 -4.562185 -0.156577

H -1.314482 -3.913241 -2.147675

H 0.100914 -4.895266 -1.706576

C -2.092179 -3.669375 0.483122

C -2.776870 -1.768669 -0.877262

C -3.539920 -3.871077 -0.019655

C -3.763937 -2.747576 -1.003313

C -4.786536 -2.588019 -1.934437

C -4.802515 -1.443198 -2.731217

C -3.814277 -0.465724 -2.594842

C -2.790825 -0.618723 -1.660385

H -5.559054 -3.343284 -2.043977

H -5.593158 -1.309512 -3.462656

H -3.844683 0.426877 -3.209923

H -2.033238 0.145974 -1.536929

H -3.654229 -4.870500 -0.446377

H -4.240950 -3.799644 0.820261

H -1.984925 -3.898180 1.542889

H -1.948602 -1.568622 1.123500

C 0.549264 0.203768 1.104834

O 1.704724 0.631898 1.277224

C -0.348363 -0.060012 2.314749

C -1.527833 0.655059 2.563175

C -2.297665 0.370379 3.696497

C -1.887070 -0.606691 4.593012

C -0.694652 -1.301303 4.379217

C 0.064889 -1.012737 3.251849

O -2.012636 1.639551 1.747802

H -3.203805 0.942768 3.860944

H -2.489976 -0.812173 5.471828

H -0.355127 -2.045193 5.091976

H 1.010613 -1.519744 3.087081

C -1.146173 2.133010 0.705127

C -0.266681 3.254808 1.372834

C -2.029741 2.785206 -0.359538

O -0.413039 1.136750 0.124000

F -1.038562 4.316210 1.703086

F 0.336581 2.855323 2.501956

F 0.677333 3.690483 0.523217

C -1.489087 3.022054 -1.626019

C -2.263461 3.618706 -2.617873

C -3.583076 3.982023 -2.352813

C -4.121115 3.749887 -1.088969

C -3.346767 3.159293 -0.092517

H -0.464779 2.733136 -1.825579

H -1.833494 3.803472 -3.597389

H -4.186341 4.449030 -3.125190

H -5.146644 4.033473 -0.874047

H -3.763748 2.981827 0.890118

TS2_2-A-minor_

C 0.510484 -1.088108 0.052568

N 1.597239 -1.444683 -0.651690

N 1.463680 -2.653732 -1.325841

C 0.258001 -3.019184 -1.023986

N -0.351180 -2.104024 -0.193391

C -1.736696 -2.301412 0.242040

C -0.478436 -4.281866 -1.315408

C 2.831402 -0.740932 -0.764230

C 3.015351 0.223103 -1.757290

C 4.216469 0.910169 -1.879939

C 5.245574 0.612577 -0.996366

C 5.104632 -0.354565 -0.008714

C 3.895308 -1.027054 0.095007

Cl 1.730142 0.578505 -2.881810

H 4.341563 1.660187 -2.648486

Cl 6.763773 1.472730 -1.135552

H 5.914257 -0.576866 0.672309

Cl 3.717588 -2.242869 1.331096

O -1.069434 -4.713696 -0.092259

H -1.229401 -4.132926 -2.101106

H 0.210466 -5.063218 -1.631355

C -2.024000 -3.828802 0.504485

C -2.759130 -1.905529 -0.806926

C -3.458023 -4.061880 -0.031862

C -3.725751 -2.899883 -0.957524

C -4.771332 -2.722752 -1.860545

C -4.831628 -1.543809 -2.602932

C -3.863193 -0.549706 -2.442493

C -2.816694 -0.722638 -1.538146

H -5.528238 -3.490756 -1.989642

H -5.640567 -1.396267 -3.311400

H -3.926110 0.367658 -3.017506

H -2.070467 0.052533 -1.404445

H -3.522498 -5.040092 -0.515492

H -4.172868 -4.072401 0.799023

H -1.946561 -4.048029 1.568796

H -1.866581 -1.732151 1.162704

C 0.567318 0.479006 1.201561

O 1.738452 0.784388 1.331239

C -0.312743 0.068606 2.355482

C -1.565872 0.659451 2.544736

C -2.380506 0.270225 3.610716

C -1.930645 -0.694836 4.503007

C -0.663824 -1.264827 4.349520

C 0.137764 -0.871030 3.285086

O -2.062032 1.629817 1.712262

H -3.345951 0.748547 3.731888

H -2.564117 -0.989448 5.333543

H -0.303457 -1.998954 5.062046

H 1.132505 -1.284189 3.155279

C -1.149366 2.224255 0.781114

C -0.417051 3.386518 1.541556

C -1.966000 2.822967 -0.359884

O -0.267401 1.301414 0.244373

F -1.302524 4.340508 1.898668

F 0.188963 2.977023 2.668097

F 0.508302 3.957826 0.756003

C -1.350622 3.046325 -1.593759

C -2.075101 3.605280 -2.643661

C -3.414890 3.946801 -2.468035

C -4.026862 3.728461 -1.235466

C -3.305070 3.172796 -0.181798

H -0.311446 2.773614 -1.726011

H -1.589496 3.777035 -3.599000

H -3.977798 4.385205 -3.286145

H -5.069135 3.993968 -1.090269

H -3.780711 3.002669 0.775202

PC_2-A-minor_

C 0.076386 1.404723 0.241532

N -0.869576 2.221518 -0.279435

N -0.447966 3.509259 -0.633127

C 0.809748 3.467800 -0.331798

N 1.157417 2.242676 0.193549

C 2.535417 1.949313 0.563060

C 1.854327 4.530019 -0.364071

C -2.235056 1.883582 -0.464226

C -2.680672 1.310621 -1.659748

C -4.013992 0.969516 -1.851767

C -4.915496 1.212661 -0.823965

C -4.517062 1.785603 0.375640

C -3.178417 2.117267 0.541585

Cl -1.550138 1.007736 -2.955532

H -4.337654 0.524796 -2.782562

Cl -6.600339 0.785846 -1.049152

H -5.227626 1.963910 1.170499

Cl -2.682483 2.837744 2.050451

O 2.580718 4.458578 0.862570

H 2.517743 4.411129 -1.230938

H 1.401504 5.519082 -0.406261

C 3.262156 3.229883 1.129089

C 3.400334 1.517392 -0.607692

C 4.686771 3.197055 0.516002

C 4.604742 2.219509 -0.630963

C 5.546202 1.957988 -1.624478

C 5.263593 0.989827 -2.587117

C 4.055992 0.287222 -2.556552

C 3.113342 0.546095 -1.563100

H 6.485501 2.502513 -1.655144

H 5.987398 0.781413 -3.368812

H 3.847226 -0.465327 -3.309461

H 2.174160 0.003661 -1.536508

H 4.987876 4.204437 0.215874

H 5.413990 2.862606 1.264984

H 3.296020 3.189734 2.217798

H 2.494648 1.170365 1.327189

C -1.436428 -1.422524 1.451590

O -2.525797 -0.926957 1.437543

C -0.450176 -1.333836 2.536828

C 0.820194 -1.881591 2.358368

C 1.794300 -1.765162 3.344800

C 1.479133 -1.099500 4.525217

C 0.208038 -0.551985 4.723451

C -0.751279 -0.668442 3.727745

O 1.161281 -2.505991 1.186883

H 2.772264 -2.202105 3.179778

H 2.232259 -1.013401 5.301814

H -0.025223 -0.036154 5.647997

H -1.740442 -0.240996 3.840538

C 0.114962 -2.965788 0.351121

C -0.345726 -4.368288 0.872310

C 0.659129 -3.086397 -1.063050

O -1.022705 -2.128883 0.332412

F 0.694712 -5.214022 0.951807

F -0.894987 -4.284802 2.100925

F -1.261666 -4.902031 0.053511

C -0.169900 -2.837410 -2.157824

C 0.328508 -2.974338 -3.450861

C 1.649607 -3.365346 -3.656779

C 2.475782 -3.616087 -2.562810

C 1.983475 -3.480579 -1.267887

H -1.192324 -2.523436 -1.995440

H -0.319423 -2.773790 -4.297553

H 2.033843 -3.475492 -4.665860

H 3.506474 -3.918966 -2.715107

H 2.628581 -3.670129 -0.419689

RC_2-B_

C 4.604742 2.219509 -0.630963

N -3.230141 -0.241283 -1.758789

N -2.201127 0.362975 -1.048114

C -1.491108 -0.636272 -0.633092

N -2.017274 -1.840351 -1.037000

C -1.397728 -3.102084 -0.635387

C -0.341153 -0.695978 0.313939

C -4.189129 0.568889 -2.427250

C -3.849905 1.273650 -3.593326

C -4.794162 2.053826 -4.252843

C -6.078298 2.143937 -3.733098

C -6.434593 1.498321 -2.556958

C -5.480228 0.727797 -1.902820

Cl -2.239390 1.227955 -4.235235

H -4.522550 2.581649 -5.156482

Cl -7.270560 3.115848 -4.567367

H -7.428456 1.599811 -2.143829

Cl -5.914696 -0.006039 -0.385891

O -0.654790 -1.674848 1.303329

H 0.591726 -0.930351 -0.211557

H -0.227086 0.253577 0.833675

C -0.931442 -3.013586 0.860351

C -0.125727 -3.452455 -1.391730

C 0.324928 -3.908745 0.917952

C 0.841627 -3.924984 -0.501879

C 2.082480 -4.337989 -0.977846

C 2.338347 -4.262448 -2.347148

C 1.369280 -3.782109 -3.231230

C 0.120373 -3.373011 -2.760298

H 2.843923 -4.704142 -0.295309

H 3.304809 -4.574137 -2.730924

H 1.587809 -3.722262 -4.291441

H -0.630763 -2.979914 -3.439195

H 1.035814 -3.514575 1.648039

H 0.050340 -4.917931 1.248851

H -1.720951 -3.356172 1.529399

H -2.158018 -3.878797 -0.735387

C -4.223758 -2.633945 -2.009457

O -4.735826 -3.027625 -0.964754

C -4.489974 -3.005294 -3.354684

C -3.527648 -2.566576 -4.336115

C -3.797218 -2.931557 -5.697749

C -4.899339 -3.688550 -6.011143

C -5.817629 -4.137457 -5.023976

C -5.605210 -3.800267 -3.710907

O -2.496488 -1.913996 -3.971804

H -3.094500 -2.607590 -6.457316

H -5.073782 -3.960860 -7.049169

H -6.674501 -4.735794 -5.313409

H -6.282099 -4.122572 -2.925548

C 1.598988 -2.910879 -7.667477

C 2.104519 -2.268783 -8.906117

O 2.279900 -3.108710 -6.688816

C 0.109366 -3.369357 -7.615602

C 3.454710 -1.883438 -8.917863

C 4.002365 -1.279664 -10.039829

C 3.208320 -1.052309 -11.165400

C 1.867406 -1.430533 -11.163071

C 1.312526 -2.036490 -10.040859

H 4.053675 -2.068575 -8.034180

H 5.046282 -0.984995 -10.040963

H 3.635799 -0.579836 -12.043843

H 1.250620 -1.253354 -12.037421

H 0.269574 -2.322307 -10.057669

F -0.144593 -4.277777 -8.583798

F -0.182808 -3.922215 -6.443035

F -0.723683 -2.323138 -7.809607

TS1_2-B_

C -1.995982 -1.850438 -2.999923

N -2.013216 -0.521804 -3.226612

N -1.072718 0.146385 -2.473115

C -0.496916 -0.795667 -1.791548

N -1.033686 -2.027825 -2.061343

C -0.619281 -3.191465 -1.243439

C 0.440177 -0.692043 -0.638897

C -2.954575 0.263750 -3.958446

C -2.553480 1.011319 -5.073697

C -3.477110 1.779624 -5.774522

C -4.794698 1.820625 -5.340651

C -5.207886 1.142483 -4.200772

C -4.276377 0.382270 -3.507686

Cl -0.902109 1.044769 -5.584348

H -3.161304 2.337563 -6.644976

Cl -5.957805 2.776659 -6.229914

H -6.227552 1.210498 -3.848699

Cl -4.783847 -0.380530 -2.021099

O -0.195425 -1.359946 0.447799

H 1.421274 -1.119210 -0.871825

H 0.561937 0.349388 -0.347360

C -0.522583 -2.747604 0.262519

C 0.756266 -3.779115 -1.532369

C 0.571239 -3.654685 0.856002

C 1.420159 -4.040878 -0.330038

C 2.677350 -4.637655 -0.336708

C 3.258918 -4.976207 -1.557850

C 2.578759 -4.740663 -2.753773

C 1.315482 -4.148002 -2.753318

H 3.197715 -4.839518 0.594847

H 4.241562 -5.436775 -1.577270

H 3.030452 -5.028570 -3.697362

H 0.767984 -3.989213 -3.674215

H 1.110573 -3.127179 1.646029

H 0.117430 -4.543066 1.311944

H -1.485495 -2.866760 0.759792

H -1.403418 -3.934476 -1.360912

C -3.029659 -2.912172 -3.359560

O -3.186319 -3.749255 -2.480891

C -3.917709 -2.809911 -4.521422

C -3.571507 -2.296245 -5.806851

C -4.580740 -2.287507 -6.802151

C -5.850474 -2.763513 -6.554236

C -6.174237 -3.309759 -5.300796

C -5.211991 -3.343280 -4.315461

O -2.377664 -1.842328 -6.125865

H -4.305089 -1.900394 -7.776258

H -6.598604 -2.733075 -7.341057

H -7.168162 -3.699141 -5.109456

H -5.434635 -3.765165 -3.342938

C -0.948688 -2.938400 -5.954636

C 0.074210 -2.076715 -6.677086

O -0.796473 -3.193859 -4.733549

C -1.415162 -4.174275 -6.789549

C 1.275944 -1.808430 -6.019128

C 2.265226 -1.039547 -6.627216

C 2.061327 -0.526409 -7.906026

C 0.863250 -0.789089 -8.569230

C -0.124383 -1.558311 -7.961308

H 1.425786 -2.205410 -5.022938

H 3.195856 -0.843958 -6.103118

H 2.830405 0.072386 -8.384224

H 0.694432 -0.388348 -9.564002

H -1.057803 -1.739491 -8.475132

F -0.336709 -4.964661 -7.010206

F -2.316718 -4.911136 -6.124678

F -1.943507 -3.889696 -8.000401

Int1_2-B_

C -2.004838 -1.853952 -3.012353

N -2.078574 -0.518439 -3.215318

N -1.163361 0.179500 -2.440770

C -0.567968 -0.753642 -1.761101

N -1.064249 -2.003077 -2.043283

C -0.625295 -3.171639 -1.244742

C 0.368053 -0.632086 -0.607070

C -3.044813 0.242849 -3.934356

C -2.673077 1.011455 -5.049803

C -3.619614 1.773536 -5.733730

C -4.935649 1.785602 -5.282279

C -5.324284 1.082419 -4.144263

C -4.367166 0.333677 -3.467583

Cl -1.027963 1.066119 -5.586738

H -3.323885 2.349071 -6.601879

Cl -6.126486 2.731479 -6.145118

H -6.342909 1.126349 -3.780518

Cl -4.840105 -0.463881 -1.987384

O -0.249092 -1.334671 0.466981

H 1.363199 -1.029342 -0.847778

H 0.461078 0.411539 -0.303169

C -0.461100 -2.739203 0.265967

C 0.728326 -3.767836 -1.609152

C 0.734460 -3.569752 0.786741

C 1.489697 -3.981206 -0.453786

C 2.749505 -4.573626 -0.540297

C 3.231722 -4.958797 -1.792875

C 2.451381 -4.775586 -2.939453

C 1.186467 -4.187304 -2.859283

H 3.345550 -4.737541 0.354291

H 4.214320 -5.416175 -1.874515

H 2.826872 -5.100099 -3.905931

H 0.562864 -4.061986 -3.739655

H 1.319776 -2.977674 1.498677

H 0.370430 -4.453109 1.329359

H -1.379376 -2.947360 0.819364

H -1.423763 -3.906074 -1.344621

C -2.921614 -2.973664 -3.469288

O -3.046973 -3.905127 -2.680407

C -3.866556 -2.806640 -4.609440

C -3.545888 -2.370253 -5.913529

C -4.561096 -2.283667 -6.878892

C -5.871411 -2.626180 -6.572359

C -6.191679 -3.102739 -5.296302

C -5.190629 -3.205548 -4.340724

O -2.308285 -1.989511 -6.324343

H -4.279658 -1.956813 -7.874530

H -6.640706 -2.543837 -7.335740

H -7.208898 -3.396173 -5.054672

H -5.413121 -3.588011 -3.350431

C -1.117504 -2.825196 -5.955450

C 0.053266 -2.067908 -6.605813

O -1.009533 -3.022943 -4.648040

C -1.306901 -4.204830 -6.669890

C 1.211604 -1.836460 -5.861260

C 2.292670 -1.154864 -6.423323

C 2.225296 -0.697126 -7.739794

C 1.069920 -0.927247 -8.490219

C -0.008543 -1.610047 -7.928515

H 1.253193 -2.186535 -4.836719

H 3.188173 -0.982006 -5.831275

H 3.066131 -0.166162 -8.179109

H 1.006934 -0.572866 -9.516099

H -0.904993 -1.779715 -8.512990

F -0.161723 -4.916720 -6.587571

F -2.279230 -4.938585 -6.095648

F -1.620366 -4.093220 -7.983324

TS2_2-B_

C -1.630789 -1.882962 -2.966624

N -1.757297 -0.544018 -2.966669

N -1.487050 0.051536 -1.739898

C -1.177400 -0.961797 -0.994792

N -1.254213 -2.148308 -1.691753

C -0.868901 -3.424851 -1.055414

C -0.891870 -1.025265 0.466223

C -2.055650 0.293262 -4.076900

C -1.020294 0.838255 -4.844866

C -1.288140 1.651608 -5.938274

C -2.612871 1.935987 -6.245772

C -3.664161 1.445988 -5.481646

C -3.375032 0.631079 -4.392922

Cl 0.637265 0.526215 -4.416021

H -0.479610 2.043732 -6.538800

Cl -2.967614 2.951900 -7.625897

H -4.689092 1.693369 -5.720762

Cl -4.686741 0.052840 -3.406237

O -1.625478 -2.128902 0.983716

H 0.184273 -1.124831 0.658319

H -1.254655 -0.128797 0.966003

C -1.226357 -3.407884 0.483759

C 0.626565 -3.670818 -1.095792

C 0.029690 -3.959225 1.208256

C 1.128089 -3.950097 0.174679

C 2.485266 -4.216925 0.346785

C 3.322770 -4.204987 -0.766847

C 2.808701 -3.948705 -2.041146

C 1.452217 -3.685330 -2.217912

H 2.886808 -4.434744 1.332099

H 4.381985 -4.408773 -0.645347

H 3.469292 -3.966078 -2.901760

H 1.033898 -3.530066 -3.205693

H 0.247539 -3.352982 2.092401

H -0.158207 -4.976500 1.568543

H -2.099933 -4.032480 0.665678

H -1.401281 -4.210790 -1.586928

C -1.874068 -3.330653 -4.263144

O -1.358506 -4.325893 -3.770583

C -3.351609 -3.249876 -4.554361

C -3.809203 -2.775562 -5.784628

C -5.173625 -2.723283 -6.069751

C -6.088255 -3.165439 -5.123127

C -5.646676 -3.675131 -3.899845

C -4.285277 -3.722093 -3.630392

O -2.951943 -2.348357 -6.769787

H -5.492139 -2.356296 -7.038982

H -7.149360 -3.128274 -5.347392

H -6.360505 -4.040058 -3.169231

H -3.923957 -4.135552 -2.694846

C -1.581815 -2.724854 -6.647635

C -0.761090 -1.784544 -7.524800

O -1.115071 -2.630866 -5.340462

C -1.443744 -4.183775 -7.212798

C 0.605317 -1.632928 -7.283101

C 1.369532 -0.795841 -8.092018

C 0.776198 -0.106046 -9.147284

C -0.586975 -0.260171 -9.391995

C -1.353226 -1.100678 -8.588489

H 1.062552 -2.160860 -6.456668

H 2.430695 -0.682249 -7.895001

H 1.373455 0.544652 -9.778352

H -1.057359 0.272516 -10.212103

H -2.411228 -1.220646 -8.780763

F -0.181460 -4.623109 -7.085193

F -2.250224 -5.065225 -6.598564

F -1.760145 -4.209622 -8.523618

PC_2-B_

C -0.223282 -1.374122 -0.243630

N -1.260193 -2.163467 0.129097

N -1.058295 -3.546176 0.020465

C 0.158132 -3.598558 -0.419063

N 0.683909 -2.337337 -0.592551

C 2.060751 -2.159798 -1.038385

C 0.990845 -4.761008 -0.840551

C -2.518658 -1.700684 0.586366

C -2.717169 -1.345514 1.925989

C -3.947994 -0.887399 2.383925

C -5.001054 -0.797207 1.483600

C -4.851790 -1.151686 0.149321

C -3.609771 -1.600569 -0.285603

Cl -1.407963 -1.480399 3.069913

H -4.078020 -0.614420 3.422015

Cl -6.559541 -0.224023 2.045736

H -5.680815 -1.081467 -0.541182

Cl -3.427077 -2.038320 -1.963144

O 1.619540 -4.422873 -2.075061

H 1.729228 -5.018541 -0.069758

H 0.368879 -5.634157 -1.030982

C 2.476557 -3.274075 -2.068400

C 3.078576 -2.291780 0.080050

C 3.939556 -3.635052 -1.703859

C 4.134808 -3.120669 -0.297458

C 5.185590 -3.358622 0.586068

C 5.161131 -2.760005 1.845378

C 4.101244 -1.928662 2.216981

C 3.050194 -1.686582 1.333148

H 6.010841 -4.006108 0.304226

H 5.971062 -2.944063 2.544326

H 4.093418 -1.471076 3.201035

H 2.223588 -1.042422 1.615049

H 4.097696 -4.711914 -1.807004

H 4.632441 -3.145237 -2.397839

H 2.398033 -2.889663 -3.084839

H 2.118398 -1.179520 -1.513253

C 0.832927 1.414913 -2.026579

O 1.391003 0.625217 -2.737693

C -0.554918 1.871247 -2.154134

C -1.096371 2.701277 -1.171447

C -2.426513 3.102753 -1.228461

C -3.212560 2.672705 -2.291962

C -2.683879 1.848283 -3.290055

C -1.358577 1.446923 -3.216170

O -0.345821 3.108512 -0.099513

H -2.821973 3.744109 -0.449769

H -4.249423 2.988155 -2.344567

H -3.308434 1.518561 -4.112245

H -0.921616 0.795148 -3.963212

C 1.061426 3.040891 -0.210087

C 1.650972 2.980887 1.188993

O 1.530458 1.925354 -0.947808

C 1.553726 4.330828 -0.948092

C 2.878918 2.352243 1.403588

C 3.430421 2.324394 2.681659

C 2.764803 2.926414 3.747038

C 1.541709 3.557133 3.530747

C 0.985527 3.589344 2.255166

H 3.392687 1.876348 0.578687

H 4.382286 1.828903 2.841063

H 3.196868 2.903596 4.742348

H 1.016313 4.025692 4.356381

H 0.032298 4.074757 2.090693

F 2.891957 4.368146 -1.005917

F 1.090041 4.386611 -2.213491

F 1.126124 5.431871 -0.311337

RC_2-B-minor_

C -2.047209 0.403006 0.540269

N -2.513205 -0.343502 -0.485285

N -2.983522 0.436936 -1.530974

C -2.816169 1.648578 -1.106301

N -2.272581 1.674903 0.155592

C -2.036962 2.944268 0.843262

C -3.253000 2.956350 -1.673557

C -2.489667 -1.759381 -0.628789

C -1.558853 -2.378209 -1.478274

C -1.533714 -3.762914 -1.612132

C -2.454236 -4.527569 -0.908624

C -3.416542 -3.944067 -0.095510

C -3.437020 -2.559599 0.025271

Cl -0.428740 -1.447173 -2.404148

H -0.804366 -4.229071 -2.259905

Cl -2.415947 -6.269679 -1.068501

H -4.147338 -4.546802 0.425349

Cl -4.698154 -1.834691 0.981286

O -3.943506 3.657776 -0.640833

H -2.398343 3.526634 -2.055073

H -3.965362 2.800882 -2.481725

C -3.231688 3.927106 0.577634

C -0.821565 3.706286 0.341859

C -2.574597 5.324901 0.564944

C -1.131753 5.060778 0.202790

C -0.155039 5.958002 -0.218552

C 1.123483 5.477447 -0.504109

C 1.423138 4.119549 -0.373526

C 0.449626 3.216067 0.055202

H -0.384186 7.013444 -0.333397

H 1.892406 6.166359 -0.839888

H 2.415595 3.750658 -0.603551

H 0.672394 2.156271 0.135725

H -3.098066 5.980701 -0.134830

H -2.654982 5.784908 1.557559

H -3.990345 3.828362 1.354072

H -1.973426 2.724135 1.910126

C -1.894020 -0.000804 2.021089

O -2.857613 0.328575 2.710461

C -0.699471 -0.674343 2.390264

C 0.379114 -0.668431 1.427686

C 1.560049 -1.398549 1.802054

C 1.654035 -2.001852 3.030924

C 0.599024 -1.949614 3.982077

C -0.558954 -1.289345 3.658456

O 0.267614 -0.031158 0.334885

H 2.373414 -1.442123 1.085934

H 2.566179 -2.534405 3.288808

H 0.711998 -2.436122 4.944674

H -1.391880 -1.233293 4.352589

C 4.252193 0.734393 -0.669341

C 5.254221 -0.184231 -0.072305

O 3.993691 1.825278 -0.219589

C 3.500808 0.284512 -1.957999

C 5.818076 0.199755 1.154762

C 6.768584 -0.600924 1.770675

C 7.172961 -1.792346 1.165839

C 6.622519 -2.179268 -0.054333

C 5.664698 -1.383473 -0.674019

H 5.490855 1.128230 1.607090

H 7.195243 -0.300407 2.721601

H 7.917233 -2.418907 1.646698

H 6.938588 -3.103854 -0.525145

H 5.249363 -1.698390 -1.621705

F 2.559453 1.158908 -2.300031

F 4.374267 0.187264 -2.991383

F 2.924811 -0.923504 -1.803991

TS1_2-B-minor_

C -1.992496 -1.826204 -2.975039

N -2.084121 -0.500386 -3.206310

N -1.161218 0.219012 -2.477733

C -0.515623 -0.690707 -1.814254

N -0.989822 -1.949921 -2.076469

C -0.492605 -3.092147 -1.280063

C 0.438675 -0.545222 -0.680053

C -3.094812 0.233051 -3.902001

C -2.788420 0.998257 -5.036431

C -3.784292 1.713635 -5.693908

C -5.080844 1.686303 -5.200375

C -5.402777 0.989773 -4.042789

C -4.400253 0.284471 -3.391982

Cl -1.171841 1.114268 -5.638968

H -3.538957 2.285346 -6.578128

Cl -6.333011 2.574899 -6.035876

H -6.407043 1.003666 -3.643217

Cl -4.800580 -0.500977 -1.885429

O -0.135761 -1.256660 0.414992

H 1.436200 -0.914605 -0.938421

H 0.508184 0.498540 -0.379772

C -0.410513 -2.656892 0.227253

C 0.921282 -3.568507 -1.583483

C 0.726327 -3.519228 0.805068

C 1.602335 -3.815795 -0.387577

C 2.901723 -4.312123 -0.410193

C 3.510757 -4.556434 -1.641230

C 2.818830 -4.325843 -2.831253

C 1.512168 -3.838143 -2.813372

H 3.436206 -4.504620 0.515376

H 4.527473 -4.935272 -1.672771

H 3.294994 -4.530646 -3.784265

H 0.953175 -3.696076 -3.726575

H 1.229999 -2.987088 1.615111

H 0.318253 -4.445219 1.228824

H -1.364746 -2.818043 0.729573

H -1.223727 -3.887963 -1.401093

C -2.994706 -2.933410 -3.289307

O -3.170029 -3.712802 -2.365127

C -3.858352 -2.877045 -4.475706

C -3.451391 -2.382881 -5.742507

C -4.402321 -2.360983 -6.784037

C -5.694785 -2.808951 -6.583828

C -6.082720 -3.333917 -5.342162

C -5.166172 -3.376906 -4.310173

O -2.215237 -1.965396 -5.982529

H -4.077671 -1.990669 -7.749276

H -6.407808 -2.772339 -7.402036

H -7.092705 -3.699752 -5.192107

H -5.438067 -3.779200 -3.341425

C -1.040926 -3.206973 -5.785678

C -1.362315 -4.197906 -6.899455

O -1.070820 -3.635838 -4.589880

C 0.209192 -2.321211 -6.083758

C -1.559645 -5.531031 -6.534803

C -1.855671 -6.492495 -7.497901

C -1.957968 -6.130168 -8.838677

C -1.767843 -4.799564 -9.209346

C -1.474508 -3.837141 -8.247348

H -1.476297 -5.794307 -5.487405

H -2.005140 -7.525671 -7.199705

H -2.186472 -6.878413 -9.591485

H -1.851490 -4.508517 -10.252034

H -1.341343 -2.804914 -8.543648

F 0.466548 -1.476961 -5.059089

F 1.295761 -3.125826 -6.199054

F 0.157403 -1.576741 -7.203547

Int1_2-B-minor_

C -1.993694 -1.846741 -2.988973

N -2.094950 -0.517219 -3.201803

N -1.192878 0.201503 -2.439319

C -0.557580 -0.712510 -1.774145

N -1.014719 -1.972309 -2.063932

C -0.507085 -3.126947 -1.294244

C 0.380300 -0.577416 -0.624578

C -3.113036 0.221240 -3.880003

C -2.817315 1.014604 -4.997740

C -3.818855 1.743738 -5.631392

C -5.112396 1.699509 -5.131281

C -5.425498 0.971532 -3.990588

C -4.416317 0.255564 -3.362374

Cl -1.204748 1.143794 -5.610307

H -3.580585 2.338616 -6.502204

Cl -6.372545 2.604260 -5.936990

H -6.427942 0.970510 -3.586174

Cl -4.803445 -0.569769 -1.873989

O -0.192810 -1.325086 0.446048

H 1.387251 -0.922928 -0.880300

H 0.428441 0.459957 -0.298926

C -0.421426 -2.728104 0.223669

C 0.909120 -3.578649 -1.620004

C 0.745942 -3.571346 0.770945

C 1.610552 -3.833366 -0.438070

C 2.917994 -4.306621 -0.487807

C 3.512833 -4.520057 -1.731562

C 2.800119 -4.281681 -2.907951

C 1.485796 -3.816954 -2.862972

H 3.469604 -4.505258 0.426392

H 4.535320 -4.880730 -1.783816

H 3.265932 -4.461901 -3.870988

H 0.909334 -3.665667 -3.764431

H 1.249758 -3.038150 1.580383

H 0.366575 -4.511761 1.189379

H -1.364712 -2.931681 0.731058

H -1.231435 -3.926075 -1.434903

C -2.943979 -2.971320 -3.377595

O -3.119517 -3.811459 -2.509146

C -3.841434 -2.841394 -4.551034

C -3.445242 -2.358499 -5.815687

C -4.399517 -2.258417 -6.837074

C -5.717254 -2.628923 -6.616260

C -6.105646 -3.149098 -5.377866

C -5.168819 -3.263187 -4.364399

O -2.175851 -1.987749 -6.087826

H -4.070262 -1.896374 -7.803775

H -6.442015 -2.536226 -7.419091

H -7.130710 -3.461522 -5.210185

H -5.442715 -3.669547 -3.398104

C -1.117314 -3.097383 -5.828931

C -1.365879 -4.201033 -6.868683

O -1.129085 -3.491615 -4.583238

C 0.192637 -2.303599 -6.148600

C -1.484565 -5.515783 -6.419115

C -1.718146 -6.552422 -7.320440

C -1.834812 -6.282988 -8.681765

C -1.721757 -4.969979 -9.136895

C -1.490958 -3.933539 -8.236618

H -1.395246 -5.703942 -5.356399

H -1.808789 -7.571693 -6.957540

H -2.014765 -7.090100 -9.385378

H -1.816009 -4.751473 -10.196311

H -1.411047 -2.915646 -8.597319

F 0.478171 -1.414733 -5.170150

F 1.238559 -3.160555 -6.207639

F 0.180683 -1.617136 -7.308909

TS2_2-B-minor_

C -1.957229 -1.819246 -2.997056

N -2.332476 -0.542295 -2.786739

N -1.845512 0.002205 -1.599036

C -1.152944 -0.973307 -1.102551

N -1.192496 -2.084989 -1.910942

C -0.467962 -3.302264 -1.521272

C -0.472952 -1.107774 0.217309

C -3.147955 0.296238 -3.598243

C -2.591537 1.044855 -4.641877

C -3.372965 1.907568 -5.402246

C -4.721963 2.030908 -5.097587

C -5.303097 1.322493 -4.053695

C -4.506877 0.463089 -3.307179

Cl -0.895919 0.919812 -5.000888

H -2.930510 2.473540 -6.210202

Cl -5.715705 3.112006 -6.049719

H -6.352982 1.434051 -3.821348

Cl -5.230313 -0.401187 -1.979502

O -0.883329 -2.350367 0.780260

H 0.616644 -1.046891 0.109084

H -0.803685 -0.324586 0.897340

C -0.587186 -3.536946 0.026253

C 1.028286 -3.227443 -1.764729

C 0.774095 -4.149365 0.429455

C 1.727183 -3.714254 -0.658527

C 3.118232 -3.766967 -0.679392

C 3.795365 -3.325041 -1.816199

C 3.089947 -2.842923 -2.920825

C 1.696487 -2.793333 -2.905182

H 3.671471 -4.141414 0.176910

H 4.879971 -3.357272 -1.842803

H 3.626576 -2.510153 -3.803040

H 1.147064 -2.431935 -3.765085

H 1.056014 -3.816958 1.431604

H 0.696750 -5.242639 0.464623

H -1.421454 -4.202082 0.247833

H -0.935434 -4.121766 -2.068264

C -2.421718 -3.296941 -4.138240

O -2.598509 -4.250839 -3.382270

C -3.527129 -2.774848 -5.020200

C -3.254424 -2.221824 -6.273279

C -4.278692 -1.712537 -7.069374

C -5.591242 -1.779817 -6.615615

C -5.885308 -2.376198 -5.386942

C -4.854413 -2.875835 -4.600474

O -1.971607 -2.189275 -6.762212

H -4.033093 -1.293227 -8.038623

H -6.390841 -1.388494 -7.236363

H -6.913364 -2.454631 -5.050092

H -5.053926 -3.354556 -3.648866

C -1.157759 -3.257610 -6.263842

C -1.564005 -4.594131 -6.889951

O -1.117275 -3.277206 -4.872963

C 0.280276 -2.865733 -6.703523

C -1.469889 -5.773403 -6.149532

C -1.802676 -6.992969 -6.734689

C -2.224482 -7.044126 -8.060576

C -2.315415 -5.868147 -8.803470

C -1.984028 -4.648189 -8.222654

H -1.162962 -5.726647 -5.113387

H -1.736640 -7.903817 -6.148448

H -2.485020 -7.995512 -8.513662

H -2.646010 -5.899925 -9.836723

H -2.053768 -3.735259 -8.801319

F 0.677348 -1.706980 -6.142679

F 1.160642 -3.811436 -6.336579

F 0.363949 -2.714692 -8.035860

PC_2-B-minor_

C 0.793748 -1.366821 -0.454979

N 0.488353 -2.612490 -0.018045

N 1.556126 -3.514913 0.082868

C 2.554303 -2.779992 -0.290198

N 2.145497 -1.506409 -0.619034

C 3.115576 -0.500913 -1.037972

C 3.986719 -3.124885 -0.514095

C -0.811595 -3.068673 0.312650

C -1.304700 -2.961897 1.618609

C -2.579186 -3.406732 1.954248

C -3.367396 -3.975741 0.962933

C -2.913890 -4.110291 -0.342667

C -1.636562 -3.656915 -0.653356

Cl -0.317079 -2.254557 2.867563

H -2.942278 -3.310872 2.968162

Cl -4.976909 -4.540696 1.370153

H -3.535525 -4.559871 -1.104551

Cl -1.069332 -3.832983 -2.292669

O 4.376707 -2.566544 -1.768070

H 4.615967 -2.752943 0.304937

H 4.118704 -4.202706 -0.593313

C 4.262561 -1.143928 -1.903109

C 3.851402 0.158380 0.114910

C 5.555963 -0.410361 -1.466038

C 5.222982 0.211889 -0.131014

C 6.075598 0.808430 0.795538

C 5.536430 1.347346 1.963246

C 4.160624 1.295100 2.201167

C 3.305720 0.699730 1.274971

H 7.146051 0.851928 0.616205

H 6.191292 1.811821 2.693918

H 3.752096 1.724540 3.110144

H 2.237161 0.660119 1.452613

H 6.392680 -1.113303 -1.432180

H 5.818927 0.360077 -2.200450

H 4.058735 -1.002631 -2.964513

H 2.570847 0.242986 -1.621567

C -0.035338 1.773652 -1.571676

O 0.912054 1.723138 -2.307058

C -1.414162 1.388791 -1.902598

C -2.374601 1.334851 -0.889040

C -3.658854 0.865592 -1.145157

C -3.982378 0.466049 -2.436714

C -3.039015 0.530974 -3.467864

C -1.757144 0.983440 -3.196847

O -2.059973 1.717805 0.387198

H -4.379736 0.822242 -0.337178

H -4.984275 0.104385 -2.643576

H -3.305945 0.216407 -4.470307

H -0.994619 1.019294 -3.965857

C -0.966768 2.611766 0.485679

C -1.367388 4.041088 0.134382

O 0.157390 2.190763 -0.268909

C -0.507636 2.490984 1.964803

C -0.422913 4.926133 -0.391225

C -0.784391 6.239724 -0.679200

C -2.084160 6.677850 -0.437663

C -3.024240 5.798919 0.096776

C -2.667782 4.485578 0.387144

H 0.588192 4.585512 -0.577389

H -0.047562 6.919770 -1.093454

H -2.364214 7.701523 -0.664154

H -4.037620 6.135602 0.288904

H -3.397549 3.803913 0.806501

F -0.152761 1.236262 2.269308

F 0.545996 3.289353 2.196044

F -1.499414 2.854043 2.794258

RC_3-A_

C -1.109136 -1.607405 -3.170643

N -0.282286 -1.438257 -2.127245

N 0.030513 -0.109163 -1.910933

C -0.640121 0.513840 -2.830935

N -1.348352 -0.362880 -3.621317

C -2.251916 0.113794 -4.681136

C -0.658694 1.950522 -3.233754

C 0.216579 -2.450987 -1.256618

C -0.458550 -2.750259 -0.066073

C 0.006861 -3.751453 0.777822

C 1.160270 -4.443175 0.427968

C 1.865821 -4.150701 -0.733320

C 1.389811 -3.146950 -1.567381

Cl -1.878822 -1.865048 0.389852

H -0.526848 -3.984637 1.688648

Cl 1.745996 -5.707194 1.481415

H 2.768451 -4.688772 -0.987396

Cl 2.292789 -2.755059 -3.003447

O -0.555187 1.979468 -4.653080

H -1.567136 2.449589 -2.875287

H 0.210323 2.470756 -2.835058

C -1.634013 1.383167 -5.384530

C -3.584609 0.592539 -4.138150

C -2.826914 2.349255 -5.584778

C -3.902827 1.849904 -4.651328

C -5.106560 2.457099 -4.301336

C -5.975133 1.788128 -3.439855

C -5.652350 0.526338 -2.933137

C -4.447745 -0.083826 -3.278982

H -5.368200 3.435288 -4.693417

H -6.917861 2.249047 -3.163686

H -6.347323 0.008878 -2.281081

H -4.196918 -1.066715 -2.894831

H -2.511208 3.377859 -5.390755

H -3.164834 2.315673 -6.626491

H -1.172325 1.101775 -6.329822

H -2.365428 -0.707718 -5.390742

C -1.874774 -2.897399 -3.533015

O -2.726768 -3.123772 -2.669699

C -1.533182 -3.548877 -4.740344

C -2.261575 -4.765844 -5.163747

C -1.837128 -5.316174 -6.448140

C -0.835713 -4.768792 -7.189832

C -0.130513 -3.607775 -6.747964

C -0.477577 -3.033865 -5.559049

O -3.163595 -5.301623 -4.504050

H -2.371269 -6.201436 -6.777774

H -0.558233 -5.222708 -8.138124

H 0.676688 -3.199719 -7.346497

H 0.083058 -2.165790 -5.218493

O -7.308841 -2.413758 -1.348096

C -6.788308 -3.300745 -1.988827

C -6.093402 -4.460682 -1.394390

C -6.906548 -3.221399 -3.543689

F -7.364460 -4.378390 -4.057405

F -7.755747 -2.249513 -3.900747

F -5.716483 -2.953648 -4.118860

C -5.158279 -5.228929 -2.102098

C -4.512313 -6.282632 -1.461586

C -4.806089 -6.587561 -0.135919

C -5.735921 -5.820929 0.574227

C -6.366422 -4.754546 -0.046774

H -4.867443 -5.002019 -3.119085

H -3.771486 -6.847461 -2.015545

H -4.307774 -7.419635 0.352720

H -5.962643 -6.057890 1.608825

H -7.080174 -4.138574 0.488328

TS1_3-A_

C -1.181480 -1.788919 -2.827358

N -0.329398 -1.431143 -1.851189

N -0.207528 -0.067430 -1.735067

C -1.026679 0.390084 -2.636272

N -1.664615 -0.626281 -3.307069

C -2.666099 -0.280686 -4.338452

C -1.192246 1.770354 -3.181568

C 0.445033 -2.276859 -0.998711

C -0.055233 -2.670618 0.245738

C 0.695611 -3.476443 1.090408

C 1.961223 -3.880620 0.680840

C 2.491937 -3.492284 -0.543410

C 1.727147 -2.684675 -1.375261

Cl -1.631734 -2.148935 0.758640

H 0.295471 -3.784956 2.046176

Cl 2.911511 -4.898053 1.735082

H 3.479068 -3.810441 -0.848742

Cl 2.391993 -2.190275 -2.908584

O -1.088071 1.652165 -4.598674

H -2.136946 2.224325 -2.866949

H -0.366980 2.399907 -2.852978

C -2.066392 0.831338 -5.266648

C -3.925784 0.372241 -3.783445

C -3.290114 1.657229 -5.702787

C -4.276413 1.469073 -4.575136

C -5.418643 2.208377 -4.283554

C -6.198676 1.842084 -3.187439

C -5.846556 0.742520 -2.402803

C -4.709453 -0.008272 -2.698489

H -5.697578 3.060149 -4.896471

H -7.090187 2.412120 -2.946467

H -6.471327 0.457667 -1.562949

H -4.458412 -0.887827 -2.121928

H -3.003511 2.695897 -5.882566

H -3.692135 1.262917 -6.643797

H -1.524496 0.394688 -6.104517

H -2.900419 -1.191655 -4.884776

C -1.377781 -3.246570 -3.277064

O -1.257076 -4.084730 -2.410164

C -1.510769 -3.443101 -4.723576

C -2.432212 -4.406999 -5.253414

C -2.383824 -4.624600 -6.661595

C -1.497697 -3.952111 -7.474610

C -0.621532 -2.986743 -6.946277

C -0.647502 -2.736387 -5.587236

O -3.304806 -5.023946 -4.530439

H -3.083766 -5.347435 -7.066208

H -1.486182 -4.159987 -8.540966

H 0.080885 -2.466485 -7.588391

H 0.059508 -2.022734 -5.170380

O -3.812951 -2.815711 -3.168522

C -4.399041 -3.894770 -3.378074

C -4.607423 -4.895317 -2.270996

C -5.535316 -3.784741 -4.433306

F -6.040071 -4.944952 -4.882898

F -6.567564 -3.107949 -3.862059

F -5.146361 -3.066548 -5.504582

C -4.915684 -6.241964 -2.496522

C -5.122956 -7.100098 -1.421486

C -5.031151 -6.628090 -0.112488

C -4.717380 -5.290892 0.117513

C -4.502668 -4.432784 -0.957035

H -4.959525 -6.615771 -3.509175

H -5.351906 -8.144994 -1.606621

H -5.198160 -7.301398 0.722838

H -4.637587 -4.916248 1.133578

H -4.244904 -3.394186 -0.789282

Int1_3-A_

C -1.286832 -1.781317 -2.854165

N -0.443072 -1.451970 -1.861861

N -0.265388 -0.090871 -1.753759

C -1.033739 0.391515 -2.683921

N -1.691614 -0.603293 -3.367371

C -2.649027 -0.236287 -4.428307

C -1.145337 1.777535 -3.225639

C 0.259384 -2.310589 -0.960205

C -0.286649 -2.614473 0.289529

C 0.396128 -3.422803 1.188227

C 1.644005 -3.917061 0.828295

C 2.224727 -3.612513 -0.396935

C 1.526736 -2.801814 -1.281613

Cl -1.838252 -1.972532 0.744984

H -0.041545 -3.662167 2.147324

Cl 2.510639 -4.939531 1.949952

H 3.199260 -3.997979 -0.662487

Cl 2.261072 -2.407795 -2.812928

O -1.020778 1.666671 -4.641194

H -2.083435 2.255986 -2.925730

H -0.309999 2.382780 -2.877622

C -2.016973 0.886353 -5.325902

C -3.923403 0.406579 -3.902661

C -3.219019 1.750807 -5.755659

C -4.240915 1.532628 -4.665610

C -5.389760 2.265885 -4.384027

C -6.209094 1.858790 -3.331282

C -5.890198 0.726352 -2.579415

C -4.743634 -0.014984 -2.861611

H -5.645471 3.142445 -4.971751

H -7.107041 2.422895 -3.099993

H -6.547405 0.408210 -1.777231

H -4.510176 -0.919750 -2.316352

H -2.910653 2.791088 -5.883541

H -3.601638 1.405806 -6.723738

H -1.481462 0.455595 -6.171293

H -2.863713 -1.137221 -4.998497

C -1.531268 -3.229591 -3.298965

O -1.154176 -4.098747 -2.527974

C -1.616230 -3.421471 -4.788489

C -2.663309 -4.172752 -5.349625

C -2.666578 -4.421654 -6.727662

C -1.640175 -3.948103 -7.534713

C -0.601508 -3.195304 -6.986212

C -0.599718 -2.938722 -5.618421

O -3.663754 -4.708207 -4.623057

H -3.491981 -4.991412 -7.139158

H -1.655528 -4.161233 -8.598918

H 0.205716 -2.829108 -7.611440

H 0.221099 -2.376605 -5.181107

O -3.493071 -2.858225 -3.148674

C -4.182409 -3.959741 -3.431175

C -4.308723 -4.991758 -2.304470

C -5.602367 -3.532664 -3.927051

F -6.358176 -4.572538 -4.326737

F -6.283354 -2.898507 -2.946639

F -5.515038 -2.679334 -4.970430

C -4.519235 -6.348904 -2.560417

C -4.662658 -7.247726 -1.506553

C -4.609893 -6.799325 -0.188087

C -4.407545 -5.445686 0.070588

C -4.257855 -4.548172 -0.983474

H -4.554945 -6.700413 -3.583484

H -4.816053 -8.301862 -1.716494

H -4.723800 -7.501600 0.632197

H -4.361507 -5.087642 1.094815

H -4.083206 -3.497443 -0.790795

RC_3-A-minor_

C -0.854954 -1.251676 -2.988958

N -0.096146 -1.189459 -1.883852

N 0.347577 0.090275 -1.619400

C -0.178212 0.797314 -2.572940

N -0.915127 0.017843 -3.434830

C -1.671875 0.595304 -4.564210

C -0.009574 2.235489 -2.928154

C 0.242477 -2.268576 -1.016672

C -0.500771 -2.502879 0.147238

C -0.183144 -3.565826 0.984576

C 0.889052 -4.386015 0.655016

C 1.656387 -4.166474 -0.482975

C 1.327826 -3.100552 -1.310443

Cl -1.825181 -1.469171 0.573695

H -0.769223 -3.748079 1.874599

Cl 1.289535 -5.725284 1.702631

H 2.492888 -4.807671 -0.723497

Cl 2.306056 -2.804845 -2.722446

O 0.194676 2.287139 -4.334128

H -0.882125 2.823510 -2.616666

H 0.879300 2.645855 -2.452427

C -0.910458 1.857293 -5.135553

C -3.034658 1.116032 -4.152839

C -1.994702 2.952312 -5.301900

C -3.207159 2.437296 -4.567004

C -4.411094 3.092178 -4.317851

C -5.430114 2.405616 -3.658969

C -5.255356 1.078130 -3.260462

C -4.052964 0.418414 -3.504625

H -4.558222 4.120236 -4.635450

H -6.373310 2.905382 -3.462209

H -6.057337 0.536122 -2.773778

H -3.941773 -0.614797 -3.195213

H -1.621239 3.907965 -4.922314

H -2.213115 3.106275 -6.363961

H -0.444500 1.602161 -6.085829

H -1.741577 -0.188799 -5.318887

C -1.763222 -2.427116 -3.402979

O -2.551950 -2.680101 -2.491664

C -1.628095 -2.938161 -4.714879

C -2.664859 -3.841480 -5.259357

C -2.413101 -4.303233 -6.623157

C -1.315188 -3.929805 -7.336475

C -0.329414 -3.054429 -6.785930

C -0.496357 -2.585192 -5.513524

O -3.684735 -4.177007 -4.642154

H -3.161812 -4.970211 -7.038408

H -1.176860 -4.305810 -8.347480

H 0.549100 -2.790034 -7.364578

H 0.281328 -1.957223 -5.082108

O -6.064327 -2.177418 -3.255855

C -6.077201 -3.371189 -3.063467

C -6.652634 -4.350271 -4.018633

C -5.577920 -3.878980 -1.677341

F -4.658593 -4.848515 -1.752628

F -5.086593 -2.878853 -0.945911

F -6.644563 -4.389787 -0.995384

C -6.735047 -5.725255 -3.766371

C -7.308983 -6.572744 -4.708570

C -7.799184 -6.060461 -5.907431

C -7.714458 -4.691663 -6.167348

C -7.144694 -3.843825 -5.229210

H -6.348366 -6.142696 -2.846865

H -7.367543 -7.637434 -4.507767

H -8.243250 -6.726469 -6.640816

H -8.089348 -4.290633 -7.103486

H -7.061868 -2.779683 -5.413917

TS1_3-A-minor_

C -1.076665 -1.868848 -2.927074

N -0.245597 -1.473048 -1.945558

N -0.231699 -0.109251 -1.790008

C -1.089060 0.309356 -2.673577

N -1.648503 -0.733473 -3.372498

C -2.717521 -0.439248 -4.348389

C -1.381564 1.690511 -3.160135

C 0.591165 -2.272093 -1.105851

C 0.083493 -2.828107 0.073026

C 0.901170 -3.575023 0.910125

C 2.235678 -3.752439 0.562891

C 2.769487 -3.203364 -0.596838

C 1.937725 -2.460270 -1.424541

Cl -1.583782 -2.606342 0.497297

H 0.498900 -4.013418 1.812794

Cl 3.269750 -4.690865 1.612198

H 3.808543 -3.350819 -0.856577

Cl 2.598475 -1.777307 -2.886877

O -1.326584 1.634201 -4.583272

H -2.345441 2.056579 -2.793043

H -0.595675 2.371137 -2.836735

C -2.274045 0.771723 -5.239809

C -3.999367 0.058097 -3.694224

C -3.590104 1.509173 -5.554619

C -4.490787 1.163782 -4.392583

C -5.675213 1.775329 -3.991602

C -6.351657 1.272107 -2.880612

C -5.857124 0.163904 -2.189237

C -4.677497 -0.460091 -2.594119

H -6.065054 2.633924 -4.529930

H -7.273216 1.742721 -2.553398

H -6.401904 -0.226951 -1.336537

H -4.321004 -1.354065 -2.099027

H -3.405600 2.578601 -5.679912

H -4.009375 1.139947 -6.498232

H -1.751976 0.433407 -6.133976

H -2.890961 -1.344895 -4.925409

C -1.141758 -3.325231 -3.405712

O -0.876085 -4.156144 -2.567426

C -1.305129 -3.548857 -4.849371

C -2.124591 -4.622481 -5.311664

C -2.103794 -4.894498 -6.701368

C -1.321068 -4.158717 -7.572603

C -0.543861 -3.086892 -7.110951

C -0.549342 -2.787076 -5.758144

O -2.908329 -5.296482 -4.513511

H -2.729399 -5.704930 -7.057948

H -1.320800 -4.406094 -8.630246

H 0.073278 -2.516360 -7.796672

H 0.088260 -1.985963 -5.390754

O -3.659615 -3.096274 -3.498008

C -4.144608 -4.237384 -3.722945

C -5.283107 -4.358622 -4.727500

C -4.336520 -5.117524 -2.456350

F -4.552759 -6.423869 -2.695803

F -3.308605 -5.023322 -1.604149

F -5.435590 -4.654474 -1.795320

C -5.662982 -5.571853 -5.314157

C -6.720646 -5.613772 -6.217836

C -7.415576 -4.450078 -6.546429

C -7.043677 -3.240855 -5.965021

C -5.983527 -3.197392 -5.062078

H -5.114602 -6.472447 -5.075637

H -7.001967 -6.559983 -6.670073

H -8.241542 -4.487815 -7.250274

H -7.581745 -2.329734 -6.209572

H -5.694265 -2.263051 -4.597134

Int1_3-A-minor_

C -1.130045 -1.876975 -2.963212

N -0.297293 -1.466903 -1.987382

N -0.275025 -0.099508 -1.858931

C -1.123652 0.306389 -2.755668

N -1.685750 -0.745840 -3.437888

C -2.773179 -0.467381 -4.396592

C -1.419154 1.682328 -3.255063

C 0.528068 -2.249701 -1.121427

C 0.001806 -2.794189 0.054690

C 0.808536 -3.520762 0.919728

C 2.151899 -3.688887 0.603924

C 2.704775 -3.150096 -0.551517

C 1.883188 -2.427824 -1.407280

Cl -1.676563 -2.584542 0.440436

H 0.391199 -3.950392 1.819776

Cl 3.172989 -4.601741 1.688658

H 3.750693 -3.289817 -0.786834

Cl 2.569666 -1.759207 -2.864979

O -1.397296 1.610963 -4.677735

H -2.372860 2.057441 -2.869994

H -0.623721 2.363320 -2.956410

C -2.368533 0.749596 -5.298598

C -4.039715 0.013519 -3.704528

C -3.698882 1.485344 -5.560369

C -4.559476 1.123935 -4.372713

C -5.731938 1.724351 -3.921830

C -6.365138 1.204216 -2.792981

C -5.841935 0.089620 -2.133247

C -4.674766 -0.523393 -2.588060

H -6.145629 2.587203 -4.435014

H -7.276202 1.666772 -2.427058

H -6.355114 -0.314698 -1.267321

H -4.295508 -1.425579 -2.124004

H -3.522606 2.556810 -5.680750

H -4.148471 1.124546 -6.493112

H -1.880728 0.418140 -6.214464

H -2.950734 -1.380161 -4.961385

C -1.169805 -3.333342 -3.423815

O -0.821203 -4.153250 -2.602190

C -1.249753 -3.586567 -4.886624

C -2.088119 -4.618761 -5.359640

C -2.023571 -4.968169 -6.714392

C -1.147407 -4.317039 -7.575205

C -0.340600 -3.276547 -7.113226

C -0.398638 -2.915152 -5.770664

O -2.945346 -5.251590 -4.547072

H -2.677131 -5.756059 -7.070325

H -1.104234 -4.613183 -8.618739

H 0.342594 -2.768459 -7.785432

H 0.258286 -2.132662 -5.398766

O -3.464545 -3.138760 -3.539954

C -3.965186 -4.310865 -3.820076

C -5.200880 -4.301236 -4.741996

C -4.260557 -5.144911 -2.534705

F -4.630166 -6.420486 -2.780542

F -3.215413 -5.186954 -1.695942

F -5.284067 -4.561726 -1.859514

C -5.610948 -5.442026 -5.441269

C -6.730841 -5.399282 -6.267680

C -7.460183 -4.219032 -6.404321

C -7.061587 -3.082657 -5.705479

C -5.939273 -3.126018 -4.879977

H -5.045611 -6.359170 -5.340411

H -7.034935 -6.291656 -6.806595

H -8.333945 -4.187945 -7.048304

H -7.626308 -2.159560 -5.798365

H -5.630068 -2.247424 -4.330173

RC_3-B_

C -1.395746 -1.641033 -3.312953

N -0.997904 -1.571175 -2.034973

N -0.755378 -0.271440 -1.629902

C -1.035049 0.433250 -2.683070

N -1.428652 -0.363571 -3.733911

C -1.870183 0.199642 -5.019621

C -0.878228 1.889906 -2.963326

C -0.866045 -2.659835 -1.121971

C -1.933496 -3.011225 -0.287934

C -1.829025 -4.092940 0.575582

C -0.640491 -4.809816 0.609456

C 0.447020 -4.466967 -0.183780

C 0.325599 -3.387066 -1.045951

Cl -3.404384 -2.088534 -0.302989

H -2.666867 -4.374004 1.197769

Cl -0.505713 -6.180016 1.685291

H 1.367762 -5.031504 -0.141236

Cl 1.695410 -2.937258 -2.025468

O -0.274985 1.998214 -4.248355

H -1.844112 2.408056 -2.917472

H -0.198829 2.347512 -2.246655

C -1.039653 1.500616 -5.351684

C -3.314642 0.662605 -4.991722

C -2.080236 2.523728 -5.871583

C -3.424336 1.968738 -5.469014

C -4.674945 2.577256 -5.549438

C -5.801155 1.861883 -5.146605

C -5.683260 0.551783 -4.675054

C -4.436208 -0.064898 -4.595261

H -4.773879 3.593356 -5.919198

H -6.780504 2.326073 -5.202645

H -6.569519 0.004948 -4.371896

H -4.347469 -1.084474 -4.233132

H -1.868854 3.514286 -5.459539

H -2.004189 2.616007 -6.960475

H -0.282176 1.276032 -6.100963

H -1.699692 -0.571266 -5.773225

C -2.021729 -2.859077 -4.019950

O -3.156573 -3.046054 -3.577774

C -1.275603 -3.488241 -5.043223

C -1.838356 -4.657225 -5.748148

C -1.032827 -5.146073 -6.862342

C 0.172581 -4.601957 -7.190461

C 0.720311 -3.506129 -6.457890

C 0.006620 -2.980451 -5.417842

O -2.901674 -5.207559 -5.424271

H -1.444231 -5.989408 -7.407223

H 0.740239 -5.013630 -8.021726

H 1.692529 -3.104716 -6.722804

H 0.438981 -2.157133 -4.852753

O -1.644860 -5.863576 -2.461067

C -2.526385 -6.540787 -2.939628

C -2.275408 -7.711912 -3.819348

C -3.983882 -6.271650 -2.454253

F -4.034979 -5.207855 -1.647582

F -4.878632 -6.102338 -3.432877

F -4.389210 -7.348434 -1.720835

C -3.292822 -8.486623 -4.391733

C -2.971125 -9.585513 -5.180954

C -1.638014 -9.920364 -5.410590

C -0.619914 -9.148388 -4.850279

C -0.937066 -8.051843 -4.060682

H -4.332093 -8.229762 -4.240734

H -3.764648 -10.178366 -5.623870

H -1.392491 -10.778618 -6.028388

H 0.419514 -9.401633 -5.032121

H -0.160628 -7.437634 -3.620951

TS1_3-B_

C -1.476821 -1.924866 -3.907423

N -0.961056 -2.137801 -2.685038

N -0.738087 -0.957411 -2.006154

C -1.134196 -0.037331 -2.829716

N -1.600894 -0.580508 -3.998345

C -2.169534 0.289077 -5.051394

C -1.018850 1.447500 -2.753633

C -0.728255 -3.339236 -1.941501

C -1.750623 -3.866206 -1.145613

C -1.515258 -4.965012 -0.331725

C -0.242606 -5.523539 -0.311354

C 0.796642 -5.006870 -1.074194

C 0.545519 -3.902833 -1.880028

Cl -3.325561 -3.138679 -1.138138

H -2.315280 -5.380119 0.265009

Cl 0.061213 -6.910898 0.707934

H 1.784611 -5.444448 -1.041309

Cl 1.867238 -3.211621 -2.781136

O -0.503193 1.874691 -4.009100

H -1.984866 1.911333 -2.522612

H -0.296962 1.732683 -1.990707

C -1.338605 1.628147 -5.145632

C -3.584572 0.744557 -4.737226

C -2.396567 2.737322 -5.349692

C -3.699092 2.126647 -4.895642

C -4.921322 2.756261 -4.673273

C -6.018260 1.985510 -4.292389

C -5.900104 0.600759 -4.149778

C -4.681736 -0.036282 -4.377116

H -5.020639 3.830707 -4.794174

H -6.975135 2.464659 -4.112175

H -6.765987 0.011140 -3.868699

H -4.595036 -1.113556 -4.304840

H -2.107190 3.636083 -4.798863

H -2.446136 3.018665 -6.407633

H -0.633327 1.576686 -5.974228

H -2.124567 -0.263807 -5.987990

C -1.953394 -2.808869 -5.083694

O -3.032538 -2.459430 -5.519422

C -0.981070 -3.658882 -5.783390

C -1.433174 -4.863878 -6.382878

C -0.544612 -5.553783 -7.226076

C 0.731039 -5.066668 -7.471553

C 1.182281 -3.897279 -6.851701

C 0.326153 -3.206343 -6.005677

O -2.638051 -5.352172 -6.133910

H -0.888636 -6.479506 -7.672940

H 1.392572 -5.612808 -8.137332

H 2.184630 -3.526589 -7.037196

H 0.667343 -2.284820 -5.541171

O -2.308865 -4.761289 -3.777661

C -2.906479 -5.621227 -4.515843

C -2.468096 -7.084886 -4.366065

C -4.460199 -5.480043 -4.490982

F -4.861981 -4.200365 -4.563050

F -5.110180 -6.155028 -5.463100

F -4.916657 -5.964022 -3.306712

C -2.872896 -8.093587 -5.248345

C -2.441495 -9.404230 -5.063392

C -1.598921 -9.726695 -3.999751

C -1.186673 -8.725810 -3.124180

C -1.619943 -7.414726 -3.310641

H -3.513581 -7.848247 -6.084634

H -2.761913 -10.177072 -5.755778

H -1.265192 -10.750273 -3.858090

H -0.528494 -8.964060 -2.293762

H -1.303780 -6.623081 -2.645551

Int1_3-B_

C -1.357823 -2.141137 -4.151901

N -0.589388 -2.423645 -3.084182

N -0.247287 -1.297607 -2.362090

C -0.833930 -0.336662 -3.005467

N -1.520343 -0.800615 -4.102203

C -2.323920 0.123776 -4.944044

C -0.751917 1.136682 -2.798549

C -0.165648 -3.690956 -2.584166

C -0.927822 -4.346588 -1.610343

C -0.529304 -5.573734 -1.099082

C 0.661643 -6.128753 -1.552276

C 1.467148 -5.478539 -2.477842

C 1.051967 -4.250277 -2.980339

Cl -2.378037 -3.610008 -0.996636

H -1.142148 -6.086727 -0.371567

Cl 1.172506 -7.677909 -0.922433

H 2.404431 -5.909587 -2.801031

Cl 2.095348 -3.401989 -4.083497

O -0.553288 1.717713 -4.079471

H -1.655371 1.521295 -2.308588

H 0.112623 1.384636 -2.185261

C -1.649205 1.552945 -4.984464

C -3.706169 0.368035 -4.374849

C -2.795585 2.561803 -4.712132

C -3.957034 1.731586 -4.226362

C -5.185280 2.164602 -3.729742

C -6.149667 1.216688 -3.393387

C -5.900441 -0.147277 -3.571801

C -4.676885 -0.585447 -4.073109

H -5.392736 3.223955 -3.610383

H -7.109093 1.541474 -3.003146

H -6.671709 -0.871457 -3.331125

H -4.483250 -1.633475 -4.270952

H -2.463293 3.321557 -3.998530

H -3.055222 3.095118 -5.632761

H -1.194525 1.714459 -5.960676

H -2.385964 -0.340714 -5.925342

C -1.973057 -3.031626 -5.330936

O -2.883716 -2.410680 -5.937043

C -0.820376 -3.614800 -6.158478

C -0.563987 -4.986965 -6.213986

C 0.470472 -5.488211 -7.007276

C 1.246480 -4.619849 -7.762532

C 0.985157 -3.248623 -7.744810

C -0.047739 -2.764134 -6.952515

O -1.293209 -5.913795 -5.513541

H 0.633825 -6.559934 -7.031474

H 2.045478 -5.016360 -8.380770

H 1.574881 -2.568919 -8.350649

H -0.273704 -1.701951 -6.951341

O -2.470180 -4.221458 -4.413330

C -2.542694 -5.484096 -4.959943

C -2.920710 -6.471796 -3.855574

C -3.608409 -5.598425 -6.113048

F -3.235067 -4.993641 -7.249272

F -3.818057 -6.899098 -6.419109

F -4.786279 -5.078700 -5.727958

C -2.294334 -7.714343 -3.748164

C -2.659549 -8.597426 -2.734518

C -3.660766 -8.252415 -1.829138

C -4.295286 -7.016723 -1.942382

C -3.927580 -6.129464 -2.950685

H -1.519925 -7.984211 -4.453740

H -2.161992 -9.558789 -2.656142

H -3.948561 -8.944359 -1.043731

H -5.079229 -6.740689 -1.244210

H -4.413177 -5.166011 -3.033980

RC_3-B-minor_

C -1.395746 -1.641033 -3.312953

N -0.997904 -1.571175 -2.034973

N -0.755378 -0.271440 -1.629902

C -1.035049 0.433250 -2.683070

N -1.428652 -0.363571 -3.733911

C -1.870183 0.199642 -5.019621

C -0.878228 1.889906 -2.963326

C -0.866045 -2.659835 -1.121971

C -1.933496 -3.011225 -0.287934

C -1.829025 -4.092940 0.575582

C -0.640491 -4.809816 0.609456

C 0.447020 -4.466967 -0.183780

C 0.325599 -3.387066 -1.045951

Cl -3.404384 -2.088534 -0.302989

H -2.666867 -4.374004 1.197769

Cl -0.505713 -6.180016 1.685291

H 1.367762 -5.031504 -0.141236

Cl 1.695410 -2.937258 -2.025468

O -0.274985 1.998214 -4.248355

H -1.844112 2.408056 -2.917472

H -0.198829 2.347512 -2.246655

C -1.039653 1.500616 -5.351684

C -3.314642 0.662605 -4.991722

C -2.080236 2.523728 -5.871583

C -3.424336 1.968738 -5.469014

C -4.674945 2.577256 -5.549438

C -5.801155 1.861883 -5.146605

C -5.683260 0.551783 -4.675054

C -4.436208 -0.064898 -4.595261

H -4.773879 3.593356 -5.919198

H -6.780504 2.326073 -5.202645

H -6.569519 0.004948 -4.371896

H -4.347469 -1.084474 -4.233132

H -1.868854 3.514286 -5.459539

H -2.004189 2.616007 -6.960475

H -0.282176 1.276032 -6.100963

H -1.699692 -0.571266 -5.773225

C -2.021729 -2.859077 -4.019950

O -3.156573 -3.046054 -3.577774

C -1.275603 -3.488241 -5.043223

C -1.838356 -4.657225 -5.748148

C -1.032827 -5.146073 -6.862342

C 0.172581 -4.601957 -7.190461

C 0.720311 -3.506129 -6.457890

C 0.006620 -2.980451 -5.417842

O -2.901674 -5.207559 -5.424271

H -1.444231 -5.989408 -7.407223

H 0.740239 -5.013630 -8.021726

H 1.692529 -3.104716 -6.722804

H 0.438981 -2.157133 -4.852753

O -1.644860 -5.863576 -2.461067

C -2.526385 -6.540787 -2.939628

C -2.275408 -7.711912 -3.819348

C -3.983882 -6.271650 -2.454253

F -4.034979 -5.207855 -1.647582

F -4.878632 -6.102338 -3.432877

F -4.389210 -7.348434 -1.720835

C -3.292822 -8.486623 -4.391733

C -2.971125 -9.585513 -5.180954

C -1.638014 -9.920364 -5.410590

C -0.619914 -9.148388 -4.850279

C -0.937066 -8.051843 -4.060682

H -4.332093 -8.229762 -4.240734

H -3.764648 -10.178366 -5.623870

H -1.392491 -10.778618 -6.028388

H 0.419514 -9.401633 -5.032121

H -0.160628 -7.437634 -3.620951

TS1_3-B-minor_

C -1.382510 -1.968761 -3.946618

N -0.742327 -2.118417 -2.771262

N -0.536743 -0.912064 -2.138860

C -1.067414 -0.039547 -2.939585

N -1.603083 -0.640310 -4.050661

C -2.302956 0.160589 -5.078607

C -1.035771 1.450786 -2.907904

C -0.382207 -3.311643 -2.064338

C -1.295235 -3.891004 -1.177165

C -0.996974 -5.075614 -0.522822

C 0.244153 -5.661321 -0.737835

C 1.204045 -5.064415 -1.545389

C 0.888592 -3.875744 -2.190503

Cl -2.809579 -3.103322 -0.837104

H -1.726353 -5.537942 0.126788

Cl 0.622918 -7.168173 0.058696

H 2.182872 -5.506471 -1.667352

Cl 2.140190 -3.064653 -3.092315

O -0.655410 1.869677 -4.213661

H -2.004119 1.866168 -2.605055

H -0.269455 1.798824 -2.217916

C -1.571685 1.548646 -5.265410

C -3.718920 0.530931 -4.673581

C -2.711211 2.586953 -5.391637

C -3.935242 1.899656 -4.839963

C -5.178881 2.451153 -4.541072

C -6.194172 1.615985 -4.078387

C -5.975563 0.243982 -3.930502

C -4.735326 -0.314613 -4.232453

H -5.357502 3.514731 -4.666664

H -7.166620 2.034146 -3.839242

H -6.780223 -0.397925 -3.588690

H -4.572468 -1.382769 -4.156830

H -2.439009 3.505981 -4.865926

H -2.857677 2.858303 -6.443043

H -0.940748 1.523257 -6.152973

H -2.287603 -0.417842 -6.000913

C -1.890308 -2.946722 -5.042121

O -3.016202 -2.695834 -5.425647

C -0.912397 -3.838055 -5.657726

C -1.290848 -5.133914 -6.136673

C -0.322686 -5.847537 -6.890620

C 0.915412 -5.310686 -7.177581

C 1.282127 -4.045129 -6.692487

C 0.375132 -3.335438 -5.929323

O -2.445833 -5.678880 -5.900151

H -0.603896 -6.835605 -7.237415

H 1.620828 -5.882152 -7.774185

H 2.257174 -3.627290 -6.917542

H 0.647308 -2.344565 -5.575886

O -2.418240 -4.770766 -3.502099

C -3.036493 -5.636013 -4.162345

C -4.525182 -5.490301 -4.414384

C -2.577394 -7.087968 -3.840308

F -2.982678 -7.390616 -2.575691

F -3.079282 -8.043941 -4.642593

F -1.237444 -7.202279 -3.854089

C -5.194669 -6.105174 -5.477130

C -6.569278 -5.943899 -5.625060

C -7.293611 -5.180390 -4.710331

C -6.632521 -4.571023 -3.646409

C -5.255082 -4.722901 -3.503840

H -4.631289 -6.684910 -6.194703

H -7.077967 -6.416308 -6.459921

H -8.367038 -5.063647 -4.826207

H -7.190185 -3.982784 -2.923139

H -4.728116 -4.256093 -2.681049

Int1_3-B-minor_

C -1.535613 -2.151635 -3.792754

N -0.850223 -2.286000 -2.644442

N -0.482952 -1.067317 -2.104752

C -0.971951 -0.203929 -2.939499

N -1.627368 -0.817523 -3.979170

C -2.321319 -0.013492 -5.014704

C -0.791433 1.274157 -3.008868

C -0.496345 -3.458055 -1.907017

C -1.333787 -3.924306 -0.888875

C -0.975767 -5.019592 -0.116531

C 0.243303 -5.639584 -0.362397

C 1.107897 -5.186091 -1.349087

C 0.732799 -4.087448 -2.113022

Cl -2.845249 -3.125739 -0.562993

H -1.641872 -5.385453 0.652040

Cl 0.700028 -7.027240 0.597394

H 2.056270 -5.673932 -1.525956

Cl 1.832832 -3.497104 -3.326362

O -0.463522 1.573621 -4.360232

H -1.690283 1.806229 -2.674531

H 0.050675 1.581680 -2.391539

C -1.496256 1.300057 -5.314240

C -3.676796 0.487561 -4.555711

C -2.547274 2.435630 -5.379966

C -3.790148 1.864969 -4.743798

C -4.970728 2.521157 -4.401386

C -6.027508 1.779168 -3.876991

C -5.915687 0.395058 -3.717674

C -4.739827 -0.267177 -4.064074

H -5.070544 3.593125 -4.543656

H -6.951175 2.279205 -3.603221

H -6.757179 -0.172191 -3.333893

H -4.651026 -1.345726 -4.004687

H -2.161563 3.331248 -4.884944

H -2.737381 2.712661 -6.422749

H -0.952717 1.194636 -6.252192

H -2.420357 -0.653424 -5.888815

C -2.185625 -3.171585 -4.812852

O -3.223143 -2.692957 -5.342232

C -1.122554 -3.774351 -5.728480

C -1.198764 -5.140626 -6.016000

C -0.306408 -5.728408 -6.914072

C 0.657341 -4.948996 -7.542730

C 0.742939 -3.583475 -7.273178

C -0.147406 -3.010976 -6.369571

O -2.155591 -5.955242 -5.485528

H -0.388523 -6.792493 -7.105586

H 1.344336 -5.411481 -8.244132

H 1.493437 -2.971952 -7.762722

H -0.077806 -1.946735 -6.159620

O -2.421690 -4.359472 -3.769001

C -2.904990 -5.514579 -4.331128

C -4.394703 -5.494078 -4.678019

C -2.616506 -6.633491 -3.283584

F -3.247816 -6.390219 -2.115999

F -3.018321 -7.837588 -3.720054

F -1.298232 -6.718645 -3.015191

C -4.896705 -6.213524 -5.762126

C -6.264732 -6.218414 -6.026659

C -7.141304 -5.519508 -5.201288

C -6.642450 -4.810290 -4.109662

C -5.276020 -4.796872 -3.849699

H -4.215696 -6.757701 -6.403808

H -6.644433 -6.772209 -6.879608

H -8.207262 -5.526142 -5.406983

H -7.319658 -4.262341 -3.461579

H -4.882535 -4.236466 -3.011014

RC_4-A_

C -2.003507 -1.143684 -2.453234

N -0.692960 -0.885077 -2.596676

N -0.462227 0.192262 -3.426951

C -1.655382 0.581701 -3.764807

N -2.630532 -0.200049 -3.190177

C -4.090417 -0.110207 -3.541231

C -2.004913 1.732271 -4.654885

C 0.418308 -1.621763 -2.087887

C 1.188468 -1.112739 -1.035262

C 2.279348 -1.815480 -0.541655

C 2.604551 -3.036871 -1.120333

C 1.881040 -3.554263 -2.187181

C 0.796660 -2.835817 -2.677898

Cl 0.807789 0.433532 -0.325503

H 2.859084 -1.416296 0.278908

Cl 3.968364 -3.934556 -0.500707

H 2.156254 -4.496189 -2.641037

Cl -0.062988 -3.453248 -4.053669

O -3.273424 1.540884 -5.243848

H -1.957092 2.669374 -4.084098

H -1.279705 1.798859 -5.465422

C -4.312138 1.210123 -4.332935

C -5.001290 0.080904 -2.351308

C -4.670672 2.274335 -3.266542

C -5.324712 1.435190 -2.195508

C -6.188621 1.835314 -1.182050

C -6.725132 0.867204 -0.332438

C -6.421678 -0.483767 -0.508314

C -5.562619 -0.892595 -1.527840

H -6.453706 2.881015 -1.058435

H -7.400228 1.166341 0.463079

H -6.862765 -1.227328 0.146389

H -5.353094 -1.944400 -1.680701

H -3.778379 2.782374 -2.881681

H -5.322626 3.049062 -3.676191

H -5.181538 1.039876 -4.968456

H -4.311156 -1.001125 -4.126440

C -2.706000 -2.417583 -1.950653

O -3.211534 -2.988381 -2.922751

C -2.713946 -2.728980 -0.573514

C -3.439634 -3.928416 -0.099263

C -3.307786 -4.197550 1.329022

C -2.641545 -3.363017 2.173913

C -2.019925 -2.166625 1.702627

C -2.065094 -1.875620 0.369865

O -4.133142 -4.653286 -0.830104

H -3.800690 -5.095985 1.686532

H -2.589641 -3.599976 3.233976

H -1.525786 -1.498196 2.399257

H -1.616593 -0.949295 0.023798

C -4.735459 -3.779124 -5.452471

C -3.836640 -3.248245 -6.612159

C -4.674000 -5.223086 -5.140933

O -5.407020 -2.968418 -4.860901

F -3.710647 -1.914675 -6.551599

F -2.597583 -3.777088 -6.582049

F -4.376988 -3.550532 -7.816679

C -4.825303 -5.607945 -3.800531

C -4.777408 -6.955404 -3.462908

C -4.613310 -7.922259 -4.453721

C -4.469029 -7.542565 -5.789056

C -4.481032 -6.196682 -6.134232

H -4.896666 -4.860542 -3.021427

H -4.853220 -7.235532 -2.418244

H -4.584916 -8.974269 -4.186543

H -4.340042 -8.295357 -6.559871

H -4.363188 -5.910132 -7.172659

TS1_4-A_

C -2.861934 -1.503484 -2.252017

N -1.666965 -1.401718 -2.862766

N -1.487243 -0.181920 -3.462747

C -2.582925 0.462584 -3.187546

N -3.437543 -0.294929 -2.426610

C -4.760951 0.253695 -2.097753

C -3.091504 1.763374 -3.712575

C -0.624522 -2.373793 -2.974479

C 0.291016 -2.555768 -1.936397

C 1.327095 -3.472319 -2.048481

C 1.444406 -4.200382 -3.227011

C 0.558689 -4.028941 -4.283694

C -0.473916 -3.108052 -4.155386

Cl 0.144458 -1.631047 -0.461552

H 2.024903 -3.611948 -1.234640

Cl 2.743249 -5.359042 -3.383641

H 0.660285 -4.604763 -5.193094

Cl -1.590338 -2.899676 -5.463280

O -4.397762 1.534907 -4.228157

H -3.082213 2.536370 -2.936099

H -2.472832 2.091633 -4.546191

C -5.378316 0.944424 -3.354437

C -4.735547 1.373016 -1.064947

C -6.306115 2.016748 -2.752013

C -5.650518 2.363400 -1.435578

C -5.838355 3.480637 -0.628660

C -5.089393 3.607479 0.541473

C -4.160913 2.628626 0.897233

C -3.980145 1.500890 0.095845

H -6.547012 4.252892 -0.911727

H -5.221476 4.479084 1.174103

H -3.574422 2.743764 1.802585

H -3.249385 0.751768 0.377474

H -6.408840 2.863190 -3.434097

H -7.307074 1.596694 -2.596336

H -5.896804 0.211413 -3.965923

H -5.389919 -0.580974 -1.795126

C -3.384388 -2.820632 -1.654992

O -2.730571 -3.799392 -1.930628

C -4.567027 -2.781201 -0.781981

C -5.692041 -3.633405 -1.037370

C -6.721081 -3.623560 -0.053208

C -6.630619 -2.866545 1.097898

C -5.521797 -2.038649 1.330156

C -4.511831 -1.999577 0.384881

O -5.814859 -4.359129 -2.102943

H -7.580475 -4.259084 -0.235864

H -7.431939 -2.908857 1.830277

H -5.446356 -1.454219 2.240398

H -3.625427 -1.402522 0.580190

C -5.800935 -3.379615 -3.759586

C -5.188412 -4.575868 -4.536217

C -7.291644 -3.206272 -3.986109

O -5.079612 -2.371209 -3.630614

F -5.892468 -5.716725 -4.444836

F -5.170735 -4.232633 -5.857293

F -3.925793 -4.838187 -4.188297

C -7.698824 -2.073488 -4.698729

C -9.045353 -1.850681 -4.975620

C -10.004463 -2.760880 -4.537796

C -9.605652 -3.897646 -3.836033

C -8.258636 -4.126554 -3.567870

H -6.943992 -1.385997 -5.062081

H -9.343776 -0.972549 -5.540373

H -11.055721 -2.590444 -4.748817

H -10.348098 -4.613227 -3.496359

H -7.945092 -5.002182 -3.017935

Int1_4-A_

C -2.984955 -1.530997 -2.338198

N -1.818212 -1.405573 -2.995256

N -1.704558 -0.190748 -3.631207

C -2.803750 0.425430 -3.314305

N -3.599746 -0.341502 -2.500741

C -4.916161 0.170560 -2.099189

C -3.378938 1.700377 -3.833554

C -0.737214 -2.333559 -3.111443

C 0.279366 -2.352883 -2.154712

C 1.363736 -3.210287 -2.276112

C 1.424379 -4.048569 -3.383422

C 0.435784 -4.043679 -4.359229

C -0.643136 -3.178167 -4.221934

Cl 0.198019 -1.294319 -0.766723

H 2.140139 -3.222089 -1.523945

Cl 2.783071 -5.136368 -3.551539

H 0.494217 -4.704520 -5.212973

Cl -1.890579 -3.180992 -5.422606

O -4.703021 1.416768 -4.272512

H -3.355246 2.488800 -3.072665

H -2.817754 2.032637 -4.705239

C -5.620646 0.861369 -3.313911

C -4.854150 1.273090 -1.052210

C -6.488701 1.956341 -2.660398

C -5.774339 2.276100 -1.368491

C -5.922465 3.378290 -0.532027

C -5.130890 3.473609 0.612590

C -4.201334 2.477937 0.916576

C -4.059225 1.367069 0.085081

H -6.634497 4.162001 -0.772115

H -5.232116 4.332960 1.267421

H -3.584664 2.567853 1.804640

H -3.333128 0.599312 0.325282

H -6.596134 2.810536 -3.332406

H -7.495027 1.566173 -2.467344

H -6.202216 0.134680 -3.875686

H -5.505980 -0.684377 -1.777339

C -3.412689 -2.820632 -1.649187

O -2.650680 -3.757924 -1.751361

C -4.539973 -2.787055 -0.664679

C -5.752861 -3.477671 -0.873780

C -6.694840 -3.500475 0.165907

C -6.437147 -2.894712 1.388274

C -5.232844 -2.226268 1.601589

C -4.297979 -2.182464 0.573912

O -6.062749 -4.170489 -1.985609

H -7.626702 -4.025004 -0.011172

H -7.181212 -2.943680 2.177050

H -5.017737 -1.760725 2.557114

H -3.336673 -1.706184 0.746095

C -5.777329 -3.563483 -3.350319

C -5.240426 -4.814467 -4.119227

C -7.142318 -3.159751 -3.943707

O -4.918846 -2.571375 -3.313258

F -6.111608 -5.846660 -4.115480

F -5.018224 -4.490273 -5.416801

F -4.080726 -5.266760 -3.620272

C -7.163287 -2.205750 -4.965034

C -8.368828 -1.804834 -5.536339

C -9.570171 -2.354808 -5.091304

C -9.554134 -3.315530 -4.082735

C -8.346586 -3.722555 -3.517128

H -6.223227 -1.791040 -5.308614

H -8.369489 -1.068194 -6.334268

H -10.511218 -2.043261 -5.534184

H -10.484511 -3.755980 -3.737102

H -8.333315 -4.478351 -2.742532

RC_4-A-minor_

C -2.176281 -0.568860 -2.570011

N -0.892003 -0.169529 -2.563443

N -0.752741 1.141523 -2.960775

C -1.972613 1.526454 -3.193011

N -2.877485 0.517463 -2.964992

C -4.340546 0.620895 -3.290713

C -2.410247 2.884944 -3.642352

C 0.282303 -0.932544 -2.284759

C 1.010076 -0.708956 -1.110002

C 2.168401 -1.424299 -0.839464

C 2.601431 -2.368386 -1.763008

C 1.916790 -2.595466 -2.949820

C 0.764055 -1.864465 -3.213648

Cl 0.487488 0.488662 0.044740

H 2.716103 -1.249236 0.075928

Cl 4.052281 -3.281492 -1.427432

H 2.272336 -3.322017 -3.667247

Cl -0.053688 -2.110268 -4.724301

O -3.666947 2.822430 -4.282137

H -2.422286 3.571418 -2.785156

H -1.697989 3.273787 -4.369660

C -4.668665 2.116862 -3.565138

C -5.250073 0.309097 -2.123763

C -5.096578 2.703893 -2.198217

C -5.673679 1.493272 -1.505460

C -6.550867 1.444433 -0.427429

C -6.999201 0.202209 0.022146

C -6.593698 -0.973531 -0.611180

C -5.720880 -0.931377 -1.697689

H -6.892156 2.356820 0.052218

H -7.683324 0.150725 0.863124

H -6.966011 -1.930997 -0.263616

H -5.428029 -1.841322 -2.205929

H -4.242802 3.108151 -1.641487

H -5.809903 3.522141 -2.318887

H -5.529506 2.120490 -4.234491

H -4.491906 -0.013032 -4.160865

C -2.747442 -1.995572 -2.535299

O -3.372609 -2.217443 -3.580119

C -2.491397 -2.816393 -1.414234

C -2.800780 -4.264343 -1.472963

C -2.493578 -5.005307 -0.252184

C -1.994320 -4.408593 0.864274

C -1.736653 -3.003807 0.905250

C -1.978527 -2.250438 -0.206658

O -3.268621 -4.836710 -2.467555

H -2.703221 -6.069573 -0.284795

H -1.794097 -5.005974 1.750531

H -1.371295 -2.543334 1.816716

H -1.828993 -1.176079 -0.148938

C -4.003551 -2.417714 -6.320948

C -5.453043 -2.668693 -5.800677

C -3.095082 -3.569450 -6.545725

O -3.747298 -1.286462 -6.662769

F -5.965734 -1.553853 -5.246786

F -6.214773 -2.965172 -6.882023

F -5.601888 -3.665078 -4.923217

C -2.281740 -3.518047 -7.689802

C -1.388922 -4.546237 -7.959518

C -1.276361 -5.618000 -7.071022

C -2.055090 -5.652998 -5.916965

C -2.973980 -4.640847 -5.649519

H -2.371884 -2.671578 -8.361495

H -0.779620 -4.513553 -8.857047

H -0.573745 -6.419789 -7.277156

H -1.947332 -6.463609 -5.204431

H -3.520484 -4.656650 -4.716695

TS1_4-A-minor_

C -2.771145 -1.582001 -2.404091

N -1.525693 -1.388141 -2.872131

N -1.350367 -0.129884 -3.391986

C -2.507940 0.438802 -3.214755

N -3.393141 -0.399460 -2.582423

C -4.779276 0.055488 -2.377061

C -3.049959 1.716995 -3.764659

C -0.423670 -2.296784 -2.857253

C 0.348512 -2.451515 -1.703266

C 1.435663 -3.315034 -1.677904

C 1.751595 -4.019059 -2.833696

C 1.013072 -3.874212 -4.002225

C -0.072576 -3.007961 -4.009621

Cl -0.035159 -1.549282 -0.257818

H 2.021214 -3.431125 -0.776570

Cl 3.115945 -5.109456 -2.817815

H 1.269209 -4.429940 -4.893593

Cl -0.996096 -2.828830 -5.465446

O -4.263457 1.398940 -4.435826

H -3.191869 2.463867 -2.975907

H -2.366757 2.116450 -4.512467

C -5.301648 0.741017 -3.680246

C -4.936937 1.139574 -1.320947

C -6.340703 1.752047 -3.161467

C -5.856485 2.092410 -1.771234

C -6.205138 3.166952 -0.959623

C -5.614855 3.287927 0.298609

C -4.689256 2.341942 0.740274

C -4.347261 1.256437 -0.066748

H -6.918665 3.909484 -1.303610

H -5.873355 4.126518 0.936987

H -4.233876 2.448020 1.719299

H -3.633087 0.522840 0.287128

H -6.411934 2.610848 -3.832211

H -7.328897 1.278268 -3.127458

H -5.713276 -0.005839 -4.354057

H -5.380625 -0.826478 -2.172474

C -3.313732 -2.964876 -1.991310

O -2.745036 -3.903911 -2.500478

C -4.383462 -2.997525 -0.991145

C -5.553135 -3.803106 -1.178285

C -6.454685 -3.873834 -0.079771

C -6.209744 -3.221307 1.110680

C -5.066681 -2.421533 1.273086

C -4.179676 -2.307282 0.218761

O -5.831742 -4.420953 -2.283954

H -7.348091 -4.472262 -0.220182

H -6.917983 -3.317310 1.928768

H -4.874664 -1.914835 2.212410

H -3.265145 -1.734109 0.351497

C -5.821351 -3.407468 -3.893522

C -7.325470 -3.024195 -3.783742

C -5.507781 -4.532176 -4.849111

O -5.055053 -2.427796 -3.791131

F -7.543723 -2.172806 -2.753895

F -7.672253 -2.348604 -4.913812

F -8.191236 -4.039293 -3.659765

C -4.646076 -4.243672 -5.910276

C -4.339243 -5.216641 -6.857508

C -4.886962 -6.492433 -6.747012

C -5.740611 -6.788443 -5.684780

C -6.055732 -5.815105 -4.741865

H -4.224466 -3.248062 -5.979678

H -3.673554 -4.978877 -7.681770

H -4.648308 -7.254330 -7.482936

H -6.160528 -7.784860 -5.587744

H -6.698486 -6.051215 -3.905744

Int1_4-A-minor_

C -2.855500 -1.565896 -2.521723

N -1.593566 -1.378648 -2.946879

N -1.378951 -0.100172 -3.407002

C -2.526727 0.484815 -3.228451

N -3.445598 -0.362146 -2.659471

C -4.827368 0.107054 -2.468378

C -3.027173 1.801435 -3.722596

C -0.502422 -2.301185 -2.952717

C 0.272784 -2.487207 -1.806064

C 1.354684 -3.357084 -1.803026

C 1.664264 -4.035297 -2.975742

C 0.926521 -3.855458 -4.139572

C -0.153181 -2.982064 -4.122905

Cl -0.101299 -1.614867 -0.339254

H 1.940887 -3.498274 -0.905715

Cl 3.021972 -5.135403 -2.988342

H 1.179591 -4.388069 -5.045777

Cl -1.072904 -2.752974 -5.575982

O -4.229136 1.545677 -4.440776

H -3.177329 2.507371 -2.898358

H -2.317113 2.226775 -4.429859

C -5.299221 0.874918 -3.744682

C -5.001189 1.126537 -1.355173

C -6.328300 1.883216 -3.198421

C -5.882635 2.127671 -1.774927

C -6.236659 3.156025 -0.907556

C -5.691497 3.179019 0.376604

C -4.808037 2.180940 0.789700

C -4.460036 1.142219 -0.074352

H -6.920009 3.937111 -1.226644

H -5.954313 3.980717 1.059132

H -4.391093 2.210021 1.790951

H -3.782802 0.363165 0.255435

H -6.354494 2.781193 -3.819316

H -7.329195 1.436432 -3.223236

H -5.714500 0.180431 -4.471830

H -5.443615 -0.778547 -2.340493

C -3.393843 -2.936504 -2.088519

O -2.711263 -3.892690 -2.399028

C -4.430614 -2.965454 -1.010557

C -5.668203 -3.611887 -1.193466

C -6.543456 -3.719697 -0.104541

C -6.191368 -3.229330 1.145582

C -4.963891 -2.594579 1.333387

C -4.097496 -2.467059 0.253403

O -6.055050 -4.184552 -2.349715

H -7.498867 -4.203836 -0.271578

H -6.881646 -3.337056 1.976257

H -4.679739 -2.217510 2.309856

H -3.121713 -2.011564 0.400105

C -5.762401 -3.491926 -3.662119

C -7.160287 -2.879034 -4.012410

C -5.421655 -4.617532 -4.644131

O -4.888835 -2.510146 -3.569572

F -7.519594 -1.939968 -3.095654

F -7.126118 -2.252868 -5.209622

F -8.157458 -3.779394 -4.059186

C -4.591064 -4.318918 -5.723736

C -4.275903 -5.296751 -6.664118

C -4.788862 -6.584932 -6.530723

C -5.619142 -6.888223 -5.452982

C -5.940608 -5.908613 -4.517051

H -4.189699 -3.316567 -5.805914

H -3.626875 -5.053628 -7.500145

H -4.541876 -7.349624 -7.261000

H -6.018718 -7.891491 -5.340225

H -6.581092 -6.147117 -3.677708

RC_4-B_

C -1.924254 -1.297433 -2.502769

N -0.703407 -0.747917 -2.388943

N -0.636357 0.496824 -2.978446

C -1.837355 0.688332 -3.435672

N -2.661332 -0.379951 -3.166800

C -4.045694 -0.528773 -3.732713

C -2.340934 1.900004 -4.154721

C 0.485497 -1.320788 -1.843597

C 1.007385 -0.859061 -0.629360

C 2.170862 -1.400580 -0.100322

C 2.819914 -2.407534 -0.805881

C 2.347661 -2.862083 -2.030177

C 1.186554 -2.304703 -2.554976

Cl 0.220721 0.428868 0.244565

H 2.558477 -1.043762 0.843693

Cl 4.277735 -3.104275 -0.142372

H 2.873615 -3.631024 -2.578929

Cl 0.637622 -2.818932 -4.117394

O -3.462164 1.581542 -4.952029

H -2.570710 2.690836 -3.428096

H -1.566627 2.275696 -4.822947

C -4.485077 0.856558 -4.286192

C -5.113595 -0.804386 -2.699055

C -5.193825 1.560187 -3.103616

C -5.762008 0.386077 -2.342959

C -6.812993 0.364019 -1.432962

C -7.207958 -0.858844 -0.889260

C -6.575825 -2.044830 -1.265677

C -5.525785 -2.031665 -2.183264

H -7.327993 1.279105 -1.156659

H -8.026555 -0.889684 -0.177288

H -6.904708 -2.989822 -0.847630

H -5.050249 -2.956392 -2.488587

H -4.488174 2.127193 -2.484701

H -5.950184 2.267553 -3.450493

H -5.220657 0.654737 -5.065609

H -3.960870 -1.292206 -4.502642

C -2.390603 -2.747577 -2.292262

O -2.707943 -3.231905 -3.386642

C -2.441796 -3.280172 -0.985035

C -2.917762 -4.665776 -0.770841

C -2.877505 -5.122480 0.614988

C -2.501474 -4.314531 1.643488

C -2.115658 -2.957697 1.419148

C -2.092169 -2.475152 0.143281

O -3.341261 -5.402936 -1.674535

H -3.191979 -6.147866 0.780774

H -2.504897 -4.699608 2.660394

H -1.854398 -2.319769 2.256429

H -1.831572 -1.433143 -0.010363

C -2.125958 -4.375102 -6.070656

C -3.657659 -4.126817 -6.229531

C -1.649660 -5.681787 -5.556287

O -1.395749 -3.527154 -6.528985

F -4.031940 -4.676253 -7.409622

F -4.438697 -4.653948 -5.279738

F -3.929401 -2.809615 -6.300972

C -0.539895 -6.250618 -6.203235

C -0.021589 -7.461087 -5.765056

C -0.581503 -8.094350 -4.652998

C -1.656979 -7.513301 -3.986338

C -2.206926 -6.316038 -4.438093

H -0.106150 -5.738886 -7.055209

H 0.820463 -7.909181 -6.282836

H -0.168887 -9.035497 -4.302226

H -2.072244 -7.975349 -3.097596

H -2.998380 -5.853902 -3.866048

TS1_4-B_

C -2.407879 -1.361123 -2.246244

N -1.148875 -1.232673 -1.779911

N -0.603512 -0.007990 -2.091563

C -1.551209 0.607992 -2.729430

N -2.666441 -0.179999 -2.853122

C -3.866931 0.323796 -3.555532

C -1.543579 1.918573 -3.439773

C -0.291070 -2.207243 -1.172743

C -0.165421 -2.298880 0.215706

C 0.612550 -3.291727 0.795179

C 1.301178 -4.167978 -0.035639

C 1.281477 -4.032298 -1.417406

C 0.503523 -3.030756 -1.979231

Cl -0.896254 -1.105214 1.259145

H 0.685160 -3.369222 1.870879

Cl 2.259267 -5.439543 0.681714

H 1.851518 -4.696083 -2.051612

Cl 0.607342 -2.754513 -3.691682

O -2.087232 1.683945 -4.732100

H -2.107324 2.677832 -2.883868

H -0.521441 2.266989 -3.575449

C -3.443829 1.229549 -4.776344

C -4.729585 1.227063 -2.694856

C -4.455689 2.400590 -4.767030

C -5.055707 2.396370 -3.382920

C -5.874521 3.352425 -2.786787

C -6.360391 3.122240 -1.500847

C -6.042851 1.943867 -0.821455

C -5.227216 0.983548 -1.416903

H -6.132951 4.265376 -3.314460

H -6.996408 3.861618 -1.025387

H -6.438383 1.770063 0.173419

H -5.003556 0.061819 -0.890404

H -3.951405 3.333432 -5.031813

H -5.229161 2.233963 -5.524942

H -3.491555 0.653663 -5.699197

H -4.405533 -0.554960 -3.903213

C -3.451787 -2.518352 -2.251020

O -4.102871 -2.575594 -3.275322

C -3.715807 -3.212080 -0.990919

C -4.080753 -4.596193 -0.974467

C -4.511173 -5.134879 0.265362

C -4.598390 -4.360295 1.404212

C -4.218368 -3.009645 1.384477

C -3.771297 -2.460427 0.196886

O -4.014782 -5.369481 -2.017953

H -4.771568 -6.187328 0.277448

H -4.948841 -4.807362 2.330130

H -4.279252 -2.403394 2.281577

H -3.499179 -1.409909 0.176242

C -2.475571 -5.287860 -2.979596

C -1.851786 -6.561583 -2.336716

C -3.039230 -5.496121 -4.372360

O -1.831522 -4.246429 -2.708312

F -0.663321 -6.812942 -2.950742

F -1.580387 -6.376904 -1.031638

F -2.585399 -7.684152 -2.446108

C -2.451950 -4.778397 -5.415873

C -2.892931 -4.942551 -6.726587

C -3.934415 -5.824083 -7.005398

C -4.529339 -6.539223 -5.966369

C -4.083570 -6.381885 -4.657511

H -1.645505 -4.093955 -5.183196

H -2.422645 -4.383104 -7.530103

H -4.282078 -5.954226 -8.025974

H -5.346929 -7.222219 -6.176377

H -4.555665 -6.924536 -3.850476

Int1_4-B_

C 0.134104 1.256649 -0.006375

N 1.161554 1.399978 0.852074

N 1.687003 2.676921 0.849849

C 0.949717 3.303587 -0.012621

N -0.007074 2.482449 -0.561020

C -0.910937 2.995206 -1.622056

C 1.116735 4.668185 -0.585261

C 1.874231 0.388864 1.561750

C 1.615973 0.122551 2.909540

C 2.306896 -0.878294 3.580709

C 3.286100 -1.587722 2.895171

C 3.624252 -1.288592 1.581252

C 2.930492 -0.278074 0.927487

Cl 0.472469 1.090197 3.794566

H 2.086975 -1.091596 4.617376

Cl 4.146541 -2.863818 3.725344

H 4.416950 -1.820301 1.074057

Cl 3.444237 0.210402 -0.657010

O 1.092447 4.509081 -1.997641

H 0.337636 5.355140 -0.232827

H 2.091827 5.070833 -0.317501

C -0.145095 4.031663 -2.537081

C -2.096038 3.771408 -1.083815

C -1.151700 5.184475 -2.773040

C -2.213732 5.009060 -1.716613

C -3.255885 5.870746 -1.380396

C -4.177356 5.473027 -0.413437

C -4.074454 4.217588 0.191683

C -3.036499 3.351797 -0.146184

H -3.354340 6.836472 -1.867251

H -4.991559 6.136933 -0.140822

H -4.816933 3.908254 0.919930

H -2.978760 2.351367 0.264250

H -0.633424 6.146424 -2.732620

H -1.587756 5.105221 -3.775053

H 0.151928 3.552348 -3.468833

H -1.242700 2.120049 -2.174932

C -0.866852 0.047022 -0.335580

O -1.868139 0.479267 -0.966980

C -1.154814 -0.813844 0.902720

C -0.695440 -2.129323 1.031687

C -1.029512 -2.898227 2.149387

C -1.851289 -2.367962 3.134417

C -2.360082 -1.075139 2.999868

C -2.013085 -0.318139 1.888554

O 0.080048 -2.733649 0.081215

H -0.649290 -3.911876 2.212212

H -2.114873 -2.973044 3.996117

H -3.020502 -0.663576 3.755645

H -2.411330 0.684245 1.773310

C -0.022482 -2.153559 -1.230352

C 1.204732 -2.764054 -1.969635

C -1.312036 -2.605580 -1.926158

O 0.179562 -0.787950 -1.196836

F 1.336086 -2.229322 -3.194563

F 2.353936 -2.548472 -1.303783

F 1.080086 -4.096763 -2.113111

C -1.851281 -1.837372 -2.958849

C -2.982802 -2.274333 -3.640690

C -3.587065 -3.481774 -3.297196

C -3.049536 -4.254200 -2.270372

C -1.912156 -3.822376 -1.593299

H -1.393952 -0.889434 -3.203799

H -3.399907 -1.663719 -4.435282

H -4.473655 -3.818299 -3.825791

H -3.513178 -5.196620 -1.996270

H -1.488699 -4.430426 -0.803628

RC_4-B-minor_

C -2.168172 -1.277525 -2.092755

N -0.888083 -1.021910 -2.411189

N -0.778476 -0.066996 -3.400562

C -2.010205 0.253257 -3.660752

N -2.895802 -0.455790 -2.883114

C -4.383333 -0.440978 -3.084190

C -2.485068 1.261380 -4.658810

C 0.298249 -1.648916 -1.922521

C 1.163686 -0.957590 -1.065931

C 2.328357 -1.548443 -0.596866

C 2.628848 -2.844159 -1.002359

C 1.808343 -3.543550 -1.877391

C 0.648189 -2.937571 -2.348984

Cl 0.808411 0.678772 -0.575796

H 2.983415 -1.008113 0.072198

Cl 4.085926 -3.602347 -0.408749

H 2.064838 -4.542681 -2.201586

Cl -0.331805 -3.788010 -3.499556

O -3.804742 0.970275 -5.072296

H -2.405868 2.270827 -4.234354

H -1.853786 1.221679 -5.545904

C -4.728641 0.755710 -4.016304

C -5.179677 -0.111763 -1.841818

C -5.001774 1.946171 -3.064987

C -5.528482 1.245547 -1.835722

C -6.303177 1.763345 -0.803742

C -6.724604 0.909474 0.216135

C -6.394264 -0.446366 0.193927

C -5.623344 -0.974745 -0.841216

H -6.586795 2.811366 -0.793920

H -7.328764 1.301356 1.028117

H -6.742323 -1.101312 0.985030

H -5.385181 -2.031842 -0.864621

H -4.086071 2.504554 -2.837763

H -5.705819 2.657136 -3.502718

H -5.658242 0.493241 -4.522709

H -4.621615 -1.411522 -3.515839

C -2.802446 -2.450362 -1.324028

O -3.478634 -3.123663 -2.112863

C -2.597500 -2.571866 0.067857

C -3.246648 -3.676309 0.809143

C -2.914884 -3.743186 2.228872

C -2.133352 -2.813117 2.842761

C -1.584368 -1.709657 2.120533

C -1.817866 -1.609282 0.780040

O -4.035726 -4.484144 0.295192

H -3.352961 -4.571661 2.776050

H -1.929815 -2.898377 3.907491

H -0.996255 -0.959027 2.637174

H -1.423840 -0.749556 0.248388

C -2.611838 -6.565914 -5.416838

C -2.502542 -7.416905 -6.720808

C -3.428179 -7.081854 -4.299276

O -2.011476 -5.513908 -5.405033

F -1.899862 -8.604155 -6.478633

F -3.721269 -7.678815 -7.242099

F -1.788730 -6.781520 -7.649979

C -3.610142 -6.237719 -3.190543

C -4.345823 -6.659132 -2.090923

C -4.910080 -7.935906 -2.093821

C -4.743088 -8.782448 -3.190820

C -4.007319 -8.363465 -4.292196

H -3.184111 -5.242864 -3.194358

H -4.457849 -5.990122 -1.244263

H -5.483543 -8.272998 -1.235704

H -5.185001 -9.773562 -3.186820

H -3.882900 -9.036068 -5.130381

TS1_4-B-minor_

C -2.643379 -1.472535 -2.275760

N -1.327379 -1.472388 -1.993041

N -0.712427 -0.301688 -2.384601

C -1.672978 0.409913 -2.886204

N -2.867090 -0.260526 -2.844067

C -4.079481 0.366342 -3.414872

C -1.626605 1.708829 -3.616023

C -0.438760 -2.499162 -1.534042

C -0.064841 -2.573106 -0.192732

C 0.888395 -3.490779 0.233463

C 1.492046 -4.308686 -0.711936

C 1.171794 -4.227034 -2.062163

C 0.209123 -3.314083 -2.470654

Cl -0.734104 -1.472067 0.982088

H 1.159438 -3.549969 1.278252

Cl 2.696160 -5.464166 -0.191233

H 1.653082 -4.867412 -2.788096

Cl -0.146485 -3.176413 -4.160894

O -2.331601 1.515095 -4.835608

H -2.051369 2.521476 -3.015062

H -0.598612 1.957522 -3.872792

C -3.714223 1.154114 -4.728738

C -4.709819 1.414349 -2.515977

C -4.634127 2.396105 -4.699852

C -5.022638 2.559560 -3.250779

C -5.648545 3.637906 -2.631264

C -5.956811 3.554839 -1.274083

C -5.656304 2.401392 -0.546738

C -5.034290 1.318294 -1.165697

H -5.894048 4.532811 -3.194803

H -6.440965 4.390759 -0.779985

H -5.914471 2.343250 0.505156

H -4.825882 0.417094 -0.599897

H -4.113870 3.258130 -5.124177

H -5.520171 2.220904 -5.320644

H -3.889439 0.528526 -5.602850

H -4.764968 -0.446022 -3.644417

C -3.809539 -2.484769 -2.136542

O -4.569758 -2.453414 -3.085514

C -4.154245 -3.034023 -0.808816

C -4.706534 -4.335867 -0.752266

C -5.229610 -4.790192 0.465827

C -5.213290 -3.977960 1.593102

C -4.632720 -2.709435 1.545921

C -4.101389 -2.248906 0.346183

O -4.702046 -5.132924 -1.821024

H -5.636773 -5.794103 0.500707

H -5.634630 -4.345464 2.523763

H -4.602613 -2.083873 2.431683

H -3.655582 -1.259533 0.309184

C -3.238517 -5.364057 -2.425128

C -3.591013 -5.687864 -3.911512

C -2.758883 -6.626270 -1.685955

O -2.479513 -4.318599 -2.310924

F -3.938494 -4.595726 -4.606857

F -2.492092 -6.205323 -4.516656

F -4.584290 -6.592033 -4.068986

C -1.487075 -6.628429 -1.118417

C -1.012765 -7.742656 -0.428578

C -1.814305 -8.873283 -0.296885

C -3.092302 -8.877338 -0.855248

C -3.564012 -7.762683 -1.543192

H -0.883303 -5.738476 -1.222306

H -0.017350 -7.725621 0.005530

H -1.449246 -9.744441 0.238800

H -3.727415 -9.752108 -0.751543

H -4.561844 -7.767998 -1.961128

Int1_4-B-minor_

C -2.698406 -1.596062 -2.262172

N -1.369621 -1.650738 -2.043008

N -0.716976 -0.517328 -2.482765

C -1.667157 0.228795 -2.951715

N -2.889098 -0.382219 -2.838641

C -4.089039 0.293896 -3.380952

C -1.592507 1.509881 -3.709769

C -0.516159 -2.686522 -1.544468

C -0.132589 -2.700855 -0.202161

C 0.789182 -3.628161 0.271182

C 1.347317 -4.527413 -0.627149

C 1.018873 -4.509675 -1.977209

C 0.100424 -3.574893 -2.435735

Cl -0.754069 -1.512738 0.913309

H 1.072000 -3.631775 1.314663

Cl 2.507254 -5.698654 -0.047128

H 1.468979 -5.209441 -2.667280

Cl -0.234911 -3.504066 -4.134287

O -2.351748 1.329136 -4.898326

H -1.955701 2.352186 -3.109191

H -0.564939 1.706668 -4.010071

C -3.741800 1.013556 -4.733770

C -4.623313 1.405289 -2.497332

C -4.619489 2.285630 -4.723932

C -4.926636 2.530947 -3.266346

C -5.474197 3.661181 -2.665931

C -5.714535 3.650470 -1.292305

C -5.424307 2.517483 -0.529805

C -4.881102 1.382411 -1.130073

H -5.711128 4.541078 -3.256309

H -6.137063 4.527378 -0.812776

H -5.629350 2.516356 0.535428

H -4.681064 0.496902 -0.537504

H -4.096043 3.108002 -5.217281

H -5.542189 2.107899 -5.288320

H -3.965199 0.361450 -5.577239

H -4.824427 -0.491656 -3.542191

C -3.898902 -2.579354 -2.064059

O -4.799867 -2.380959 -2.878592

C -4.225998 -3.063499 -0.681778

C -4.761480 -4.362314 -0.593347

C -5.259761 -4.826528 0.623372

C -5.233458 -4.004706 1.747628

C -4.677007 -2.729988 1.675502

C -4.176240 -2.267076 0.458616

O -4.807258 -5.168527 -1.680645

H -5.658649 -5.833553 0.666096

H -5.633039 -4.371047 2.687967

H -4.642698 -2.094763 2.554413

H -3.750016 -1.270132 0.403805

C -3.526663 -5.251875 -2.459751

C -4.030345 -5.573231 -3.902579

C -2.743825 -6.419171 -1.827242

O -2.830570 -4.106909 -2.445027

F -4.564158 -4.509004 -4.505021

F -2.987448 -5.982028 -4.666038

F -4.960148 -6.557157 -3.941367

C -1.915347 -6.125063 -0.740543

C -1.242875 -7.134678 -0.057206

C -1.376239 -8.461455 -0.458396

C -2.197309 -8.765293 -1.540845

C -2.883405 -7.756924 -2.214376

H -1.806928 -5.094351 -0.431564

H -0.610500 -6.883389 0.788719

H -0.847345 -9.250019 0.067876

H -2.312374 -9.795130 -1.864753

H -3.526204 -8.028474 -3.039806

A1-RC_1-A_

C -2.011683 -1.050700 -2.721687

N -0.681070 -0.905345 -2.799085

N -0.325827 0.330817 -3.287870

C -1.462716 0.934129 -3.458596

N -2.528247 0.138869 -3.093383

C -3.954261 0.485936 -3.396332

C -1.669840 2.323295 -3.969464

C 0.330859 -1.833889 -2.407502

C 0.917109 -1.740758 -1.138244

C 1.897251 -2.638322 -0.735640

C 2.300742 -3.631437 -1.622084

C 1.763189 -3.729162 -2.899328

C 0.786630 -2.820138 -3.292489

Cl 0.458154 -0.470191 -0.040876

H 2.335809 -2.559835 0.249545

Cl 3.526709 -4.766908 -1.124106

H 2.096454 -4.496047 -3.584526

Cl 0.160928 -2.903253 -4.907222

O -2.926067 2.440296 -4.601992

H -1.559113 3.038222 -3.143054

H -0.910455 2.550022 -4.716429

C -4.025437 1.981943 -3.836243

C -4.874254 0.468820 -2.194661

C -4.342139 2.781094 -2.549616

C -5.088626 1.769154 -1.719101

C -5.933310 1.989218 -0.635410

C -6.566921 0.900350 -0.037908

C -6.375442 -0.391626 -0.530522

C -5.532664 -0.615601 -1.618392

H -6.098398 2.993285 -0.259713

H -7.225787 1.060448 0.809293

H -6.892712 -1.227211 -0.071118

H -5.404951 -1.617894 -2.009636

H -3.433139 3.110669 -2.037216

H -4.924031 3.678992 -2.768522

H -4.871506 2.046498 -4.520543

H -4.236077 -0.189508 -4.201957

C -2.658295 -2.221947 -1.992881

O -2.472429 -2.168367 -0.753273

C -3.308374 -3.232456 -2.724527

C -3.417129 -3.125675 -4.178841

C -4.033807 -4.250462 -4.850168

C -4.512645 -5.320368 -4.152691

C -4.430567 -5.390031 -2.727239

C -3.843665 -4.369254 -2.040550

O -3.009239 -2.115011 -4.801615

H -4.102583 -4.198785 -5.931145

H -4.972424 -6.145008 -4.690647

H -4.823634 -6.254874 -2.205250

H -3.772409 -4.402985 -0.959068

C -4.000534 -0.818458 -7.324407

C -2.473163 -0.554884 -7.485517

C -4.632652 -1.876360 -8.153032

O -4.619872 -0.100442 -6.575222

F -2.019278 0.269725 -6.541373

F -1.731629 -1.677204 -7.458352

F -2.257096 0.036273 -8.687672

C -5.988541 -2.147248 -7.911037

C -6.657455 -3.108913 -8.653771

C -5.982728 -3.807066 -9.656885

C -4.639700 -3.538257 -9.911891

C -3.962148 -2.579897 -9.164787

H -6.496112 -1.590886 -7.132345

H -7.703529 -3.315915 -8.454810

H -6.504935 -4.558210 -10.240650

H -4.116575 -4.076252 -10.695109

H -2.920195 -2.385314 -9.379279

C -2.328938 -2.535991 2.709943

N -2.233602 -3.549781 1.783766

N -2.727042 -1.370188 2.086735

S -1.974290 -2.679634 4.336925

H -2.335903 -3.261788 0.811532

C -1.820496 -4.885348 1.923852

H -2.748172 -1.404298 1.070900

C -2.864911 -0.071284 2.601771

C -1.239808 -5.475754 0.795137

C -0.845676 -6.809696 0.814361

C -1.014179 -7.580690 1.958399

C -1.599857 -6.991843 3.078432

C -2.007879 -5.662739 3.074971

H -1.084557 -4.883959 -0.099061

C -0.277455 -7.425020 -0.435626

H -0.690415 -8.613304 1.981219

C -1.811602 -7.846324 4.301879

H -2.460774 -5.232360 3.952777

C -3.296908 0.226768 3.897733

C -3.425634 1.557391 4.293382

C -3.143871 2.605884 3.424983

C -2.738810 2.302771 2.126038

C -2.599806 0.983754 1.716587

H -3.535100 -0.567702 4.586153

C -3.839294 1.855049 5.712089

H -3.243384 3.633463 3.749442

C -2.474096 3.428234 1.166472

H -2.276037 0.761881 0.707585

F -4.404781 3.077225 5.825334

F -4.731820 0.956103 6.175962

F -2.783733 1.832275 6.553771

F -1.848212 3.008260 0.037313

F -3.620747 4.033218 0.764710

F -1.706109 4.391661 1.710471

F -2.274226 -7.144932 5.352125

F -2.701671 -8.837804 4.059637

F -0.664665 -8.447820 4.690019

F 0.552628 -8.452068 -0.166248

F -1.250905 -7.907443 -1.241337

F 0.419938 -6.525546 -1.170171

A1-TS1_1-A_

C -2.252770 -0.737658 -3.524256

N -1.239751 -1.125420 -2.725993

N -0.391584 -0.081368 -2.417594

C -0.920590 0.934686 -3.027688

N -2.062386 0.584617 -3.701170

C -2.807104 1.602149 -4.469064

C -0.404025 2.321231 -3.216438

C -0.966921 -2.383366 -2.103580

C -1.622851 -2.742481 -0.920331

C -1.310630 -3.922547 -0.259129

C -0.313418 -4.736742 -0.783178

C 0.377716 -4.392764 -1.938341

C 0.053452 -3.209146 -2.590889

Cl -2.842937 -1.701297 -0.242678

H -1.832632 -4.194229 0.647845

Cl 0.088231 -6.224380 0.037746

H 1.160141 -5.029264 -2.327816

Cl 0.941632 -2.766658 -4.014473

O -0.489581 2.599501 -4.605467

H -0.969153 3.039315 -2.609876

H 0.646996 2.371642 -2.937608

C -1.804756 2.593935 -5.178164

C -3.675262 2.500758 -3.606425

C -2.489951 3.975537 -5.080844

C -3.502108 3.837175 -3.971315

C -4.251723 4.832676 -3.349405

C -5.158322 4.475129 -2.353056

C -5.323904 3.136665 -1.989068

C -4.588827 2.134324 -2.620207

H -4.137716 5.872471 -3.638573

H -5.746554 5.243025 -1.861455

H -6.039439 2.872899 -1.217373

H -4.734745 1.090800 -2.369101

H -1.742428 4.753141 -4.904050

H -2.976866 4.213970 -6.031311

H -1.638751 2.288683 -6.208052

H -3.379550 1.055976 -5.213798

C -3.512468 -1.523364 -3.935926

O -4.549705 -0.981607 -3.492818

C -3.384107 -3.015306 -4.003367

C -2.568096 -3.619479 -4.966935

C -2.451585 -5.006074 -5.028522

C -3.155934 -5.793638 -4.123064

C -3.998975 -5.204772 -3.179023

C -4.115890 -3.820726 -3.127098

O -1.871091 -2.842881 -5.850256

H -1.820783 -5.445232 -5.792255

H -3.064296 -6.873819 -4.169486

H -4.567732 -5.818186 -2.489312

H -4.763688 -3.353195 -2.396962

C -2.699135 -1.872027 -6.575453

C -1.613725 -0.940159 -7.196823

C -3.486871 -2.613231 -7.664300

O -3.478796 -1.134284 -5.734141

F -0.738663 -1.572706 -7.992695

F -2.201054 0.032253 -7.924746

F -0.891012 -0.325374 -6.230113

C -4.854976 -2.397230 -7.825710

C -5.572908 -3.086151 -8.802093

C -4.928861 -4.003938 -9.625527

C -3.563206 -4.233549 -9.464222

C -2.846380 -3.545776 -8.490635

H -5.366288 -1.699262 -7.178938

H -6.636578 -2.903594 -8.913316

H -5.486640 -4.540911 -10.385918

H -3.053756 -4.953141 -10.097103

H -1.788394 -3.738055 -8.368262

C -7.502236 -0.166567 -5.400130

S -9.039856 0.296828 -5.863105

N -7.201399 -1.197074 -4.546322

N -6.342640 0.438671 -5.843276

H -5.478484 -0.080805 -5.711330

C -6.208244 1.649773 -6.547867

H -6.253148 -1.194424 -4.157594

C -8.052283 -2.151028 -3.965424

C -7.719659 -2.597457 -2.684212

C -8.449381 -3.616101 -2.076323

C -9.539456 -4.187626 -2.720126

C -9.878616 -3.727499 -3.993114

C -9.146967 -2.727511 -4.623087

H -6.884772 -2.141261 -2.165316

C -8.007596 -4.097036 -0.723087

H -10.108257 -4.978436 -2.250398

C -11.085798 -4.310799 -4.680435

H -9.411772 -2.406451 -5.618218

C -5.226382 1.732154 -7.537648

C -5.004216 2.936611 -8.205760

C -5.760566 4.063402 -7.909559

C -6.734596 3.975028 -6.913800

C -6.962091 2.787304 -6.231793

H -4.647145 0.855051 -7.798203

C -3.891836 3.023917 -9.214317

H -5.603469 4.990429 -8.445673

C -7.487858 5.221612 -6.532333

H -7.708787 2.742313 -5.454251

F -4.099537 3.996775 -10.123118

F -2.704572 3.311201 -8.607454

F -3.710999 1.871513 -9.883224

F -8.585679 4.962026 -5.800923

F -6.709805 6.056575 -5.795596

F -7.877416 5.924216 -7.617912

F -11.020750 -4.187677 -6.021156

F -11.230443 -5.627115 -4.404370

F -12.225964 -3.704438 -4.282060

F -8.844632 -5.007411 -0.194223

F -6.777166 -4.679206 -0.780642

F -7.898258 -3.083629 0.164589

A1-Int1_1-A_

C -2.265706 -0.755346 -3.314302

N -1.077540 -1.097165 -2.779533

N -0.240521 -0.013749 -2.650811

C -0.946930 0.980506 -3.101417

N -2.193566 0.575498 -3.497655

C -3.136067 1.544698 -4.085897

C -0.566357 2.398516 -3.363485

C -0.617848 -2.358829 -2.290547

C -1.104357 -2.872126 -1.081907

C -0.640140 -4.078966 -0.577643

C 0.344516 -4.760994 -1.282944

C 0.877258 -4.256874 -2.462657

C 0.400574 -3.049844 -2.962184

Cl -2.292232 -1.988800 -0.160640

H -1.033410 -4.469908 0.350419

Cl 0.936416 -6.280923 -0.661879

H 1.654766 -4.789834 -2.992249

Cl 1.098463 -2.421340 -4.417717

O -0.967691 2.696990 -4.690211

H -1.024655 3.068159 -2.625124

H 0.515550 2.510730 -3.319244

C -2.363683 2.567440 -5.002529

C -3.834589 2.421381 -3.060184

C -3.120680 3.898292 -4.808587

C -3.833566 3.750917 -3.487728

C -4.475511 4.728358 -2.731938

C -5.098980 4.361957 -1.540166

C -5.092488 3.031829 -1.114168

C -4.466656 2.046854 -1.876762

H -4.493911 5.760364 -3.067239

H -5.598954 5.115881 -0.940946

H -5.589113 2.759606 -0.188829

H -4.489538 1.009317 -1.567202

H -2.421727 4.737065 -4.845950

H -3.839338 4.045150 -5.621177

H -2.368191 2.230308 -6.036071

H -3.830867 0.963752 -4.686253

C -3.541240 -1.582161 -3.393537

O -4.539565 -1.009039 -2.969255

C -3.471841 -3.028749 -3.638432

C -2.678329 -3.582734 -4.664770

C -2.650146 -4.973223 -4.829266

C -3.391502 -5.794338 -3.991762

C -4.204728 -5.246373 -2.992907

C -4.252230 -3.872952 -2.828364

O -1.966811 -2.794891 -5.491770

H -2.048700 -5.381110 -5.632419

H -3.354619 -6.870396 -4.128010

H -4.797176 -5.887277 -2.349890

H -4.869923 -3.437816 -2.053151

C -2.839448 -1.869788 -6.421729

C -1.691333 -0.995930 -7.026000

C -3.460810 -2.802117 -7.472330

O -3.667960 -1.144382 -5.717439

F -0.666337 -1.679979 -7.565318

F -2.192526 -0.197317 -7.993301

F -1.154073 -0.178208 -6.087815

C -4.841823 -2.782648 -7.669808

C -5.442212 -3.623156 -8.605370

C -4.665967 -4.500869 -9.355449

C -3.286356 -4.536894 -9.158007

C -2.687562 -3.698132 -8.223089

H -5.453649 -2.113656 -7.081326

H -6.517745 -3.588379 -8.745318

H -5.130842 -5.154188 -10.086841

H -2.672198 -5.222252 -9.733723

H -1.617140 -3.745169 -8.074350

C -7.336225 -0.202884 -5.540110

S -8.856177 0.287624 -6.026220

N -7.083442 -1.207881 -4.633884

N -6.151302 0.334366 -5.997863

H -5.315800 -0.261041 -5.942650

C -5.992230 1.515949 -6.745565

H -6.174396 -1.176925 -4.179661

C -7.983856 -2.114884 -4.048720

C -7.760077 -2.477182 -2.719791

C -8.552019 -3.447865 -2.109322

C -9.594022 -4.051086 -2.801224

C -9.822955 -3.673987 -4.125561

C -9.028576 -2.724049 -4.755565

H -6.961503 -1.997043 -2.167099

C -8.227795 -3.844962 -0.696884

H -10.209396 -4.804446 -2.328493

C -10.974911 -4.295065 -4.871824

H -9.205889 -2.468151 -5.788573

C -5.046068 1.529289 -7.773046

C -4.797785 2.703782 -8.482204

C -5.489671 3.872610 -8.188198

C -6.427982 3.853894 -7.155411

C -6.681245 2.694501 -6.432655

H -4.511201 0.622838 -8.023995

C -3.719664 2.710824 -9.532434

H -5.309767 4.777563 -8.753324

C -7.095784 5.141604 -6.754988

H -7.402009 2.703023 -5.628665

F -3.900952 3.690797 -10.441816

F -2.498728 2.918585 -8.975405

F -3.648918 1.544682 -10.200307

F -8.256441 4.944625 -6.105743

F -6.297642 5.863044 -5.920202

F -7.349572 5.933737 -7.816862

F -10.804631 -4.244394 -6.208190

F -11.143515 -5.594808 -4.537225

F -12.141203 -3.670547 -4.597305

F -9.129387 -4.695166 -0.174097

F -7.014952 -4.461977 -0.621201

F -8.153078 -2.776119 0.126451

A1-TS1'_1-A_

C -2.200911 -0.782678 -3.477164

N -1.185320 -1.159143 -2.676776

N -0.342892 -0.108227 -2.376149

C -0.877949 0.900565 -2.992972

N -2.018095 0.539406 -3.663409

C -2.768959 1.547290 -4.438185

C -0.369159 2.288574 -3.191691

C -0.904883 -2.411223 -2.045971

C -1.558155 -2.766199 -0.860020

C -1.238726 -3.939961 -0.191080

C -0.236975 -4.751787 -0.710125

C 0.451703 -4.411476 -1.867840

C 0.120210 -3.234180 -2.528136

Cl -2.784074 -1.727676 -0.188822

H -1.758748 -4.208634 0.717925

Cl 0.173624 -6.231580 0.120479

H 1.237729 -5.045931 -2.253378

Cl 1.005212 -2.795863 -3.955007

O -0.456865 2.556351 -4.582608

H -0.938007 3.007824 -2.589994

H 0.681670 2.346776 -2.913654

C -1.772447 2.540331 -5.154023

C -3.642638 2.446363 -3.581680

C -2.464847 3.918886 -5.064459

C -3.476651 3.781490 -3.954503

C -4.232013 4.776577 -3.338902

C -5.137146 4.420070 -2.340860

C -5.295594 3.082924 -1.968953

C -4.554730 2.080872 -2.593731

H -4.123495 5.815239 -3.634184

H -5.729830 5.187677 -1.854184

H -6.010065 2.819861 -1.196031

H -4.695002 1.038092 -2.336417

H -1.721409 4.701310 -4.891692

H -2.952551 4.149648 -6.016391

H -1.605745 2.229751 -6.182217

H -3.337958 0.992522 -5.179190

C -3.456411 -1.578571 -3.881833

O -4.496367 -1.039862 -3.441564

C -3.319442 -3.070118 -3.939432

C -2.500884 -3.676228 -4.899641

C -2.376223 -5.062562 -4.951449

C -3.075079 -5.847866 -4.039819

C -3.920706 -5.257347 -3.099124

C -4.045581 -3.873686 -3.056766

O -1.809567 -2.901992 -5.789377

H -1.743441 -5.503424 -5.712559

H -2.977083 -6.927786 -4.078725

H -4.485227 -5.869186 -2.404558

H -4.695222 -3.404864 -2.329106

C -2.643963 -1.940841 -6.520367

C -1.564153 -1.007639 -7.149449

C -3.428819 -2.694055 -7.602997

O -3.426624 -1.201429 -5.683445

F -0.686718 -1.641098 -7.941990

F -2.157354 -0.043329 -7.883378

F -0.843566 -0.382510 -6.187787

C -4.798151 -2.486231 -7.764779

C -5.513415 -3.185821 -8.735510

C -4.865368 -4.106236 -9.552842

C -3.498419 -4.327680 -9.391102

C -2.784276 -3.629264 -8.423140

H -5.312472 -1.786246 -7.122605

H -6.578097 -3.009474 -8.847085

H -5.421009 -4.651549 -10.308850

H -2.985861 -5.049205 -10.019256

H -1.725215 -3.815216 -8.300327

C -7.455381 -0.254108 -5.351904

S -8.996286 0.197305 -5.815724

N -7.147372 -1.277646 -4.492277

N -6.299840 0.355185 -5.800204

H -5.432735 -0.159046 -5.667066

C -6.173070 1.563123 -6.511499

H -6.198825 -1.266709 -4.104456

C -7.991990 -2.232598 -3.903894

C -7.655203 -2.668429 -2.620132

C -8.378256 -3.687126 -2.004421

C -9.465826 -4.269228 -2.642988

C -9.809240 -3.819643 -3.918594

C -9.084151 -2.819735 -4.556259

H -6.822319 -2.203849 -2.105467

C -7.931896 -4.156372 -0.648596

H -10.029540 -5.060082 -2.167221

C -11.014094 -4.414149 -4.600374

H -9.351957 -2.506939 -5.553223

C -5.191779 1.646050 -7.501821

C -4.977383 2.847944 -8.177030

C -5.740872 3.971633 -7.887350

C -6.714168 3.882865 -6.890922

C -6.934040 2.697725 -6.201940

H -4.606945 0.771160 -7.757312

C -3.865964 2.936117 -9.186574

H -5.589843 4.896437 -8.428992

C -7.475659 5.126632 -6.516569

H -7.680403 2.652568 -5.424095

F -4.079011 3.903600 -10.099828

F -2.679756 3.232176 -8.581761

F -3.679679 1.781466 -9.850119

F -8.573750 4.863596 -5.786770

F -6.704493 5.969308 -5.781403

F -7.866685 5.822316 -7.606073

F -10.951145 -4.300137 -5.942005

F -11.151646 -5.729204 -4.314885

F -12.156931 -3.810853 -4.205007

F -8.763467 -5.067224 -0.112021

F -6.698636 -4.732754 -0.704131

F -7.826188 -3.136275 0.231821

A1-Int1'_1-A_

C -1.807220 -0.558794 -3.375019

N -0.871429 -1.033625 -2.529761

N -0.003719 -0.046239 -2.098758

C -0.443452 1.023798 -2.683144

N -1.545110 0.762329 -3.458189

C -2.223574 1.872359 -4.154554

C 0.142703 2.394365 -2.750581

C -0.671627 -2.331757 -1.966036

C -1.344169 -2.701017 -0.795511

C -1.083292 -3.914087 -0.172809

C -0.122170 -4.753693 -0.723612

C 0.582109 -4.404451 -1.869313

C 0.309928 -3.186330 -2.480656

Cl -2.523195 -1.632106 -0.090233

H -1.616661 -4.192242 0.725611

Cl 0.215119 -6.282678 0.049575

H 1.335632 -5.061833 -2.280491

Cl 1.218103 -2.731366 -3.889218

O 0.130339 2.773195 -4.119115

H -0.412766 3.096392 -2.117401

H 1.182013 2.373083 -2.427408

C -1.160357 2.881278 -4.734940

C -3.073183 2.721492 -3.224685

C -1.792449 4.276197 -4.525416

C -2.830120 4.077707 -3.448423

C -3.532702 5.039299 -2.726502

C -4.466368 4.625654 -1.777464

C -4.702285 3.266616 -1.556475

C -4.010591 2.299241 -2.284292

H -3.357862 6.096954 -2.897592

H -5.018534 5.366679 -1.208776

H -5.435976 2.960536 -0.818186

H -4.204329 1.242318 -2.140758

H -1.019042 5.003866 -4.266434

H -2.252729 4.616653 -5.457677

H -0.969491 2.665853 -5.783512

H -2.801296 1.432033 -4.962203

C -3.060743 -1.251035 -4.014079

O -4.119712 -0.733045 -3.488520

C -2.951175 -2.766912 -4.044130

C -2.173946 -3.378202 -5.027405

C -2.055604 -4.761549 -5.102264

C -2.732792 -5.546406 -4.173464

C -3.535041 -4.954296 -3.196154

C -3.644590 -3.567465 -3.137359

O -1.499743 -2.585665 -5.928372

H -1.451098 -5.200178 -5.887484

H -2.651884 -6.627002 -4.225573

H -4.082107 -5.569960 -2.492021

H -4.258746 -3.090417 -2.382936

C -2.311468 -1.539594 -6.477458

C -1.269018 -0.585475 -7.128669

C -3.299297 -2.109651 -7.496277

O -2.956692 -0.779401 -5.496666

F -0.536223 -1.178362 -8.081518

F -1.886836 0.475893 -7.676612

F -0.408709 -0.111757 -6.198644

C -4.659421 -1.809323 -7.417386

C -5.556751 -2.354585 -8.334317

C -5.100957 -3.205688 -9.336156

C -3.744049 -3.517045 -9.414064

C -2.847054 -2.976223 -8.499182

H -5.019470 -1.160125 -6.631169

H -6.611312 -2.112022 -8.258801

H -5.798927 -3.629191 -10.050998

H -3.382524 -4.186036 -10.188232

H -1.796893 -3.232154 -8.561406

C -7.341210 0.067618 -4.423451

S -8.906214 0.414755 -4.910939

N -6.979870 -0.991877 -3.628345

N -6.229878 0.774905 -4.816321

H -5.327398 0.389373 -4.527325

C -6.184280 1.957892 -5.580987

H -5.972682 -1.153441 -3.529208

C -7.809214 -1.941325 -3.003863

C -7.364587 -3.263887 -2.961081

C -8.094840 -4.233661 -2.274934

C -9.289624 -3.908336 -1.646490

C -9.737155 -2.587656 -1.702995

C -9.009877 -1.605506 -2.364066

H -6.454459 -3.538033 -3.481246

C -7.546742 -5.631307 -2.205337

H -9.867984 -4.664222 -1.132597

C -10.995952 -2.214840 -0.964321

H -9.367609 -0.587762 -2.388054

C -5.204878 2.071855 -6.567975

C -5.073046 3.257392 -7.294638

C -5.921570 4.330530 -7.060459

C -6.900033 4.209928 -6.071526

C -7.035290 3.042891 -5.332433

H -4.559728 1.228499 -6.784309

C -3.978141 3.360302 -8.319430

H -5.833145 5.240530 -7.638871

C -7.764370 5.403028 -5.760680

H -7.789845 2.970067 -4.564421

F -4.120223 4.430398 -9.123827

F -2.755974 3.485488 -7.721270

F -3.903540 2.268533 -9.102351

F -8.880200 5.071448 -5.086309

F -7.097380 6.307316 -5.000438

F -8.144415 6.052827 -6.882516

F -11.562382 -1.093102 -1.445823

F -11.922251 -3.196607 -1.024934

F -10.749535 -1.998494 0.351012

F -8.448553 -6.522257 -1.754032

F -7.110751 -6.068539 -3.406752

F -6.474690 -5.699842 -1.367232

A1-TS2_1-A_

C -1.896053 -0.674022 -3.473722

N -0.969984 -1.126480 -2.605385

N -0.080289 -0.150806 -2.162539

C -0.499002 0.913509 -2.768806

N -1.592118 0.644142 -3.561833

C -2.254906 1.740160 -4.281054

C 0.092092 2.281009 -2.840912

C -0.800030 -2.432518 -2.065075

C -1.478790 -2.815424 -0.902698

C -1.254648 -4.051848 -0.310588

C -0.328404 -4.909741 -0.890869

C 0.371213 -4.560226 -2.038887

C 0.132369 -3.318534 -2.616271

Cl -2.624534 -1.730244 -0.163728

H -1.789027 -4.333798 0.586100

Cl -0.035933 -6.469461 -0.156699

H 1.093498 -5.235018 -2.477066

Cl 1.020895 -2.871628 -4.043319

O 0.091862 2.658095 -4.212586

H -0.465936 2.990837 -2.217937

H 1.129207 2.263952 -2.510489

C -1.193017 2.764420 -4.837208

C -3.149573 2.581688 -3.387282

C -1.834990 4.156692 -4.626848

C -2.912458 3.941289 -3.592865

C -3.650244 4.891404 -2.890534

C -4.616290 4.463182 -1.981237

C -4.846541 3.100511 -1.777150

C -4.114834 2.145963 -2.482394

H -3.476099 5.951947 -3.044719

H -5.195912 5.195030 -1.428079

H -5.603494 2.782386 -1.067938

H -4.300379 1.086973 -2.340343

H -1.072853 4.880131 -4.325509

H -2.261030 4.514727 -5.568618

H -0.990260 2.561230 -5.886719

H -2.807645 1.294835 -5.104712

C -3.575634 -1.446508 -4.370310

O -4.524281 -0.894785 -3.785931

C -3.315102 -2.914658 -4.268046

C -2.470256 -3.518159 -5.198622

C -2.191952 -4.877974 -5.148116

C -2.784326 -5.647417 -4.150356

C -3.647676 -5.064750 -3.219991

C -3.906770 -3.699209 -3.277728

O -1.882713 -2.737038 -6.168662

H -1.533166 -5.314318 -5.889642

H -2.583916 -6.712697 -4.108518

H -4.126419 -5.672886 -2.461766

H -4.557565 -3.225816 -2.552425

C -2.748930 -1.743049 -6.690286

C -1.801821 -0.781016 -7.459967

C -3.811243 -2.355623 -7.600550

O -3.329179 -0.930045 -5.683462

F -1.150335 -1.409180 -8.447339

F -2.503831 0.231345 -7.995075

F -0.879615 -0.247720 -6.636164

C -5.141242 -1.936220 -7.547782

C -6.092750 -2.508978 -8.389605

C -5.720803 -3.501647 -9.291270

C -4.393177 -3.923222 -9.348119

C -3.441701 -3.354932 -8.508129

H -5.440973 -1.171128 -6.844346

H -7.123675 -2.176599 -8.334758

H -6.462037 -3.947363 -9.946360

H -4.097198 -4.698443 -10.047138

H -2.412417 -3.688429 -8.554340

C -7.800434 0.008108 -4.594757

S -9.357371 0.323134 -5.121167

N -7.437238 -1.056109 -3.803523

N -6.694089 0.750164 -4.939032

H -5.789754 0.370875 -4.656634

C -6.639166 1.931678 -5.705991

H -6.435091 -1.206263 -3.692345

C -8.243561 -2.054083 -3.225951

C -7.710612 -3.342164 -3.147442

C -8.411019 -4.361079 -2.503916

C -9.663234 -4.120909 -1.954314

C -10.197918 -2.834856 -2.046182

C -9.502850 -1.802862 -2.664624

H -6.751620 -3.553178 -3.606119

C -7.767717 -5.715799 -2.397470

H -10.217706 -4.915285 -1.472952

C -11.527789 -2.556916 -1.394325

H -9.930517 -0.813854 -2.715301

C -5.590097 2.074870 -6.613617

C -5.445534 3.256332 -7.342946

C -6.350870 4.296969 -7.191986

C -7.399266 4.147474 -6.281827

C -7.547796 2.985013 -5.537983

H -4.893010 1.258421 -6.763465

C -4.291028 3.370621 -8.298863

H -6.249673 5.204966 -7.771282

C -8.337643 5.305896 -6.067230

H -8.357615 2.891856 -4.831209

F -4.283679 4.539197 -8.966218

F -3.097602 3.287316 -7.641584

F -4.285206 2.381036 -9.212904

F -9.472009 4.942465 -5.441771

F -7.760907 6.272613 -5.311994

F -8.685834 5.889185 -7.235251

F -12.109751 -1.439804 -1.867212

F -12.395840 -3.576237 -1.576772

F -11.395763 -2.398145 -0.055303

F -8.616796 -6.661197 -1.955542

F -7.271687 -6.137153 -3.581216

F -6.715964 -5.695587 -1.532950

A1-PC_1-A_

C -2.785729 -0.868389 -3.671838

N -2.157786 -1.251779 -2.532928

N -1.386159 -0.274125 -1.893339

C -1.562545 0.748469 -2.666718

N -2.379406 0.436139 -3.731034

C -2.765955 1.465030 -4.688568

C -0.959029 2.111764 -2.634247

C -2.239783 -2.534365 -1.936971

C -3.350242 -2.902322 -1.167101

C -3.447519 -4.158249 -0.577451

C -2.397373 -5.051143 -0.745221

C -1.268862 -4.720912 -1.484639

C -1.204790 -3.465850 -2.079315

Cl -4.649751 -1.766662 -0.911298

H -4.323799 -4.427056 -0.004742

Cl -2.497173 -6.636327 -0.004913

H -0.454817 -5.423071 -1.600959

Cl 0.201267 -3.066864 -3.030700

O -0.555010 2.438045 -3.962182

H -1.668231 2.848382 -2.234966

H -0.061624 2.123148 -2.017889

C -1.584674 2.469512 -4.958483

C -3.899666 2.349379 -4.202889

C -2.258216 3.860276 -5.071803

C -3.613048 3.695857 -4.426141

C -4.543406 4.672913 -4.078385

C -5.755115 4.283935 -3.508253

C -6.035640 2.933833 -3.282212

C -5.104622 1.955400 -3.628108

H -4.331315 5.724660 -4.246738

H -6.490036 5.035955 -3.240214

H -6.984980 2.644517 -2.844947

H -5.314649 0.905371 -3.449703

H -1.632124 4.622008 -4.599786

H -2.362183 4.138316 -6.126053

H -1.064324 2.197503 -5.875778

H -3.032539 0.953839 -5.613447

C -4.456697 -2.448284 -6.308636

O -5.555764 -2.029653 -6.010874

C -3.887549 -3.726616 -5.885968

C -2.534021 -3.975056 -6.134504

C -1.915909 -5.120819 -5.647903

C -2.674423 -6.035605 -4.926354

C -4.036425 -5.817237 -4.687961

C -4.637241 -4.663003 -5.161935

O -1.786300 -3.068899 -6.836732

H -0.861310 -5.278020 -5.838976

H -2.200645 -6.935432 -4.548934

H -4.615246 -6.536571 -4.121278

H -5.686299 -4.467887 -4.977199

C -2.520338 -2.253318 -7.720575

C -1.577989 -1.053419 -8.016178

C -2.922685 -2.986286 -8.993559

O -3.662344 -1.661107 -7.086788

F -0.470181 -1.480056 -8.636868

F -2.192146 -0.160876 -8.802926

F -1.209306 -0.431751 -6.887171

C -4.034720 -2.568997 -9.730040

C -4.369590 -3.220869 -10.913624

C -3.594244 -4.283125 -11.372380

C -2.476280 -4.689827 -10.647219

C -2.136287 -4.041131 -9.464284

H -4.634228 -1.735928 -9.384909

H -5.236149 -2.894090 -11.478200

H -3.857840 -4.789724 -12.294850

H -1.865393 -5.512627 -11.002880

H -1.261541 -4.353026 -8.907152

C -8.358961 -0.146647 -4.942739

S -9.602060 0.786773 -4.328617

N -8.026543 -1.403621 -4.476324

N -7.496208 0.215866 -5.950049

H -6.775733 -0.468050 -6.163172

C -7.328527 1.403803 -6.678915

H -7.143785 -1.779017 -4.806742

C -8.667013 -2.236062 -3.543732

C -7.854802 -3.154544 -2.866501

C -8.412142 -4.090043 -2.001198

C -9.783972 -4.113326 -1.768600

C -10.586341 -3.190707 -2.434792

C -10.048856 -2.262222 -3.321967

H -6.781421 -3.131271 -3.018074

C -7.507730 -5.055725 -1.286409

H -10.220172 -4.842950 -1.100464

C -12.071838 -3.170900 -2.177095

H -10.696193 -1.577809 -3.846778

C -6.128903 1.504179 -7.396746

C -5.857023 2.622434 -8.175044

C -6.768805 3.669128 -8.258744

C -7.962356 3.563068 -7.549014

C -8.256537 2.449505 -6.767660

H -5.400797 0.701744 -7.337993

C -4.551130 2.669873 -8.918538

H -6.560084 4.539139 -8.865778

C -8.937259 4.711970 -7.582452

H -9.190304 2.389128 -6.233348

F -4.386957 3.813034 -9.607806

F -3.490814 2.561024 -8.073270

F -4.436243 1.648807 -9.798535

F -10.205299 4.314493 -7.362728

F -8.643839 5.636261 -6.638502

F -8.915772 5.353378 -8.771953

F -12.772890 -2.877142 -3.291626

F -12.520282 -4.360889 -1.721926

F -12.401510 -2.245929 -1.250159

F -8.158293 -6.157169 -0.869870

F -6.485577 -5.468768 -2.079411

F -6.933691 -4.500592 -0.190538

A1-TS2_1-A'_

C -2.081366 -1.963522 -3.639978

N -0.887487 -2.280870 -3.104437

N -0.023253 -1.196473 -2.967080

C -0.734838 -0.208732 -3.408949

N -1.985053 -0.623326 -3.811549

C -2.968371 0.359088 -4.300222

C -0.347297 1.198316 -3.714989

C -0.419915 -3.547724 -2.654149

C -0.678054 -3.979078 -1.348119

C -0.171209 -5.182573 -0.874240

C 0.611772 -5.956660 -1.721739

C 0.899436 -5.554681 -3.020050

C 0.383304 -4.347185 -3.474444

Cl -1.643323 -2.999116 -0.279805

H -0.382228 -5.503987 0.136290

Cl 1.253006 -7.474791 -1.138343

H 1.514210 -6.163593 -3.668308

Cl 0.763920 -3.834680 -5.093843

O -0.823859 1.454115 -5.036447

H -0.765847 1.906400 -2.991511

H 0.734963 1.309740 -3.732174

C -2.250990 1.445874 -5.189224

C -3.619924 1.160408 -3.187337

C -2.891493 2.794499 -4.784450

C -3.564923 2.527502 -3.460935

C -4.126241 3.440919 -2.571128

C -4.739974 2.966408 -1.413011

C -4.805734 1.594909 -1.153707

C -4.250573 0.676317 -2.043107

H -4.086864 4.507039 -2.773866

H -5.176910 3.668541 -0.709961

H -5.301281 1.239957 -0.255989

H -4.329773 -0.391276 -1.872810

H -2.131762 3.578756 -4.754712

H -3.623880 3.100649 -5.539970

H -2.396098 1.225184 -6.245622

H -3.700685 -0.195152 -4.881870

C -3.790586 -2.832831 -3.930180

O -4.573226 -2.325401 -3.142342

C -3.476577 -4.301319 -3.949100

C -2.942558 -4.903085 -5.088744

C -2.624553 -6.256507 -5.110299

C -2.858386 -7.020067 -3.969756

C -3.427092 -6.442686 -2.832661

C -3.739792 -5.087500 -2.827456

O -2.719489 -4.142649 -6.220069

H -2.218035 -6.698111 -6.011960

H -2.616017 -8.077635 -3.979497

H -3.630968 -7.050655 -1.957832

H -4.192761 -4.613048 -1.964664

C -3.730259 -3.155809 -6.440944

C -3.095138 -2.236300 -7.520162

C -5.028407 -3.787457 -6.944800

O -3.935580 -2.318817 -5.350579

F -2.790405 -2.895499 -8.658186

F -3.895885 -1.216946 -7.835209

F -1.918419 -1.699291 -7.073831

C -6.257791 -3.292457 -6.506294

C -7.443006 -3.855374 -6.974545

C -7.407557 -4.909695 -7.883399

C -6.180796 -5.407101 -8.320626

C -4.994384 -4.849544 -7.854636

H -6.278177 -2.484469 -5.786242

H -8.394585 -3.470577 -6.622693

H -8.331952 -5.347514 -8.246325

H -6.145606 -6.234531 -9.021643

H -4.045610 -5.251410 -8.188482

C 1.411200 -1.713541 -9.090897

S 2.832024 -1.713276 -9.958479

N 0.743950 -0.578547 -8.673601

N 0.726706 -2.842134 -8.683442

H -0.230726 -2.691405 -8.394121

C 1.036670 -4.205313 -8.862982

H 0.056238 -0.708834 -7.942990

C 1.060770 0.774874 -8.926002

C -0.046731 -5.084618 -8.983499

C 0.167669 -6.450168 -9.113299

C 1.460921 -6.969073 -9.136268

C 2.531551 -6.092113 -9.005446

C 2.335445 -4.719261 -8.860666

H -1.057662 -4.696832 -8.981216

C -1.003971 -7.390176 -9.183979

H 1.629232 -8.031886 -9.248163

C 3.943586 -6.617820 -9.058936

H 3.182153 -4.062369 -8.740786

C 0.862423 1.692238 -7.891747

C 1.111834 3.047338 -8.102524

C 1.567730 3.502847 -9.334403

C 1.750875 2.582039 -10.363995

C 1.493988 1.227976 -10.176112

H 0.508970 1.357339 -6.921917

C 0.855017 4.018246 -6.981109

H 1.768304 4.553051 -9.494568

C 2.274200 3.048790 -11.698130

H 1.617951 0.535257 -10.994039

F 1.385730 5.232291 -7.224297

F -0.474077 4.206030 -6.778507

F 1.366245 3.586318 -5.806838

F 1.734631 2.353896 -12.721498

F 2.008180 4.355137 -11.913718

F 3.613374 2.900695 -11.785531

F -0.797610 -8.394687 -10.055978

F -1.250170 -7.970006 -7.979476

F -2.144991 -6.764106 -9.548645

F 4.749198 -5.980718 -8.183206

F 4.001989 -7.936393 -8.772931

F 4.487623 -6.454985 -10.283241

A3-TS2_1-A_

C -1.722349 -0.647403 -3.464154

N -0.688377 -1.023371 -2.687434

N 0.273996 -0.032548 -2.501508

C -0.211925 0.964247 -3.170624

N -1.412622 0.638066 -3.758534

C -2.142196 1.649039 -4.529216

C 0.372362 2.299944 -3.487318

C -0.463547 -2.270711 -2.039780

C -0.963294 -2.505895 -0.754320

C -0.686535 -3.686979 -0.077509

C 0.109263 -4.640374 -0.700680

C 0.629691 -4.438759 -1.972732

C 0.342257 -3.249591 -2.632856

Cl -1.949455 -1.303461 0.033051

H -1.081425 -3.854359 0.914992

Cl 0.464721 -6.132611 0.138738

H 1.250643 -5.187491 -2.444795

Cl 1.005848 -2.988844 -4.219177

O 0.195427 2.523108 -4.882022

H -0.093244 3.087910 -2.882919

H 1.444403 2.299946 -3.297760

C -1.150771 2.521902 -5.378968

C -2.867207 2.660976 -3.658796

C -1.777814 3.934859 -5.367670

C -2.656708 3.954638 -4.139531

C -3.252252 5.040253 -3.502460

C -4.052125 4.813033 -2.382187

C -4.261372 3.515840 -1.908232

C -3.671261 2.424972 -2.546882

H -3.094367 6.050881 -3.866605

H -4.516505 5.651921 -1.873950

H -4.890898 3.354601 -1.039513

H -3.845391 1.413991 -2.194627

H -0.995700 4.697707 -5.370339

H -2.378740 4.084862 -6.271477

H -1.057981 2.118070 -6.386018

H -2.828874 1.124458 -5.188198

C -3.506694 -1.415068 -4.074159

O -4.366800 -0.743404 -3.478457

C -3.327596 -2.874915 -3.810628

C -2.689880 -3.670789 -4.760125

C -2.508118 -5.034062 -4.553793

C -2.983850 -5.606962 -3.378725

C -3.636960 -4.827400 -2.421117

C -3.804117 -3.464405 -2.638311

O -2.196948 -3.097194 -5.907740

H -2.010866 -5.624720 -5.314347

H -2.860301 -6.672179 -3.216021

H -4.026266 -5.285459 -1.519604

H -4.295697 -2.836846 -1.903673

C -2.976535 -2.033171 -6.426220

C -1.990423 -1.287455 -7.369511

C -4.202565 -2.539164 -7.180580

O -3.340724 -1.070790 -5.454738

F -1.555891 -2.088379 -8.351333

F -2.586071 -0.220191 -7.927995

F -0.913486 -0.840430 -6.694100

C -5.356939 -1.755971 -7.252492

C -6.466543 -2.211418 -7.959888

C -6.426821 -3.445036 -8.605482

C -5.270695 -4.220971 -8.548030

C -4.159552 -3.769374 -7.842155

H -5.383542 -0.792944 -6.759792

H -7.362804 -1.601612 -8.000307

H -7.293976 -3.800683 -9.152215

H -5.233286 -5.181001 -9.052175

H -3.259909 -4.370933 -7.800853

C -7.666036 0.058400 -4.394456

O -8.759357 0.351429 -4.833827

N -7.325708 -1.164463 -3.840482

N -6.586801 0.940143 -4.390175

H -5.656332 0.575631 -4.191222

C -6.708529 2.245059 -4.878487

H -6.360898 -1.269353 -3.539309

C -8.157771 -2.276377 -3.680539

C -7.580085 -3.447270 -3.172300

C -8.349369 -4.586443 -2.965092

C -9.707983 -4.595698 -3.265077

C -10.276660 -3.432652 -3.778173

C -9.527139 -2.278627 -3.987546

H -6.520651 -3.467970 -2.944567

C -7.670536 -5.819744 -2.435395

H -10.304916 -5.482883 -3.108349

C -11.755188 -3.389403 -4.065399

H -9.984025 -1.387262 -4.388921

C -5.828026 2.724275 -5.849359

C -5.885096 4.026178 -6.321596

C -6.867195 4.884635 -5.846732

C -7.766270 4.432379 -4.888520

C -7.669779 3.138217 -4.392577

F -4.876691 1.905262 -6.339851

F -4.998850 4.453659 -7.232518

F -6.938513 6.140337 -6.297877

F -8.696340 5.265356 -4.410574

F -8.496723 2.772658 -3.413295

F -12.035258 -2.679953 -5.178181

F -12.276031 -4.624610 -4.236084

F -12.442103 -2.813086 -3.052276

F -8.543242 -6.793882 -2.113584

F -6.802640 -6.344904 -3.331712

F -6.944512 -5.551849 -1.319630

A3-TS2_1-A-minor_

C -2.237959 -0.936140 -3.193173

N -1.022440 -1.029759 -2.618381

N -0.444764 0.199474 -2.307512

C -1.348891 1.044111 -2.689055

N -2.443361 0.402595 -3.222119

C -3.596509 1.187338 -3.674094

C -1.319808 2.533586 -2.757162

C -0.263857 -2.189989 -2.293473

C -0.356054 -2.766758 -1.022099

C 0.441204 -3.844939 -0.660020

C 1.346905 -4.344859 -1.587692

C 1.473189 -3.791844 -2.855757

C 0.669071 -2.710828 -3.196850

Cl -1.483154 -2.130491 0.144715

H 0.356084 -4.281389 0.325499

Cl 2.354290 -5.703785 -1.146988

H 2.186391 -4.186516 -3.566035

Cl 0.849260 -1.995628 -4.772981

O -1.789966 2.905906 -4.048372

H -1.921128 2.978951 -1.955433

H -0.297344 2.897632 -2.672316

C -3.120778 2.499371 -4.398493

C -4.465932 1.694307 -2.535689

C -4.174212 3.560425 -4.007366

C -4.786978 3.039148 -2.729216

C -5.597913 3.701630 -1.811114

C -6.075199 3.003436 -0.701890

C -5.753743 1.656841 -0.516214

C -4.946757 0.987723 -1.436365

H -5.853173 4.747638 -1.951266

H -6.703627 3.510625 0.023017

H -6.139804 1.124755 0.346747

H -4.714548 -0.063969 -1.311982

H -3.705299 4.542567 -3.910911

H -4.934487 3.642040 -4.792180

H -3.070393 2.342458 -5.475358

H -4.157800 0.563510 -4.364427

C -3.732166 -2.186072 -3.714930

O -4.662366 -1.907307 -2.937278

C -3.054100 -3.517454 -3.678163

C -2.412984 -3.993488 -4.818351

C -1.801261 -5.241492 -4.836045

C -1.843408 -6.027619 -3.688894

C -2.495714 -5.574014 -2.540355

C -3.101766 -4.322430 -2.539244

O -2.361466 -3.218297 -5.955717

H -1.329203 -5.588156 -5.747900

H -1.386125 -7.010938 -3.698861

H -2.549822 -6.204322 -1.660544

H -3.620409 -3.952365 -1.662345

C -3.459077 -2.363609 -6.198082

C -4.649145 -3.232474 -6.731663

C -3.047243 -1.344506 -7.252354

O -3.917156 -1.677151 -5.048298

F -5.072804 -4.123651 -5.811800

F -5.700857 -2.465893 -7.056259

F -4.275407 -3.919674 -7.821633

C -3.815624 -0.190998 -7.437125

C -3.451875 0.740761 -8.405260

C -2.323437 0.528406 -9.195632

C -1.564193 -0.624767 -9.018118

C -1.926149 -1.563183 -8.053807

H -4.700764 -0.029416 -6.835896

H -4.057651 1.629839 -8.546897

H -2.041764 1.256033 -9.949749

H -0.687677 -0.799877 -9.633216

H -1.336277 -2.459936 -7.918390

C -8.142067 -2.211043 -3.486946

N -7.328925 -3.302741 -3.234217

N -7.431378 -1.013014 -3.397847

O -9.326221 -2.259766 -3.746802

H -6.352676 -3.102459 -3.038426

C -7.702873 -4.650023 -3.248879

H -6.414009 -1.051747 -3.390403

C -8.053812 0.218049 -3.617716

C -9.188844 0.621816 -2.904381

C -9.792813 1.849981 -3.144627

C -9.245870 2.733927 -4.066899

C -8.100267 2.370672 -4.760949

C -7.535043 1.124463 -4.543694

F -9.696877 -0.153353 -1.947214

F -10.879087 2.207907 -2.452569

F -9.807115 3.929425 -4.271440

F -7.543555 3.219854 -5.638632

F -6.426800 0.796186 -5.241308

C -6.688488 -5.606036 -3.114811

C -6.994028 -6.963278 -3.084858

C -8.308231 -7.402383 -3.198386

C -9.313215 -6.447654 -3.343067

C -9.032569 -5.085945 -3.366696

H -5.654978 -5.286276 -3.042970

C -5.867533 -7.945875 -2.917995

H -8.544589 -8.456793 -3.168548

C -10.736244 -6.904769 -3.531565

H -9.823602 -4.359960 -3.470562

F -6.292190 -9.224383 -2.905031

F -4.951684 -7.840873 -3.908681

F -5.193264 -7.742364 -1.756798

F -11.629939 -5.998409 -3.089062

F -11.016935 -7.127998 -4.836685

F -10.983576 -8.063949 -2.879988

A3-TS2_1-B_

C -1.788530 -1.702447 -2.364112

N -1.386077 -1.766665 -1.082218

N -0.798278 -0.596521 -0.610045

C -0.869137 0.192544 -1.634849

N -1.461299 -0.433553 -2.709879

C -1.668409 0.295647 -3.962439

C -0.308810 1.556723 -1.855439

C -1.469162 -2.877382 -0.194669

C -2.611571 -3.092847 0.580127

C -2.697813 -4.166232 1.457471

C -1.614687 -5.031442 1.561999

C -0.453858 -4.835304 0.822481

C -0.388651 -3.753070 -0.045499

Cl -3.966765 -1.995500 0.468711

H -3.589974 -4.320073 2.048633

Cl -1.714639 -6.392586 2.648841

H 0.383891 -5.512605 0.914456

Cl 1.071341 -3.499916 -0.960118

O 0.354442 1.540141 -3.116538

H -1.094271 2.321409 -1.815235

H 0.443163 1.786561 -1.102699

C -0.443809 1.236008 -4.266906

C -2.858196 1.238935 -3.924764

C -1.071081 2.503286 -4.895389

C -2.515186 2.483172 -4.455092

C -3.470024 3.494034 -4.534473

C -4.762461 3.242896 -4.075216

C -5.100456 1.994282 -3.548170

C -4.148231 0.978953 -3.471691

H -3.213434 4.467388 -4.941564

H -5.512795 4.025268 -4.126261

H -6.110488 1.812018 -3.196757

H -4.413590 0.008018 -3.066311

H -0.521398 3.392790 -4.577961

H -0.996125 2.455004 -5.987812

H 0.251500 0.742773 -4.944916

H -1.772243 -0.440876 -4.757884

C -2.358369 -3.353763 -3.448284

O -2.208020 -4.301826 -2.671452

C -3.709918 -2.938838 -3.938979

C -3.861958 -2.438341 -5.230061

C -5.110499 -2.068265 -5.721263

C -6.227204 -2.214912 -4.907356

C -6.099036 -2.738142 -3.618041

C -4.846145 -3.105226 -3.144136

O -2.771916 -2.263646 -6.056946

H -5.188435 -1.688015 -6.733089

H -7.204244 -1.934796 -5.286371

H -6.975432 -2.872722 -2.994099

H -4.730218 -3.520328 -2.150516

C -1.625142 -3.062151 -5.801387

C -0.451009 -2.411145 -6.521121

O -1.317001 -3.166872 -4.432738

C -1.872359 -4.497294 -6.377278

C 0.837156 -2.502133 -5.991574

C 1.911082 -1.923248 -6.663536

C 1.704885 -1.252146 -7.866370

C 0.419828 -1.165566 -8.399429

C -0.656010 -1.747589 -7.734648

H 1.002320 -3.016617 -5.055379

H 2.907945 -2.010601 -6.246252

H 2.543062 -0.804940 -8.390847

H 0.253341 -0.650401 -9.339832

H -1.651847 -1.683131 -8.153909

F -0.775795 -5.254910 -6.225172

F -2.889242 -5.125801 -5.757124

F -2.170025 -4.442135 -7.685261

C -1.832364 -7.726476 -2.304687

N -0.830231 -6.877155 -2.742089

N -2.979435 -7.013738 -1.946364

O -1.746385 -8.933624 -2.220265

H -1.057646 -5.886195 -2.778029

C 0.451951 -7.233761 -3.163259

H -3.060005 -6.051647 -2.262722

C -4.147777 -7.662804 -1.535573

C -5.361712 -7.395560 -2.174444

C -6.548578 -7.994450 -1.778372

C -6.538097 -8.911525 -0.734948

C -5.345230 -9.194735 -0.081127

C -4.169503 -8.558809 -0.461322

F -5.385404 -6.520663 -3.194235

F -7.694636 -7.707984 -2.407743

F -7.672309 -9.506402 -0.352512

F -5.340937 -10.053559 0.943798

F -3.064816 -8.794305 0.247346

C 1.290569 -6.194289 -3.594332

C 2.591401 -6.457732 -3.999175

C 3.091815 -7.758898 -3.997293

C 2.253357 -8.786730 -3.579734

C 0.945602 -8.545372 -3.162404

H 0.913955 -5.179126 -3.599468

C 3.453340 -5.331742 -4.497026

H 4.106222 -7.963564 -4.310346

C 2.775076 -10.198623 -3.512349

H 0.307280 -9.354357 -2.841229

F 1.865468 -11.096192 -3.943482

F 3.889261 -10.366634 -4.258212

F 3.097000 -10.548716 -2.245226

F 4.763807 -5.549134 -4.268124

F 3.317709 -5.136049 -5.829611

F 3.138481 -4.147003 -3.908390

A3-TS2_1-B-minor_

C -1.953852 -1.702337 -2.669438

N -1.539238 -1.821942 -1.393883

N -1.153981 -0.621472 -0.805067

C -1.363585 0.240474 -1.748545

N -1.851897 -0.367765 -2.883796

C -2.228150 0.450210 -4.037251

C -1.051074 1.697046 -1.825878

C -1.496350 -2.992981 -0.584593

C -2.604926 -3.366760 0.181188

C -2.570892 -4.485542 1.003728

C -1.399925 -5.232004 1.063662

C -0.271198 -4.878318 0.333305

C -0.328180 -3.753201 -0.478981

Cl -4.064999 -2.408669 0.138351

H -3.437647 -4.762883 1.587725

Cl -1.341723 -6.641299 2.092292

H 0.627798 -5.475018 0.384721

Cl 1.092866 -3.286481 -1.372328

O -0.429669 1.933422 -3.085711

H -1.955164 2.303989 -1.693839

H -0.329839 1.972334 -1.058265

C -1.187014 1.605477 -4.258567

C -3.549690 1.179024 -3.857409

C -2.034196 2.798788 -4.757872

C -3.434652 2.505988 -4.274674

C -4.538777 3.352631 -4.220077

C -5.749905 2.856405 -3.738427

C -5.858866 1.528015 -3.321610

C -4.756690 0.675917 -3.381489

H -4.459332 4.387310 -4.539641

H -6.615199 3.509130 -3.684301

H -6.807086 1.155219 -2.948925

H -4.845648 -0.356775 -3.060460

H -1.621029 3.738719 -4.384029

H -2.001662 2.847931 -5.852541

H -0.432863 1.302069 -4.982598

H -2.248354 -0.197609 -4.909574

C -2.281758 -3.303778 -3.903470

O -1.885598 -4.284674 -3.261470

C -3.733753 -3.103256 -4.235480

C -4.104723 -2.463416 -5.419576

C -5.442562 -2.240305 -5.733008

C -6.426822 -2.687964 -4.860184

C -6.078761 -3.361020 -3.685834

C -4.738829 -3.562730 -3.379826

O -3.145160 -2.033705 -6.312193

H -5.690142 -1.736693 -6.660414

H -7.471336 -2.528785 -5.105754

H -6.847450 -3.740144 -3.022771

H -4.454288 -4.067854 -2.464904

C -1.948512 -2.803720 -6.256551

C -2.141752 -4.192580 -6.859986

O -1.414725 -2.820188 -4.951617

C -0.924103 -1.987035 -7.093598

C -1.289352 -5.233048 -6.487847

C -1.423565 -6.489857 -7.070982

C -2.406503 -6.713420 -8.032230

C -3.248664 -5.671896 -8.417087

C -3.113655 -4.412716 -7.840011

H -0.519262 -5.054982 -5.751874

H -0.755736 -7.290677 -6.772600

H -2.514249 -7.694773 -8.482477

H -4.012380 -5.838522 -9.169521

H -3.763146 -3.603240 -8.149349

F 0.269182 -2.589047 -7.106039

F -1.345805 -1.850067 -8.358551

F -0.747600 -0.744125 -6.592810

C -0.696034 -7.532794 -2.799624

N -2.025703 -7.155180 -2.847331

N 0.159276 -6.445515 -2.978779

O -0.290240 -8.663535 -2.622345

H -0.230721 -5.539423 -3.224095

C 1.550650 -6.624673 -3.001080

C 2.300754 -6.286946 -4.128896

C 3.679824 -6.441900 -4.168688

C 4.341113 -6.975559 -3.069781

C 3.619705 -7.327558 -1.935186

C 2.244882 -7.138036 -1.902241

F 1.680798 -5.797666 -5.214353

F 4.367584 -6.102475 -5.263247

F 5.665462 -7.140092 -3.099541

F 4.257729 -7.817415 -0.867974

F 1.593384 -7.424723 -0.769207

H -2.216917 -6.172072 -3.024831

C -3.139401 -7.984140 -2.692772

C -4.398160 -7.419098 -2.934882

C -5.557621 -8.165931 -2.756286

C -5.495594 -9.495510 -2.353151

C -4.240488 -10.059331 -2.131634

C -3.068049 -9.327128 -2.290399

H -4.467262 -6.391381 -3.273150

C -6.880138 -7.490153 -2.991280

H -6.396941 -10.075103 -2.211893

C -4.139164 -11.515572 -1.758218

H -2.104935 -9.776711 -2.104701

F -3.094168 -11.761822 -0.942704

F -5.251826 -11.955251 -1.129077

F -3.976096 -12.297367 -2.850742

F -7.925009 -8.328100 -2.855306

F -7.078563 -6.466892 -2.116997

F -6.959168 -6.946579 -4.227018

A3-TS2_2-A_

C -1.453124 -1.597812 -2.860133

N -1.219905 -0.313457 -3.190250

N -0.952452 0.518181 -2.107043

C -1.013730 -0.296081 -1.102212

N -1.313798 -1.578001 -1.509721

C -1.351043 -2.667703 -0.523498

C -0.937749 -0.032014 0.363718

C -1.174359 0.260005 -4.490957

C 0.033181 0.316845 -5.196518

C 0.104732 0.903734 -6.453483

C -1.048972 1.455115 -6.996693

C -2.258502 1.442503 -6.313110

C -2.309223 0.849553 -5.057137

Cl 1.485890 -0.327656 -4.486455

H 1.039816 0.922671 -6.994849

Cl -0.974855 2.200565 -8.577032

H -3.144277 1.891158 -6.740970

Cl -3.812129 0.875132 -4.177824

O -2.007077 -0.746965 0.973081

H 0.037293 -0.326877 0.771177

H -1.097810 1.024992 0.569194

C -1.967420 -2.172190 0.840161

C 0.028103 -3.174967 -0.148475

C -1.107433 -2.849087 1.935912

C 0.158439 -3.280166 1.236117

C 1.344870 -3.757946 1.788510

C 2.388911 -4.122635 0.939674

C 2.249826 -4.017941 -0.446673

C 1.062739 -3.545206 -1.002682

H 1.458444 -3.845592 2.864749

H 3.317580 -4.495459 1.359338

H 3.066460 -4.311845 -1.097196

H 0.950605 -3.474875 -2.078356

H -0.941473 -2.155089 2.764235

H -1.643717 -3.710327 2.347770

H -3.012944 -2.467011 0.916032

H -1.951054 -3.465224 -0.955063

C -2.008768 -3.329009 -3.878840

O -1.722309 -4.241371 -3.093348

C -3.423527 -2.964055 -4.203277

C -3.761667 -2.593209 -5.504622

C -5.073344 -2.271950 -5.843816

C -6.061316 -2.330505 -4.869391

C -5.747134 -2.723866 -3.566041

C -4.436009 -3.046334 -3.244458

O -2.815522 -2.528633 -6.502751

H -5.299839 -1.997793 -6.867691

H -7.084591 -2.083151 -5.131139

H -6.522601 -2.786799 -2.811023

H -4.183629 -3.364830 -2.239331

C -1.618831 -3.259013 -6.302207

C -0.566547 -2.732229 -7.267525

O -1.121615 -3.145995 -4.990044

C -1.899824 -4.766253 -6.639886

C 0.788915 -2.877784 -6.966220

C 1.754031 -2.439907 -7.869335

C 1.372426 -1.857630 -9.076272

C 0.019355 -1.714351 -9.377781

C -0.949446 -2.155446 -8.480450

H 1.088993 -3.318551 -6.025358

H 2.804465 -2.560792 -7.626220

H 2.125799 -1.520952 -9.781204

H -0.285323 -1.260894 -10.314980

H -1.999652 -2.046779 -8.717887

F -0.777269 -5.495542 -6.495869

F -2.836957 -5.314157 -5.843615

F -2.323436 -4.896567 -7.905923

C -0.255850 -7.326791 -2.411857

N 0.120472 -6.578382 -3.527798

N -1.430290 -6.832814 -1.865080

O 0.367344 -8.280218 -1.997412

H -1.833545 -6.024968 -2.328205

C -2.005749 -7.181771 -0.646066

H -0.271748 -5.645569 -3.624244

C 1.237637 -6.897395 -4.301666

C 1.437435 -8.169082 -4.850229

C 2.551420 -8.447006 -5.633441

C 3.467690 -7.446055 -5.933519

C 3.271814 -6.167312 -5.429000

C 2.182681 -5.916828 -4.609596

F 0.539672 -9.135610 -4.661031

F 2.723453 -9.672037 -6.138785

F 4.522022 -7.708092 -6.711386

F 4.135988 -5.185951 -5.721026

F 2.022859 -4.669573 -4.118758

C -3.095292 -6.412391 -0.214168

C -3.712077 -6.674089 1.002694

C -3.275183 -7.717477 1.813975

C -2.204545 -8.492169 1.371901

C -1.564862 -8.241471 0.161270

H -3.465276 -5.613165 -0.846372

C -4.811367 -5.760155 1.463344

H -3.763958 -7.932516 2.754303

C -1.683368 -9.597111 2.254952

H -0.739607 -8.853424 -0.169647

F -1.158718 -10.614463 1.545759

F -0.708615 -9.149101 3.081040

F -2.652669 -10.112172 3.043304

F -5.617929 -6.330901 2.374094

F -4.304945 -4.632959 2.043570

F -5.587272 -5.333436 0.441241

A3-TS2_2-A-minor_

C -2.342440 -1.873627 -3.163743

N -2.759679 -0.592113 -3.217032

N -2.539203 0.126086 -2.045268

C -1.959427 -0.742965 -1.280788

N -1.821446 -1.958089 -1.913775

C -1.257869 -3.091839 -1.169983

C -1.587796 -0.668486 0.160860

C -3.507697 0.065129 -4.235240

C -2.887400 0.922407 -5.150816

C -3.631282 1.608911 -6.104374

C -5.010133 1.444615 -6.123901

C -5.662139 0.628691 -5.207891

C -4.903669 -0.047655 -4.260887

Cl -1.167555 1.163087 -5.103747

H -3.139028 2.263653 -6.810147

Cl -5.953179 2.296679 -7.326134

H -6.738274 0.526596 -5.219960

Cl -5.722229 -1.017040 -3.067767

O -2.157781 -1.804162 0.803725

H -0.499666 -0.627494 0.288214

H -2.030991 0.212926 0.620957

C -1.790240 -3.100573 0.306404

C 0.251379 -3.069443 -1.010105

C -0.631831 -3.717074 1.119973

C 0.600165 -3.448560 0.287985

C 1.940700 -3.564647 0.643578

C 2.920532 -3.296298 -0.312171

C 2.565302 -2.912917 -1.606635

C 1.223143 -2.798340 -1.967534

H 2.222728 -3.862798 1.648793

H 3.968875 -3.391943 -0.049695

H 3.336343 -2.717458 -2.343763

H 0.949079 -2.506837 -2.972927

H -0.599493 -3.280977 2.121031

H -0.792218 -4.794762 1.242991

H -2.708814 -3.682047 0.378751

H -1.588064 -3.991311 -1.684115

C -2.401943 -3.519094 -4.328302

O -2.670563 -4.444993 -3.538319

C -3.354558 -3.125901 -5.417561

C -2.882138 -2.398523 -6.509218

C -3.731309 -1.988046 -7.529573

C -5.077582 -2.335312 -7.462312

C -5.560503 -3.099633 -6.398158

C -4.699308 -3.493392 -5.380181

O -1.542404 -2.084375 -6.571784

H -3.329124 -1.424563 -8.363374

H -5.748139 -2.026464 -8.257254

H -6.605620 -3.386756 -6.364712

H -5.057583 -4.084588 -4.546711

C -0.716258 -3.132338 -6.079978

C -0.726321 -4.337736 -7.018563

O -1.029018 -3.484851 -4.747494

C 0.701871 -2.503757 -5.998546

C -0.635935 -5.634650 -6.512704

C -0.614356 -6.725311 -7.378501

C -0.676114 -6.526682 -8.755008

C -0.756266 -5.231854 -9.264779

C -0.777641 -4.140751 -8.402242

H -0.585924 -5.788019 -5.443374

H -0.548068 -7.729846 -6.974319

H -0.659618 -7.376839 -9.429119

H -0.801149 -5.070204 -10.336753

H -0.834497 -3.135434 -8.801156

F 0.731343 -1.463579 -5.144615

F 1.590974 -3.409534 -5.565060

F 1.104314 -2.057354 -7.197322

C -3.053890 -7.040954 -1.370559

N -4.094313 -6.226920 -1.821821

N -1.890605 -6.818221 -2.091096

O -3.173805 -7.830539 -0.457133

H -3.860288 -5.446410 -2.431076

C -5.341897 -6.204543 -1.189697

H -1.928649 -6.086889 -2.797146

C -0.640303 -7.406695 -1.869926

C 0.461455 -6.839749 -2.533321

C 1.728380 -7.382477 -2.382916

C 1.938238 -8.493113 -1.564251

C 0.847330 -9.043943 -0.904485

C -0.437991 -8.519987 -1.047178

H 0.323155 -5.964851 -3.157555

C 2.909916 -6.768601 -3.085407

H 2.926361 -8.918902 -1.451136

C 1.038372 -10.213290 0.026452

H -1.276178 -8.962090 -0.530403

C -6.121971 -7.352632 -1.015958

C -7.359225 -7.288334 -0.385442

C -7.867593 -6.067157 0.041306

C -7.125403 -4.908363 -0.150323

C -5.876600 -4.992180 -0.746949

F -5.707054 -8.529944 -1.481264

F -8.085868 -8.397973 -0.223627

F -9.067781 -6.005970 0.624261

F -7.605849 -3.727071 0.252956

F -5.156186 -3.863120 -0.904405

F 0.080534 -11.149715 -0.136282

F 2.229529 -10.822470 -0.155235

F 0.988264 -9.826849 1.322246

F 3.797104 -6.245945 -2.206407

F 3.587867 -7.686137 -3.811762

F 2.555016 -5.776679 -3.927124

A3-TS2_2-B_

C 0.479899 -0.983674 -0.231021

N 1.511646 -1.339983 -1.018949

N 1.337950 -2.556691 -1.665198

C 0.167747 -2.935132 -1.266108

N -0.388933 -2.017933 -0.399492

C -1.722234 -2.287132 0.153277

C -0.551274 -4.222914 -1.479543

C 2.752207 -0.675183 -1.249177

C 2.892236 0.227234 -2.308326

C 4.123651 0.800618 -2.605469

C 5.226084 0.449846 -1.835558

C 5.123804 -0.453538 -0.783576

C 3.886124 -1.017807 -0.503732

Cl 1.520107 0.641397 -3.295223

H 4.214973 1.509239 -3.416232

Cl 6.780163 1.155152 -2.200430

H 5.990518 -0.720318 -0.194736

Cl 3.771547 -2.175370 0.789509

O -0.990841 -4.674344 -0.200899

H -1.385434 -4.105476 -2.181123

H 0.131625 -4.976956 -1.866503

C -1.878123 -3.810301 0.516813

C -2.861440 -2.026417 -0.817868

C -3.361941 -4.125874 0.219493

C -3.789085 -3.068509 -0.769660

C -4.933343 -3.021514 -1.561158

C -5.132304 -1.924138 -2.398650

C -4.202562 -0.882974 -2.439307

C -3.057921 -0.923974 -1.643781

H -5.658735 -3.829076 -1.534608

H -6.016942 -1.879437 -3.025615

H -4.369797 -0.033311 -3.092609

H -2.345597 -0.108723 -1.670809

H -3.462100 -5.149448 -0.149287

H -3.951906 -4.058563 1.141283

H -1.623510 -3.979793 1.562997

H -1.831471 -1.690954 1.055910

C 0.648836 0.551509 1.035436

O 1.829564 0.501774 1.421346

C -0.490634 0.272499 1.975561

C -1.754052 0.803414 1.711094

C -2.844346 0.512589 2.526137

C -2.660647 -0.318237 3.627332

C -1.397197 -0.839200 3.920657

C -0.316352 -0.540539 3.097441

O -1.940094 1.600215 0.606789

H -3.811359 0.941693 2.290845

H -3.504623 -0.547004 4.269520

H -1.257784 -1.472192 4.789996

H 0.666704 -0.949553 3.301920

C -0.797703 2.396464 0.297688

C -1.100773 2.974761 -1.112281

C -0.620564 3.511191 1.327585

O 0.360558 1.617281 0.113353

F -2.217656 3.720582 -1.099584

F -0.087431 3.738995 -1.532815

F -1.284002 1.993285 -2.017357

C 0.651203 3.935797 1.710921

C 0.799550 4.969331 2.633032

C -0.322895 5.591869 3.171249

C -1.596413 5.177857 2.783631

C -1.746776 4.144359 1.865194

H 1.524528 3.452277 1.298214

H 1.794297 5.283565 2.929911

H -0.206980 6.396756 3.889619

H -2.475698 5.660357 3.197619

H -2.738076 3.827776 1.565218

C 4.437397 1.925581 3.133379

N 3.487430 1.075279 3.698184

N 4.096675 2.264166 1.831204

O 5.422142 2.315479 3.724880

H 3.280692 1.796072 1.441026

C 4.724629 3.211721 1.018399

H 2.804223 0.646037 3.077880

C 3.615238 0.557827 4.988811

C 3.441553 -0.811659 5.210620

C 3.519544 -1.373197 6.475839

C 3.806146 -0.562850 7.567020

C 3.976088 0.803028 7.378881

C 3.853390 1.360424 6.111197

F 3.160770 -1.613957 4.164815

F 3.337340 -2.688445 6.644831

F 3.898946 -1.091745 8.790863

F 4.213649 1.589930 8.433126

F 3.925932 2.686311 5.998798

C 5.895339 3.893982 1.370776

C 6.429316 4.841603 0.497468

C 5.833534 5.129853 -0.723381

C 4.669597 4.443164 -1.070036

C 4.118382 3.498123 -0.217299

H 6.367726 3.684699 2.318713

C 7.689349 5.553158 0.917844

H 6.256182 5.873876 -1.384425

C 4.036656 4.706124 -2.408576

H 3.205299 2.987260 -0.499929

F 2.751590 4.301710 -2.462966

F 4.065875 6.013401 -2.736316

F 4.690314 4.047662 -3.405363

F 8.086562 6.472405 0.012281

F 7.530041 6.197591 2.094538

F 8.716545 4.690736 1.084423

A3-TS2_2-B-minor_

C -1.101385 -1.654715 -2.930358

N -1.001984 -0.340988 -3.220171

N -0.636656 0.464969 -2.140833

C -0.504653 -0.399151 -1.179567

N -0.772397 -1.680391 -1.612328

C -0.534456 -2.811380 -0.706871

C -0.223610 -0.204740 0.275882

C -1.128103 0.273804 -4.495601

C -0.054947 0.257433 -5.400978

C -0.151028 0.884093 -6.641221

C -1.329416 1.554036 -6.961231

C -2.404337 1.612907 -6.077651

C -2.291933 0.972503 -4.846093

Cl 1.438963 -0.534328 -4.978808

H 0.674931 0.843754 -7.339549

Cl -1.460030 2.355849 -8.510434

H -3.309797 2.147231 -6.336071

Cl -3.627112 1.063663 -3.729916

O -1.110596 -1.053186 0.998818

H 0.830005 -0.416916 0.506444

H -0.447209 0.821360 0.573487

C -0.994570 -2.460514 0.754269

C 0.943055 -3.132326 -0.559931

C 0.059576 -3.137943 1.667298

C 1.274604 -3.319121 0.785626

C 2.584188 -3.640765 1.142258

C 3.550053 -3.762266 0.139583

C 3.212943 -3.568070 -1.204386

C 1.900894 -3.253466 -1.564553

H 2.854037 -3.788190 2.184958

H 4.574165 -4.007578 0.407023

H 3.968262 -3.660853 -1.978411

H 1.642681 -3.097427 -2.607636

H 0.238627 -2.527085 2.558622

H -0.321428 -4.108207 2.014150

H -1.998244 -2.845407 0.943983

H -1.091444 -3.662659 -1.096421

C -1.852544 -3.360006 -3.898205

O -1.432337 -4.298112 -3.196161

C -3.297086 -2.972774 -3.921130

C -3.877695 -2.554533 -5.122176

C -5.223186 -2.193409 -5.185989

C -6.000206 -2.267666 -4.033353

C -5.446263 -2.722188 -2.830823

C -4.103164 -3.081391 -2.781958

O -3.146871 -2.489862 -6.286759

H -5.640657 -1.878820 -6.136903

H -7.048970 -1.988324 -4.078060

H -6.062623 -2.801534 -1.940657

H -3.667621 -3.460998 -1.862740

C -1.942425 -3.233052 -6.335314

C -1.098784 -2.695807 -7.482502

O -1.192400 -3.140266 -5.140888

C -2.290619 -4.732801 -6.615959

C 0.280904 -2.922242 -7.499500

C 1.053089 -2.460531 -8.565424

C 0.450471 -1.780509 -9.625206

C -0.929733 -1.567441 -9.616123

C -1.705100 -2.027242 -8.551735

H 0.757197 -3.451319 -6.684888

H 2.124153 -2.639652 -8.558787

H 1.051550 -1.423933 -10.457526

H -1.406783 -1.043397 -10.439469

H -2.776026 -1.862430 -8.546440

F -1.168545 -5.474550 -6.628725

F -3.112793 -5.258879 -5.684930

F -2.897546 -4.856110 -7.807413

C -0.532380 -7.444357 -2.321029

N 0.104729 -6.690752 -3.292635

N -1.804467 -6.927441 -2.043141

O -0.072531 -8.431289 -1.770988

H -1.993643 -5.973098 -2.348173

C -2.587818 -7.419660 -0.995756

H -0.365937 -5.826302 -3.561767

C 1.450728 -6.762473 -3.654609

C 2.287644 -7.855883 -3.373482

C 3.622470 -7.814978 -3.773297

C 4.156172 -6.713994 -4.447682

C 3.315720 -5.638018 -4.726829

C 1.976019 -5.661374 -4.346445

H 1.889709 -8.710027 -2.843964

C 4.533306 -8.960553 -3.411350

H 5.195682 -6.699615 -4.749492

C 3.832547 -4.407989 -5.417986

H 1.329514 -4.816814 -4.566952

C -3.093218 -6.546131 -0.025062

C -3.910075 -6.975713 1.013332

C -4.223461 -8.328066 1.119679

C -3.733745 -9.222633 0.171222

C -2.947119 -8.770783 -0.886551

F -2.787008 -5.227438 -0.098948

F -4.371496 -6.101241 1.917328

F -4.997657 -8.762322 2.119745

F -4.057719 -10.517049 0.255587

F -2.568576 -9.649915 -1.813438

F 3.182156 -4.171820 -6.586600

F 3.644556 -3.295324 -4.653490

F 5.145814 -4.477964 -5.700054

F 5.513379 -9.127579 -4.329039

F 5.145353 -8.749260 -2.222277

F 3.866780 -10.128149 -3.311396
